# A Ferroptosis-Related Prognostic Risk Score Model to Predict Clinical Significance and Immunogenic Characteristics in Glioblastoma Multiforme

**DOI:** 10.1155/2021/9107857

**Published:** 2021-11-09

**Authors:** Dongdong Xiao, Yujie Zhou, Xuan Wang, Hongyang Zhao, Chuansheng Nie, Xiaobing Jiang

**Affiliations:** Department of Neurosurgery, Union Hospital, Tongji Medical College, Huazhong University of Science and Technology, Wuhan 430022, China

## Abstract

**Background:**

Ferroptosis is a recently identified cell death pathway, and the susceptibility to ferroptosis inducers varies among cancer cell types. There have been recent attempts to clarify the mechanisms implicated in ferroptosis, glioma invasion, and the immune microenvironment but little is known about ferroptosis regulation in GBM.

**Methods:**

Screening ferroptosis-related genes from published reports and existing databases, we constructed an integrated model based on the RNA-sequencing data in GBM. The association of FRGPRS and overall survival is identified and validated across several different datasets. Genomic and clinical characteristics, immune infiltration, enriched pathways, pan-cancer, drug resistance, and immune checkpoint inhibitor therapy are compared among various FRGPRS subgroups.

**Results:**

We identified and confirmed the influences of five ferroptosis key hub genes in the FRGPRS model. The FRGPRS model could serve to predict overall survival and progression-free survival in GBM patients, and high FRGPRS was associated with comparatively stronger immunity, higher proportions of tumour tissue, and good cytolytic immune and chemotherapeutics response in GBM patients.

**Conclusions:**

The five ferroptosis key hub genes constituting the FRGPRS model could serve to predict overall survival and progression-free survival in patients with GBM and help guide timely and efficacious therapeutic strategies customised and optimised for each individual patient. This discovery may lay the foundation for the development and optimisation of other iterations of this model for the improved forecasting, detection, and treatment of other malignancies notorious for their drug resistance and immune escape.

## 1. Introduction

Glioblastoma multiforme (GBM) is a primary malignant brain tumour. Despite the fact that it is treated with multidisciplinary synthetic therapy, including surgical resection, radiotherapy, and chemotherapy, the patients' overall survival time is only approximately 15 months [1, 2]. Tumour necrosis is common in GBM, and it is positively correlated with tumour aggressiveness and poor outcome [3, 4]. Previous studies proposed that oxidative phosphorylation disorders and intracellular adenosine triphosphate (ATP) depletion lead to cell death in chronic ischaemia microenvironments [5, 6]. Extensive tumour tissue hypoxia together with rapid tumour expansion triggers necrosis. Collectively, they comprise the fundamental stimuli of GBM stem cell progression [7]. Intense research efforts have elucidated the cell death pathways in other cancers. However, no such breakthrough has been made for GBM. Moreover, the mechanisms by which GBM escapes programmed cell death remain unclear [8–10]. Recent studies have demonstrated that targeting the cell death pathway is a promising therapeutic strategy for preventing the progression of GBM. For example, cell death-targeting drugs combined with immunotherapy suppressed tumour growth in murine GBM models [11]. However, chemoradiotherapy resistance and immune evasion are extremely variable among GBM patients. Molecular alterations, such as isocitrate dehydrogenase1 (*IDH1*) mutation and tumour protein p53 (*TP53*) mutation, are widely utilised for the prognosis and treatment of GBM. Nevertheless, few of these strategies have been successful. Therefore, new, efficacious treatments for GBM are urgently required [12, 13].

Ferroptosis is a recently identified cell death pathway characterised by iron-dependent lipid peroxidation. It differs from apoptosis, necroptosis, and pyroptosis [14]. Overloading of intracellular iron ions leads to glutathione (GSH) depletion, reactive oxygen species (ROS) accumulation, and, ultimately, cell death [15, 16]. Chemotherapy-resistant GBM and other cancer cells, especially those that are mesenchymal and metastatic, are relatively more sensitive to ferroptosis induced by glutathione peroxidase-4 (GPX4) inhibition [17–19]. However, susceptibility to ferroptosis inducers varies among cancer cell types [20]. Despite the presence of continuous oxidative stress stimulation, ferroptosis is not always triggered during cancer progression [21]. There have been recent attempts to clarify the mechanisms implicated in ferroptosis, glioma invasion, and the immune microenvironment [22–25]. Unfortunately, these studies have not specifically focused on GBM, and little is known about ferroptosis regulation in GBM. Thus, ferroptosis-related prognosis and treatment indicators for GBM are promptly needed.

Now, the treatment of GBM has entered an era of the comprehensive treatment, therefore, identifying optimal biomarkers is the key to maximizing the comprehensive therapeutic effect. In the present study, we constructed a model which consists of 5 ferroptosis regulators and proposed it as a potential molecular classification for GBM, which could serve to predict overall survival and progression-free survival in patients with GBM and could identify distinct mutation pattern, immune infiltration, cytolytic immune response, and the drug resistance. This discovery may lay the foundation for the development and optimisation of other iterations of this model for the improved forecasting, detection, and treatment of other malignancies notorious for their drug resistance and immune escape.

## 2. Methods

### 2.1. Patients and Datasets

We download from cBioPortal database (https://www.cbioportal.org/) TCGA malignant glioblastoma (glioblastoma multiforme, GBM), genome sequencing data (whole exome sequencing, WES, 388 samples), copy number variation data (SNP6.0 chip data, HG19, 575 samples), transcriptome data (RNA-SEQ, 155 samples), and clinical information data (585 samples). The sample size of intersection of transcriptome data and clinical data was 155. Rna-seq data included RSEM standardized count and *Z*-score standardized expression profile. We download a set of validation set of data (GSE4412), including the transcriptome data and clinical data, from the GEO resource platform (https://www.ncbi.nlm.nih.gov/gds). Microarray data of GPL96 transcriptome sequencing platform (Affymetrix Human Genome U133A Array) were selected, including 85 samples of right frontal, right frontal parietal, right frontal temporal, right parietal, right parietal occipital, right temporal, right temporal parietal, right anterior temporal, right cerebellum, left frontal, left frontal temporal, left parietal, left parietal occipital, left temporal, left temporal parietal, and thalamus. 74 patients were diagnosed with grade III (*n* = 24) or grade IV (*n* = 50) gliomas during the initial surgical treatment and were provided with fresh frozen materials for analysis as part of the study. Normal brain tissue RNA-sequencing expression data for 1,671 patients were obtained from the GTEx project (https://gtexportal.org/home/). RNA-sequencing data for 514 low-grade glioma (LGG) samples and 407 bladder urothelial carcinoma (BLCA) samples and their corresponding survival information were downloaded from the cBioportal database (https://www.cbioportal.org/) for pan-cancer analyses of ferroptosis-related risk factors. Clinical information and RNA-sequencing data for 298 patients with urothelial carcinoma being administered the PD-L1 inhibitor atezolizumab were extracted using the “IMvigor210CoreBiologies” package in R4.0.3 (R Core Team, Vienna, Austria). For patients with GBM, clinical data, including radiotherapy, race, and ethnicity, were downloaded from the Xena data resources (https://xenabrowser.net/datapages/?cohort=GDC%20TCGA%20Glioblastoma%20(GBM)&removeHub=https%3A%2F%2Fxena.treehouse.gi.ucsc.edu%3A443). The intersection of the transcriptome and clinical data was 155. Data type and sample size information are summarised in Table 1.

Two hundred and sixty-nine ferroptosis genes were obtained from known studies and related databases. There were 259 in the FerrDb database (http://www.zhounan.org/ferrdb/), 60 in the study by Yee et al. [5], and 52 in the study by Liang et al. [26]. Data regarding the interactions among transcription factors (TF), mRNAs, miRNAs, and lnRNAs were downloaded from the Transfac (http://gene-regulation.com/), Chipbase (http://rna.sysu.edu.cn/chipbase/), miTarbase (http://mirtarbase.cuhk.edu.cn/php/index.php), Starbase (http://starbase.info/), and LncMAP (http://www.bio-bigdata.com/LncMAP) datahttp://2fxena.treehouse.gbases. The protein–protein interaction (PPI) network of the coding genes was constructed using STRING (V11∙0; https://string-db.org/cgi/input.pl). Clinical and phenotypic data for TCGA GBM samples matching the transcriptome data were sorted as shown in Table 2.

### 2.2. Identification of Ferroptosis-Related Hub Genes

For the RNA-sequencing data, genes not expressed in more than five samples were excluded. Log2-transformation was performed for both the control and validation groups, namely, 155 tumours (expression data) in TCGA database vs. 155 samples (expression data) in the GTEx. After gene overlap of the control and validation groups, expression levels were obtained for 13,762 genes in tumour and normal tissues. Batch effects were removed using the ComBat function in the “sva” package of R. A differentially expressed gene (DEG) analysis between the GBM and normal brain samples was performed using the “DESeq2” package in R. The false discovery rate- (FDR-) corrected threshold for statistical significance was *p* ≤ 0∙01 (Benjamini and Hochberg method; FC ≥ 2 or FC ≤ 1/2). Instead of using log2FC, we use FC directly to represent the threshold. Differential expression between tumour tissues and normal tissues was divided into two types: upregulation group (FC ≥ 2) and downregulation group (FC ≤ 0.5).

A Kyoto Encyclopedia of Genes and Genomes (KEGG) function enrichment analysis was performed on the DEGs using the “clusterProfiler” package in R to identify significantly (*p* ≤ 0∙01) enriched pathways. Differentially ferroptosis-related (ferroptosis-DE) genes were selected among the DEGs and used in subsequent Gene Ontology (GO) functional and KEGG pathway enrichment analyses using MSigDB (V7∙2) (http://www.gsea-msigdb.org/gsea/msigdb). The top 15 significantly enriched GO terms and pathways were determined, and the related genes were extracted as Candidate-Ferroptosis-Geneset1 (cd-Ferr-Geneset1).

Ferroptosis-DE genes were screened out from among the DEGs. Consensus clustering analysis was performed using the “ConsensusClusterPlus” package in R to discover the ferroptosis-DE gene-based clusters in patients with GBM. Relative changes in the area under the cumulative distribution function (CDF) curve were evaluated for cluster number *k* in the range of two to ten. The optimal number of categories was determined to be four as the area under the CDF curve underwent the greatest changes between classes 4 and 5. The differences in survival among the four subcategories were evaluated using a log-rank test. The “survival” package in R was used to plot Kaplan–Meier (K–M) survival curves. For all GBM patients within the four categories, differential expression analyses were performed using the “DESeq2” package in R (FDR ≤ 0∙05; Benjamini and Hochberg method; FC ≥ 1∙5 or FC ≤ 2/3). The Candidate-Ferroptosis-Geneset2 (cd-Ferr-geneset2) was obtained by the intersection of DEGs among the four categories. The cd-Ferr-Geneset2 expression levels in the four categories were plotted with a heatmap using the “heatmap” package in R. A principal component analysis (PCA) was conducted using the “psych” package in R.

A weighted correlation network analysis (WGCNA) of the expression levels in the gene set collection was performed to screen for hub genes. Mean connectivity was used to select soft thresholds. A hierarchical cluster tree was plotted to reflect the significance levels of the hub genes and their associations with the clinical phenotypes. An association analysis was conducted to evaluate the correlations between the module genes and the clinical phenotypic data. The modules were identified, and their threshold values were ≥0∙7 and ≥0∙2 for gene significance (GS) and module membership (MM), respectively. Genes in the PPI networks with degree > 5 were designated hub genes. The intersection of the key module and hub genes in the PPI network was designated as the ferroptosis-related disease hub gene dataset. Based on the regulatory factors of screened key hub genes, a multifactor regulatory network was constructed using the Cytoscape (https://cytoscape.org/download.html).

### 2.3. Ferroptosis-Related Gene Prognostic Risk Score (FRGPRS) Construction and Validation

Based on the median expression values of the disease hub and known ferroptosis genes, the patients with GBM were separated into two groups. Thirteen prognosis-related core genes significantly influencing progression-free survival (PFS) were recognised with univariate Cox regression models (*p* ≤ 0∙01; log-rank test) and screened and verified using least absolute shrinkage and selection operator- (Lasso-) logistic regression analysis. FRGPRS was constructed based on the prognostic gene expression levels using the regression coefficient from the multivariate Cox proportional hazards regression analysis. FRGPRS of the *i*^th^ sample was calculated as follows:

(1) $$\begin{array}{c} \text{Risk scor}{\text{e}}_{i}\;=\;\overset{n}{\underset{j=1}{\sum }}C_{j}*\exp_{ij}, \end{array}$$

where *C*_*j*_ is the regression coefficient of the *j*^th^ prognostic factor in the Cox regression model, and exp_*ij*_ is the expression level of the *j*^th^ prognostic factor in the *i*^th^ sample.

The patients with GBM were separated into two groups based on their median FRGPRS values. The relationship between FRGPRS and patients' overall survival (OS) was evaluated using log-rank test. ROC curves were plotted using the “timeROC” package of R and used to estimate the prognostic performance of the FRGPRS. GEO data (GSE4412) were used for validation analysis. Based on the FRGPRS, pan-cancer analyses were performed on TCGA GBM, GEO GBM, TCGA LGG, and TCGA BLCA.

### 2.4. Comprehensive Analysis of Genomic, Clinical, and Immune Characteristics, Pan-Cancer, Drug Resistance, and Immune Checkpoint Inhibitor Therapy among Various FRGPRS Subgroups

The relationships among FRGPRS level and clinical (gender, radiotherapy, and age) and genomic (*IDH1* mutation, *1p/19q* codeletion, and *TP53* mutation status) characteristics were examined. Moreover, the patients with GBM were divided into high-risk and low-risk groups based on their median FRGPRS values. Correlation analyses were performed on the immune characteristics and genome variants among the different groups. The immune subtypes come from previous studies on immune characteristics analysis of TCGA data [27]. For immune subtypes, gliomas involve only 3 subtypes, namely, C1, C4, and C5. The homologous recombination deficiency (HRD) scores, neoantigens, fractions altered, and mRNAsi indices were assessed according to previous analyses of the genomic characteristics of TCGA data [27, 28]. Nonsynonymous GBM tumour mutation burdens were calculated using the tumour mutational burden (TMB) analysis. Chromosomal instability was associated with HRD, and genomic DNA damage was assessed by loss-of-heterozygosity, large scale transition, and telomeric allelic imbalance (NtAI) [29]. The infiltration levels of 22 different immunocytes were determined with the “CIBERSORT” package in R [30]. The Wilcoxon rank-sum test compares significance between pairs, while the KW test is a nonparametric test that compares multiple groups. Based on the proportions of the stromal and cellular components, the immune, stromal, and ESTIMATE scores were evaluated using the “ESTIMATE” package in R [31]. Based on GBM cell line and drug response data derived from the Genomics of Drug Sensitivity in Cancer website (http://www.cancerrxgene.org/), a drug sensitivity prediction model for the patients with GBM was constructed using ridge regression [32].

Survival analyses of FRGPRS groups were conducted to explore the predictive performance of FRGPRS in patients undergoing immune checkpoint inhibitor therapy. Based on their immunotherapy responses, the 298 aforementioned patients with urothelial carcinoma were divided into complete response (CR), partial response (PR), stable disease (SD), and progressive disease (PD) groups for validation analysis [33].

### 2.5. Genomic Variant and Copy Number Variation Analyses

To establish the differences in variation between low and high FRGPRS, mutation spectra were plotted using the “Maftools” package in R for the top 30 genes with highest frequency. The copy number alteration (CNA) frequency was calculated using the “copynumber” package in R. The log2CNA thresholds were set to ±0∙3, namely, -0∙3 for loss and 0∙3 for gain. A Wilcoxon test was used to plot the CNA frequency distribution graphs [34].

### 2.6. Individualized Prognostic Prediction Models

During the quantification of the risk on individuals in a clinical setting with the integration of multiple risk factors, the nomogram acts as a powerful tool in the assessment. A nomogram was constructed using the survival rate and “RMS” R package, and a correction curve was drawn to evaluate the consistency between the actual and predicted survival rates.

### 2.7. Statistics

A Wilcoxon rank-sum test was used to calculate the significance levels in pairs of groups. A Kruskal–Wallis test was used to compare the gene expression levels between two or more groups. The Benjamini and Hochberg method was used to correct the significance levels. Univariate and multivariate logistic regression models were applied to calculate the hazard ratios (HRs). Predictive performance of the model was evaluated by ROC curve analysis. *p* ≤ 0∙05 was considered statistically significant.

## 3. Results

### 3.1. Differential Expression Analysis between GBM and Normal Tissues

**Figure 1:**
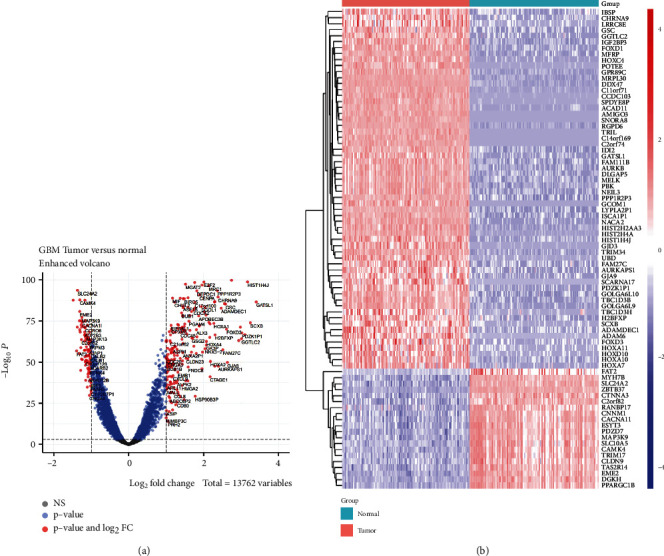
Differential expression analysis between GBM samples and normal brain tissue. (a) Volcano plot showing DEGs between GBM and normal brain tissue. (b) Heatmap showing differences in DEG expression patterns between GBM and normal brain tissue. Red, upregulated; blue, downregulated. GBM: glioblastoma multiforme; DEGs: differentially expressed genes.

We assessed the expression profiles of preprocessed GBM and normal brain tissue sample data downloaded from TCGA and GTEx. A differential expression analysis was performed using the “DEseq2” package in R, the screening threshold was FDR ≤ 0∙01, and the Benjamini and Hochberg correction significance level was FC ≥ 2 OR FC ≤ 1/2. Three hundred and fifty-seven DEGs were identified. Of these, 266 were upregulated and 91 were downregulated (Table S1). A volcano map was plotted based on the foregoing results (Figure 1(a)). To display the gene expression levels in the tumour and normal brain tissues, we extracted 60 upregulated and 20 downregulated DEGs (Table S1) and used their expression levels to plot a standardized expression profile heat map (Figure 1(b)).

### 3.2. Candidate Ferroptosis Geneset1 (cd-Ferr-Geneset1) Was Obtained Based on GO and KEGG Pathway Analyses of Ferroptosis-DE Genes

**Figure 2:**
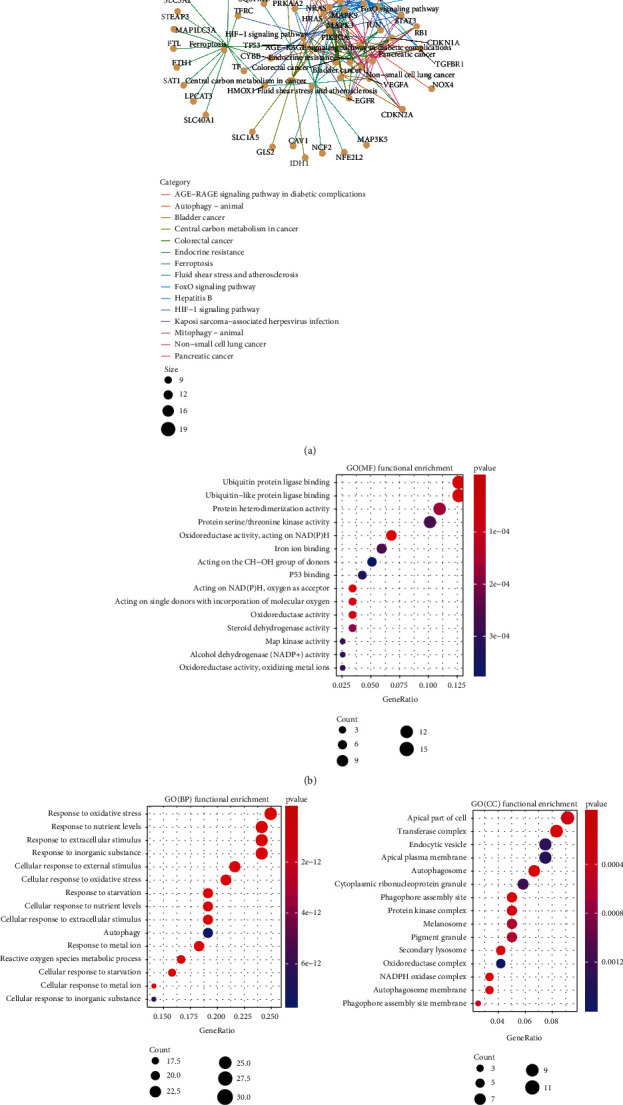
Functional enrichment analysis of ferroptosis-related DEGs in GBM samples. (a) KEGG pathway enrichment analysis network for ferroptosis-related DEGs. (b)–(d) GO enrichment analysis of MF, BP, and CC ranked by adjusted *p* value (*p*.adjust ≤ 0∙05), respectively. DEGs: differentially expressed genes; KEGG: Kyoto Encyclopedia of Genes and Genomes; GBM: glioblastoma multiforme; GO: Gene Ontology; MF: molecular function; BP: biological process; CC: cellular component.

A KEGG function enrichment analysis was performed on the DEGs identified in the GBM and normal brain tissue samples. We screened significantly enriched pathways (Figure S1). The DEGs were enriched in pathways related to cell growth and development, transcriptional regulation, and calcium signalling, such as “cell cycle,” “p53 signalling,” “Toll-like receptor signalling,” and “calcium signalling” (Figure S1). A GO enrichment analysis showed that the DEGs were not enriched in any iron-related function. Hence, 122 ferroptosis-DE genes were screened from among the DEGs for KEGG and GO enrichment analyses. According to the significance threshold, the top 15 significantly enriched pathways and GO terms were screened out for display. A KEGG functional enrichment analysis showed that the ferroptosis-DE genes were enriched in iron-related pathways, such as “ferroptosis” (*p* = 3∙16*E* − 17; Figure 2(a); Table S2). The GO enrichment analysis showed that the ferroptosis-DE genes were enriched in iron and oxygen consumption-related functions. For example, the GO functions in molecular function included “iron ion binding” (*p* = 2∙53*E* − 04; Figure 2(b); Table S3). The GO functions in biological process include “iron ion transport” (*p* = 5∙15*E* − 08), “iron ion homeostasis” (*p* = 1∙97*E* − 08), “cellular iron ion homeostasis” (*p* = 3∙97*E* − 08), “response to iron ion” (*p* = 7∙07*E* − 07), and “iron ion transmembrane transport” (*p* = 4∙70*E* − 04) (Figure 2(c); Table S4). In terms of cell components, however, no significant iron-related functions were found (Figure 2(d); Table S5). To obtain the cd-Ferr-Geneset1 (Table S6), we downloaded all pathway and GO term genes from the MSigDB (V7∙2) database. Furthermore, from the KEGG and GO term analysis of ferroptosis-DE genes, we extracted genes as they were enriched in iron and oxygen consumption-related functions.

### 3.3. Candidate Ferroptosis Geneset2 (cd-Ferr-Geneset2) Obtained Based on Cluster Analysis of Ferroptosis-DE Genes

**Figure 3:**
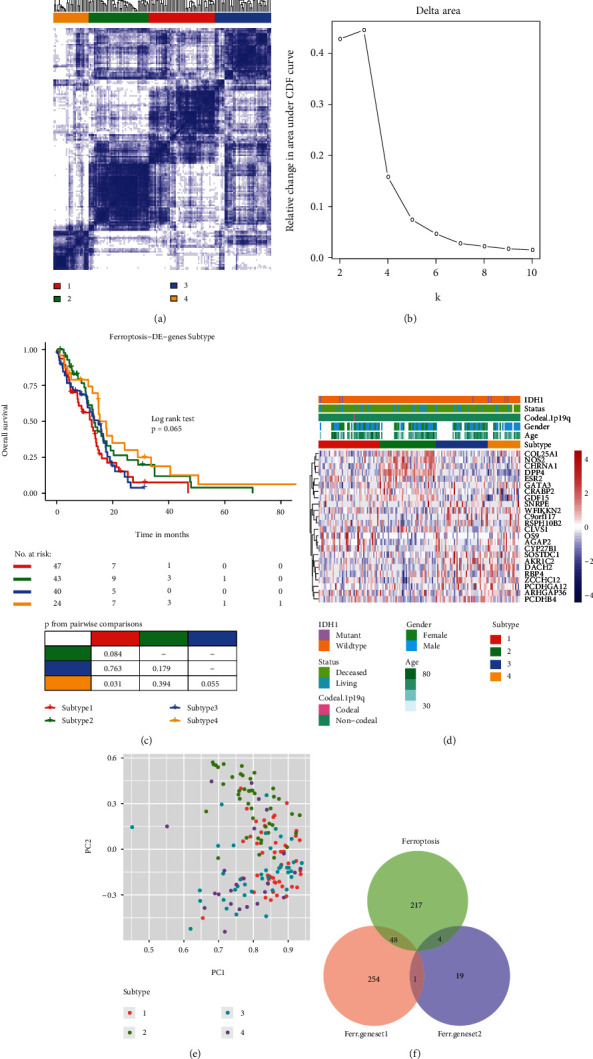
Identification of candidate ferroptosis genes in GBM samples. (a) Heatmap showing optimal consensus cluster effect (four subtypes), white (not clustered), and blue (clustered). (b) AUC of cumulative distribution function (c, d, and f). (c) Overall survival of patients among subtypes. (d) Heatmap showing DEG expression levels in four subtypes. (e) PCA showing expression levels in four subtypes. (f) Venn diagram of two categories of newly identified genes (cd-Ferr-Geneset1; cd-Ferr-Geneset2) and known ferroptosis genes. GBM: glioblastoma multiforme; AUC: area under curve; DEGs: differentially expressed genes; PCA: principal component analysis.

Based on the ferroptosis-DE gene expression levels, we used the consensus cluster analysis method on patients with GBM and explore the correlations between clustering category and patient survival time (Figures 3(a) and 3(b)). The optimal clustering effect was realised when the patients with GBM were divided into four subtypes (Figure 3(b)). A survival analysis showed that subtype 4 had relatively longer survival times than subtypes 1 and 3 (*p* = 0∙031 and *p* = 0∙055, respectively) (Figure 3(c)). We used the “DEseq2” package in R for differential gene expression analysis and compared differential gene expression among the four subtypes. In all cases, the screening threshold was FDR ≤ 0∙05, and the Benjamini and Hochberg correction significance level was FC ≥ 1∙5 OR FC ≤ 2/3. Intersection of the DEGs identified in the four subtypes identified the ferroptosis-DE genes in the GBM samples. These 24 genes were then used as cd-Ferr-Geneset2 (Table S7). A heatmap of the cd-Ferr-Geneset2 expression levels in the four ferroptosis subtypes was plotted using the “pheatmap” package in R. The genes in the cd-Ferr-Geneset2 were significantly differentially expressed among the four subtypes (Table S8-11). We used the “Psych” package in R to conduct a PCA based on the cd-Ferr-Geneset2 expression levels in the four ferroptosis subtypes. We displayed the first two principal components (PC1 and PC2) contributing to the majority of the sample characteristics. The genes in the cd-Ferr-Geneset2 were localised mainly to PC1 for all four ferroptosis subtypes. Subtype 2 had a higher degree of discrimination than the other subtypes (Figure 3(e)). By combining cd-Ferr-Geneset1, cd-Ferr-Geneset2, and known ferroptosis genes (Table S12), we found that forty-eight genes intersected between ferroptosis genes and cd-Ferr-Geneset1, whereas four genes intersected between ferroptosis genes and cd-Ferr-Geneset2. Only one gene intersected between cd-Ferr-Geneset1 and cd-Ferr-Geneset2 (Figure 3(f)).

### 3.4. Weighted Gene Coexpression Network Analysis (WGCNA) Based on Ferroptosis Geneset

**Figure 4:**
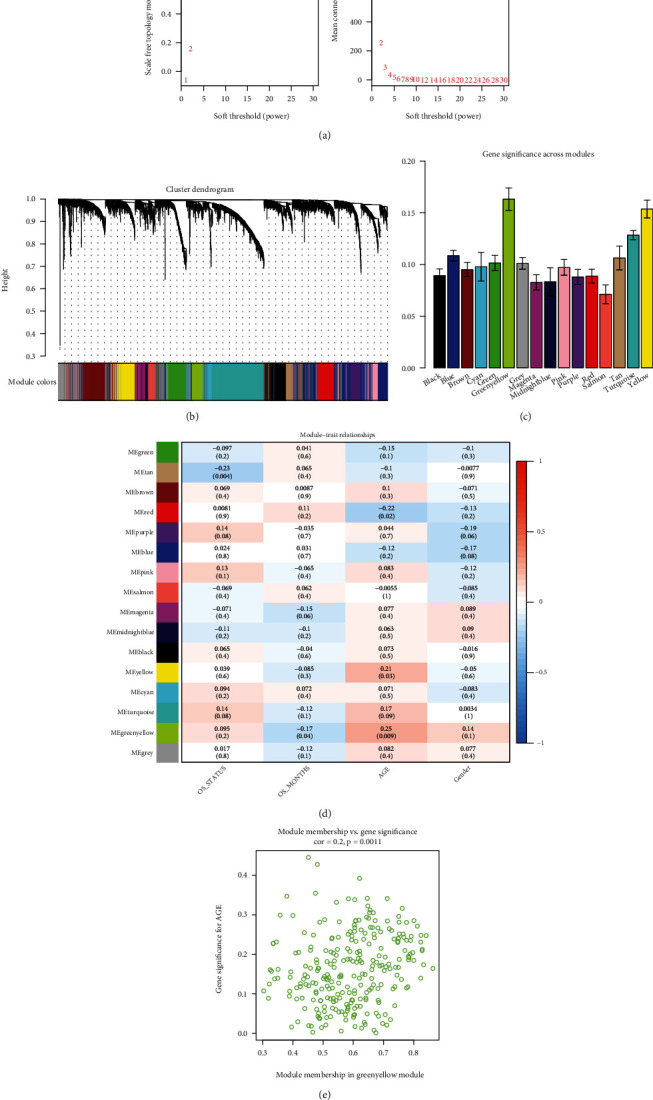
WGCNA of gene expression level in GBM. (a) Distribution diagram of soft threshold and mean connectivity. (b) Hierarchical cluster displaying various modules. Different colours represent genes in different modules; grey = unclassified genes. (c) Gene significance across modules. Average correlations among various module genes and clinical phenotypes. (d) Association analysis of module genes and clinical phenotypes; red (positive correlation), and blue (negative correlation). (e) Scattergram of correlations between green-yellow modules and the clinical age phenotypes. WGCNA: weighted gene coexpression network analysis; GBM: glioblastoma multiforme.

The union of known ferroptosis genes, cd-Ferr-Geneset1, and cd-Ferr-Geneset2 was plotted to generate a ferroptosis geneset consisting of 543 genes (Figure 3(f)). A weighted gene network was constructed by calculating Pearson's correlation coefficient between gene pairs. The soft threshold was calculated to the *n*^th^ power operation of Pearson's correlation coefficient. Based on the soft threshold distribution diagram and the mean connectivity, we obtained a power of five (Figure 4(a)). A hierarchical cluster dendrogram was plotted using the Pearson's correlation coefficients for gene pairs. Different colours and cluster tree branches represent different modules and gene modules, respectively. We divided the genes into 16 modules (Figures 4(b) and 4(c); Table 3). Based on their weighted Pearson's correlation coefficients, the genes were classified by expression pattern. Genes with similar patterns were grouped into a single module. We found that most of the modules had a significance level of approximately 0∙1. The mean significance level of the green-yellow module was the highest (0∙165) (Figure 4(c)). A correlation analysis between the modules and clinical features revealed that the green-yellow module had the strongest positive correlation with the clinical feature age (*r* = 0∙25; *p* = 0∙009; Figure 4(d)). In the present study, then, the green-yellow module was selected for the subsequent downstream analysis. When we calculated the correlations between the green-yellow module and age separately, we identified significant positive correlations between both modules (*r* = 0∙2; *p* = 0∙0011; Figure 4(e)).

### 3.5. Construction of a Multifactor Regulatory Network of Ferroptosis Key Hub Genes

**Figure 5:**
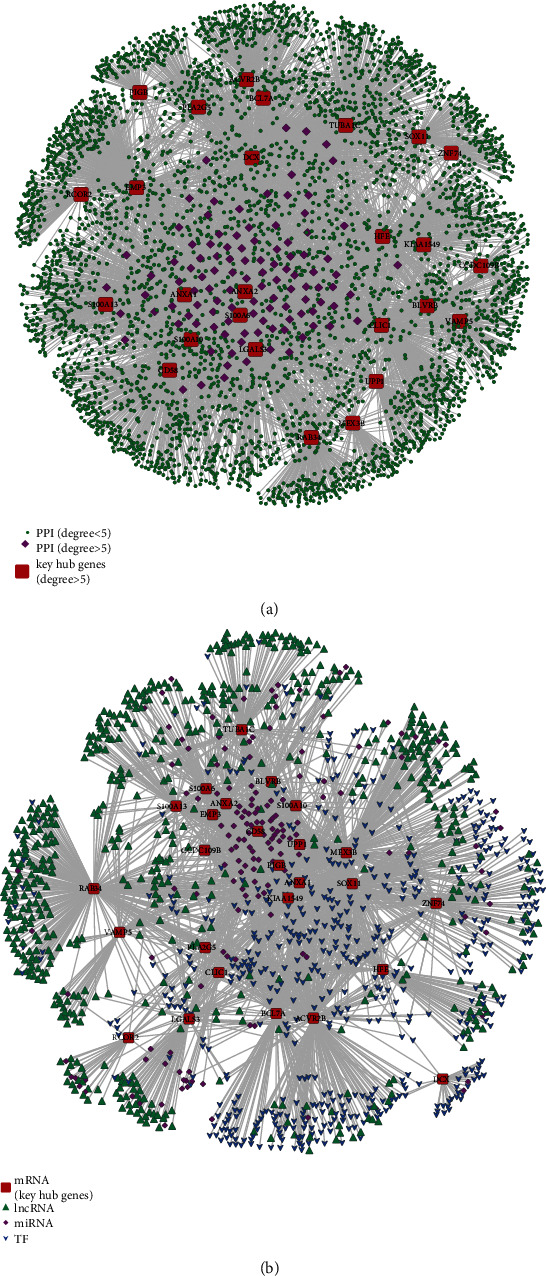
Identification of ferroptosis-related hub genes. (a) Screening of ferroptosis-related hub genes by PPI network; red (hub genes), purple (degree > five), and green (degree < five). (b) Multifactor regulatory network of ferroptosis-related hub genes in GBM; red (hub genes), green (lnRNA), purple (miRNA), and blue (TF). PPI: protein-protein interaction; TF: transcription factor.

Twenty-nine genes were screened according to the threshold of the correlation coefficient of MM and GS. Based on the degrees of the known PPI interaction network and using degree ≥ 5 as the threshold, 194 hub genes were screened (Figure 5(a)). There were 26 intersections between the hub nodes genes and the key module genes (Figure S2). These intersections were regarded as the ferroptosis key hub genes in GBM. Regulatory relationship data for the TFs and noncoding RNAs (miRNAs and lncRNAs) on the mRNAs were downloaded from known databases to identify the regulatory factors of ferroptosis key hub genes in GBM. Cytoscape was used to construct a multifactor regulatory network of the ferroptosis key hub genes (Figure 5(b); Table S13).

### 3.6. Univariate Cox Regression Analysis Screened Key Hub Genes Related to GBM Prognosis

**Figure 6:**
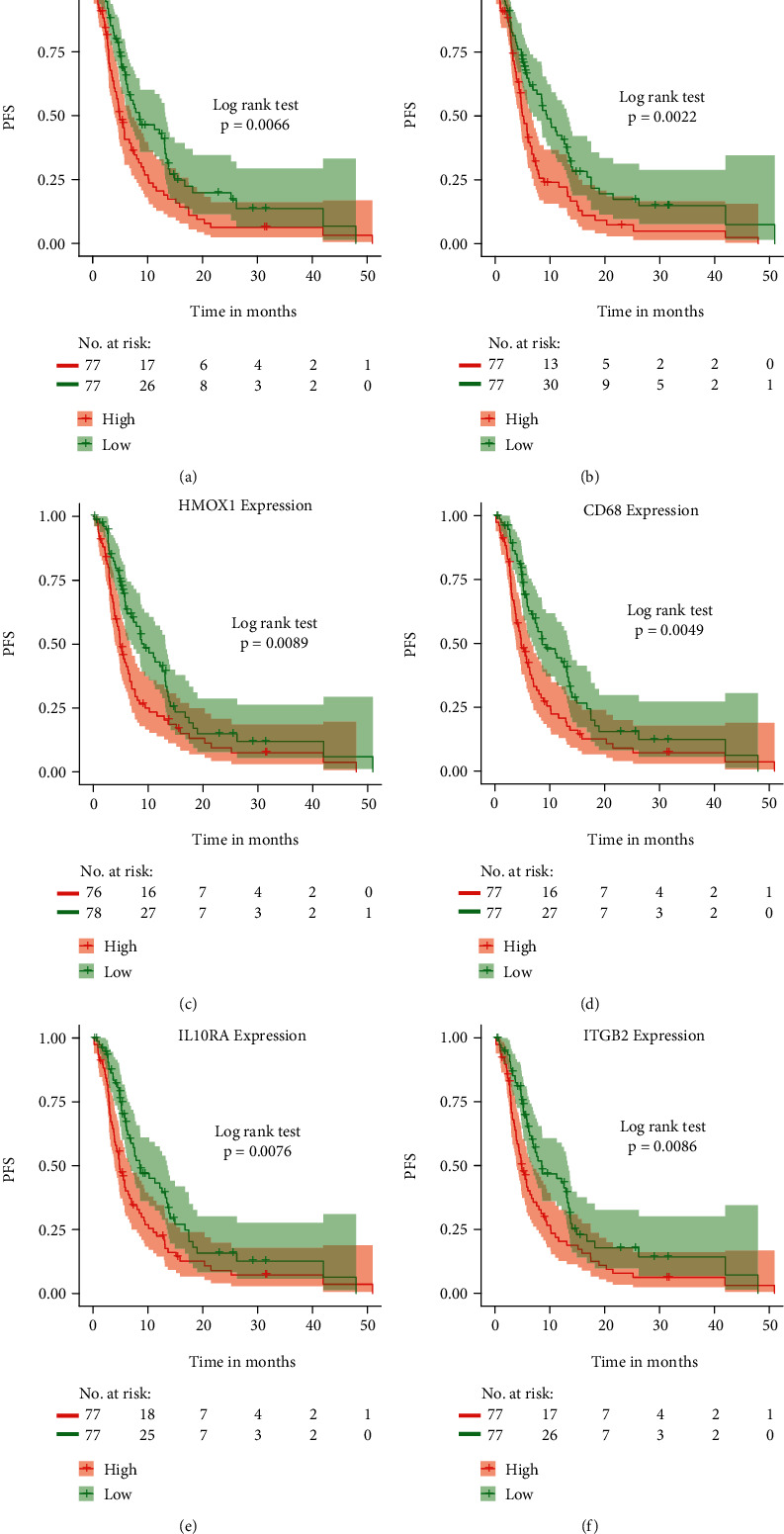
Identification of prognostic ferroptosis key hub genes. Prognostic ferroptosis key hub genes in GBM samples were screened using univariate Cox regression analysis and depicted by Kaplan-Meier survival analysis. (a)–(c) Known ferroptosis-related genes (AlOX5, CDKN1, and AHMOX1). Ferroptosis-related hub genes (CD68, IL10RA, and ITGB2) (d)–(f). GBM: glioblastoma multiforme.

**Figure 7:**
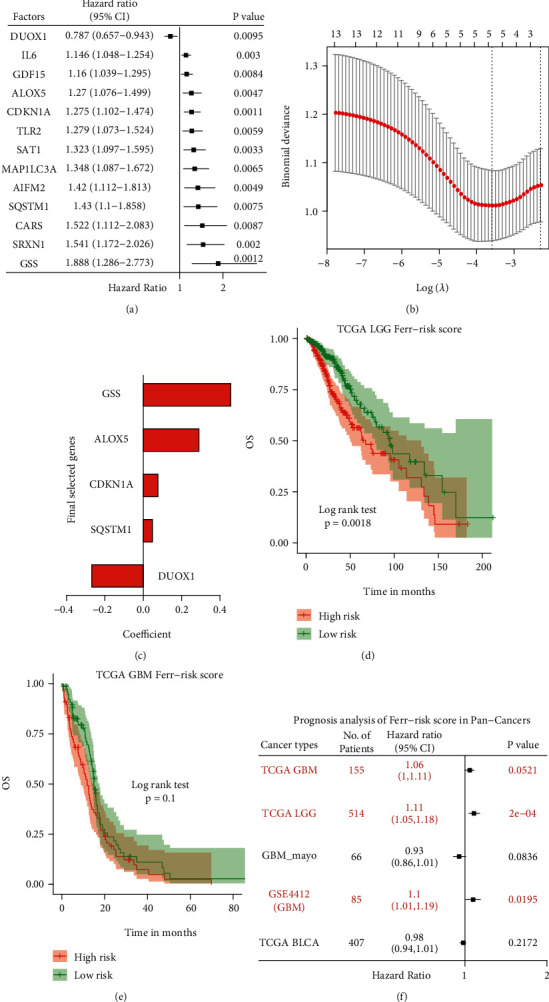
Construction of FRGPRS model for GBM. (a) Univariate Cox regression analysis. Forest plot of associations among 13 prognosis factors and GBM survival. Genes with HR > 1 are risk factors, whereas genes with HR < 1 are protective factors. (b) Identification of five-gene OS risk signature using Lasso-logistic regression analysis. (c) Lasso coefficient spectrum of five genes in GBM. (d)–(f) Prognostic analysis of FRGPRS in pan-cancers. (d) Kaplan-Meier survival analysis for TCGA LGG. (e) Kaplan-Meier survival analysis for TCGA GBM. (f) Cox regression analysis outcomes of pan-cancers. GBM: glioblastoma multiforme; OS: overall survival; Lasso: the least absolute shrinkage and selection operator.

The patients with GBM were grouped according to the median expression levels of ferroptosis key hub genes and known ferroptosis genes. The relationships between the expression levels of the prognostic key genes and patient survival time were explored using the log-rank test. The analysis demonstrated that high expression levels of certain known ferroptosis-related genes and some of ferroptosis key hub genes are associated with significantly worse patient survival time (Figure 6). Other ferroptosis-related genes are listed in Figure S3. Thirteen prognosis-related genes were screened (Figure 7(a)).

### 3.7. Construction of a Prognostic Risk Scoring Model for Ferroptosis Key Hub Genes

A Lasso-logistic regression was used to screen for other prognostic factors and remove redundant ones. The model had optimal performance when it included five prognostic factors (Figure 7(b)). Hence, they were selected for the subsequent analyses. A Cox regression analysis identified one protective factor (DUOX1) and four risk factors (CDKN1A, GSS, ALOX5, and SQSTM1) (Figure 7(c), Table S14).

### 3.8. Evaluation of the Effectiveness of the Risk Scoring Model and Pan-Cancer Analysis

**Figure 8:**
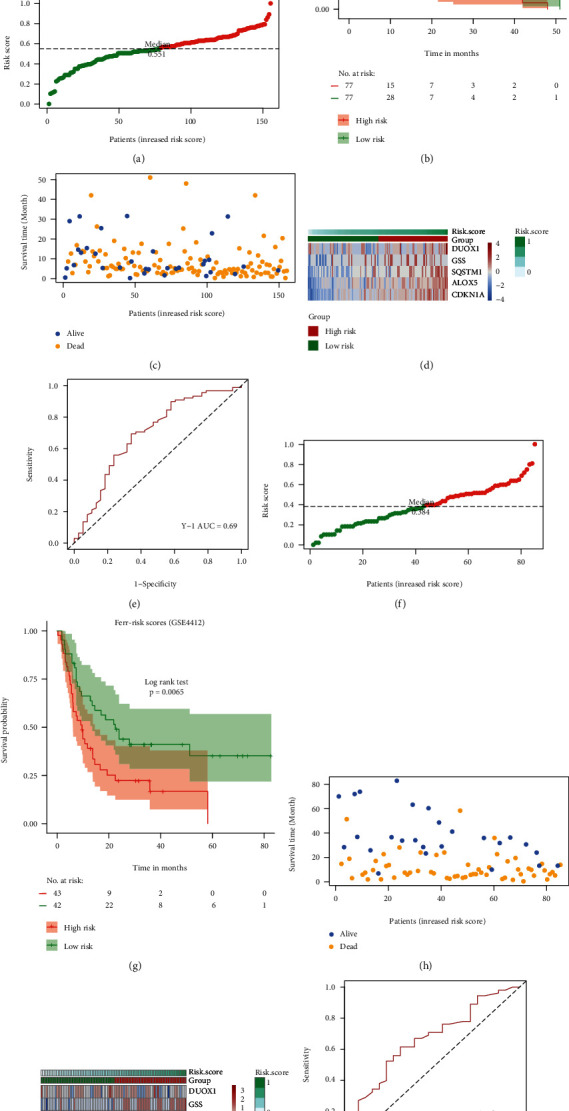
Efficiency evaluation of FRGPRS model for GBM. (a) and (e) Evaluation with TCGA GBM. (f) and (j) Evaluation with GEO validation set (GSE4412). (a) and (f) distribution diagram of two patient FRGPRS datasets. Groups higher than median are high-risk, whereas those lower than median are low-risk. (b) and (g) Kaplan-Meier survival analysis of two patient FRGPRS datasets. (c) and (h) Distribution diagram of two survival FRGPRS datasets. (d) and (i) Expression levels of various prognostic factors in FRGPRS. (e) and (j) ROC curve of two survival FRGPRS datasets. GBM: glioblastoma multiforme; RCO: receiver operator characteristic.

To evaluate the overall influence of these prognostic factors on patient survival time, a scoring model was constructed based on their expression levels and Cox regression coefficients. We calculated the sample FRGPRS as well (Materials and Methods). The model was used to evaluate the predictive efficacy of TCGA GBM data and the validation dataset (GSE4412) (Table S15). For patient survival time (PFS) in TCGA, FRGPRS was ranked from low to high, and a median score of 0∙551 was obtained (Figure 8(a)). The patients were grouped according to median score. Those in the FRGPRS group had significantly worse PFS (*p* = 5∙4*E* − 03; Figure 8(b)). When the scoring model was applied to the GEO dataset, the median FRGPRS was 0∙384 (Figure 8(f)). Patients in the high-risk group had significantly worse (OS; *p* = 6∙5*E* − 03; Figure 8(g)). The patient survival status figure was drawn by the FRGPRS and ranked from small to large. Deceased patients had greater FRGPRS than living patients, especially in the TCGA GBM dataset (Figures 8(c) and 8(h)). Heatmaps of the prognostic factors were plotted on TCGA GBM data and GEO validation dataset. Patients with high CDKN1A, GSS, ALOX5, and SQSTM1 expression levels were relatively more likely to be enriched in FRGPRS. By contrast, the expression levels of the protective factor DUOX1 were negatively correlated with patient FRGPRS (Figures 8(d) and 8(i)). These findings were consistent with the effect of the expression level of a single gene on patient survival (Figures 6 and S3). We used the ROC curve to evaluate the prediction efficacy of the model. The areas under the curve (AUC) for one-year survival time were 0∙69 (TCGA GBM dataset) and 0∙68 (GEO validation data set) (Figures 8(e) and 8(j)). To characterise the prognostic efficacy of FRGPRS in pan-cancer, we downloaded multicentre data, including GBM (TCGA data, 155 samples; GEO data, 85 samples), LGG (514 samples), and BLCA (407 samples), calculated the FRGPRS, and explored the impact of the score on patient survival time (OS). Patients with high FRGPRS in TCGA LGG showed significantly worse OS (*p* = 0∙0018; log-rank test; Figure 7(d)). A Cox regression analysis demonstrated that FRGPRS was a significant risk factor, and it affected the OS of patients with LGG (HR = 1∙11; 95% CI [1∙05, 1∙18]; *p* = 2*E* − 04; Figure 7(f)). FRGPRS also influenced the OS of patients with GEO GBM as a significant risk factor (HR = 1∙1; 95% CI [1∙01, 1∙19]; *p* = 0∙019; Figures 7(f) and 8(g)). However, FRGPRS was not correlated with patient OS for TCGA GBM or BLCA (Figures 7(e) and 7(f), and S4A). Compared with other models [1, 2, 17], our ferroptosis model had superior prognostic efficacy. The AUC of our model was 0∙69, whereas those of previous models were 0∙65 and 0∙66, respectively (Figure S4B).

### 3.9. Differential Analysis of FRGPRS in Grouping Genomic and Clinical Characteristics

**Figure 9:**
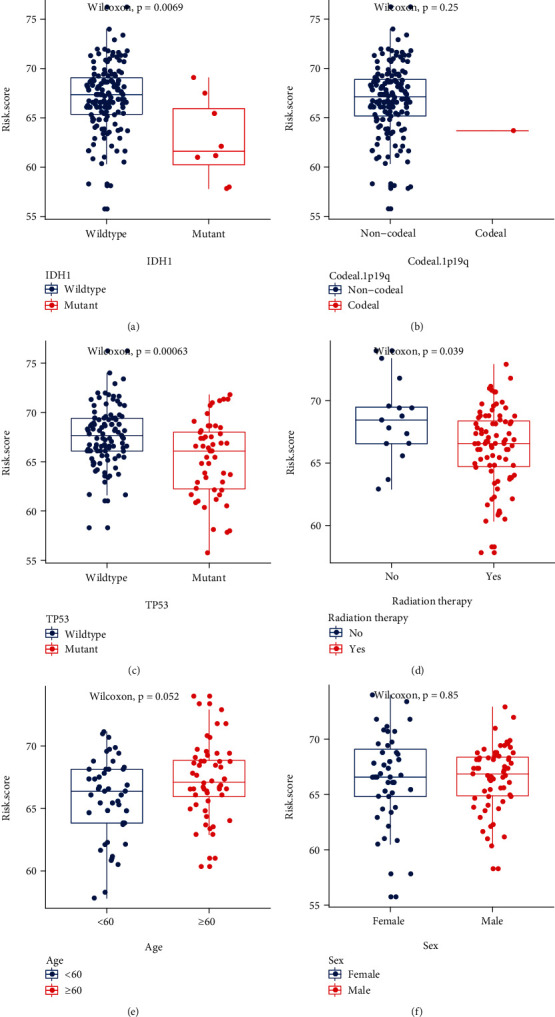
FRGPRS associated with genomic mutation and clinical characteristics. (a)–(c) Box plots depicting differences among patients with IDH1 mutation (a), 1p/19q co-del (b), and TP53 mutation (c). (d)–(f) Box plots depicting differences among patients according to radiotherapy (d), age (e), and sex (f). GBM: glioblastoma multiforme.

The genomic characteristics included IDH1 mutation, 1p/19q co-del, and TP53 mutation status. The clinical characteristics included gender, radiotherapy, and age. Patients with IDH1 mutation had significantly lower FRGPRS than those with wildtype IDH (*p* = 0∙0069; Wilcoxon rank-sum test; Figure 9(a)). Moreover, the patient with 1p/19q co-del had comparatively low FRGPRS. As there was only one patient of this type, an accurate significance level could not be calculated (Figure 9(b)). Patients with TP53 mutation had significantly lower FRGPRS (*p* = 6∙3*E* − 04; Figure 9(a)) than patients with wildtype TP53. Patients aged 60 years (*p* = 0∙039) or subjected to radiotherapy (*p* = 0∙052) had relatively reduced FRGPRS (Figures 9(d) and 9(e)). No correlation was found between gender and FRGPRS (*p* = 0∙85; Figure 9(f)). HRD scores, mutation and neoantigens loads, fractions altered, chromosome instability, and stemness indices (mRNAsi) were obtained from published studies. FRGPRS associated with genomic characteristics was reflected in Figure S5.

### 3.10. FRGPRS Tumour Immune Microenvironment Analysis

**Figure 10:**
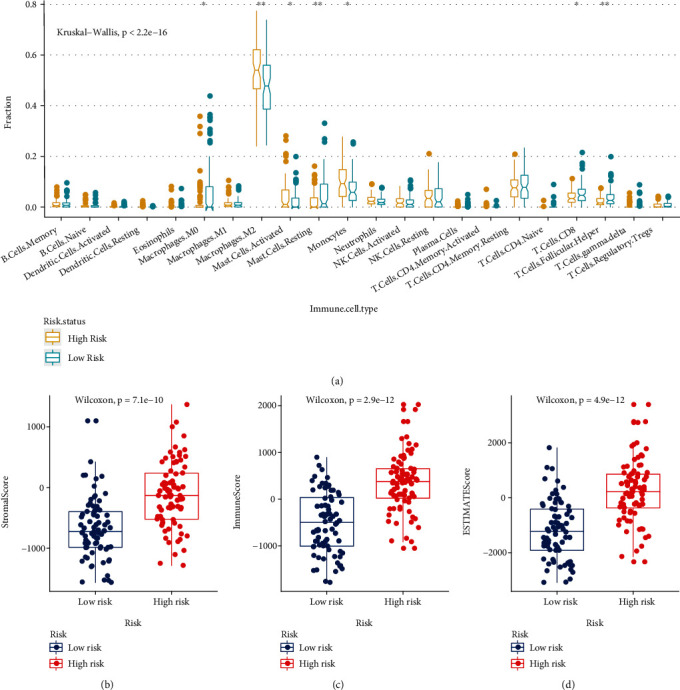
FRGPRS associated with TIME. (a) Infiltration abundance level distribution of 22 immune cell types in high- and low-risk groups (^∗^*p* < 0∙05 and ^∗∗^*p* < 0∙01). (b)–(d) Box plots diagram showing differences in (c) stromal score, (d) immune score, and (e) tumour purity between high- and low-FRGPRS groups. TIME: tumour immune microenvironment.

There were various overall immune cell infiltration scores among the high- and low-FRGPRS groups (*p* < 2∙2*E* − 16; Figure 10(a)). In addition, patients with high FRGPRS had higher infiltration scores for M0 macrophages (*p* < 0∙05), M2 macrophages (*p* < 0∙01), activated mast cells (*p* < 0∙05), and monocytes (*p* < 0∙05; Figure 10(a)). By contrast, patients with low FRGPRS had higher infiltration scores for resting mast cells (*p* < 0∙01), CD8^+^ T cells (*p* < 0∙05), and follicular helper T cells (*p* < 0∙01; Figure 10(a)). We estimated the immune, stromal, and tumour purity (ESTIMATE) scores based on the stromal: immune cell ratios. Patients with high FRGPRS had significantly higher stromal cell (*p* = 7∙1*E* − 10; Figure 10(b)), immune cell (*p* = 2∙9*E* − 12; Figure 10(c)), and tumour purity (*p* = 4∙9*E* − 12; Figure 10(d)) scores than those with low FRGPRS.

### 3.11. FRGPRS Genomic Mutation and CNA Analyses

**Figure 11:**
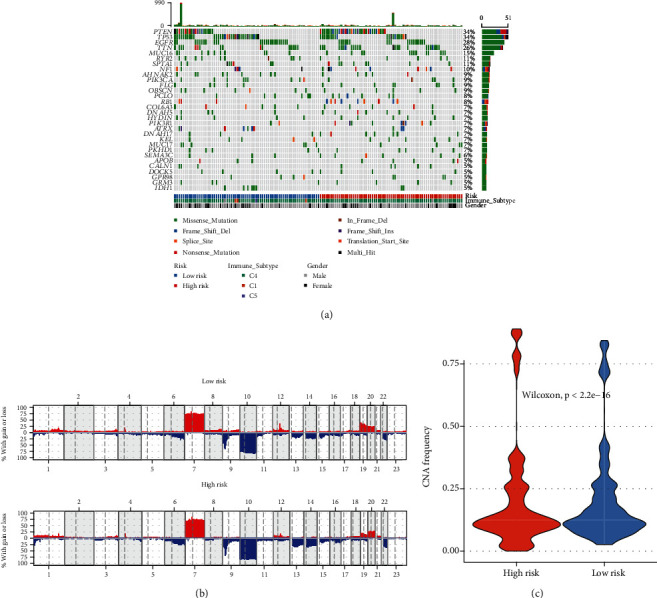
Analysis of genomic variation in FRGPRS. (a) OncoPrint diagram showing gene mutation distributions of high- and low-risk FRGPRS. (b) CNA distributions of high- and low-risk FRGPRS. (c) Violin plots depicting correlation analyses between CNA and FRGPRS. CNA: copy number alteration.

**Figure 12:**
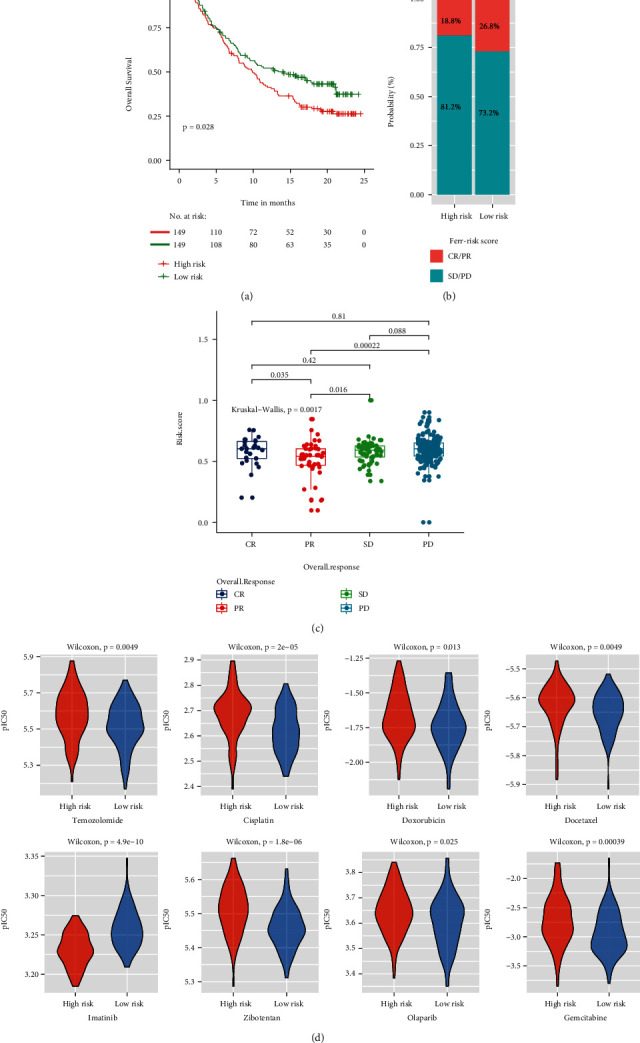
FRGPRS in prediction of immunotherapeutic benefits. (a) Kaplan–Meier curves and clinical responses to anti-PD-1 therapy in patients in IMvigor210 cohort with high- and low-FRGPRS. (b) Proportions of immunotherapy responses in high- and low-risk FRGPRS. (c) Box plots diagram depicting correlation analyses of overall response status and FRGPRS. (d) Violin plots depicting differences in estimated IC50 for temozolomide, cisplatin, doxorubicin, docetaxel, imatinib, zibotentan, olaparib, and gemcitabine between high- and low-FRGPRS groups. CR: complete response; PR: partial response; SD: stable disease; PD: progressive disease.

Silent mutations are removed while nonsynonymous mutations playing roles in protein-coding genes are retained. Thirty genes ranking from high- to low-mutation frequency were extracted and displayed using the “Maftools” package in R. PTEN (34%) had the highest mutation frequency. It included several mutations in GBM, including Nonsense_Mutation, Frame_Shift_Del, and Missense_Mutation (Figure 11(a)). PTEN is more likely to be mutated in the high-risk (41∙6%) than in the low-risk (25∙7%) patients. By contrast, for TP53 (34%), the major mutation type was Missense_Mutation, and it was more likely to occur in low-risk patients. We also explored the relationship between edge disturbance characteristic subtypes and CNA frequency. We used the “copynumber” package in R to plot the CNA frequency for each subtype. This parameter differed between high- and low-risk groups (Figures 12(b) and 12(c); *p* < 2∙2*E* − 16; Wilcoxon test).

### 3.12. Association of FRGPRS with GBM Drug Resistance and Immunotherapy

To determine whether FRGPRS could serve as an immunotherapy response marker, we extracted transcriptome and clinical data for urothelial carcinoma patients treated with the PD-L1 blocker atezolizumab. High FRGPRS was associated with poor patient outcome (*p* = 0∙028; log-rank test; Figure 12(a)). Patients in the high-risk group had comparatively lower atezolizumab response rates (CR/PR = 13∙2%), whereas those in the low-risk group had relatively higher atezolizumab response rates (CR/PR = 16∙1%; Figure 12(b)). The atezolizumab response (CR/PR) and nonresponse (SD/PD) groups differed in terms of their FRGPRS (*p* = 0∙0017; Kruskal–Wallis test; Figure 12(c)). Patients with SD and PD had higher FRGPRS than patients with CR and PR. Ridge regression was used to predict drug sensitivity in patients based on cell line expression and drug response data downloaded from GDSC. We examined the correlations between the high- and low-risk groups and their drug response patterns. Low-risk GBM patients were comparatively more sensitive to temozolomide (*p* = 4∙9*E* − 03; Figure 12(d)), cisplatin (*p* = 2*E* − 05), the PARP inhibitor olaparib (*p* = 0∙025), and anthracycline/taxanes. Low-risk GBM patients had significantly lower levels IC50 for doxorubicin (*p* = 0∙013) and docetaxel (*p* = 0∙0049). By contrast, high-risk GBM patients showed lower IC50 for imatinib (*p* = 4∙9*E* − 10) (Figure 12(d)). In addition, the response patterns of patients with low FRGPRS that were sensitive to zibotentan (*p* = 1∙8*E* − 06) and gemcitabine (*p* = 3∙9*E* − 04) were consistent with those for temozolomide (Figure 12(d)).

### 3.13. Independent Prognostic Factor Analysis of FRGPRS

**Figure 13:**
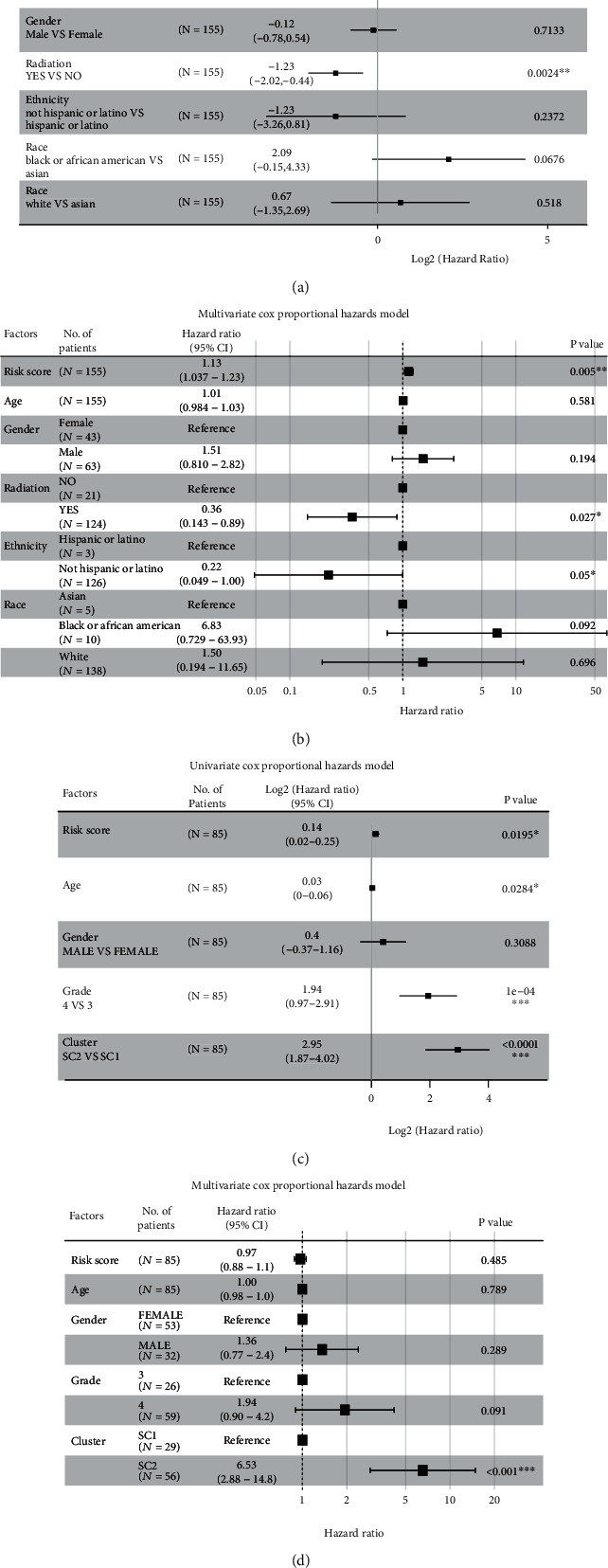
Analysis of independent prognostic factors of FRGPRS. (a) and (b) Forest plots showing univariate Cox (a) and multifactor Cox (b) regression analyses of training dataset (TCGA GBM; progression-free survival). (c) and (d) Forest plots showing univariate Cox (c) and multifactor Cox (d) regression analyses of validation dataset GSE4412 GBM; overall survival.

FRGPRS was applied to TCGA GBM and GEO samples in univariate Cox analyses. FRGPRS (log^2^HR = 0∙15; 95% CI [0∙07, 0∙23]; *p* = 0∙0024; Figure 13(a)) and age (log^2^HR = −0∙03; 95% CI [0, -0∙05]; *p* = 0∙019) were significant risk factors influencing GBM patient survival. Moreover, the radiotherapy status of patients with GBM positively affected patient survival (log^2^HR = −1∙23; 95% CI [-2∙02, -0∙44]; *p* = 0∙0024; Figure 13(a)). After adjusting for age, sex, race, and radiotherapy status, a multivariate Cox regression analysis showed that FRGPRS was an independent risk prognostic factor influencing patient survival (HR = 1∙13; 95% CI [1∙037, 1∙23]; *p* = 0∙005; Figure 13(b)). Radiotherapy status was also an independent protective prognostic factor (HR = 0∙36; 95% CI [0∙143, 0∙89]; *p* = 0∙027). FRGPRS was applied to the validation set (GEO) and indicated to be a significant risk factor (log^2^HR = 0∙14; 95% CI [0∙02, 0∙25]; *p* = 0∙0195; Figure 13(c)). Age, tumour grade, and GBM subtype were significant risk factors affecting patient survival (age: log^2^HR = 0∙03; 95% CI [0, 0∙06]; *p* = 0∙028; tumour grade: log^2^HR = 1∙94; 95% CI [0∙97, 2∙91]; *p* = 1*E* − 04; GBM subtype: log^2^HR = 2∙95; 95% CI [1∙87, 4∙02]; *p* < 0∙0001; Figure 13(c)). After correcting for age, tumour grade, and GBM subtype, however, the multivariate Cox regression analysis showed that the influence of FRGPRS was not significant (*p* = 0∙48; Figure 13(d)).

### 3.14. FRGPRS Nomogram

**Figure 14:**
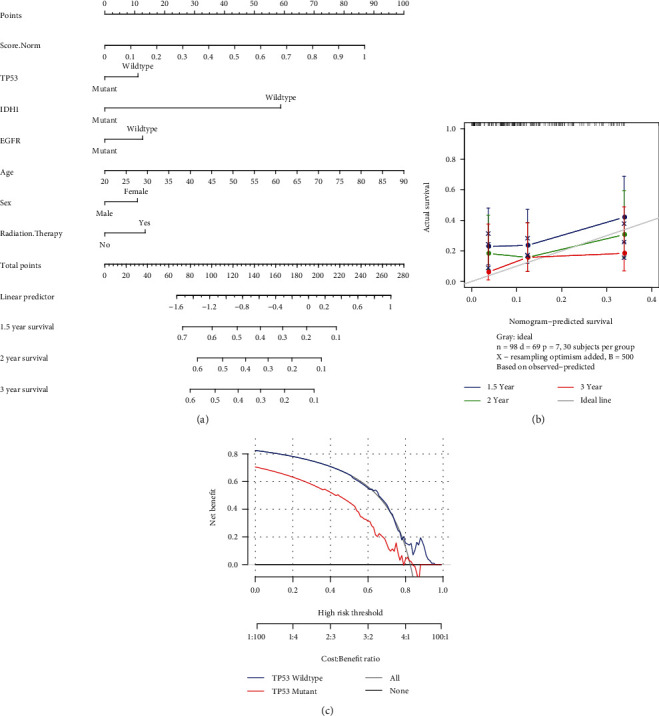
(a) Nomogram of FRGPRS in GBM patients. (b) Calibration maps were used to predict 1.5-year, 2-year, and 3-year survival. (c) Decision curve analysis of nomogram for *TP53* mutation risk.

**Figure 15:**
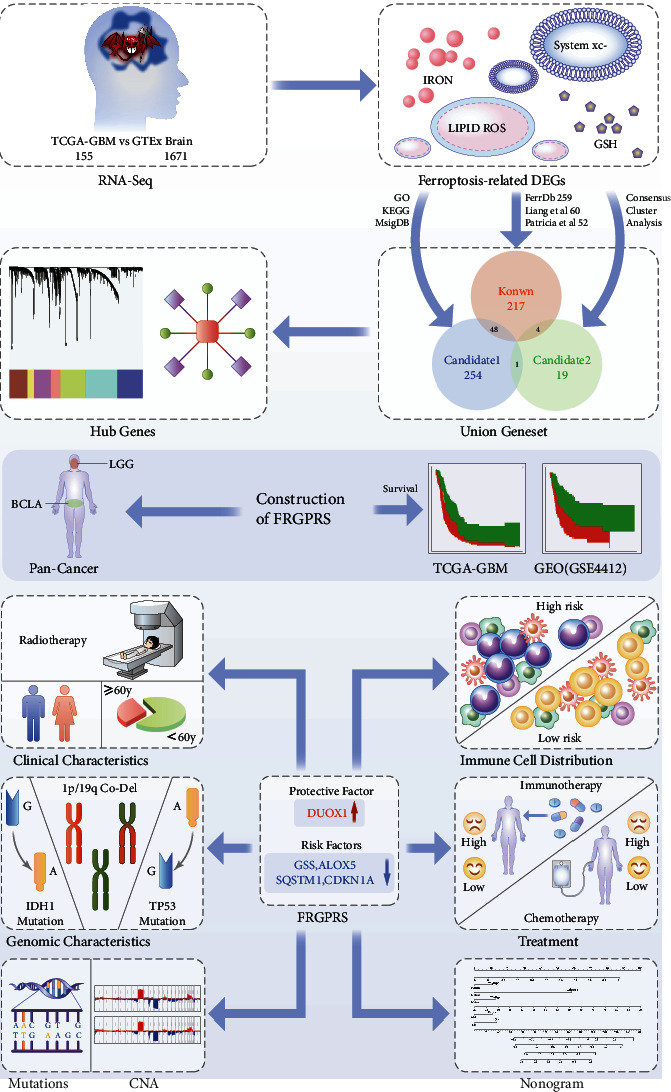
Graphical abstract for comprehensive characterization of FRGPRS groups in GBM. CNA: copy number alteration.

Using the FRGPRS, TP53/IDH1/EGFR mutation status, age, gender, and radiotherapy status data, a nomogram for the clinical analysis of patients with GBM was generated with the “RMS” package in R. In this study, the *c*-index calculated after 500 iterations is 0.683. For instance, if one patient had FRGPRS = 0∙6 (points = 52), age = 50 (points = 43), TP53 mutation status = wildtype (points = 10), IDH1 mutation status = wildtype (points = 58), EGFR mutation status = wildtype (points = 12), and sex = female (points = 10), then points = 185. The 1.5-year, 2-year, and three-year survival rates would be 22%, 17%, and 15%, respectively (Figure 14). A calibration curve was plotted for the nomogram to compare between the actual and predicted risk at years one and a half, two, and three. The curves for years two and three approximated the ideal line (Figure 14(b)). A decision curve analysis was conducted based on the TP53 mutation state. The patient with wildtype TP53 had comparatively superior net benefit (Figure 14(c)). If we choose to diagnose and treat GBM with a predicted probability of 50%, then 5/100 patients with TP53 mutation will benefit from it without reducing the benefit to other patients. However, 7/100 patients with wildtype TP53 will benefit from it without decreasing the benefit to other patients (Figure 14(c)). Graphical abstract is for comprehensive characterization of FRGPRS groups in GBM (Figure 15).

## 4. Discussion

In the present study, we comprehensively evaluated ferroptosis-related genes and their correlations with patient prognosis, drug resistance, immune infiltration, immunotherapy response, and gene mutation in GBM. As prognosis and survival are poor for GBM, concerted efforts have been made to improve quality of life and clinical benefit for GBM patients. To these ends, we constructed a prognostic risk model of five ferroptosis-related genes in patients with GBM. It was based on the PFS in TCGA chart and was validated with a GEO dataset. The calibration curve indicated that the prediction effect for years two and three was relatively good (Figure 14(b)). High-FRGPRS levels indicated poor prognosis and insensitivity to first-line chemotherapy in GBM patients. Immunotherapy results revealed that the anti-PD-L1 response to urothelial cancer was comparatively more sensitive in low-risk patients. FRGPRS was significantly correlated with CAN and reflected the predictive power of gene mutation and immune infiltration, and these were closely related to clinical features, outcome, recurrence, and immune function.

There is growing evidence to suggest that ferroptosis is indispensable in eradicating cancer cells and that ferroptosis sensitivity differs among cancer types [35]. FRGPRS comprises five genes, namely, one protective factor (dual oxidase 1; DUOX1) and four risk factors (CDKN1A, GSS, ALOX5, and SQSTM1). DUOX1 is normally expressed in epithelial cells and plays an important role in the immune response [36]. DUOX1 silencing frequently occurs in epithelial-derived cancers and correlates with positive prognosis in certain tumours. However, DUOX1 expression levels in GBM are unknown [37–39]. Conditional DUOX1 overexpression could serve to evaluate the correlation between DUOX1 silencing and cancer progression or response to therapy [40]. Recent studies have revealed that DUOX1 suppression in cancer is driven mainly by hypermethylation of its promoter. Hence, DNA methyltransferase inhibition may be a promising approach toward recovering DUOX1 expression [37–39]. Defective cell cycle control is a common cause of tumorigenesis. CDKN1A is a prognostic marker for ferroptosis-related GBM [25]. CDKN1A is transcriptionally controlled by p53-dependent and p53-independent pathways and may regulate cell migration, DNA repair, and DNA reprogramming during induced pluripotent stem cell generation [40]. CDKN1A can act as a tumour suppressor or oncogene, depending on the cellular context [41]. The GPX4-GSS/GSR-GGT axis is a crucial target of ammonium ferric citrate-induced ferroptosis. Interactions between the rapamycin kinase and GPX4 targets may regulate autophagy-dependent ferroptosis in cancer cells. GPX4 downregulation enhances sensitivity to chemotherapy by promoting ferroptotic cell death [42, 43]. Lipid peroxidation is positively regulated by ALOX5 and contributes to ferroptotic cell death. Here, differential ALOX5 expression was observed between the high- and low-FRGPRS groups. This discovery was consistent with a previous report [24]. Nrf2 and p62/SQSTM1 jointly contribute to mesenchymal transition and tumour infiltration in GBM [24]. The present study showed that high SQSTM1 expression indicated poor prognosis in GBM.

Significantly higher immune, stromal, and ESTIMATE scores were observed in the high-FRGPRS group than in the low-FRGPRS group. Patients in the former group had comparatively higher immune activity, greater proportions of tumour tissue, and favourable cytolytic immune response. Prior research emphasised the importance of tumour immune classification and the evaluation of local immunological biomarkers to make decisions regarding patient prognosis and prediction of treatment efficacy [44–46]. Ferroptosis may participate in cancer immune evasion. There has been growing interest in clarifying the mechanisms regulating cancer cell sensitivity to ferroptosis [47]. PTGS2 upregulation and PGE2 release induce ferroptosis which may, in turn, modulate antitumor immunosuppression [48, 49]. Further research is required to elucidate the immunomodulatory roles of ferroptosis in antitumor immunity [48, 49]. Elevated CD8+ and follicular helper T cell counts and infiltration in the low-FRGPRS group were indicative of antitumor efficacy. Immunotherapy promotes effector T cell function mainly by inducing cell death through the perforin-granzyme and Fas–Fas ligand pathways [50–52]. There is emerging evidence that ferroptosis is associated with various pathological scenarios. However, it is unclear whether, or how, ferroptosis is implicated in T cell immunity and cancer immunotherapy. CD8+ T cells secrete interferon gamma, regulate SLC3A2 and SLC7A11 expression, and promote cancer cell lipid peroxidation and ferroptosis [53]. Evidently, T cell-induced cancer ferroptosis is an antitumor mechanism that may serve as a novel approach toward GBM immunotherapy.

We assessed the differences in gene mutation between the low- and high-FRGPRS groups to clarify the mechanisms of ferroptosis. Patients in the high-FRGPRS group showed significantly lower copy number variation frequencies than those in the low-FRGPRS group. Missense mutation and 1p19q codeletion furnish prognostically relevant information along with histological classification [54]. IDH1 mutation status was considered the basis for glioma diagnosis according to the 2016 WHO classification of CNS tumours. Gliomas with IDH1 mutations have relatively better outcomes and superior responses to therapy than those with the wildtype IDH1 gene. Nevertheless, the underlying mechanism has not been clarified [55]. A recent study revealed the roles of mutant IDH1 and 2-hydroxyglutarate in ferroptosis. The former reduces the GPX4 protein levels, thereby promoting the accumulating of lipid ROS and by extension ferroptosis [56]. Another study demonstrated that a TP53 gene variant plays a vital role in the functional interactions among thiol-based redox signalling, metabolism, and ferroptosis [57]. The low- and high-FRGPRS groups markedly differed in terms of their types of IDH1 and TP53 mutations. Overall, the wildtype forms were more abundant in the high-FRGPRS group. Hence, high FRGPRS is associated with a relatively greater risk of wildtype mutation and, therefore, worse prognosis. This finding was consistent with our survival results.

The present study revealed a significant association between FRGPRS and immunotherapy response in urothelial carcinoma patients treated with atezolizumab (anti-PD-L1). The high-risk groups presented with worse survival after atezolizumab treatment. In general, PD-L1 (+) tumours respond better to anti-PD-1/PD-L1 therapy than PD-L1 (-) tumours [58, 59]. However, certain studies failed to show any significant correlation in this case possibly because of a lack of consistency in the measurements and variability of the threshold used to define PD-L1 positivity [60]. Therefore, further investigations are needed to establish the correlation between PD-L1 and GBM.

Drug resistance is a major hindrance in GBM therapy. Our research showed that low-FRGPRS patients were relatively more sensitive to temozolomide, cisplatin, and olaparib than high-FRGPRS patients. Liu et al. reported that TMZ-resistant glioma cells are more likely to undergo ferroptosis than normal glioma cells [24]. This finding was consistent with our research. The pathways regulating ferroptosis and inducing GBM TMZ resistance are complex, multifactorial processes that remain to be elucidated [18, 61–63]. A recent study demonstrated that ferroptosis plays a vital role in cancer cell chemoresistance, and glioma-ferroptosis resistance is a putative TMZ resistance mechanism [64]. Iron is an important element in drug-resistant cancer cells. Iron-dependent ROS accumulation triggers ferroptosis [65]. Targeted ferroptosis-related pathways are promising strategies for reversing TMZ resistance.

The present study had certain limitations. As the survival time of GBM patient is short, we constructed a model based on PFS rather than OS. The independent prognostic risk factor FRGPRS was not significantly correlated with patient survival according to a multivariate Cox regression, possibly because of sample size and other factors. Nevertheless, the training and validation sets showed that high FRGPRS significantly shortened patient survival and demonstrated the reliability of the model. The present study exclusively analysed GBM samples. Thus, it is unclear whether the FRGPRS model could be applied to any glioma sample from any genetic background. Moreover, the five-gene-based ferroptosis-related signature should be validated using larger samples. Future experiments should explore the potential mechanisms of the five genes in GBM ferroptosis and attempt to establish their correlations with immunotherapy and drug resistance.

## 5. Conclusions

In conclusion, the FRGPRS model is a powerful tool for predicting the survival and guiding the treatment of GBM. It might help distinguish immune and molecular characteristics, predict patient outcome, and stratify GBM patients benefiting from chemotherapy and immunotherapy. However, further research is required to identify and confirm the prognostic value of FRGPRS.

## Figures and Tables

**Table 1:** Basic information of the datasets included in this study.

Dataset	Data type	*N*
TCGA GBM	Expression	155
	Mutation	388
	Copy number	575
	Clinical data	585
GTEx brain	Expression	1671
GEO (GSE4412)	Expression, survival	85
IMvigor210CoreBiologies	Expression, survival, drug response	298
GDSC	Drug response, genomic markers of sensitivity	155

**Table 2:** Summary of clinical information in TCGA-GBM dataset.

Total patient (*n*)	155
Age at diagnosis (median, range)	60 (21-89)
Gender	
Male	63
Female	43
Unknown	49
Vital status	
Alive	32
Dead	122
Unknown	1
Radiotherapy	
Yes	124
No	21
Unknown	10
Ethnicity	
Hispanic or Latino	3
Not Hispanic or Latino	126
Unknown	26
Race	
Black or African American	10
Asian	5
White	138
Unknown	2

**Table 3:** Gene number of each module in WGCNA.

Module type	Number
Black	361
Blue	864
Brown	433
Cyan	99
Green	378
Green-yellow	265
Grey	699
Magenta	276
Midnight blue	85
Pink	338
Purple	273
Red	362
Yellow	402
Turquoise	1,201
Tan	183
Salmon	131

## Data Availability

All data are available on public repositories, which are listed in Table 1 and main context.

## Supplementary Materials

Supplementary 1 Figure S1: Kyoto Encyclopedia of Genes and Genomes (KEGG) pathway analysis of differentially expressed genes (DEGs) (*p* < 0.05). Figure S2: Venn diagram of ferroptosis key hub genes. Figure S3: prognostic ferroptosis key hub genes in GBM samples were screened using univariate Cox regression analysis and depicted by K-M curve. (a) DUOX1 (*p* = 0.026), (b) SAT1 (*p* = 0.0042), MUC1 (*p* = 0.0027), (d) RB1 (*p* = 0.0016), (e) HSPA5 (*p* = 0.0095), and (f) HSPB1 (*p* = 0.0047). Figure S4: (a) K-M curve of two patient FRGPRS in The Cancer Genome Atlas (TCGA) Bladder Urothelial Carcinoma (BLCA), (b) ROC curve analysis of FRGPRS model and known models. Figure S5: FRGPRS associated with genomic characteristics. (a) HRD score. (b) TMB. (c) Neoantigens. (d) Fractions altered. (e)–(g) Chromosome instability. (h) Stemness index (mRNAsi). HRD: homologous recombination deficiency; TMB: tumour mutational burden.

Supplementary 2 Table S1: DEGs between GBM and normal brain tissue. Table S2: KEGG pathways enriched in ferroptosis-related genes. Table S3: GO enrichment analysis of molecular function (MF). Table S4: GO enrichment analysis of biological process (BP). Table S5: GO enrichment analysis of cellular component (CC). Table S6: cd-Ferr-Geneset1. Table S7: cd-Ferr-geneset2. Table S8: DEG.Subtype1. Table S9: DEG.Subtype2. Table S10: DEG.Subtype3. Table S11: DEG.Subtype4. Table S12: known ferroptosis genes. Table S13: a multifactor regulatory network of the ferroptosis key hub genes. Table S14: Lasso-logistic regression analysis of prognosis factors. Table S15: FRGPRS model applied for TCGA GBM and GSE4412 GBM dataset.
