# Supplementary material for: A Ferroptosis-Related Prognostic Risk Score Model to Predict Clinical Significance and Immunogenic Characteristics in Glioblastoma Multiforme
Source: Oxid Med Cell Longev. 2021 Nov 9;2021:9107857. doi: 10.1155/2021/9107857 (PMC8596022; doi:10.1155/2021/9107857)
Supplement: Supplementary 1 — Figure S1: Kyoto Encyclopedia of Genes and Genomes (KEGG) pathway analysis of differentially expressed genes (DEGs) (p < 0.05). Figure S2: Venn diagram of ferroptosis key hub genes. Figure S3: prognostic ferroptosis key hub genes in GBM samples were screened using univariate Cox regression analysis and depicted by K-M curve. (a) DUOX1 (p = 0.026), (b) SAT1 (p = 0.0042), MUC1 (p = 0.0027), (d) RB1 (p = 0.0016), (e) HSPA5 (p = 0.0095), and (f) HSPB1 (p = 0.0047). Figure S4: (a) K-M curve of two patient FRGPRS in The Cancer Genome Atlas (TCGA) Bladder Urothelial Carcinoma (BLCA), (b) ROC curve analysis of FRGPRS model and known models. Figure S5: FRGPRS associated with genomic characteristics. (a) HRD score. (b) TMB. (c) Neoantigens. (d) Fractions altered. (e)–(g) Chromosome instability. (h) Stemness index (mRNAsi). HRD: homologous recombination deficiency; TMB: tumour mutational burden. [file 9107857.f1.docx]

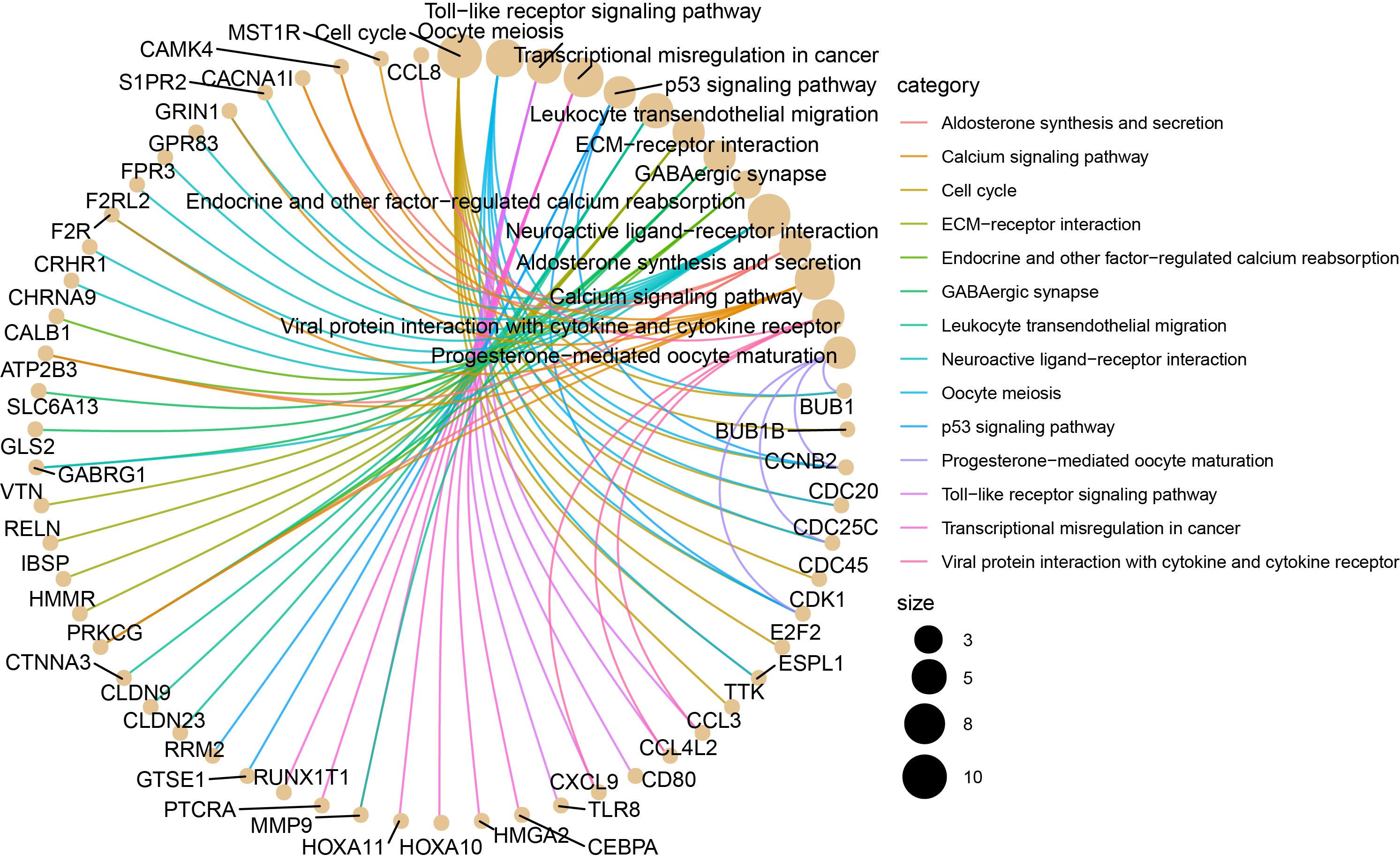


Figure S1. Kyoto Encyclopedia of Genes and Genomes (KEGG) pathway analysis of differentially expressed genes (DEGs)(p < 0.05).


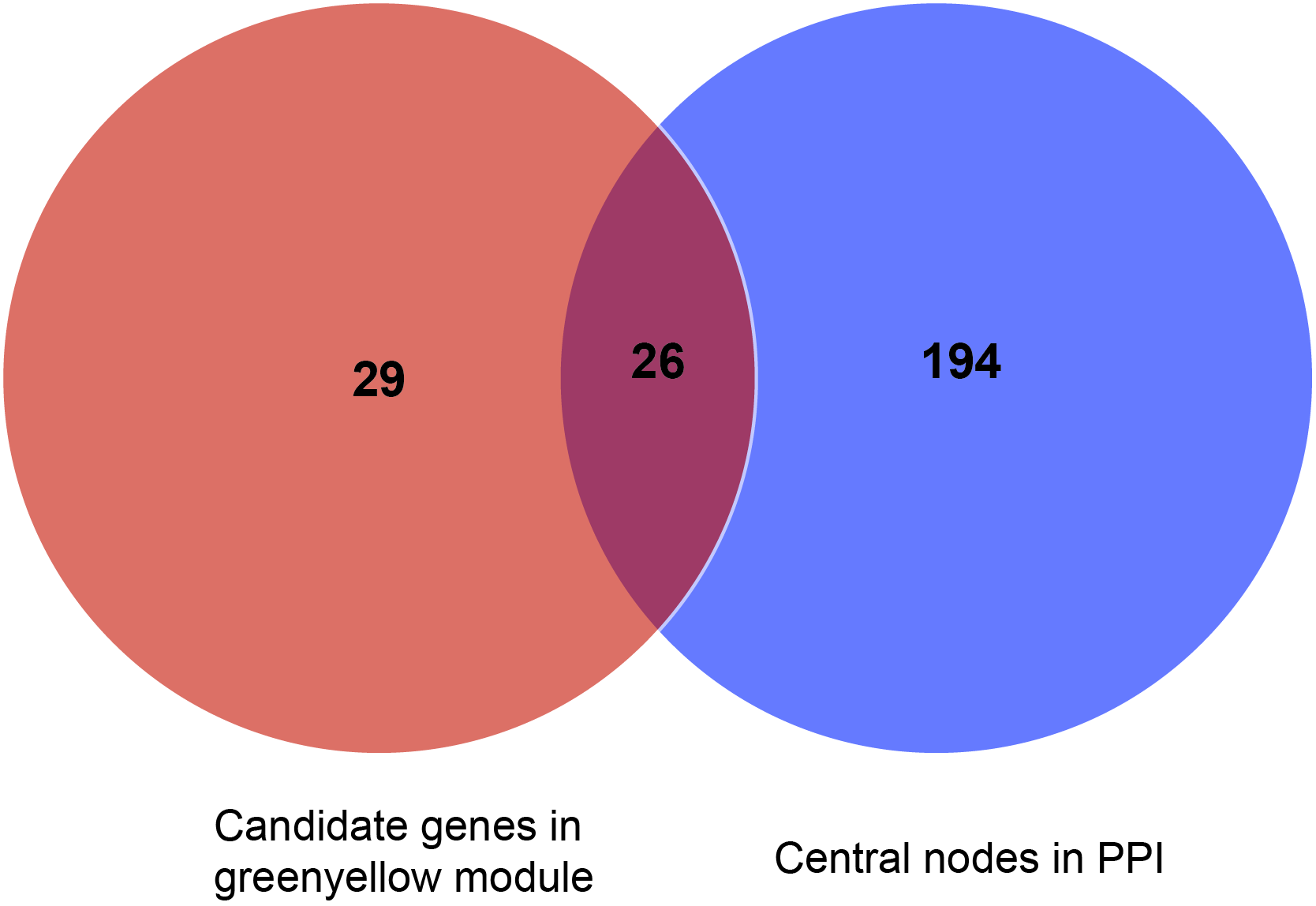


Figure S2. Venn diagram of ferroptosis key hub genes.


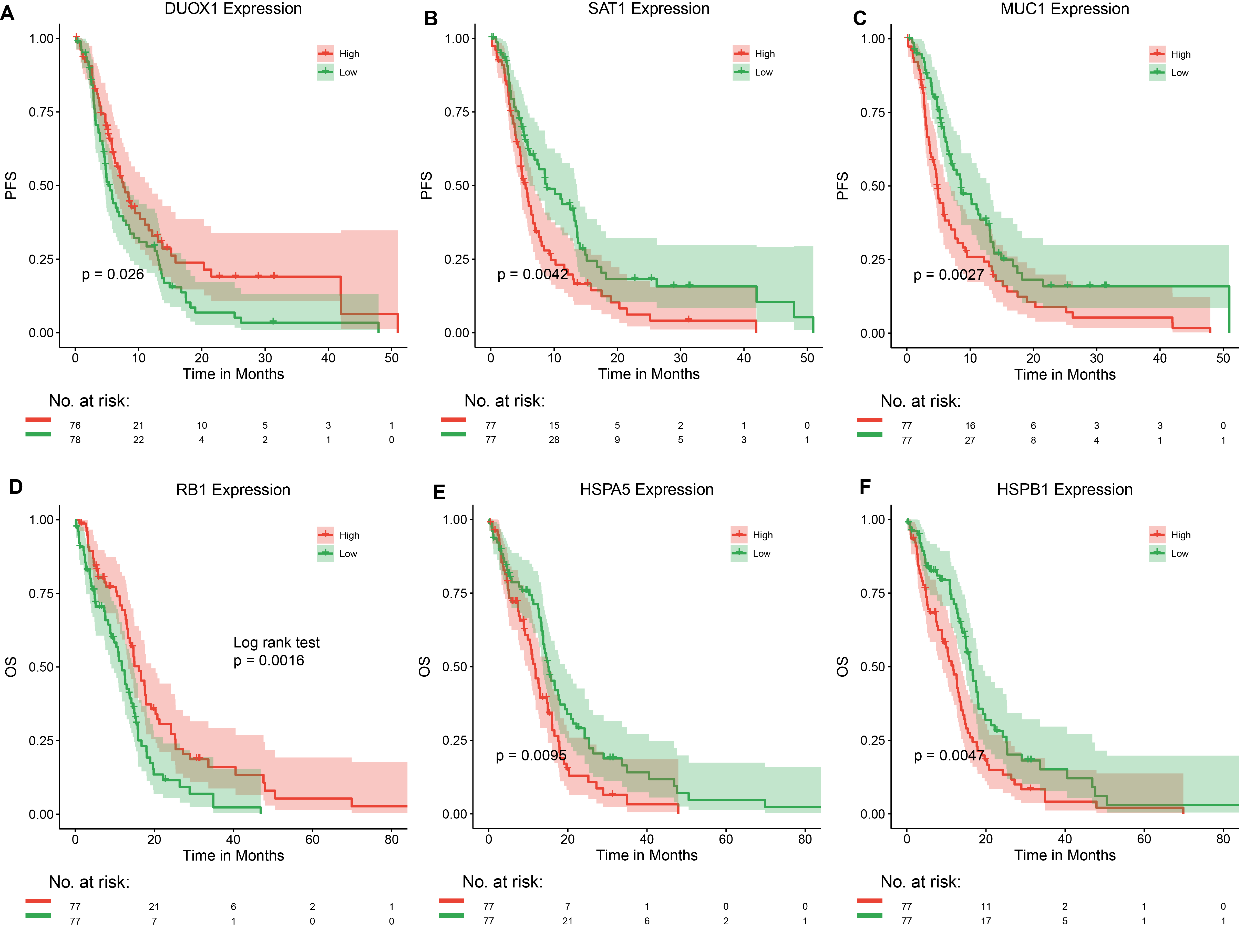


Figure S3. Prognostic ferroptosis key hub genes in GBM samples were screened using univariate Cox regression analysis and depicted by K-M curve. (A) DUOX1 (p = 0.026), (B) SAT1(p = 0.0042),

1. MUC1 (p = 0.0027), (D) RB1 (p = 0.0016), (E) HSPA5 (p = 0.0095), (F) HSPB1 (p = 0.0047).


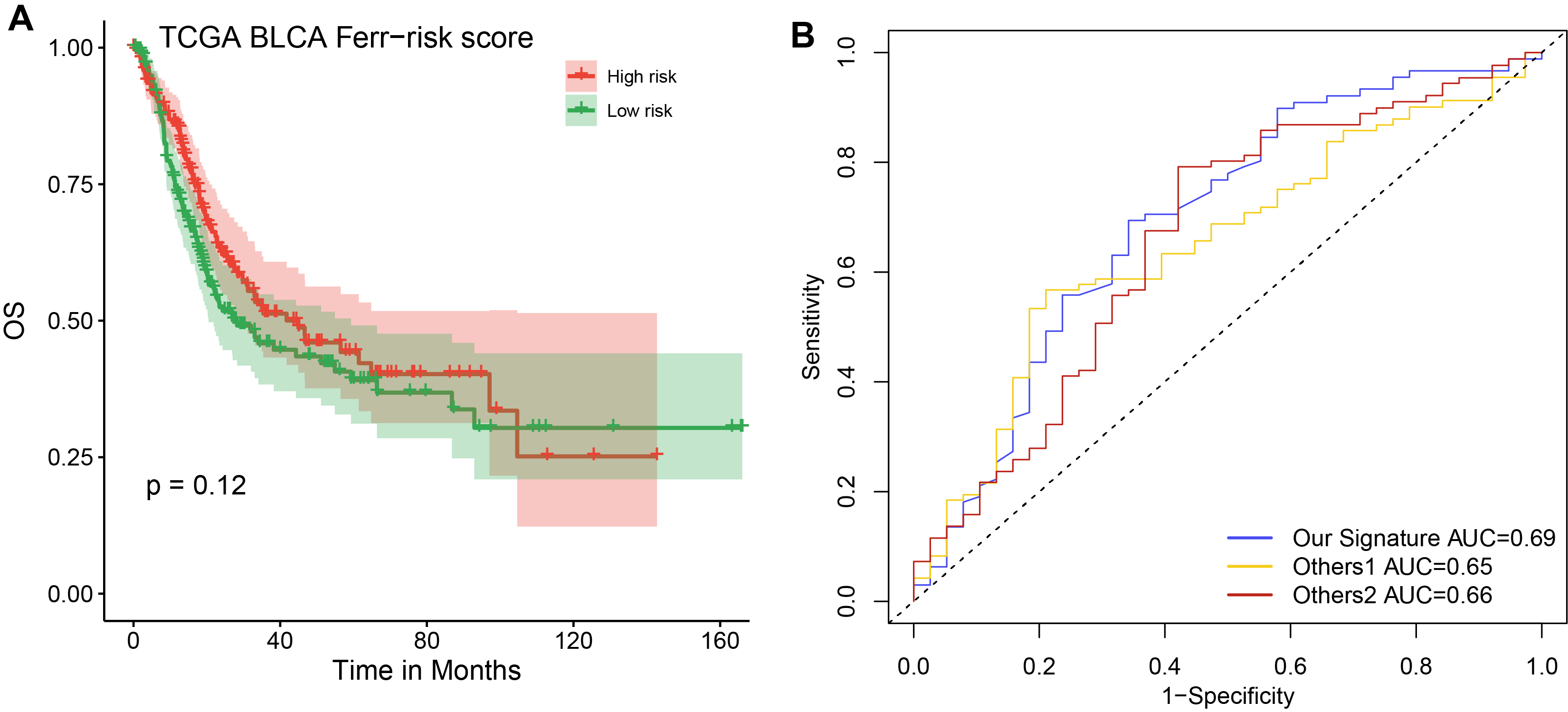


Figure S4. (A) K-M curve of two patient FRGPRS in The Cancer Genome Atlas (TCGA) Bladder Urothelial Carcinoma (BLCA), (B) ROC curve analysis of FRGPRS model and known models.


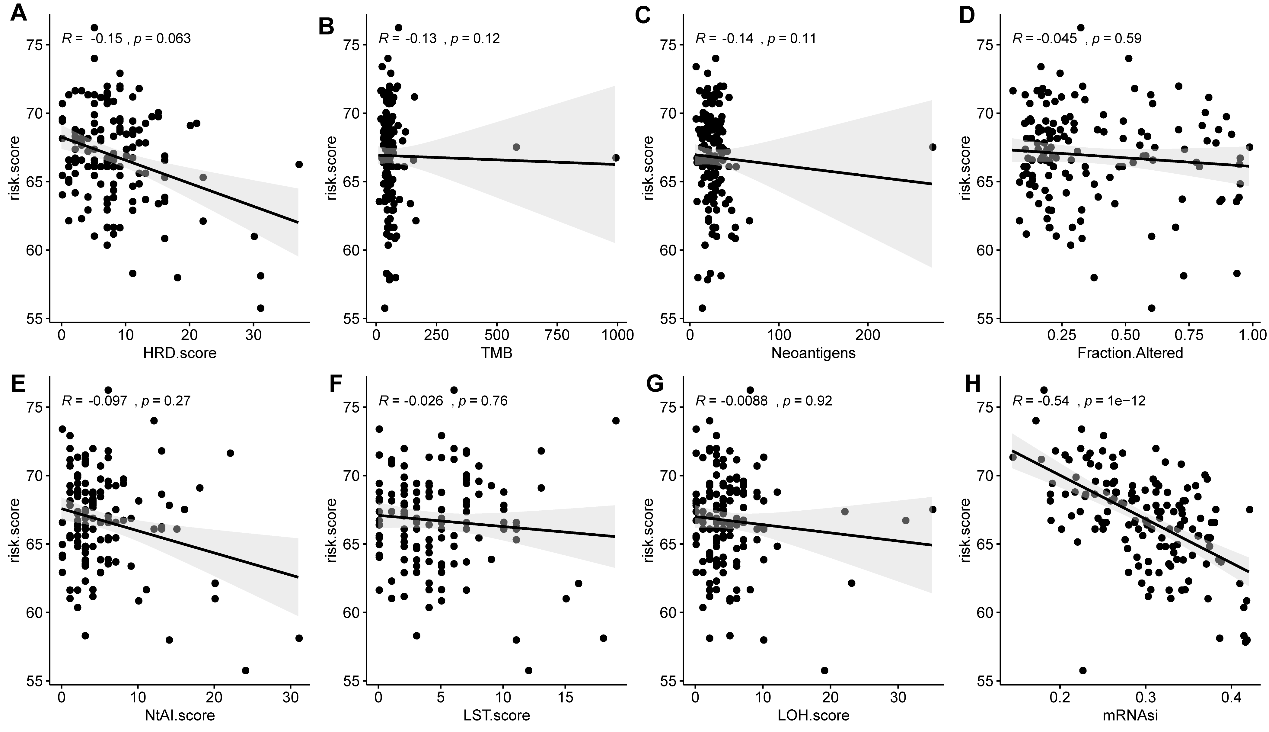


**Figure S5. FRGPRS associated with genomic characteristics**. (A) HRD score. (B) TMB. (C) Neoantigens. (D) Fractions altered. (E, F, G) Chromosome instability. (H) Stemness index (mRNAsi). HRD: Homologous Recombination Deficiency; TMB: Tumor Mutational Burden.

HRD score (r = -0∙15; p = 0∙063) and stemness index (mRNAsi) (r = -0∙54; p=1E-12) were negatively correlated with FRGPRS (Figures S5A and S5B), whereas other genomic characteristics were not significantly correlated with it.
