# Supplementary material for: A Ferroptosis-Related Prognostic Risk Score Model to Predict Clinical Significance and Immunogenic Characteristics in Glioblastoma Multiforme
Source: Oxid Med Cell Longev. 2021 Nov 9;2021:9107857. doi: 10.1155/2021/9107857 (PMC8596022; doi:10.1155/2021/9107857)
Supplement: Supplementary 2 — Table S1: DEGs between GBM and normal brain tissue. Table S2: KEGG pathways enriched in ferroptosis-related genes. Table S3: GO enrichment analysis of molecular function (MF). Table S4: GO enrichment analysis of biological process (BP). Table S5: GO enrichment analysis of cellular component (CC). Table S6: cd-Ferr-Geneset1. Table S7: cd-Ferr-geneset2. Table S8: DEG.Subtype1. Table S9: DEG.Subtype2. Table S10: DEG.Subtype3. Table S11: DEG.Subtype4. Table S12: known ferroptosis genes. Table S13: a multifactor regulatory network of the ferroptosis key hub genes. Table S14: Lasso-logistic regression analysis of prognosis factors. Table S15: FRGPRS model applied for TCGA GBM and GSE4412 GBM dataset. [file 9107857.f2.zip › Table S1.pdf]

DEGs between GBM and normal brain tissue

ID	baseMean	log2FoldChange	lfcSE	stat	pvalue	padj
TRIL	5.416280946	5.950221855	0.16817504	35.38112349	3.33E-274	4.59E-270
CCDC103	5.118005203	5.57678714	0.168441561	33.10814214	2.27E-240	1.56E-236
SNORA8	4.201163218	5.583810264	0.169324385	32.9770001	1.74E-238	7.96E-235
C2orf74	4.123667364	5.557055693	0.16943758	32.79706726	6.48E-236	2.23E-232
AMIGO3	3.851862367	5.458693175	0.16980714	32.14642908	9.91E-227	2.73E-223
C14orf169	3.640891682	5.377272671	0.170149536	31.60321672	3.33E-219	7.65E-216
GCOM1	3.435092559	5.293769429	0.170641807	31.02269911	2.66E-211	5.24E-208
RPS17	8.075885155	2.095001953	0.072984147	28.70489054	3.32E-181	5.70E-178
MAGED4B	7.324704436	2.292707071	0.079927111	28.68497369	5.87E-181	8.98E-178
DDX47	5.809080537	3.458869335	0.12085366	28.62031089	3.76E-180	5.17E-177
GJD3	3.128709778	4.858470672	0.171521193	28.3257747	1.66E-176	2.08E-173
MFRP	5.721075378	3.07919207	0.109902841	28.01740202	9.97E-173	1.14E-169
MRPL30	5.480560101	3.386339912	0.121911242	27.77709315	8.20E-170	8.69E-167
SPDYE8P	2.600736854	4.729814347	0.172622215	27.39980107	2.76E-165	2.71E-162
ADAM6	3.554959805	4.667839151	0.171939495	27.14814976	2.66E-162	2.44E-159
RGPD6	5.199559321	3.22231464	0.1196137	26.93934415	7.60E-160	6.54E-157
ANXA2P2	7.174757362	2.080216017	0.077252631	26.92744544	1.05E-159	8.49E-157
ACAD11	5.028135682	4.497157154	0.168645627	26.66631351	1.16E-156	8.85E-154
EIF3CL	8.639968625	1.745243632	0.065944499	26.46534077	2.43E-154	1.76E-151
MAGED4	6.636195007	2.083439233	0.080288701	25.94934541	1.85E-148	1.27E-145
GTF2IP1	9.248102121	1.606444225	0.062244663	25.80854579	7.11E-147	4.66E-144
C11orf71	4.460816196	4.316518291	0.169264163	25.50166679	1.89E-143	1.18E-140
UBD	3.615299307	4.334214093	0.170750883	25.38326016	3.86E-142	2.31E-139
TOP2A	7.171564591	1.883709685	0.074333755	25.34124223	1.12E-141	6.44E-139
IGF2BP3	5.028494258	2.666516738	0.105401747	25.29860086	3.31E-141	1.82E-138
RRM2	6.40660389	2.037686821	0.081010558	25.15334894	1.30E-139	6.87E-137
PBK	5.579311362	2.309523249	0.092059929	25.08717161	6.87E-139	3.50E-136
HIST2H4A	4.518695385	4.198922075	0.168628954	24.90036243	7.37E-137	3.62E-134
FOXD1	4.455849838	3.139602737	0.126450776	24.82865537	4.40E-136	2.09E-133
HIST2H2AA3	4.474045334	3.082279294	0.124203697	24.81632493	5.98E-136	2.74E-133
HOXC4	3.647718087	4.208101185	0.1705028	24.6805401	1.73E-134	7.68E-132
DLGAP5	4.596052596	2.77401228	0.113191854	24.50717237	1.24E-132	5.33E-130
MELK	4.965038044	2.465623207	0.101082699	24.39213859	2.07E-131	8.64E-129
AURKB	4.750193698	2.563286103	0.105723051	24.24529064	7.41E-130	3.00E-127
POTEE	1.969336361	4.24032612	0.175420495	24.17235302	4.35E-129	1.71E-126
TRIM34	4.287732671	2.88196274	0.120330548	23.95038328	9.16E-127	3.50E-124
CEBPA	6.180386607	1.930653934	0.080692308	23.92612121	1.64E-126	6.09E-124
MYBL2	6.15441633	1.938501644	0.081066712	23.91242478	2.27E-126	8.24E-124
GPR89C	4.017571714	3.580264947	0.150527723	23.78475452	4.80E-125	1.69E-122
SUMO1P3	5.244423144	2.191897211	0.092369928	23.72955426	1.79E-124	6.15E-122
SERF1A	6.69256499	1.7776187	0.075351867	23.59090461	4.78E-123	1.60E-120
GTF2IRD2P1	5.244700246	2.152603153	0.091592012	23.50208383	3.88E-122	1.27E-119
UBE2C	6.046913569	1.910924705	0.081352916	23.48932025	5.24E-122	1.68E-119
KIF20A	5.265235111	2.13659226	0.091171107	23.43497104	1.88E-121	5.88E-119
FAM111B	4.516230829	2.440462229	0.105338522	23.16780399	9.62E-119	2.94E-116
SCARNA17	1.632195879	4.113575814	0.178018634	23.10755747	3.89E-118	1.16E-115
GOLGA6L9	3.77572818	3.647391947	0.158285933	23.04305804	1.73E-117	5.06E-115
DEXI	6.389817703	1.764525586	0.076887336	22.94949558	1.49E-116	4.27E-114
CIDEB	5.012613181	2.14425263	0.09349846	22.93356098	2.15E-116	6.04E-114
APOC2	7.061629396	1.632475919	0.071686923	22.77229717	8.63E-115	2.38E-112
TBC1D3B	3.743787697	3.107252818	0.136591997	22.74842515	1.49E-114	4.01E-112
EDARADD	4.897360255	2.145696209	0.094712843	22.65475453	1.25E-113	3.31E-111
DGKH	6.401566872	-1.791600419	0.079132258	-22.64058263	1.73E-113	4.49E-111
HJURP	5.213444962	2.024856297	0.089633954	22.59028189	5.40E-113	1.38E-110
HOXA10	4.195994286	2.526970247	0.112122165	22.53765116	1.77E-112	4.44E-110
HOXA7	4.094749486	3.117360184	0.139111129	22.4091358	3.21E-111	7.88E-109
FAM64A	5.861942887	1.794567088	0.08083204	22.20118518	3.35E-109	8.08E-107
SHOX2	4.797840314	2.111425313	0.095304186	22.15459152	9.42E-109	2.24E-106
NEIL3	3.612204064	2.972723292	0.134343306	22.127811	1.71E-108	3.98E-106
TBC1D3	5.26050827	1.936163616	0.087502099	22.12705326	1.74E-108	3.98E-106
NDC80	5.255334721	1.934235321	0.08765501	22.06645493	6.64E-108	1.50E-105
HOXD10	3.269166188	3.801704216	0.172628811	22.02242027	1.76E-107	3.90E-105
MKI67	6.475547626	1.650010716	0.074979858	22.00605288	2.52E-107	5.50E-105
NACA2	2.688879449	3.80208434	0.172798292	22.00302032	2.69E-107	5.79E-105
CEP55	4.668605835	2.115889572	0.096481346	21.930556	1.33E-106	2.81E-104
SLIT1	6.897300886	1.568326947	0.072026545	21.77429092	4.07E-105	8.48E-103
ISCA1P1	3.730907906	2.613264446	0.120538375	21.67993758	3.17E-104	6.52E-102
CCNB2	5.916171159	1.68286384	0.078855999	21.34097415	4.73E-101	9.57E-99
IBSP	4.307729209	2.347903617	0.110067109	21.33156428	5.78E-101	1.15E-98
GOLGA6L10	3.721528023	2.514643706	0.117932835	21.32267656	6.99E-101	1.38E-98
BOLA2	7.030735718	1.49272985	0.070054644	21.30807832	9.55E-101	1.85E-98
LYPLA2P1	3.47424282	2.746044052	0.129060734	21.27714581	1.85E-100	3.53E-98
HIST1H4J	3.232301827	3.174285187	0.149920127	21.17317564	1.69E-99	3.18E-97
E2F2	4.619929317	2.010451272	0.094990952	21.16466066	2.02E-99	3.76E-97
GMPPB	5.424228344	1.761808592	0.083375598	21.13098602	4.13E-99	7.58E-97
MGAT2	6.68562994	1.515043162	0.072072548	21.02108491	4.21E-98	7.62E-96
FAM45B	4.658830895	1.957758548	0.093393535	20.96246317	1.44E-97	2.58E-95
MRC1	4.278910945	2.139891464	0.102303068	20.91717786	3.74E-97	6.59E-95

SLC24A2	7.880531908	-1.367146132	0.066340149	-20.60812573	2.32E-94	4.04E-92
PPP1R2P3	3.644846466	2.369230479	0.115133364	20.57813999	4.31E-94	7.41E-92
DEPDC1	4.956799425	1.818871519	0.088404835	20.57434426	4.66E-94	7.92E-92
CENPA	4.582713346	1.88728621	0.092970533	20.2998321	1.29E-91	2.17E-89
CHRNA9	3.575357741	2.387627039	0.118458739	20.15576944	2.40E-90	3.97E-88
POLR2J2	5.748575155	1.583967642	0.078791652	20.10324196	6.91E-90	1.13E-87
MIF	9.326054765	1.168244391	0.058164865	20.08505308	9.97E-90	1.61E-87
FEM1A	7.119978781	1.372285786	0.068353451	20.07632057	1.19E-89	1.90E-87
MMP19	6.513428525	1.451550628	0.072400408	20.04892886	2.06E-89	3.26E-87
BIRC5	6.287964485	1.484452194	0.074068323	20.04166055	2.39E-89	3.73E-87
CAMK4	7.928774634	-1.307566934	0.06552723	-19.95455847	1.37E-88	2.12E-86
CTNNA3	6.5554731	-1.480733273	0.074272285	-19.93655195	1.96E-88	3.00E-86
GRIN1	9.601689369	-1.171118086	0.058746908	-19.93497416	2.02E-88	3.06E-86
CCL4L2	3.581152873	2.301002927	0.11607772	19.82295078	1.89E-87	2.82E-85
GATSL1	2.741077076	3.405789303	0.171944902	19.80744563	2.57E-87	3.80E-85
HOXD9	3.579113114	2.290342195	0.115656492	19.80297136	2.81E-87	4.11E-85
CHI3L2	8.54717661	1.222996278	0.061791308	19.79236753	3.46E-87	5.02E-85
C17orf100	4.740043106	1.763395743	0.089213323	19.76605837	5.84E-87	8.37E-85
IDI2	3.125302843	2.557697406	0.129937235	19.68409904	2.95E-86	4.19E-84
GSC	3.115798505	2.585365327	0.131523961	19.65699102	5.04E-86	7.07E-84
ASF1B	6.149101916	1.452712783	0.074499189	19.49971275	1.10E-84	1.53E-82
SGOL1	4.080186581	1.939766966	0.099593786	19.47678709	1.73E-84	2.38E-82
TROAP	5.363307901	1.581514454	0.081393442	19.43048993	4.26E-84	5.81E-82
E2F8	3.810241849	2.03991158	0.105064295	19.41584032	5.67E-84	7.65E-82
NCAPG	6.120290105	1.449009542	0.074668858	19.40580814	6.89E-84	9.21E-82
PNMA6A	4.618381485	1.770423985	0.091342814	19.38219222	1.09E-83	1.44E-81
ADAMDEC1	3.213736285	2.470173461	0.127565324	19.36398838	1.55E-83	2.04E-81
CDCA2	4.616030957	1.73286633	0.090085541	19.23578762	1.86E-82	2.41E-80
EME2	7.23299496	-1.311117124	0.068542683	-19.12847673	1.46E-81	1.88E-79
ESCO2	4.79439151	1.673090338	0.08749127	19.1229403	1.63E-81	2.07E-79
SNHG3	3.71023603	2.023927358	0.106021747	19.08973788	3.07E-81	3.88E-79
BUB1	6.133059754	1.41091246	0.074177659	19.02071965	1.15E-80	1.44E-78
HOXA3	3.79302868	2.066417621	0.109595961	18.85487021	2.68E-79	3.32E-77
ESM1	4.920013689	1.63126777	0.086632684	18.82970376	4.31E-79	5.30E-77
DTL	5.823759857	1.437011854	0.076361859	18.81845039	5.33E-79	6.49E-77
APOBEC3B	3.99021955	1.87020154	0.09948543	18.79874813	7.73E-79	9.33E-77
UBE2MP1	5.777331329	1.438093801	0.076605049	18.77283311	1.26E-78	1.51E-76
MAP3K9	7.440316596	-1.25208601	0.067033695	-18.67845741	7.41E-78	8.79E-76
VWA5B2	8.18447686	-1.164239248	0.063121943	-18.44428714	5.80E-76	6.82E-74
CNNM1	7.113814996	-1.268507263	0.068796224	-18.43861757	6.44E-76	7.51E-74
ZBTB37	6.66547963	-1.316222923	0.071410054	-18.43189919	7.29E-76	8.43E-74
PGAM4	4.689421658	1.606753746	0.087312992	18.40222975	1.26E-75	1.45E-73
CACNA1I	7.228238703	-1.246242839	0.068028709	-18.3193664	5.80E-75	6.59E-73
PLGLB2	3.199844162	2.141780124	0.117023402	18.30215231	7.95E-75	8.97E-73
SCXB	1.810646215	3.249697742	0.177824574	18.27473933	1.32E-74	1.47E-72
HOXA1	3.027801481	2.272593147	0.124610842	18.23752338	2.60E-74	2.89E-72
HOXD8	3.535650414	2.172234088	0.119401116	18.19274519	5.89E-74	6.49E-72
PA2G4P4	5.10584661	1.494943095	0.082196951	18.18733021	6.50E-74	7.10E-72
MMP9	6.407930757	1.303335518	0.071830278	18.14465357	1.42E-73	1.53E-71
PDZD7	6.807345362	-1.272864703	0.070238103	-18.12213961	2.13E-73	2.29E-71
ZNF593	6.089292911	1.329574743	0.073484115	18.09336257	3.59E-73	3.83E-71
IQSEC3	8.681240081	-1.100630272	0.060861786	-18.08409422	4.25E-73	4.50E-71
EIF5AL1	8.021808795	1.127814697	0.062391887	18.07630356	4.90E-73	5.15E-71
MAGEL2	3.664131038	1.884391116	0.10425977	18.07400029	5.11E-73	5.32E-71
NKX2-5	3.812881207	1.884746342	0.104537478	18.02938404	1.15E-72	1.19E-70
RLTPR	7.365789444	-1.204062111	0.066914699	-17.99398538	2.17E-72	2.23E-70
E2F7	5.098727885	1.478608159	0.082220982	17.98334339	2.63E-72	2.68E-70
MYH7B	6.788214934	-1.260455216	0.070247672	-17.94301767	5.44E-72	5.51E-70
ESYT3	5.91091955	-1.372667876	0.076523243	-17.93792078	5.96E-72	5.99E-70
HOXA11	2.459370328	2.983634816	0.166338379	17.93714012	6.05E-72	6.03E-70
TTK	5.377260992	1.420712335	0.079271073	17.92220402	7.91E-72	7.83E-70
CORO6	7.73026075	-1.163720911	0.064934938	-17.9213371	8.04E-72	7.90E-70
PRKCG	7.686850113	-1.170104798	0.065419057	-17.88629885	1.51E-71	1.47E-69
CKAP2L	5.344064796	1.422443006	0.079534215	17.88466769	1.55E-71	1.50E-69
ATP5EP2	5.489672555	1.39387907	0.078126243	17.84136825	3.37E-71	3.25E-69
CTAGE5	6.053027372	1.30879564	0.073439619	17.82138378	4.82E-71	4.61E-69
CPXM1	7.692089038	1.138683497	0.063953569	17.8048467	6.48E-71	6.15E-69
FOXD3	2.553770292	2.613732163	0.147001302	17.78033342	1.00E-70	9.46E-69
PTRH1	5.131075729	1.445591466	0.081354173	17.76911273	1.23E-70	1.15E-68
ALX3	3.711143943	1.807092778	0.101860505	17.74085825	2.03E-70	1.89E-68
PPARGC1B	6.222942101	-1.309397366	0.073827388	-17.73592962	2.21E-70	2.04E-68
ANKRD22	4.833751554	1.509171978	0.085227011	17.70767227	3.66E-70	3.36E-68
SPOCD1	7.591934901	1.143440054	0.064598286	17.70078012	4.14E-70	3.77E-68
RGPD4	4.223112906	1.634320938	0.09236769	17.6936431	4.69E-70	4.25E-68
H3F3C	5.050061527	1.44670723	0.08203973	17.63422715	1.35E-69	1.21E-67
RGPD1	4.730584393	1.507062004	0.085674209	17.59061467	2.91E-69	2.60E-67
METTL7B	8.21830489	1.083331418	0.061612858	17.58287896	3.33E-69	2.96E-67
TRIM17	6.205139411	-1.298873539	0.073966078	-17.56039482	4.95E-69	4.37E-67
NEFM	8.96639358	-1.048511845	0.059723814	-17.55600942	5.35E-69	4.69E-67
ATP2B3	7.322474695	-1.177056264	0.067054242	-17.55379277	5.56E-69	4.85E-67

KIFC1	6.373995701	1.246672574	0.071073448	17.54062331	7.02E-69	6.07E-67
CDC45	5.36964095	1.382997253	0.078917947	17.52449619	9.32E-69	8.01E-67
POSTN	7.193276632	1.188024823	0.067826152	17.51573383	1.09E-68	9.29E-67
PDZK1P1	2.210561855	3.02405581	0.173001603	17.4799294	2.04E-68	1.73E-66
SIGLEC7	4.493429874	1.543165776	0.088656478	17.40612535	7.41E-68	6.26E-66
GABRG1	7.794552972	-1.121006949	0.064598344	-17.35349353	1.86E-67	1.56E-65
TBC1D3H	1.742965025	3.103024999	0.17887223	17.34771798	2.05E-67	1.71E-65
CSRNP3	7.167383053	-1.170720327	0.067490942	-17.34633265	2.10E-67	1.74E-65
FAM156A	7.523775547	1.113800028	0.064275398	17.32855903	2.86E-67	2.35E-65
H2BFXP	2.658432244	2.307509396	0.133161267	17.32868314	2.86E-67	2.35E-65
NPM2	7.040102406	-1.181907793	0.068282879	-17.30899174	4.02E-67	3.28E-65
MAP3K13	7.482364023	-1.133394645	0.065692396	-17.25305698	1.06E-66	8.54E-65
RGPD5	3.433810835	1.822429349	0.10614261	17.16963007	4.48E-66	3.59E-64
GLS2	7.119801645	-1.159365908	0.067628552	-17.14314244	7.07E-66	5.63E-64
HSFX2	2.768560096	2.163347492	0.126287608	17.1303228	8.82E-66	6.97E-64
GFOD1	8.108344267	-1.071318022	0.062593388	-17.11551426	1.14E-65	8.94E-64
PEX5L	7.754291327	-1.104508935	0.064548551	-17.11128944	1.22E-65	9.56E-64
GSG2	3.793001732	1.682296513	0.098465285	17.0851738	1.91E-65	1.49E-63
HOXA5	4.04552199	1.705353821	0.100073605	17.04099524	4.08E-65	3.15E-63
SVOP	7.627647747	-1.111965177	0.065321479	-17.02296377	5.55E-65	4.27E-63
GGTLC2	1.881180109	3.012336818	0.177090726	17.0101331	6.91E-65	5.28E-63
RELN	7.762913071	-1.098578884	0.064654083	-16.99163959	9.47E-65	7.20E-63
MCM10	4.835803074	1.420056983	0.083663684	16.97339765	1.29E-64	9.77E-63
CDYL2	7.153649786	-1.141148609	0.06724848	-16.96913617	1.39E-64	1.05E-62
C2orf82	6.044962361	-1.264194563	0.074608918	-16.94428223	2.12E-64	1.59E-62
RPL36A	8.67649767	-1.018266204	0.060164149	-16.92480025	2.95E-64	2.20E-62
PDIA2	8.005532882	-1.06748823	0.063149223	-16.90421791	4.19E-64	3.10E-62
CYP51A1	8.529454189	1.007435045	0.059685867	16.87895459	6.43E-64	4.70E-62
LRRC8E	2.124975578	2.938360933	0.174132264	16.87430502	6.95E-64	5.06E-62
C21orf62	7.321871522	1.101690879	0.065376814	16.8514005	1.02E-63	7.42E-62
LRRC7	6.711198508	-1.180147022	0.070174376	-16.81734983	1.82E-63	1.31E-61
HOXA4	3.879743914	2.075564306	0.123569005	16.79680358	2.58E-63	1.84E-61
SCRT1	8.441360964	-1.028540058	0.06124754	-16.79316524	2.74E-63	1.94E-61
F2R	8.515962677	1.004452283	0.059818407	16.79169226	2.81E-63	1.98E-61
KCNB1	7.875274219	-1.064647769	0.063550164	-16.75287203	5.40E-63	3.79E-61
CDK1	6.893849607	1.122518619	0.067282863	16.68357394	1.73E-62	1.20E-60
DPEP1	4.564566263	1.444916385	0.086880641	16.63105113	4.15E-62	2.87E-60
TRPM3	7.728095976	-1.062984458	0.064156242	-16.56868324	1.17E-61	8.08E-60
GK3P	2.695759491	2.072154179	0.125655735	16.49072513	4.28E-61	2.93E-59
HOXB7	3.949862079	1.737856592	0.105982393	16.39759719	1.99E-60	1.34E-58
FPR3	5.673888305	1.237107537	0.075507553	16.38389116	2.49E-60	1.67E-58
ESPL1	5.576677479	1.242926889	0.075946065	16.3659156	3.35E-60	2.23E-58
USP49	6.732325111	-1.13188787	0.069230569	-16.34953864	4.38E-60	2.90E-58
SNURF	5.694041722	1.223966556	0.074877412	16.34627208	4.62E-60	3.04E-58
MYT1L	8.134394823	-1.017222475	0.062431584	-16.29339534	1.10E-59	7.21E-58
CLDN9	5.444764973	-1.281662312	0.07874702	-16.27569281	1.47E-59	9.58E-58
CENPF	7.507927259	1.036767486	0.06388435	16.22881793	3.15E-59	2.04E-57
ASPM	5.98006119	1.180935184	0.07283651	16.21350598	4.05E-59	2.60E-57
EEF1A1P9	6.213849542	1.152556425	0.071093763	16.2117798	4.16E-59	2.66E-57
NKX3-2	2.691021079	2.037904326	0.125930926	16.18271529	6.68E-59	4.25E-57
KIF14	5.119425262	1.286273674	0.079736356	16.13158328	1.53E-58	9.71E-57
LCOR	6.741236216	-1.111118307	0.068997551	-16.10373544	2.40E-58	1.51E-56
AJAP1	7.261161258	-1.064924417	0.066177608	-16.09191456	2.91E-58	1.82E-56
FAM27C	2.121399613	2.513537733	0.15623575	16.08810868	3.09E-58	1.93E-56
FOXO3B	6.664602944	1.096095994	0.068144595	16.08485587	3.26E-58	2.02E-56
RUNX1T1	7.218816674	-1.066754156	0.066357341	-16.07590266	3.76E-58	2.32E-56
KCNQ4	6.133526191	-1.172004091	0.072980933	-16.05904497	4.94E-58	3.04E-56
GTSE1	5.97351173	1.165489901	0.072628271	16.04733104	5.97E-58	3.65E-56
TRPV3	5.98354279	-1.186970324	0.074036024	-16.03233472	7.60E-58	4.63E-56
FAT2	5.649268598	-1.392281888	0.087073261	-15.98977547	1.51E-57	9.13E-56
CCDC64	7.6281183	-1.026967246	0.064259157	-15.98164821	1.72E-57	1.04E-55
UNC5C	6.884552316	-1.085289892	0.068186551	-15.91648032	4.87E-57	2.90E-55
POTEF	2.656284376	1.970739292	0.124024884	15.88987013	7.45E-57	4.42E-55
HOXC6	3.441272818	1.674829326	0.105437922	15.8845062	8.11E-57	4.79E-55
KIAA0101	6.129582791	1.136673797	0.071570062	15.88197308	8.45E-57	4.97E-55
CCL3	4.988106279	1.297829198	0.081885575	15.84930191	1.42E-56	8.32E-55
ANXA2P1	4.107077565	1.442547264	0.091052218	15.8430766	1.57E-56	9.15E-55
NEK2	5.62093736	1.199633423	0.075811744	15.82384683	2.13E-56	1.24E-54
CALB2	7.720495407	-1.012318749	0.064069509	-15.80031995	3.10E-56	1.79E-54
IQGAP3	6.165851707	1.12647082	0.071300191	15.7989873	3.16E-56	1.82E-54
RANBP17	4.908927367	-1.315572854	0.08338349	-15.77737812	4.45E-56	2.54E-54
ISL2	2.957936006	1.802641523	0.114293595	15.77202578	4.85E-56	2.76E-54
SULT1A3	6.854294903	1.048364115	0.066839417	15.68481832	1.92E-55	1.09E-53
RNF152	6.87677777	-1.062320761	0.067967754	-15.62977591	4.56E-55	2.57E-53
SPHKAP	6.935522644	-1.065224243	0.068232222	-15.61174773	6.06E-55	3.40E-53
CDC25C	4.724582355	1.296526036	0.083108192	15.60046017	7.23E-55	4.03E-53
CPZ	3.386375293	1.646303888	0.105538992	15.59901091	7.39E-55	4.10E-53
EXO1	5.249553428	1.217009398	0.078045042	15.59367982	8.04E-55	4.44E-53
CRHR1	6.35224494	-1.110535536	0.071309856	-15.57338071	1.10E-54	6.08E-53
FAM131C	6.704982813	-1.07373902	0.068991653	-15.56331787	1.29E-54	7.09E-53

MPPED1	6.409165168	-1.117133032	0.07193301	-15.53018612	2.17E-54	1.17E-52
GPR83	5.767256237	-1.168303578	0.075544564	-15.46509134	5.97E-54	3.21E-52
ZNF90	7.068184969	1.014642499	0.06561806	15.4628543	6.18E-54	3.31E-52
CALB1	7.149142467	-1.032571116	0.066897508	-15.43512076	9.50E-54	5.01E-52
MAP1LC3B2	7.046235232	1.009556214	0.065664381	15.37448771	2.43E-53	1.28E-51
CLDN23	3.51256464	1.532604565	0.100006008	15.32512486	5.20E-53	2.71E-51
CYP19A1	4.213897232	1.372825582	0.089648675	15.31339514	6.22E-53	3.22E-51
CDC20	7.048828989	1.005551807	0.065677544	15.3104357	6.51E-53	3.36E-51
MFAP2	5.029157418	1.223823508	0.080068397	15.28472595	9.67E-53	4.96E-51
OR2A9P	3.334403155	1.580243608	0.103406469	15.28186417	1.01E-52	5.15E-51
SRPX2	6.753147902	1.029489417	0.067432819	15.26688971	1.27E-52	6.45E-51
TAS2R14	4.887733922	-1.262194452	0.082689569	-15.26425232	1.32E-52	6.70E-51
PPP4R4	6.796583247	-1.040569958	0.068242846	-15.24804453	1.70E-52	8.55E-51
KIAA1984	5.005301233	-1.239667027	0.081459131	-15.21826979	2.68E-52	1.34E-50
GPR65	5.605279869	1.141137231	0.075014838	15.2121536	2.94E-52	1.46E-50
PABPC3	6.344884826	1.057016169	0.069556475	15.1965172	3.73E-52	1.85E-50
MPP7	5.817787804	-1.129805169	0.074512867	-15.16255123	6.26E-52	3.09E-50
KIF18A	5.101705474	1.19559759	0.078920578	15.14937708	7.65E-52	3.75E-50

C10orf128	5.586116522	-1.155419674	0.076465982	-15.11024437	1.39E-51	6.72E-50
GPR82	3.782174196	1.434026815	0.094962326	15.10100772	1.59E-51	7.70E-50
GRAP	5.858522451	1.096203362	0.072757724	15.06648781	2.69E-51	1.29E-49
HOXA2	3.118078227	2.182695757	0.145037155	15.04921793	3.49E-51	1.67E-49
RBMS3	5.893846264	-1.111569764	0.073882045	-15.04519484	3.71E-51	1.77E-49
CYP4X1	6.289956277	-1.065551966	0.071021759	-15.00317633	7.00E-51	3.32E-49
CNGA3	6.571954047	1.031188221	0.068765143	14.99579836	7.82E-51	3.70E-49
VTN	5.432853051	-1.159403972	0.077357308	-14.98764623	8.84E-51	4.15E-49
GJA9	1.745357236	2.634941904	0.17634378	14.94207454	1.75E-50	8.18E-49
ZFR2	6.807657543	-1.018402165	0.06815791	-14.94180457	1.76E-50	8.19E-49
MYO15A	5.722150442	-1.117298752	0.075006261	-14.89607326	3.50E-50	1.62E-48
CLEC5A	6.047922906	1.073986632	0.072124925	14.89064477	3.79E-50	1.75E-48
MEOX2	6.476521173	1.025408392	0.069023679	14.85589294	6.37E-50	2.90E-48
SKA3	5.763174962	1.088535918	0.073303101	14.8497935	6.98E-50	3.17E-48
S1PR2	5.976339271	1.064270632	0.071746491	14.83376571	8.86E-50	4.00E-48
LEFTY2	4.913676826	1.196728263	0.080706164	14.82821383	9.63E-50	4.33E-48
GSTO2	6.279118094	-1.052550191	0.070997801	-14.82510966	1.01E-49	4.52E-48
CASC5	5.007826412	1.178272303	0.079490563	14.82279472	1.04E-49	4.66E-48
HIST1H3E	3.638497792	1.427256097	0.096607924	14.7736959	2.17E-49	9.55E-48
OIP5	4.896150168	1.188203823	0.080430199	14.77310567	2.18E-49	9.60E-48
ADARB2	6.451782337	-1.031482426	0.070051842	-14.72455814	4.48E-49	1.95E-47
PIPSL	5.816126229	1.069765416	0.072701163	14.71455705	5.20E-49	2.25E-47
NR5A2	4.006080497	1.338750062	0.091063509	14.70127914	6.33E-49	2.72E-47
TNK1	5.26414867	-1.157618524	0.078823267	-14.68625398	7.90E-49	3.39E-47
BUB1B	6.235208432	1.022828932	0.06993223	14.6260019	1.92E-48	8.12E-47
KIF18B	6.228825592	1.025634577	0.070139109	14.62286297	2.01E-48	8.45E-47
SKA1	5.308471946	1.122284405	0.076761264	14.62045244	2.08E-48	8.70E-47
AURKAPS1	1.81909518	2.391727728	0.163891415	14.59336802	3.10E-48	1.28E-46
NOXO1	1.883401205	2.298739148	0.15753201	14.5922035	3.15E-48	1.30E-46
FNDC8	2.966252331	1.58718167	0.109403456	14.5076008	1.08E-47	4.40E-46
TREM1	5.20431703	1.139376542	0.078648873	14.48687694	1.47E-47	5.94E-46
KIF23	6.264841492	1.00894536	0.069650992	14.48572856	1.49E-47	6.02E-46
SPC25	5.630144804	1.073710316	0.074183048	14.47379618	1.77E-47	7.12E-46
SLC6A13	5.95588135	-1.056868259	0.073031003	-14.47150143	1.83E-47	7.34E-46
SYTL1	5.63817459	-1.09737649	0.076258594	-14.39020094	5.96E-47	2.37E-45
PTCRA	2.215431299	1.95532525	0.136242165	14.35183635	1.04E-46	4.11E-45
TRPM8	4.350932697	1.238882262	0.086502942	14.32185115	1.60E-46	6.30E-45
RNF43	5.617624665	-1.078992044	0.075598832	-14.27260206	3.24E-46	1.27E-44
KCNK4	5.857148272	-1.049938191	0.073573668	-14.27057022	3.34E-46	1.30E-44
NME2P1	4.273065533	1.230736832	0.086534825	14.22244548	6.65E-46	2.56E-44
SLC10A5	4.090551287	-1.28561447	0.090603225	-14.18950016	1.06E-45	4.06E-44
FCGR2B	5.672253943	1.04484249	0.074063588	14.10737064	3.42E-45	1.28E-43
EMR1	3.823015917	1.308195238	0.093365847	14.01149642	1.33E-44	4.88E-43
MTUS2	5.44413365	-1.070931028	0.076455196	-14.00730218	1.41E-44	5.16E-43
SERTAD4	5.800670706	-1.033302148	0.073771027	-14.00688303	1.41E-44	5.18E-43
HMMR	5.756466165	1.013815427	0.072764635	13.93280445	4.00E-44	1.43E-42
ARHGEF33	5.63512772	-1.03530022	0.074720843	-13.85557474	1.18E-43	4.15E-42
C19orf71	5.112846929	-1.090221277	0.07896913	-13.80566409	2.36E-43	8.25E-42
CXCL9	4.388995956	1.180187891	0.08640454	13.65886433	1.79E-42	6.09E-41
SERHL	4.68462588	1.104718957	0.08133189	13.58285113	5.06E-42	1.69E-40
CTAGE1	1.722956463	2.174013144	0.1605427	13.54165054	8.88E-42	2.94E-40
PDCD1LG2	5.17495391	1.044857292	0.07717424	13.53893854	9.21E-42	3.05E-40
ABHD12B	4.995699896	-1.084667434	0.080155947	-13.53196459	1.01E-41	3.34E-40
TLR8	4.382094321	1.151167388	0.085135092	13.52165559	1.17E-41	3.84E-40
TDRD9	5.4300855	-1.008087674	0.075948633	-13.27328269	3.31E-40	1.04E-38
RNASE2	5.153266983	1.024450529	0.077382987	13.23870489	5.24E-40	1.64E-38
RHOV	4.241128145	-1.15137102	0.087436646	-13.16806022	1.34E-39	4.10E-38
SLC16A8	4.959222746	-1.047268707	0.079815858	-13.12106058	2.49E-39	7.51E-38
HNRNPA3P1	2.587459725	1.520361402	0.115926302	13.11489612	2.71E-39	8.09E-38
HOXB4	3.420787734	1.58157839	0.120611503	13.11299791	2.77E-39	8.28E-38
HS3ST3A1	3.793966284	1.222895357	0.093522659	13.07592589	4.52E-39	1.34E-37
ALPK2	3.437346212	1.292211157	0.098833424	13.07463719	4.60E-39	1.36E-37
PLEK2	4.27327262	1.110579663	0.085359426	13.01062713	1.06E-38	3.12E-37
TNFSF8	4.28092923	1.112112253	0.085694555	12.97763037	1.64E-38	4.78E-37
IRF6	5.18795769	-1.008981443	0.078062208	-12.92535115	3.24E-38	9.30E-37
ULBP3	3.368968785	1.273773039	0.098808337	12.89135182	5.04E-38	1.44E-36
C15orf48	3.607510922	1.20965343	0.094314605	12.82572757	1.18E-37	3.28E-36
F2RL2	4.23949376	1.143273908	0.089509098	12.77271173	2.33E-37	6.40E-36
ARL11	4.753025966	1.02011564	0.080269129	12.70869211	5.29E-37	1.43E-35
DMRTC1B	4.185088014	1.090363875	0.086023783	12.67514442	8.12E-37	2.18E-35
HMGA2	3.157910635	1.41696219	0.112314288	12.61604573	1.72E-36	4.52E-35
FAAH2	4.744365374	-1.02042367	0.081254325	-12.55839207	3.58E-36	9.32E-35
MST1R	4.029867551	-1.107489514	0.08910229	-12.42941699	1.81E-35	4.63E-34
CD300C	4.601936255	1.012305596	0.081509021	12.4195528	2.05E-35	5.23E-34
MACC1	3.341523381	1.209710815	0.097696448	12.38234187	3.26E-35	8.24E-34
RIMBP3	3.827560136	1.108653518	0.090172365	12.29482583	9.66E-35	2.38E-33
MALL	4.363988498	1.023389143	0.083731679	12.22224558	2.36E-34	5.75E-33
RNF138P1	2.895297248	1.287662376	0.10583171	12.16707525	4.65E-34	1.12E-32
CYP2B7P1	4.458926731	-1.006748379	0.083708019	-12.02690486	2.57E-33	5.91E-32
FAM180A	3.522529336	1.142753079	0.096320788	11.8640338	1.82E-32	4.04E-31

KIF4B	2.662010495	1.31295653	0.110834554	11.84609387	2.25E-32	4.98E-31
OR51E1	3.598895983	1.110991158	0.094117992	11.80423774	3.71E-32	8.16E-31
CCL8	3.137905884	1.205772076	0.102598269	11.75236275	6.87E-32	1.49E-30
TAS1R3	4.279542507	-1.002646777	0.085381204	-11.7431792	7.66E-32	1.66E-30
C10orf55	2.482007086	1.333071822	0.115464925	11.54525346	7.80E-31	1.60E-29
CNGA4	3.778911072	-1.050406697	0.091422122	-11.48963373	1.49E-30	3.01E-29
HSP90B3P	1.561967217	1.774993058	0.156005501	11.37775941	5.40E-30	1.07E-28
OSR2	3.516679619	1.066812735	0.093968692	11.35285286	7.18E-30	1.41E-28
HIST1H3H	2.264712398	1.379999244	0.122008651	11.31066715	1.16E-29	2.26E-28
ABCC6P2	3.252295365	1.094102986	0.097862287	11.18002675	5.11E-29	9.68E-28
SNORA7B	1.889590753	1.476971728	0.134935729	10.94574238	6.96E-28	1.26E-26
RNASE3	3.017438384	1.105313552	0.102577282	10.77542245	4.50E-27	7.73E-26
CD80	2.221164023	1.290921852	0.121492996	10.62548369	2.27E-26	3.79E-25
GJA3	3.064434707	1.146051274	0.108594952	10.55344888	4.90E-26	8.06E-25
MORF4	2.172089261	1.250676289	0.121669457	10.27929537	8.74E-25	1.34E-23
SPATA12	2.124564891	1.168598472	0.12221659	9.561700816	1.16E-21	1.48E-20
ASIP	2.691902049	1.007019281	0.1066468	9.442564438	3.64E-21	4.50E-20
RPL13AP6	2.292049836	1.079806683	0.115997043	9.308915597	1.29E-20	1.54E-19
SIT1	2.335674597	1.017901473	0.115415827	8.819427113	1.15E-18	1.22E-17
RIMBP3C	2.103999109	1.000450662	0.1201102	8.32943963	8.12E-17	7.64E-16
PRH2	1.653146099	1.048781052	0.136068379	7.70775005	1.28E-14	1.04E-13
