# Supplementary material for: A Ferroptosis-Related Prognostic Risk Score Model to Predict Clinical Significance and Immunogenic Characteristics in Glioblastoma Multiforme
Source: Oxid Med Cell Longev. 2021 Nov 9;2021:9107857. doi: 10.1155/2021/9107857 (PMC8596022; doi:10.1155/2021/9107857)
Supplement: Supplementary 2 — Table S1: DEGs between GBM and normal brain tissue. Table S2: KEGG pathways enriched in ferroptosis-related genes. Table S3: GO enrichment analysis of molecular function (MF). Table S4: GO enrichment analysis of biological process (BP). Table S5: GO enrichment analysis of cellular component (CC). Table S6: cd-Ferr-Geneset1. Table S7: cd-Ferr-geneset2. Table S8: DEG.Subtype1. Table S9: DEG.Subtype2. Table S10: DEG.Subtype3. Table S11: DEG.Subtype4. Table S12: known ferroptosis genes. Table S13: a multifactor regulatory network of the ferroptosis key hub genes. Table S14: Lasso-logistic regression analysis of prognosis factors. Table S15: FRGPRS model applied for TCGA GBM and GSE4412 GBM dataset. [file 9107857.f2.zip › Table S10.pdf]

Table S10. DEG.Subtype3

ID	baseMean	log2FoldChange	lfcSE
ADAM6	433.9674759	2.053797054	0.38648907
AGAP2	2367.917609	-1.546818429	0.340772409
AKR1C1	100.317536	1.131827825	0.283121603
AKR1C2	50.63291694	1.908725916	0.291758117
ARHGAP36	107.7587129	-1.471033507	0.358990484
C9orf117	20.37558532	1.591787801	0.281478244
CCDC42B	45.979331	1.375998896	0.361222783
CCT6A	7870.733905	-1.016779411	0.212397159
CHRNA1	306.1261091	-2.896426109	0.416530502
CLVS1	103.7208177	-1.237177387	0.322944761
COL25A1	50.25061558	-1.787408311	0.357604386
CRABP2	188.4353398	-0.899428268	0.225769296
CYB5R1	1671.253405	0.69578486	0.161697739
CYP27B1	111.8015387	-1.507148331	0.345553384
DACH2	28.12708388	1.403157002	0.35664327
DPP4	235.5917014	-2.155257255	0.303028705
ESR2	26.52682862	-1.191952587	0.288795153
GATA3	24.11729526	-1.261901736	0.322868891
GDF15	325.389094	-1.011763463	0.262016857
GPAT2	30.16894853	0.917345226	0.238960718
ITGA10	154.7405343	-0.919773133	0.207064478
MORN5	65.86893121	1.243238619	0.314836014
NOS2	632.3971203	-2.238886387	0.415169667
OS9	13762.88913	-0.894533986	0.234018528
PCDHB4	164.0554817	1.187207199	0.276290946
PCDHGA12	69.43076177	1.232011281	0.284776327
RBP4	43.47500715	1.170327261	0.298540937
RERG	91.79779097	1.144643006	0.265133524
RSPH10B2	21.62959668	1.114944704	0.285064642
SGCE	1699.676194	0.900933953	0.203925319
SLC26A7	43.59353684	-1.530564385	0.30984855
SNRPE	792.0888098	0.858219401	0.181009424
SOSTDC1	16.31035043	1.326850147	0.299147702
TPPP3	2020.959596	1.027001792	0.258294446
WDR66	54.86403741	1.041061223	0.224658245
WFIKKN2	60.91245684	1.723407693	0.365692339
WNK4	55.48054092	-1.324882782	0.302898924
ZCCHC12	158.6829051	1.055573235	0.259823911

stat	pvalue	padj
5.313984821	1.07E-07	0.000246004
-4.539153953	5.65E-06	0.005979101
3.997673831	6.40E-05	0.031440278
6.54215189	6.06E-11	2.78E-07
-4.09769499	4.17E-05	0.022087196
5.655100657	1.56E-08	5.36E-05
3.809280476	0.000139372	0.047950863
-4.787161063	1.69E-06	0.0025866
-6.953695098	3.56E-12	2.45E-08
-3.83092571	0.000127662	0.046233819
-4.99828408	5.78E-07	0.001137188
-3.983837863	6.78E-05	0.032137855
4.302996839	1.69E-05	0.010361444
-4.361549915	1.29E-05	0.009873801
3.934343134	8.34E-05	0.035877763
-7.112386459	1.14E-12	1.57E-08
-4.12732892	3.67E-05	0.021044465
-3.90840298	9.29E-05	0.036531521
-3.861444167	0.000112719	0.043089882
3.838895499	0.000123589	0.045968433
-4.441964846	8.91E-06	0.00843029
3.948844996	7.85E-05	0.034861889
-5.392702229	6.94E-08	0.000191033
-3.822492145	0.00013211	0.046617783
4.296945727	1.73E-05	0.010361444
4.326241918	1.52E-05	0.010354127
3.920156727	8.85E-05	0.036531521
4.317232276	1.58E-05	0.010354127
3.911199565	9.18E-05	0.036531521
4.417960249	9.96E-06	0.008570005
-4.93971776	7.82E-07	0.00134585
4.7412968	2.12E-06	0.002922424
4.435434871	9.19E-06	0.00843029
3.976089342	7.01E-05	0.032137855
4.633977372	3.59E-06	0.004113763
4.712725718	2.44E-06	0.00305798
-4.374009537	1.22E-05	0.009873801
4.062648547	4.85E-05	0.024730341
