# Supplementary material for: A Ferroptosis-Related Prognostic Risk Score Model to Predict Clinical Significance and Immunogenic Characteristics in Glioblastoma Multiforme
Source: Oxid Med Cell Longev. 2021 Nov 9;2021:9107857. doi: 10.1155/2021/9107857 (PMC8596022; doi:10.1155/2021/9107857)
Supplement: Supplementary 2 — Table S1: DEGs between GBM and normal brain tissue. Table S2: KEGG pathways enriched in ferroptosis-related genes. Table S3: GO enrichment analysis of molecular function (MF). Table S4: GO enrichment analysis of biological process (BP). Table S5: GO enrichment analysis of cellular component (CC). Table S6: cd-Ferr-Geneset1. Table S7: cd-Ferr-geneset2. Table S8: DEG.Subtype1. Table S9: DEG.Subtype2. Table S10: DEG.Subtype3. Table S11: DEG.Subtype4. Table S12: known ferroptosis genes. Table S13: a multifactor regulatory network of the ferroptosis key hub genes. Table S14: Lasso-logistic regression analysis of prognosis factors. Table S15: FRGPRS model applied for TCGA GBM and GSE4412 GBM dataset. [file 9107857.f2.zip › Table S12.pdf]

Table S12. Known ferroptosis-genes

RPL8
IREB2
ATP5MC3
CS
EMC2
ACSF2
NOX1
CYBB
NOX3
NOX4
NOX5
DUOX1
DUOX2
G6PD
PGD
VDAC2
PIK3CA
FLT3
SCP2
TP53
ACSL4
LPCAT3
NRAS
KRAS
HRAS
TF
TFRC
TFR2
SLC38A1
SLC1A5
GLS2
GOT1
CARS1
ALOX5
KEAP1
HMOX1
ATG5
ATG7
NCOA4
ALOX12
ALOX12B
ALOX15
ALOX15B
ALOXE3
PHKG2
ACO1
G6PDX
ULK1
ATG3
ATG4D
BECN1
MAP1LC3A
GABARAPL2
GABARAPL1
ATG16L1
WIPI1
WIPI2

SNX4
ATG13
ULK2
SAT1
EGFR
MAPK3
MAPK1
BID
ZEB1
DPP4
CDKN2A
PEBP1
SOCS1
CDO1
MYB
MAPK8
MAPK9
CHAC1
MAPK14
LINC00472
PRKAA2
PRKAA1
ELAVL1
BAP1
ABCC1
MIR6852
ACVR1B
TGFBR1
EPAS1
HILPDA
HIF1A
IFNG
ANO6
LPIN1
HMGB1
TNFAIP3
TLR4
ATF3
ATM
YY1AP1
EGLN2
MIOX
TAZ
MTDH
IDH1
SIRT1
FBXW7
PANX1
DNAJB6
BACH1
LONP1
PTGS2
DUSP1
NOS2
NCF2
MT3
UBC
ALB

TXNRD1
SRXN1
GPX2
BNIP3
OXSR1
SELENOS
ANGPTL7
SLC7A11
DDIT4
LOC284561
ASNS
TSC22D3
DDIT3
JDP2
SESN2
SLC1A4
PCK2
TXNIP
VLDLR
GPT2
PSAT1
LURAP1L
SLC7A5
HERPUD1
XBP1
SLC3A2
CBS
ATF4
ZNF419
KLHL24
TRIB3
ZFP69B
ATP6V1G2
VEGFA
GDF15
TUBE1
ARRDC3
CEBPG
SNORA16A
RGS4
BLOC1S5-TXNDC5
LOC390705
EIF2S1
KIM-1
IL6
CXCL2
RELA
HSD17B11
AGPAT3
SETD1B
FTL
MAFG
IL33
FTH1
SLC40A1
GPX4
HAMP
HSPB1

NFE2L2
STEAP3
DRD5
DRD4
MAP3K5
SLC2A1
SLC2A3
SLC2A6
SLC2A8
SLC2A12
GLUT13
SLC2A14
EIF2AK4
TFAP2C
SP1
HBA1
NNMT
PLIN4
HIC1
STMN1
RRM2
CAPG
HNF4A
NGB
YWHAE
GABPB1
AURKA
MIR4715
RIPK1
PRDX1
MIR30B
AKR1C1
AKR1C2
AKR1C3
RB1
HSF1
GCLC
SQSTM1
NQO1
MUC1
MT1G
CISD1
FANCD2
FTMT
HSPA5
HELLS
SCD
FADS2
SRC
STAT3
PML
MTOR
NFS1
TP63
CDKN1A
MIR137
ENPP2
FH

CISD2
MIR9-1
MIR9-2
MIR9-3
ISCU
ACSL3
OTUB1
CD44
LINC00336
BRD4
PRDX6
MIR17
NF2
ARNTL
JUN
CA9
TMBIM4
PLIN2
MIR212
Fer1HCH
AIFM2
LAMP2
ZFP36
PROM2
CHMP5
CHMP6
CAV1
GCH1
CARS
GCLM
GSS
HMGCR
CRYAB
FDFT1
HSBP1
ACACA
SQLE
LOX
