# Supplementary material for: A Ferroptosis-Related Prognostic Risk Score Model to Predict Clinical Significance and Immunogenic Characteristics in Glioblastoma Multiforme
Source: Oxid Med Cell Longev. 2021 Nov 9;2021:9107857. doi: 10.1155/2021/9107857 (PMC8596022; doi:10.1155/2021/9107857)
Supplement: Supplementary 2 — Table S1: DEGs between GBM and normal brain tissue. Table S2: KEGG pathways enriched in ferroptosis-related genes. Table S3: GO enrichment analysis of molecular function (MF). Table S4: GO enrichment analysis of biological process (BP). Table S5: GO enrichment analysis of cellular component (CC). Table S6: cd-Ferr-Geneset1. Table S7: cd-Ferr-geneset2. Table S8: DEG.Subtype1. Table S9: DEG.Subtype2. Table S10: DEG.Subtype3. Table S11: DEG.Subtype4. Table S12: known ferroptosis genes. Table S13: a multifactor regulatory network of the ferroptosis key hub genes. Table S14: Lasso-logistic regression analysis of prognosis factors. Table S15: FRGPRS model applied for TCGA GBM and GSE4412 GBM dataset. [file 9107857.f2.zip › Table S13.pdf]

Table S13. A multifactor regulatory network of the ferroptosis key hub genes

Source	Target
hsa-miR-429	ACVR2B
hsa-miR-34a-5p	ACVR2B
hsa-miR-101-3p	ACVR2B
hsa-miR-186-5p	ACVR2B
hsa-miR-320b	ACVR2B
hsa-miR-3118	ACVR2B
hsa-miR-9-5p	ACVR2B
hsa-miR-199a-5p	ACVR2B
hsa-miR-488-3p	ACVR2B
hsa-miR-181b-5p	ACVR2B
hsa-miR-181a-5p	ACVR2B
hsa-miR-29c-3p	ACVR2B
hsa-miR-29b-3p	ACVR2B
hsa-miR-194-5p	ACVR2B
hsa-miR-346	ACVR2B
hsa-miR-107	ACVR2B
hsa-miR-202-3p	ACVR2B
hsa-miR-139-5p	ACVR2B
hsa-miR-34c-5p	ACVR2B
hsa-let-7a-5p	ACVR2B
hsa-miR-3167	ACVR2B
hsa-miR-200c-3p	ACVR2B
hsa-miR-613	ACVR2B
hsa-miR-148b-3p	ACVR2B
hsa-miR-26a-5p	ACVR2B
hsa-let-7i-5p	ACVR2B
hsa-miR-320d	ACVR2B
hsa-miR-16-5p	ACVR2B
hsa-miR-15a-5p	ACVR2B
hsa-miR-1297	ACVR2B
hsa-miR-4500	ACVR2B
hsa-miR-342-3p	ACVR2B
hsa-miR-127-3p	ACVR2B
hsa-miR-432-3p	ACVR2B
hsa-miR-370-3p	ACVR2B
hsa-miR-543	ACVR2B
hsa-miR-495-3p	ACVR2B
hsa-miR-300	ACVR2B
hsa-miR-381-3p	ACVR2B
hsa-miR-539-5p	ACVR2B
hsa-miR-544a	ACVR2B
hsa-miR-134-5p	ACVR2B
hsa-miR-485-5p	ACVR2B
hsa-miR-154-5p	ACVR2B
hsa-miR-410-3p	ACVR2B
hsa-miR-203a	ACVR2B
hsa-miR-365a-3p	ACVR2B
hsa-miR-138-5p	ACVR2B
hsa-miR-22-3p	ACVR2B
hsa-miR-132-3p	ACVR2B
hsa-miR-212-3p	ACVR2B
hsa-miR-195-5p	ACVR2B
hsa-miR-497-5p	ACVR2B
hsa-miR-324-5p	ACVR2B
hsa-miR-144-3p	ACVR2B
hsa-miR-152-3p	ACVR2B

hsa-miR-320c	ACVR2B
hsa-miR-1	ACVR2B
hsa-miR-27a-3p	ACVR2B
hsa-miR-23a-3p	ACVR2B
hsa-miR-181c-5p	ACVR2B
hsa-miR-181d-5p	ACVR2B
hsa-let-7e-5p	ACVR2B
hsa-miR-4429	ACVR2B
hsa-miR-4262	ACVR2B
hsa-miR-26b-5p	ACVR2B
hsa-miR-103a-3p	ACVR2B
hsa-miR-124-3p	ACVR2B
hsa-let-7c-5p	ACVR2B
hsa-miR-155-5p	ACVR2B
hsa-let-7b-5p	ACVR2B
hsa-miR-425-5p	ACVR2B
hsa-let-7g-5p	ACVR2B
hsa-miR-15b-5p	ACVR2B
hsa-miR-4458	ACVR2B
hsa-miR-449a	ACVR2B
hsa-miR-449b-5p	ACVR2B
hsa-miR-1271-5p	ACVR2B
hsa-miR-340-5p	ACVR2B
hsa-miR-206	ACVR2B
hsa-miR-4465	ACVR2B
hsa-miR-148a-3p	ACVR2B
hsa-miR-653-5p	ACVR2B
hsa-miR-182-5p	ACVR2B
hsa-miR-96-5p	ACVR2B
hsa-miR-183-5p	ACVR2B
hsa-miR-29a-3p	ACVR2B
hsa-miR-320a	ACVR2B
hsa-miR-599	ACVR2B
hsa-miR-876-5p	ACVR2B
hsa-let-7f-5p	ACVR2B
hsa-let-7d-5p	ACVR2B
hsa-miR-23b-3p	ACVR2B
hsa-miR-27b-3p	ACVR2B
hsa-miR-455-5p	ACVR2B
hsa-miR-199b-5p	ACVR2B
hsa-miR-23c	ACVR2B
hsa-miR-221-3p	ACVR2B
hsa-miR-222-3p	ACVR2B
hsa-miR-98-5p	ACVR2B
hsa-miR-374b-5p	ACVR2B
hsa-miR-374a-5p	ACVR2B
hsa-miR-384	ACVR2B
hsa-miR-503-5p	ACVR2B
hsa-miR-424-5p	ACVR2B
hsa-miR-506-3p	ACVR2B
hsa-miR-192-5p	ACVR2B
hsa-miR-215-5p	ACVR2B
hsa-miR-141-3p	ACVR2B
hsa-miR-335-5p	ACVR2B
hsa-miR-140-3p	ACVR2B
hsa-miR-4310	ACVR2B
hsa-miR-4326	ACVR2B
hsa-miR-3927-3p	ACVR2B

hsa-miR-4451	ACVR2B
hsa-miR-6831-5p	ACVR2B
hsa-miR-7157-5p	ACVR2B
hsa-miR-29b-1-5p	ACVR2B
hsa-miR-134-3p	ACVR2B
hsa-miR-1304-5p	ACVR2B
hsa-miR-7114-5p	ACVR2B
hsa-miR-7854-3p	ACVR2B
hsa-miR-196a-5p	ACVR2B
hsa-miR-196b-5p	ACVR2B
hsa-miR-4761-5p	ACVR2B
hsa-miR-4464	ACVR2B
hsa-miR-4748	ACVR2B
hsa-miR-6867-5p	ACVR2B
hsa-miR-98-3p	ACVR2B
hsa-let-7f-1-3p	ACVR2B
hsa-let-7b-3p	ACVR2B
hsa-let-7a-3p	ACVR2B
hsa-miR-4666a-3p	ACVR2B
hsa-miR-577	ACVR2B
hsa-miR-5582-3p	ACVR2B
hsa-miR-548f-3p	ACVR2B
hsa-miR-548e-3p	ACVR2B
hsa-miR-548az-3p	ACVR2B
hsa-miR-548ar-3p	ACVR2B
hsa-miR-548a-3p	ACVR2B
hsa-miR-203a-3p	ACVR2B
hsa-miR-5087	ACVR2B
hsa-miR-6134	ACVR2B
hsa-miR-576-5p	ACVR2B
hsa-miR-4263	ACVR2B
hsa-miR-4799-5p	ACVR2B
hsa-miR-545-5p	ACVR2B
hsa-miR-4282	ACVR2B
hsa-miR-6516-5p	ACVR2B
hsa-miR-3685	ACVR2B
hsa-miR-5047	ACVR2B
hsa-miR-1301-3p	ACVR2B
hsa-miR-7156-3p	ACVR2B
hsa-miR-6881-3p	ACVR2B
hsa-miR-6780a-3p	ACVR2B
hsa-miR-329-5p	ACVR2B
hsa-miR-5692b	ACVR2B
hsa-miR-5692c	ACVR2B
hsa-miR-944	ACVR2B
hsa-miR-369-3p	ACVR2B
hsa-miR-5571-5p	ACVR2B
hsa-miR-574-5p	ACVR2B
hsa-miR-2054	ACVR2B
hsa-miR-5011-5p	ACVR2B
hsa-miR-1277-5p	ACVR2B
hsa-miR-6749-3p	ACVR2B
hsa-miR-126-3p	ACVR2B
hsa-miR-361-3p	ACVR2B
hsa-miR-770-5p	ACVR2B
hsa-miR-4712-5p	ACVR2B
hsa-miR-6756-3p	ACVR2B
hsa-miR-3127-3p	ACVR2B

hsa-miR-4659b-3p	ACVR2B
hsa-miR-4659a-3p	ACVR2B
hsa-miR-6875-3p	ACVR2B
hsa-miR-190a-3p	ACVR2B
hsa-miR-488-3p	ANXA1
hsa-miR-135b-5p	ANXA1
hsa-miR-107	ANXA1
hsa-miR-708-5p	ANXA1
hsa-miR-613	ANXA1
hsa-miR-196a-5p	ANXA1
hsa-miR-135a-5p	ANXA1
hsa-miR-431-5p	ANXA1
hsa-miR-299-3p	ANXA1
hsa-miR-758-3p	ANXA1
hsa-miR-376c-3p	ANXA1
hsa-miR-410-3p	ANXA1
hsa-miR-422a	ANXA1
hsa-miR-21-5p	ANXA1
hsa-miR-338-3p	ANXA1
hsa-miR-1	ANXA1
hsa-miR-216b-5p	ANXA1
hsa-miR-103a-3p	ANXA1
hsa-miR-296-3p	ANXA1
hsa-miR-191-3p	ANXA1
hsa-miR-28-5p	ANXA1
hsa-miR-378a-3p	ANXA1
hsa-miR-340-5p	ANXA1
hsa-miR-206	ANXA1
hsa-miR-196b-5p	ANXA1
hsa-miR-590-5p	ANXA1
hsa-miR-653-5p	ANXA1
hsa-miR-491-5p	ANXA1
hsa-miR-147a	ANXA1
hsa-miR-221-3p	ANXA1
hsa-miR-222-3p	ANXA1
hsa-miR-374b-5p	ANXA1
hsa-miR-374a-5p	ANXA1
hsa-miR-384	ANXA1
hsa-miR-335-5p	ANXA1
hsa-miR-30a-5p	ANXA1
hsa-miR-26b-5p	ANXA1
hsa-miR-101-3p	ANXA2
hsa-miR-9-5p	ANXA2
hsa-miR-135b-5p	ANXA2
hsa-miR-29c-3p	ANXA2
hsa-miR-29b-3p	ANXA2
hsa-miR-613	ANXA2
hsa-miR-135a-5p	ANXA2
hsa-miR-4306	ANXA2
hsa-miR-337-3p	ANXA2
hsa-miR-432-5p	ANXA2
hsa-miR-376a-3p	ANXA2
hsa-miR-376b-3p	ANXA2
hsa-miR-377-3p	ANXA2
hsa-miR-144-3p	ANXA2
hsa-miR-142-3p	ANXA2
hsa-miR-1	ANXA2
hsa-miR-23a-3p	ANXA2

hsa-miR-155-5p	ANXA2
hsa-miR-185-5p	ANXA2
hsa-miR-425-5p	ANXA2
hsa-miR-206	ANXA2
hsa-miR-4644	ANXA2
hsa-miR-489-3p	ANXA2
hsa-miR-29a-3p	ANXA2
hsa-miR-23b-3p	ANXA2
hsa-miR-23c	ANXA2
hsa-miR-223-3p	ANXA2
hsa-miR-1-3p	ANXA2
hsa-miR-769-3p	ANXA2
hsa-miR-132-3p	ANXA2
hsa-miR-133a-3p	ANXA2
hsa-miR-218-5p	ANXA2
hsa-miR-137	BCL7A
hsa-miR-197-3p	BCL7A
hsa-miR-199a-5p	BCL7A
hsa-miR-488-3p	BCL7A
hsa-miR-29c-3p	BCL7A
hsa-miR-29b-3p	BCL7A
hsa-miR-202-3p	BCL7A
hsa-miR-302e	BCL7A
hsa-miR-129-5p	BCL7A
hsa-let-7a-5p	BCL7A
hsa-miR-3167	BCL7A
hsa-miR-613	BCL7A
hsa-miR-148b-3p	BCL7A
hsa-let-7i-5p	BCL7A
hsa-miR-16-5p	BCL7A
hsa-miR-15a-5p	BCL7A
hsa-miR-4500	BCL7A
hsa-miR-19a-3p	BCL7A
hsa-miR-19b-3p	BCL7A
hsa-miR-208a-3p	BCL7A
hsa-miR-208b-3p	BCL7A
hsa-miR-370-3p	BCL7A
hsa-miR-329-3p	BCL7A
hsa-miR-494-3p	BCL7A
hsa-miR-543	BCL7A
hsa-miR-376c-3p	BCL7A
hsa-miR-539-5p	BCL7A
hsa-miR-382-5p	BCL7A
hsa-miR-203a	BCL7A
hsa-miR-211-5p	BCL7A
hsa-miR-140-5p	BCL7A
hsa-miR-195-5p	BCL7A
hsa-miR-497-5p	BCL7A
hsa-miR-152-3p	BCL7A
hsa-miR-21-5p	BCL7A
hsa-miR-338-3p	BCL7A
hsa-miR-1	BCL7A
hsa-miR-24-3p	BCL7A
hsa-miR-27a-3p	BCL7A
hsa-let-7e-5p	BCL7A
hsa-miR-520e	BCL7A
hsa-miR-520a-3p	BCL7A
hsa-miR-520b	BCL7A

hsa-miR-520c-3p	BCL7A
hsa-miR-520d-3p	BCL7A
hsa-miR-372-3p	BCL7A
hsa-miR-373-3p	BCL7A
hsa-miR-499a-5p	BCL7A
hsa-miR-124-3p	BCL7A
hsa-let-7c-5p	BCL7A
hsa-let-7b-5p	BCL7A
hsa-miR-425-5p	BCL7A
hsa-let-7g-5p	BCL7A
hsa-miR-15b-5p	BCL7A
hsa-miR-302d-3p	BCL7A
hsa-miR-302a-3p	BCL7A
hsa-miR-302c-3p	BCL7A
hsa-miR-302b-3p	BCL7A
hsa-miR-4458	BCL7A
hsa-miR-874-3p	BCL7A
hsa-miR-206	BCL7A
hsa-miR-148a-3p	BCL7A
hsa-miR-590-5p	BCL7A
hsa-miR-29a-3p	BCL7A
hsa-miR-486-5p	BCL7A
hsa-miR-876-5p	BCL7A
hsa-miR-873-5p	BCL7A
hsa-miR-204-5p	BCL7A
hsa-let-7f-5p	BCL7A
hsa-let-7d-5p	BCL7A
hsa-miR-27b-3p	BCL7A
hsa-miR-362-3p	BCL7A
hsa-miR-98-5p	BCL7A
hsa-miR-421	BCL7A
hsa-miR-424-5p	BCL7A
hsa-miR-506-3p	BCL7A
hsa-miR-335-5p	BCL7A
hsa-miR-203a-3p	BCL7A
hsa-miR-507	BCL7A
hsa-miR-557	BCL7A
hsa-miR-4447	BCL7A
hsa-miR-4472	BCL7A
hsa-miR-3976	BCL7A
hsa-miR-6795-5p	BCL7A
hsa-miR-6887-5p	BCL7A
hsa-miR-7109-5p	BCL7A
hsa-miR-1290	BCL7A
hsa-miR-1252-5p	BCL7A
hsa-miR-4533	BCL7A
hsa-miR-4747-5p	BCL7A
hsa-miR-5196-5p	BCL7A
hsa-miR-6828-5p	BCL7A
hsa-miR-6516-5p	BCL7A
hsa-miR-9500	BCL7A
hsa-miR-3125	BCL7A
hsa-miR-3916	BCL7A
hsa-miR-6758-5p	BCL7A
hsa-miR-6856-5p	BCL7A
hsa-miR-6859-5p	BCL7A
hsa-miR-6748-5p	BCL7A
hsa-miR-6803-5p	BCL7A

hsa-miR-6769b-5p	BCL7A
hsa-miR-6769a-5p	BCL7A
hsa-miR-92a-2-5p	BCL7A
hsa-miR-4483	BCL7A
hsa-miR-1293	BCL7A
hsa-miR-6756-5p	BCL7A
hsa-miR-6766-5p	BCL7A
hsa-miR-6745	BCL7A
hsa-miR-363-5p	BCL7A
hsa-miR-6885-5p	BCL7A
hsa-miR-328-5p	BCL7A
hsa-miR-486-3p	BCL7A
hsa-miR-885-3p	BCL7A
hsa-miR-151b	BCL7A
hsa-miR-151a-5p	BCL7A
hsa-miR-611	BCL7A
hsa-miR-3131	BCL7A
hsa-miR-6848-5p	BCL7A
hsa-miR-6846-5p	BCL7A
hsa-miR-4697-5p	BCL7A
hsa-miR-4488	BCL7A
hsa-miR-1237-5p	BCL7A
hsa-miR-1251-3p	BCL7A
hsa-miR-3158-3p	BCL7A
hsa-miR-3680-3p	BCL7A
hsa-miR-450b-5p	BCL7A
hsa-miR-7113-5p	BCL7A
hsa-miR-455-3p	BCL7A
hsa-miR-4446-5p	BCL7A
hsa-miR-5006-3p	BCL7A
hsa-miR-4755-5p	BCL7A
hsa-miR-623	BCL7A
hsa-miR-1273g-5p	BCL7A
hsa-miR-203a-5p	BCL7A
hsa-miR-205-3p	BCL7A
hsa-miR-26b-5p	BLVRB
hsa-miR-200b-3p	CD58
hsa-miR-429	CD58
hsa-miR-135b-5p	CD58
hsa-miR-29b-3p	CD58
hsa-miR-200c-3p	CD58
hsa-miR-148b-3p	CD58
hsa-miR-135a-5p	CD58
hsa-miR-16-5p	CD58
hsa-miR-15a-5p	CD58
hsa-miR-432-5p	CD58
hsa-miR-539-5p	CD58
hsa-miR-382-5p	CD58
hsa-miR-195-5p	CD58
hsa-miR-152-3p	CD58
hsa-miR-216a-5p	CD58
hsa-miR-155-5p	CD58
hsa-miR-15b-5p	CD58
hsa-miR-148a-3p	CD58
hsa-miR-183-5p	CD58
hsa-miR-599	CD58
hsa-miR-374b-5p	CD58
hsa-miR-374a-5p	CD58

hsa-miR-200a-3p	CLIC1
hsa-miR-141-3p	CLIC1
hsa-miR-203a	CLIC1
hsa-miR-193b-3p	CLIC1
hsa-miR-193a-3p	CLIC1
hsa-miR-122-5p	CLIC1
hsa-miR-590-3p	CLIC1
hsa-miR-192-5p	CLIC1
hsa-miR-197-3p	CLIC1
hsa-miR-106b-3p	CLIC1
hsa-miR-183-3p	CLIC1
hsa-miR-221-3p	CLIC1
hsa-miR-92a-3p	CLIC1
hsa-miR-25-3p	CLIC1
hsa-miR-128-3p	DCX
hsa-miR-6766-5p	DCX
hsa-miR-6756-5p	DCX
hsa-miR-608	DCX
hsa-miR-7110-5p	DCX
hsa-miR-4651	DCX
hsa-miR-6842-5p	DCX
hsa-miR-6752-5p	DCX
hsa-miR-8085	DCX
hsa-miR-6731-5p	DCX
hsa-miR-6809-5p	DCX
hsa-miR-3616-3p	DCX
hsa-miR-6133	DCX
hsa-miR-6130	DCX
hsa-miR-6129	DCX
hsa-miR-6127	DCX
hsa-miR-4510	DCX
hsa-miR-4419a	DCX
hsa-miR-6732-5p	DCX
hsa-miR-6760-5p	DCX
hsa-miR-6873-5p	DCX
hsa-miR-1229-5p	DCX
hsa-miR-1225-5p	DCX
hsa-miR-1257	DCX
hsa-miR-555	DCX
hsa-miR-6782-5p	DCX
hsa-miR-4282	DCX
hsa-miR-6079	DCX
hsa-miR-1303	DCX
hsa-miR-6758-3p	DCX
hsa-miR-3120-3p	DCX
hsa-miR-1-3p	EMP3
hsa-miR-26b-5p	EMP3
hsa-miR-181b-5p	HFE
hsa-miR-210-3p	HFE
hsa-miR-302e	HFE
hsa-miR-154-5p	HFE
hsa-miR-365a-3p	HFE
hsa-miR-23a-3p	HFE
hsa-miR-181d-5p	HFE
hsa-miR-520e	HFE
hsa-miR-520a-3p	HFE
hsa-miR-520b	HFE
hsa-miR-520c-3p	HFE

hsa-miR-520d-3p	HFE
hsa-miR-372-3p	HFE
hsa-miR-373-3p	HFE
hsa-miR-153-3p	HFE
hsa-miR-155-5p	HFE
hsa-miR-198	HFE
hsa-miR-302d-3p	HFE
hsa-miR-302a-3p	HFE
hsa-miR-302c-3p	HFE
hsa-miR-302b-3p	HFE
hsa-miR-143-3p	HFE
hsa-miR-206	HFE
hsa-miR-23b-3p	HFE
hsa-miR-221-3p	HFE
hsa-miR-222-3p	HFE
hsa-miR-200b-3p	HFE
hsa-miR-200a-3p	HFE
hsa-miR-200c-3p	HFE
hsa-miR-6848-3p	HFE
hsa-miR-6843-3p	HFE
hsa-miR-6878-3p	HFE
hsa-miR-640	HFE
hsa-miR-378a-5p	HFE
hsa-miR-6821-3p	HFE
hsa-miR-1193	HFE
hsa-miR-6790-3p	HFE
hsa-miR-3653-5p	HFE
hsa-miR-1976	HFE
hsa-miR-2392	HFE
hsa-miR-4789-5p	HFE
hsa-miR-95-5p	HFE
hsa-miR-4801	HFE
hsa-miR-4731-3p	HFE
hsa-miR-4511	HFE
hsa-miR-346	LGALS3
hsa-miR-202-3p	LGALS3
hsa-miR-613	LGALS3
hsa-miR-411-5p	LGALS3
hsa-miR-300	LGALS3
hsa-miR-381-3p	LGALS3
hsa-miR-22-3p	LGALS3
hsa-miR-1	LGALS3
hsa-miR-27a-3p	LGALS3
hsa-miR-128-3p	LGALS3
hsa-miR-143-3p	LGALS3
hsa-miR-340-5p	LGALS3
hsa-miR-206	LGALS3
hsa-miR-590-3p	LGALS3
hsa-miR-335-5p	LGALS3
hsa-miR-873-5p	LGALS3
hsa-miR-27b-3p	LGALS3
hsa-miR-424-3p	LGALS3
hsa-miR-744-5p	LGALS3
hsa-miR-128-3p	PLA2G5
hsa-miR-192-3p	PLA2G5
hsa-miR-2467-3p	PLA2G5
hsa-miR-922	PLA2G5
hsa-miR-6847-5p	PLA2G5

hsa-miR-6859-5p	PLA2G5
hsa-miR-3125	PLA2G5
hsa-miR-3916	PLA2G5
hsa-miR-19a-3p	S100A6
hsa-miR-19b-3p	S100A6
hsa-miR-32-5p	S100A6
hsa-miR-363-3p	S100A6
hsa-miR-186-5p	S100A10
hsa-miR-320b	S100A10
hsa-miR-146b-5p	S100A10
hsa-miR-320d	S100A10
hsa-miR-431-5p	S100A10
hsa-miR-300	S100A10
hsa-miR-381-3p	S100A10
hsa-miR-544a	S100A10
hsa-miR-21-5p	S100A10
hsa-miR-320c	S100A10
hsa-miR-146a-5p	S100A10
hsa-miR-1271-5p	S100A10
hsa-miR-590-5p	S100A10
hsa-miR-590-3p	S100A10
hsa-miR-96-5p	S100A10
hsa-miR-335-5p	S100A10
hsa-miR-320a	S100A10
hsa-miR-31-5p	S100A10
hsa-miR-452-5p	S100A10
hsa-miR-100-5p	S100A10
hsa-miR-874-3p	S100A13
hsa-miR-590-5p	S100A13
hsa-miR-200a-3p	SOX11
hsa-miR-34a-5p	SOX11
hsa-miR-30e-5p	SOX11
hsa-miR-30c-5p	SOX11
hsa-miR-101-3p	SOX11
hsa-miR-186-5p	SOX11
hsa-miR-137	SOX11
hsa-miR-197-3p	SOX11
hsa-miR-320b	SOX11
hsa-miR-92b-3p	SOX11
hsa-miR-9-5p	SOX11
hsa-miR-214-3p	SOX11
hsa-miR-199a-5p	SOX11
hsa-miR-199a-3p	SOX11
hsa-miR-205-5p	SOX11
hsa-miR-194-5p	SOX11
hsa-miR-4295	SOX11
hsa-miR-202-3p	SOX11
hsa-miR-129-5p	SOX11
hsa-miR-34c-5p	SOX11
hsa-miR-125b-5p	SOX11
hsa-let-7a-5p	SOX11
hsa-miR-141-3p	SOX11
hsa-miR-615-3p	SOX11
hsa-miR-148b-3p	SOX11
hsa-miR-320d	SOX11
hsa-miR-15a-5p	SOX11
hsa-miR-20a-5p	SOX11
hsa-miR-92a-3p	SOX11

hsa-miR-493-5p	SOX11
hsa-miR-431-5p	SOX11
hsa-miR-370-3p	SOX11
hsa-miR-411-5p	SOX11
hsa-miR-300	SOX11
hsa-miR-381-3p	SOX11
hsa-miR-539-5p	SOX11
hsa-miR-323b-5p	SOX11
hsa-miR-154-5p	SOX11
hsa-miR-496	SOX11
hsa-miR-409-5p	SOX11
hsa-miR-211-5p	SOX11
hsa-miR-7-5p	SOX11
hsa-miR-328-3p	SOX11
hsa-miR-132-3p	SOX11
hsa-miR-212-3p	SOX11
hsa-miR-497-5p	SOX11
hsa-miR-324-5p	SOX11
hsa-miR-33b-5p	SOX11
hsa-miR-144-3p	SOX11
hsa-miR-193a-3p	SOX11
hsa-miR-152-3p	SOX11
hsa-miR-10a-5p	SOX11
hsa-miR-142-3p	SOX11
hsa-miR-301a-3p	SOX11
hsa-miR-320c	SOX11
hsa-miR-133a-3p	SOX11
hsa-miR-4319	SOX11
hsa-miR-122-5p	SOX11
hsa-miR-27a-3p	SOX11
hsa-miR-23a-3p	SOX11
hsa-miR-150-5p	SOX11
hsa-miR-125a-5p	SOX11
hsa-miR-125a-3p	SOX11
hsa-miR-512-5p	SOX11
hsa-miR-498	SOX11
hsa-miR-523-3p	SOX11
hsa-miR-524-3p	SOX11
hsa-miR-521	SOX11
hsa-miR-518d-3p	SOX11
hsa-miR-217	SOX11
hsa-miR-216b-5p	SOX11
hsa-miR-128-3p	SOX11
hsa-miR-153-3p	SOX11
hsa-miR-149-5p	SOX11
hsa-miR-499a-5p	SOX11
hsa-let-7c-5p	SOX11
hsa-miR-155-5p	SOX11
hsa-miR-130b-3p	SOX11
hsa-miR-33a-5p	SOX11
hsa-let-7b-5p	SOX11
hsa-miR-15b-5p	SOX11
hsa-miR-95-3p	SOX11
hsa-miR-218-5p	SOX11
hsa-miR-367-3p	SOX11
hsa-miR-449a	SOX11
hsa-miR-145-5p	SOX11
hsa-miR-340-5p	SOX11

hsa-miR-133b	SOX11
hsa-miR-30a-5p	SOX11
hsa-miR-148a-3p	SOX11
hsa-miR-196b-5p	SOX11
hsa-miR-590-3p	SOX11
hsa-miR-653-5p	SOX11
hsa-miR-25-3p	SOX11
hsa-miR-106b-5p	SOX11
hsa-miR-490-3p	SOX11
hsa-miR-383-5p	SOX11
hsa-miR-320a	SOX11
hsa-miR-30b-5p	SOX11
hsa-miR-30d-5p	SOX11
hsa-miR-873-5p	SOX11
hsa-miR-204-5p	SOX11
hsa-miR-27b-3p	SOX11
hsa-miR-32-5p	SOX11
hsa-miR-455-5p	SOX11
hsa-miR-199b-5p	SOX11
hsa-miR-23c	SOX11
hsa-miR-221-3p	SOX11
hsa-miR-222-3p	SOX11
hsa-miR-325	SOX11
hsa-miR-448	SOX11
hsa-miR-363-3p	SOX11
hsa-miR-20b-5p	SOX11
hsa-miR-106a-5p	SOX11
hsa-miR-424-5p	SOX11
hsa-miR-513a-5p	SOX11
hsa-miR-452-3p	SOX11
hsa-miR-339-5p	SOX11
hsa-miR-550a-5p	SOX11
hsa-miR-550a-3-5p	SOX11
hsa-miR-1271-3p	SOX11
hsa-miR-1252-3p	SOX11
hsa-miR-3662	SOX11
hsa-miR-4698	SOX11
hsa-miR-4760-5p	SOX11
hsa-miR-8061	SOX11
hsa-miR-7847-3p	SOX11
hsa-miR-6851-5p	SOX11
hsa-miR-3689d	SOX11
hsa-miR-6894-5p	SOX11
hsa-miR-765	SOX11
hsa-miR-7154-3p	SOX11
hsa-miR-766-5p	SOX11
hsa-miR-7854-3p	SOX11
hsa-miR-6754-5p	SOX11
hsa-miR-4441	SOX11
hsa-miR-4270	SOX11
hsa-miR-136-3p	SOX11
hsa-miR-505-3p	SOX11
hsa-miR-4528	SOX11
hsa-miR-3591-3p	SOX11
hsa-miR-21-3p	SOX11
hsa-miR-320e	SOX11
hsa-miR-376a-5p	SOX11
hsa-miR-550b-2-5p	SOX11

hsa-miR-7844-5p	SOX11
hsa-miR-4763-3p	SOX11
hsa-miR-1207-5p	SOX11
hsa-miR-940	SOX11
hsa-miR-6893-5p	SOX11
hsa-miR-6808-5p	SOX11
hsa-miR-215-5p	UPP1
hsa-miR-192-5p	UPP1
hsa-miR-34a-5p	ZNF74
hsa-miR-186-5p	ZNF74
hsa-miR-202-3p	ZNF74
hsa-miR-130a-3p	ZNF74
hsa-miR-34c-5p	ZNF74
hsa-miR-4500	ZNF74
hsa-miR-17-5p	ZNF74
hsa-miR-19a-3p	ZNF74
hsa-miR-20a-5p	ZNF74
hsa-miR-19b-3p	ZNF74
hsa-miR-431-5p	ZNF74
hsa-miR-496	ZNF74
hsa-miR-377-3p	ZNF74
hsa-miR-138-5p	ZNF74
hsa-miR-454-3p	ZNF74
hsa-miR-301a-3p	ZNF74
hsa-miR-21-5p	ZNF74
hsa-miR-150-5p	ZNF74
hsa-miR-519d-3p	ZNF74
hsa-miR-124-3p	ZNF74
hsa-miR-301b	ZNF74
hsa-miR-130b-3p	ZNF74
hsa-miR-449a	ZNF74
hsa-miR-449b-5p	ZNF74
hsa-miR-340-5p	ZNF74
hsa-miR-590-5p	ZNF74
hsa-miR-93-5p	ZNF74
hsa-miR-106b-5p	ZNF74
hsa-miR-875-5p	ZNF74
hsa-miR-491-5p	ZNF74
hsa-miR-31-5p	ZNF74
hsa-miR-876-5p	ZNF74
hsa-miR-221-3p	ZNF74
hsa-miR-222-3p	ZNF74
hsa-miR-374b-5p	ZNF74
hsa-miR-374a-5p	ZNF74
hsa-miR-448	ZNF74
hsa-miR-20b-5p	ZNF74
hsa-miR-106a-5p	ZNF74
hsa-miR-542-3p	ZNF74
hsa-miR-506-3p	ZNF74
hsa-miR-98-5p	ZNF74
hsa-miR-8061	ZNF74
hsa-miR-4760-5p	ZNF74
hsa-miR-1273e	ZNF74
hsa-miR-5004-5p	ZNF74
hsa-miR-611	ZNF74
hsa-miR-3131	ZNF74
hsa-miR-3907	ZNF74
hsa-miR-6126	ZNF74

hsa-miR-4493	ZNF74
hsa-miR-605-3p	ZNF74
hsa-miR-125a-3p	ZNF74
hsa-miR-3142	ZNF74
hsa-miR-1245b-5p	ZNF74
hsa-miR-4762-3p	ZNF74
hsa-miR-4303	ZNF74
hsa-miR-8068	ZNF74
hsa-miR-4524a-3p	ZNF74
hsa-miR-6761-5p	ZNF74
hsa-miR-4329	ZNF74
hsa-miR-4251	ZNF74
hsa-miR-6812-3p	ZNF74
hsa-miR-6823-5p	ZNF74
hsa-miR-4524b-3p	ZNF74
hsa-miR-5698	ZNF74
hsa-miR-6805-3p	ZNF74
hsa-miR-5691	ZNF74
hsa-miR-143-5p	ZNF74
hsa-miR-6879-3p	ZNF74
hsa-miR-4430	ZNF74
hsa-miR-3652	ZNF74
hsa-miR-122-5p	ZNF74
hsa-miR-504-3p	ZNF74
hsa-miR-3135b	ZNF74
hsa-miR-3194-3p	ZNF74
hsa-miR-6499-3p	ZNF74
hsa-miR-215-3p	ZNF74
hsa-miR-6773-3p	ZNF74
hsa-miR-500b-3p	ZNF74
hsa-miR-34a-5p	PIGB
hsa-miR-30c-5p	PIGB
hsa-miR-199a-5p	PIGB
hsa-miR-107	PIGB
hsa-miR-34c-5p	PIGB
hsa-let-7a-5p	PIGB
hsa-miR-16-5p	PIGB
hsa-miR-15a-5p	PIGB
hsa-miR-432-3p	PIGB
hsa-miR-299-3p	PIGB
hsa-miR-195-5p	PIGB
hsa-miR-497-5p	PIGB
hsa-miR-33b-5p	PIGB
hsa-miR-338-3p	PIGB
hsa-miR-122-5p	PIGB
hsa-let-7e-5p	PIGB
hsa-miR-103a-3p	PIGB
hsa-let-7c-5p	PIGB
hsa-miR-33a-5p	PIGB
hsa-let-7b-5p	PIGB
hsa-miR-15b-5p	PIGB
hsa-miR-449a	PIGB
hsa-miR-449b-5p	PIGB
hsa-miR-590-3p	PIGB
hsa-let-7f-5p	PIGB
hsa-miR-199b-5p	PIGB
hsa-miR-98-5p	PIGB
hsa-miR-503-5p	PIGB

hsa-miR-424-5p	PIGB
hsa-miR-375	PIGB
hsa-miR-9-5p	PIGB
hsa-miR-149-5p	PIGB
hsa-miR-335-5p	VAMP5
hsa-miR-9-5p	VAMP5
hsa-miR-130a-3p	CCDC109B
hsa-miR-16-5p	CCDC109B
hsa-miR-15a-5p	CCDC109B
hsa-miR-382-5p	CCDC109B
hsa-miR-195-5p	CCDC109B
hsa-miR-497-5p	CCDC109B
hsa-miR-454-3p	CCDC109B
hsa-miR-15b-5p	CCDC109B
hsa-miR-503-5p	CCDC109B
hsa-miR-424-5p	CCDC109B
hsa-miR-26b-5p	CCDC109B
hsa-miR-324-3p	CCDC109B
hsa-miR-30e-5p	KIAA1549
hsa-miR-30c-5p	KIAA1549
hsa-miR-320b	KIAA1549
hsa-miR-9-5p	KIAA1549
hsa-miR-488-3p	KIAA1549
hsa-miR-135b-5p	KIAA1549
hsa-miR-29c-3p	KIAA1549
hsa-miR-29b-3p	KIAA1549
hsa-miR-107	KIAA1549
hsa-miR-302e	KIAA1549
hsa-miR-148b-3p	KIAA1549
hsa-miR-135a-5p	KIAA1549
hsa-miR-320d	KIAA1549
hsa-miR-1297	KIAA1549
hsa-miR-4500	KIAA1549
hsa-miR-17-5p	KIAA1549
hsa-miR-431-5p	KIAA1549
hsa-miR-432-5p	KIAA1549
hsa-miR-494-3p	KIAA1549
hsa-miR-376c-3p	KIAA1549
hsa-miR-382-5p	KIAA1549
hsa-miR-485-5p	KIAA1549
hsa-miR-496	KIAA1549
hsa-miR-422a	KIAA1549
hsa-miR-132-3p	KIAA1549
hsa-miR-212-3p	KIAA1549
hsa-miR-152-3p	KIAA1549
hsa-miR-21-5p	KIAA1549
hsa-miR-320c	KIAA1549
hsa-miR-133a-3p	KIAA1549
hsa-miR-181d-5p	KIAA1549
hsa-miR-150-5p	KIAA1549
hsa-let-7e-5p	KIAA1549
hsa-miR-520e	KIAA1549
hsa-miR-520a-3p	KIAA1549
hsa-miR-520b	KIAA1549
hsa-miR-520c-3p	KIAA1549
hsa-miR-519d-3p	KIAA1549
hsa-miR-520d-3p	KIAA1549
hsa-miR-372-3p	KIAA1549

hsa-miR-373-3p	KIAA1549
hsa-miR-4429	KIAA1549
hsa-miR-216a-5p	KIAA1549
hsa-miR-216b-5p	KIAA1549
hsa-miR-4782-3p	KIAA1549
hsa-miR-153-3p	KIAA1549
hsa-miR-103a-3p	KIAA1549
hsa-let-7b-5p	KIAA1549
hsa-miR-302d-3p	KIAA1549
hsa-miR-302a-3p	KIAA1549
hsa-miR-302c-3p	KIAA1549
hsa-miR-302b-3p	KIAA1549
hsa-miR-4458	KIAA1549
hsa-miR-1271-5p	KIAA1549
hsa-miR-219a-5p	KIAA1549
hsa-miR-133b	KIAA1549
hsa-miR-30a-5p	KIAA1549
hsa-miR-148a-3p	KIAA1549
hsa-miR-590-5p	KIAA1549
hsa-miR-106b-5p	KIAA1549
hsa-miR-182-5p	KIAA1549
hsa-miR-96-5p	KIAA1549
hsa-miR-183-5p	KIAA1549
hsa-miR-29a-3p	KIAA1549
hsa-miR-320a	KIAA1549
hsa-miR-599	KIAA1549
hsa-miR-30b-5p	KIAA1549
hsa-miR-30d-5p	KIAA1549
hsa-miR-421	KIAA1549
hsa-miR-384	KIAA1549
hsa-miR-448	KIAA1549
hsa-miR-106a-5p	KIAA1549
hsa-miR-130b-3p	KIAA1549
hsa-miR-92a-3p	KIAA1549
hsa-miR-4705	KIAA1549
hsa-miR-218-5p	KIAA1549
hsa-miR-4701-5p	KIAA1549
hsa-miR-129-5p	KIAA1549
hsa-miR-3149	KIAA1549
hsa-miR-500b-3p	KIAA1549
hsa-miR-1266-3p	KIAA1549
hsa-miR-888-3p	KIAA1549
hsa-miR-200a-3p	KIAA1549
hsa-miR-141-3p	KIAA1549
hsa-miR-1306-5p	KIAA1549
hsa-miR-1303	KIAA1549
hsa-miR-3674	KIAA1549
hsa-miR-744-3p	KIAA1549
hsa-miR-4423-5p	KIAA1549
hsa-miR-6501-5p	KIAA1549
hsa-miR-4732-5p	KIAA1549
hsa-miR-6832-5p	KIAA1549
hsa-miR-4781-3p	KIAA1549
hsa-miR-483-5p	KIAA1549
hsa-miR-3180-5p	KIAA1549
hsa-miR-525-5p	KIAA1549
hsa-miR-520a-5p	KIAA1549
hsa-miR-4524a-3p	KIAA1549

hsa-miR-4524b-3p	KIAA1549
hsa-miR-7156-3p	KIAA1549
hsa-miR-6861-3p	KIAA1549
hsa-miR-1184	KIAA1549
hsa-miR-6862-3p	KIAA1549
hsa-miR-6784-3p	KIAA1549
hsa-miR-3620-3p	KIAA1549
hsa-miR-6865-3p	KIAA1549
hsa-miR-4700-3p	KIAA1549
hsa-miR-5089-5p	KIAA1549
hsa-miR-7151-3p	KIAA1549
hsa-miR-5095	KIAA1549
hsa-miR-5589-5p	KIAA1549
hsa-miR-4731-5p	KIAA1549
hsa-miR-6506-5p	KIAA1549
hsa-miR-619-5p	KIAA1549
hsa-miR-186-3p	KIAA1549
hsa-miR-4698	KIAA1549
hsa-miR-6504-3p	KIAA1549
hsa-miR-4438	KIAA1549
hsa-miR-9-5p	RAB34
hsa-miR-4295	RAB34
hsa-miR-302e	RAB34
hsa-miR-130a-3p	RAB34
hsa-miR-148b-3p	RAB34
hsa-let-7i-5p	RAB34
hsa-miR-19a-3p	RAB34
hsa-miR-19b-3p	RAB34
hsa-miR-485-5p	RAB34
hsa-miR-203a	RAB34
hsa-miR-132-3p	RAB34
hsa-miR-212-3p	RAB34
hsa-miR-152-3p	RAB34
hsa-miR-454-3p	RAB34
hsa-miR-301a-3p	RAB34
hsa-miR-133a-3p	RAB34
hsa-let-7e-5p	RAB34
hsa-miR-520e	RAB34
hsa-miR-520a-3p	RAB34
hsa-miR-520b	RAB34
hsa-miR-520c-3p	RAB34
hsa-miR-520d-3p	RAB34
hsa-miR-372-3p	RAB34
hsa-miR-373-3p	RAB34
hsa-miR-128-3p	RAB34
hsa-miR-124-3p	RAB34
hsa-miR-155-5p	RAB34
hsa-miR-301b	RAB34
hsa-miR-130b-3p	RAB34
hsa-miR-302d-3p	RAB34
hsa-miR-302a-3p	RAB34
hsa-miR-302c-3p	RAB34
hsa-miR-302b-3p	RAB34
hsa-miR-1271-5p	RAB34
hsa-miR-133b	RAB34
hsa-miR-148a-3p	RAB34
hsa-miR-590-3p	RAB34
hsa-miR-3666	RAB34

hsa-miR-182-5p	RAB34
hsa-miR-96-5p	RAB34
hsa-miR-506-3p	RAB34
hsa-miR-1-3p	RAB34
hsa-miR-30a-5p	RAB34
hsa-miR-93-3p	RAB34
hsa-let-7c-5p	RAB34
hsa-miR-516b-5p	RAB34
hsa-miR-198	RAB34
hsa-miR-4697-3p	RAB34
hsa-miR-6894-5p	RAB34
hsa-miR-765	RAB34
hsa-miR-7154-3p	RAB34
hsa-miR-766-5p	RAB34
hsa-miR-518c-5p	RAB34
hsa-miR-6837-5p	RAB34
hsa-miR-4685-5p	RAB34
hsa-miR-7113-5p	RAB34
hsa-miR-5590-3p	RAB34
hsa-miR-142-5p	RAB34
hsa-miR-6796-3p	RAB34
hsa-miR-6507-3p	RAB34
hsa-miR-301b-3p	RAB34
hsa-miR-200b-3p	MEX3B
hsa-miR-200a-3p	MEX3B
hsa-miR-429	MEX3B
hsa-miR-30e-5p	MEX3B
hsa-miR-30c-5p	MEX3B
hsa-miR-186-5p	MEX3B
hsa-miR-320b	MEX3B
hsa-miR-190b	MEX3B
hsa-miR-92b-3p	MEX3B
hsa-miR-9-5p	MEX3B
hsa-miR-199a-5p	MEX3B
hsa-miR-181b-5p	MEX3B
hsa-miR-181a-5p	MEX3B
hsa-miR-29c-3p	MEX3B
hsa-miR-29b-3p	MEX3B
hsa-miR-194-5p	MEX3B
hsa-miR-146b-5p	MEX3B
hsa-miR-129-5p	MEX3B
hsa-miR-130a-3p	MEX3B
hsa-miR-139-5p	MEX3B
hsa-miR-200c-3p	MEX3B
hsa-miR-141-3p	MEX3B
hsa-miR-26a-5p	MEX3B
hsa-let-7i-5p	MEX3B
hsa-miR-320d	MEX3B
hsa-miR-1297	MEX3B
hsa-miR-4500	MEX3B
hsa-miR-19a-3p	MEX3B
hsa-miR-19b-3p	MEX3B
hsa-miR-92a-3p	MEX3B
hsa-miR-136-5p	MEX3B
hsa-miR-379-5p	MEX3B
hsa-miR-758-3p	MEX3B
hsa-miR-494-3p	MEX3B
hsa-miR-543	MEX3B

hsa-miR-495-3p	MEX3B
hsa-miR-376c-3p	MEX3B
hsa-miR-300	MEX3B
hsa-miR-381-3p	MEX3B
hsa-miR-539-5p	MEX3B
hsa-miR-154-5p	MEX3B
hsa-miR-496	MEX3B
hsa-miR-410-3p	MEX3B
hsa-miR-203a	MEX3B
hsa-miR-211-5p	MEX3B
hsa-miR-190a-5p	MEX3B
hsa-miR-328-3p	MEX3B
hsa-miR-140-5p	MEX3B
hsa-miR-497-5p	MEX3B
hsa-miR-324-5p	MEX3B
hsa-miR-33b-5p	MEX3B
hsa-miR-454-3p	MEX3B
hsa-miR-301a-3p	MEX3B
hsa-miR-320c	MEX3B
hsa-miR-122-5p	MEX3B
hsa-miR-24-3p	MEX3B
hsa-miR-23a-3p	MEX3B
hsa-miR-181c-5p	MEX3B
hsa-miR-181d-5p	MEX3B
hsa-miR-125a-3p	MEX3B
hsa-miR-517b-3p	MEX3B
hsa-miR-372-3p	MEX3B
hsa-miR-4429	MEX3B
hsa-miR-4262	MEX3B
hsa-miR-216b-5p	MEX3B
hsa-miR-128-3p	MEX3B
hsa-miR-26b-5p	MEX3B
hsa-miR-499a-5p	MEX3B
hsa-miR-155-5p	MEX3B
hsa-miR-301b	MEX3B
hsa-miR-130b-3p	MEX3B
hsa-miR-33a-5p	MEX3B
hsa-let-7g-5p	MEX3B
hsa-miR-15b-5p	MEX3B
hsa-miR-218-5p	MEX3B
hsa-miR-367-3p	MEX3B
hsa-miR-146a-5p	MEX3B
hsa-miR-340-5p	MEX3B
hsa-miR-30a-5p	MEX3B
hsa-miR-4465	MEX3B
hsa-miR-590-3p	MEX3B
hsa-miR-25-3p	MEX3B
hsa-miR-29a-3p	MEX3B
hsa-miR-320a	MEX3B
hsa-miR-599	MEX3B
hsa-miR-875-5p	MEX3B
hsa-miR-30b-5p	MEX3B
hsa-miR-30d-5p	MEX3B
hsa-miR-873-5p	MEX3B
hsa-miR-204-5p	MEX3B
hsa-miR-23b-3p	MEX3B
hsa-miR-27b-3p	MEX3B
hsa-miR-32-5p	MEX3B

hsa-miR-199b-5p	MEX3B
hsa-miR-221-3p	MEX3B
hsa-miR-222-3p	MEX3B
hsa-miR-98-5p	MEX3B
hsa-miR-421	MEX3B
hsa-miR-374b-5p	MEX3B
hsa-miR-374a-5p	MEX3B
hsa-miR-448	MEX3B
hsa-miR-363-3p	MEX3B
hsa-miR-450a-5p	MEX3B
hsa-miR-424-5p	MEX3B
hsa-miR-224-5p	MEX3B
hsa-miR-215-5p	MEX3B
hsa-miR-192-5p	MEX3B
hsa-miR-122-5p	TUBA1C
hsa-miR-498	TUBA1C
hsa-miR-590-3p	TUBA1C
hsa-miR-16-5p	TUBA1C
hsa-miR-940	TUBA1C
hsa-miR-760	TUBA1C
hsa-miR-877-3p	TUBA1C
hsa-miR-296-3p	TUBA1C
hsa-miR-324-5p	TUBA1C
hsa-miR-221-3p	TUBA1C
hsa-miR-92a-3p	TUBA1C
hsa-let-7b-5p	TUBA1C
hsa-miR-1-3p	RCOR2
AGRN	VAMP5
TMEM52	VAMP5
HES3	VAMP5
CPSF3L	VAMP5
SAMD11	VAMP5
SSU72	VAMP5
RER1	VAMP5
THAP3	VAMP5
DFFB	VAMP5
TNFRSF9	VAMP5
ENO1-AS1	TUBA1C
DDX11L1	TUBA1C
FLJ42875	TUBA1C
PLEKHN1	TUBA1C
SAMD11	TUBA1C
TNFRSF18	TUBA1C
GABRD	TUBA1C
FAM138F	TUBA1C
C1orf170	TUBA1C
TP73-AS1	TUBA1C
LINC00115	CD58
AGRN	CD58
SLC35E2	CD58
TMEM240	CD58
MMP23A	CD58
CDK11B	CD58
KLHL21	CD58
RERE	CD58
TMEM52	CD58
FLJ42875	CD58
PLEKHG5	CD58

CPSF3L	CD58
PLEKHN1	CD58
SAMD11	CD58
TPRG1L	CD58
TNFRSF18	CD58
VAMP3	CD58
SSU72	CD58
ATAD3B	CD58
ATAD3C	CD58
CDK11A	CD58
FAM41C	CD58
RER1	CD58
SDF4	CD58
SLC35E2B	CD58
TTLL10	CD58
FAM138A	CD58
FAM138F	CD58
DVL1	CD58
SCNN1D	CD58
TAS1R1	CD58
GABRD	CD58
RPL22	CD58
ERRFI1	CD58
TNFRSF9	CD58
UBE2J2	CD58
GLTPD1	CD58
SKI	CD58
WRAP73	CD58
TP73	CD58
LRRC47	CD58
DFFB	CD58
ESPN	CD58
DNAJC11	CD58
CAMTA1	CD58
OR4F3	CD58
MXRA8	CD58
RNF223	CD58
CHD5	CD58
RNF207	CD58
ARHGEF16	CD58
GNB1	CD58
GPR153	CD58
AGRN	PIGB
LINC00115	PIGB
SLC35E2	PIGB
TMEM240	PIGB
MMP23A	PIGB
CDK11B	PIGB
SAMD11	PIGB
KLHL21	PIGB
WASH7P	PIGB
TMEM52	PIGB
ARHGEF16	PIGB
TAS1R3	PIGB
HES3	PIGB
HES4	PIGB
FLJ42875	PIGB
CPSF3L	PIGB

PLEKHN1	PIGB
KLHL17	PIGB
TPRG1L	PIGB
TNFRSF18	PIGB
C1orf174	PIGB
VAMP3	PIGB
SSU72	PIGB
ATAD3B	PIGB
ATAD3C	PIGB
SDF4	PIGB
DVL1	PIGB
PRKCZ	PIGB
FAM138F	PIGB
FAM138A	PIGB
GABRD	PIGB
RPL22	PIGB
ERRFI1	PIGB
RERE	PIGB
RER1	PIGB
FAM213B	PIGB
TNFRSF9	PIGB
UBE2J2	PIGB
AURKAIP1	PIGB
C1orf170	PIGB
GLTPD1	PIGB
SKI	PIGB
WRAP73	PIGB
TP73	PIGB
LRRC47	PIGB
DFFB	PIGB
TP73-AS1	PIGB
ESPN	PIGB
CAMTA1	PIGB
SLC45A1	PIGB
DNAJC11	PIGB
OR4F3	PIGB
MXRA8	PIGB
RNF223	PIGB
SLC35E2B	PIGB
RNF207	PIGB
OR4F29	PIGB
LINC00115	MEX3B
SLC35E2	MEX3B
TMEM240	MEX3B
MMP23A	MEX3B
CDK11B	MEX3B
RERE	MEX3B
ISG15	MEX3B
NADK	MEX3B
FLJ42875	MEX3B
HES4	MEX3B
CPSF3L	MEX3B
PLEKHN1	MEX3B
SAMD11	MEX3B
KLHL17	MEX3B
TNFRSF18	MEX3B
VAMP3	MEX3B
SSU72	MEX3B

ATAD3B	MEX3B
ATAD3C	MEX3B
CDK11A	MEX3B
FAM41C	MEX3B
GABRD	MEX3B
RER1	MEX3B
SDF4	MEX3B
TTLL10	MEX3B
FAM138F	MEX3B
DVL1	MEX3B
TAS1R1	MEX3B
FAM138A	MEX3B
RPL22	MEX3B
ERRFI1	MEX3B
UBE2J2	MEX3B
C1orf170	MEX3B
SKI	MEX3B
WRAP73	MEX3B
TP73	MEX3B
LRRC47	MEX3B
ESPN	MEX3B
DNAJC11	MEX3B
CAMTA1	MEX3B
DFFB	MEX3B
MXRA8	MEX3B
RNF223	MEX3B
SLC35E2B	MEX3B
RNF207	MEX3B
TNFRSF9	MEX3B
ARHGEF16	MEX3B
TMEM240	S100A6
MMP23A	S100A6
DDX11L1	S100A6
SAMD11	S100A6
KLHL21	S100A6
ARHGEF16	S100A6
TNFRSF25	S100A6
FLJ42875	S100A6
CPSF3L	S100A6
TPRG1L	S100A6
TNFRSF18	S100A6
VAMP3	S100A6
SSU72	S100A6
ATAD3B	S100A6
FAM41C	S100A6
RER1	S100A6
SDF4	S100A6
FAM138F	S100A6
DVL1	S100A6
TTC34	S100A6
GABRD	S100A6
RERE	S100A6
TNFRSF9	S100A6
C1orf159	S100A6
GLTPD1	S100A6
TP73	S100A6
LRRC47	S100A6
DFFB	S100A6

RNF207	S100A6
CAMTA1	S100A6
DNAJC11	S100A6
RNF223	S100A6
CHD5	S100A6
LINC00115	CLIC1
HES3	CLIC1
OR4F16	CLIC1
AGRN	CLIC1
FLJ42875	CLIC1
CPSF3L	CLIC1
PLEKHN1	CLIC1
SAMD11	CLIC1
C1orf170	CLIC1
CPSF3L	RCOR2
SAMD11	RCOR2
LINC00115	EMP3
SLC35E2	EMP3
TMEM240	EMP3
MMP23A	EMP3
KLHL21	EMP3
WASH7P	EMP3
TMEM52	EMP3
ARHGEF16	EMP3
DDX11L1	EMP3
MMP23B	EMP3
FLJ42875	EMP3
CPSF3L	EMP3
SAMD11	EMP3
TNFRSF18	EMP3
C1orf174	EMP3
ERRFI1	EMP3
VAMP3	EMP3
ACAP3	EMP3
ACTRT2	EMP3
ATAD3B	EMP3
ATAD3C	EMP3
CCNL2	EMP3
CDK11A	EMP3
SDF4	EMP3
SLC35E2B	EMP3
TTLL10	EMP3
DVL1	EMP3
UTS2	EMP3
FAM138F	EMP3
FAM138A	EMP3
GABRD	EMP3
RERE	EMP3
RER1	EMP3
UBE2J2	EMP3
GLTPD1	EMP3
WRAP73	EMP3
LRRC47	EMP3
DFFB	EMP3
ESPN	EMP3
DNAJC11	EMP3
SLC45A1	EMP3
MXRA8	EMP3

RNF223	EMP3
CHD5	EMP3
RNF207	EMP3
GPR153	EMP3
SAMD11	BLVRB
KLHL21	BLVRB
TMEM52	BLVRB
NADK	BLVRB
HES3	BLVRB
ACOT7	BLVRB
OR4F16	BLVRB
FLJ42875	BLVRB
DDX11L1	BLVRB
B3GALT6	BLVRB
CPSF3L	BLVRB
PLEKHN1	BLVRB
VAMP3	BLVRB
FAM138A	BLVRB
ACAP3	BLVRB
ACTRT2	BLVRB
ATAD3B	BLVRB
FAM41C	BLVRB
RER1	BLVRB
SDF4	BLVRB
SLC35E2B	BLVRB
SSU72	BLVRB
TTLL10	BLVRB
FAM132A	BLVRB
FAM138F	BLVRB
DVL1	BLVRB
PRDM16	BLVRB
UBE2J2	BLVRB
DNAJC11	BLVRB
MXRA8	BLVRB
RNF223	BLVRB
CHD5	BLVRB
RNF207	BLVRB
ARHGEF16	BLVRB
OR4F29	BLVRB
LINC00115	BLVRB
MMP23A	BLVRB
MEGF6	BLVRB
HES2	BLVRB
AGRN	BLVRB
PLEKHG5	BLVRB
TPRG1L	BLVRB
TNFRSF18	BLVRB
ATAD3C	BLVRB
GABRD	BLVRB
NPHP4	BLVRB
PER3	BLVRB
RPL22	BLVRB
TNFRSF9	BLVRB
DFFB	BLVRB
CAMTA1	BLVRB
OR4F3	BLVRB
C1orf86	BLVRB
LINC00115	UPP1

AGRN	UPP1
SLC35E2	UPP1
TMEM240	UPP1
MMP23A	UPP1
CDK11B	UPP1
KLHL21	UPP1
TMEM52	UPP1
DDX11L1	UPP1
HES4	UPP1
FLJ42875	UPP1
CPSF3L	UPP1
PLEKHN1	UPP1
SAMD11	UPP1
TNFRSF18	UPP1
VAMP3	UPP1
SSU72	UPP1
ATAD3B	UPP1
ATAD3C	UPP1
SDF4	UPP1
UTS2	UPP1
FAM138F	UPP1
DVL1	UPP1
CDK11A	UPP1
FAM138A	UPP1
GABRD	UPP1
ERRFI1	UPP1
RER1	UPP1
PRDM16	UPP1
GLTPD1	UPP1
OR4F3	UPP1
SKI	UPP1
WRAP73	UPP1
TP73	UPP1
LRRC47	UPP1
DFFB	UPP1
TP73-AS1	UPP1
ESPN	UPP1
DNAJC11	UPP1
CAMTA1	UPP1
MXRA8	UPP1
RNF223	UPP1
SLC35E2B	UPP1
RNF207	UPP1
TNFRSF9	UPP1
GPR153	UPP1
UBE2J2	UPP1
LINC00115	KIAA1549
SLC35E2	KIAA1549
TMEM240	KIAA1549
KLHL17	KIAA1549
KLHL21	KIAA1549
WASH7P	KIAA1549
ISG15	KIAA1549
ARHGEF16	KIAA1549
TAS1R3	KIAA1549
HES3	KIAA1549
CPSF3L	KIAA1549
C1orf174	KIAA1549

VAMP3	KIAA1549
SSU72	KIAA1549
ACAP3	KIAA1549
ACTRT2	KIAA1549
ATAD3B	KIAA1549
FAM41C	KIAA1549
GABRD	KIAA1549
RER1	KIAA1549
SDF4	KIAA1549
FAM138F	KIAA1549
TTC34	KIAA1549
FLJ42875	KIAA1549
FAM213B	KIAA1549
LRRC47	KIAA1549
TP73-AS1	KIAA1549
RNF223	KIAA1549
KLHL21	ACVR2B
CPSF3L	ACVR2B
PLEKHN1	ACVR2B
SAMD11	ACVR2B
KLHL17	ACVR2B
C1orf174	ACVR2B
TNFRSF18	ACVR2B
FAM213B	ACVR2B
MMP23A	LGALS3
CDK11B	LGALS3
TMEM52	LGALS3
DDX11L1	LGALS3
TNFRSF25	LGALS3
FLJ42875	LGALS3
CPSF3L	LGALS3
PLEKHN1	LGALS3
SAMD11	LGALS3
TNFRSF18	LGALS3
RER1	LGALS3
THAP3	LGALS3
DNAJC11	LGALS3
LINC00115	PLA2G5
TMEM52	PLA2G5
NADK	PLA2G5
HES3	PLA2G5
FLJ42875	PLA2G5
CPSF3L	PLA2G5
SAMD11	PLA2G5
RER1	PLA2G5
THAP3	PLA2G5
DNAJC11	PLA2G5
UBE2J2	PLA2G5
CPSF3L	RAB34
PLEKHN1	RAB34
SAMD11	RAB34
C1orf233	RAB34
LINC00115	S100A13
SLC35E2	S100A13
TMEM240	S100A13
MMP23A	S100A13
CDK11B	S100A13
KLHL21	S100A13

TAS1R3	S100A13
HES4	S100A13
FLJ42875	S100A13
B3GALT6	S100A13
CPSF3L	S100A13
SAMD11	S100A13
TPRG1L	S100A13
C1orf174	S100A13
TNFRSF18	S100A13
VAMP3	S100A13
SSU72	S100A13
ATAD3B	S100A13
ATAD3C	S100A13
SDF4	S100A13
SLC35E2B	S100A13
TTLL10	S100A13
PER3	S100A13
FAM138F	S100A13
DVL1	S100A13
SCNN1D	S100A13
NOL9	S100A13
FAM138A	S100A13
GABRD	S100A13
RERE	S100A13
RER1	S100A13
FAM213B	S100A13
TNFRSF9	S100A13
AURKAIP1	S100A13
C1orf170	S100A13
GLTPD1	S100A13
SKI	S100A13
WRAP73	S100A13
TP73	S100A13
LRRC47	S100A13
ESPN	S100A13
CAMTA1	S100A13
UBE2J2	S100A13
DNAJC11	S100A13
OR4F3	S100A13
MXRA8	S100A13
RNF223	S100A13
RNF207	S100A13
ARHGEF16	S100A13
NADK	BCL7A
AGRN	BCL7A
LINC00115	BCL7A
ISG15	BCL7A
HES5	BCL7A
AJAP1	BCL7A
CPSF3L	BCL7A
SAMD11	BCL7A
KLHL17	BCL7A
RER1	BCL7A
PRDM16	BCL7A
GLTPD1	BCL7A
DNAJC11	BCL7A
RNF223	BCL7A
CHD5	BCL7A

ARHGEF16	BCL7A
DFFB	BCL7A
HES3	ANXA1
HES2	ANXA1
OR4F16	ANXA1
FLJ42875	ANXA1
CPSF3L	ANXA1
PLEKHN1	ANXA1
C1orf174	ANXA1
TNFRSF18	ANXA1
SSU72	ANXA1
SDF4	ANXA1
RPL22	ANXA1
RERE	ANXA1
RER1	ANXA1
THAP3	ANXA1
ERRFI1	ANXA1
FAM213B	ANXA1
AURKAIP1	ANXA1
LRRC47	ANXA1
DNAJC11	ANXA1
SAMD11	HFE
WASH7P	HFE
CPSF3L	HFE
PLEKHN1	HFE
MXRA8	HFE
RNF223	HFE
SLC35E2B	HFE
ARHGEF16	HFE
NADK	ANXA2
LINC00115	ANXA2
AGRN	ANXA2
MEGF6	ANXA2
WASH7P	ANXA2
TMEM52	ANXA2
TAS1R3	ANXA2
HES3	ANXA2
ACOT7	ANXA2
HES2	ANXA2
OR4F16	ANXA2
FLJ42875	ANXA2
CPSF3L	ANXA2
PLEKHN1	ANXA2
SAMD11	ANXA2
TNFRSF18	ANXA2
SLC35E2B	ANXA2
SSU72	ANXA2
C1orf174	ANXA2
VAMP3	ANXA2
DVL1	ANXA2
RER1	ANXA2
FAM213B	ANXA2
UBE2J2	ANXA2
AURKAIP1	ANXA2
C1orf159	ANXA2
C1orf170	ANXA2
GLTPD1	ANXA2
SKI	ANXA2

WRAP73	ANXA2
TP73	ANXA2
LRRC47	ANXA2
DFFB	ANXA2
TP73-AS1	ANXA2
ESPN	ANXA2
DNAJC11	ANXA2
CAMTA1	ANXA2
RNF223	ANXA2
SLC35E2	SOX11
TMEM240	SOX11
OR4F5	SOX11
PEX10	SOX11
SAMD11	SOX11
KLHL21	SOX11
AGRN	SOX11
FLJ42875	SOX11
CPSF3L	SOX11
PLEKHN1	SOX11
C1orf174	SOX11
UTS2	SOX11
VAMP3	SOX11
PHF13	SOX11
FAM138A	SOX11
FAM138F	SOX11
CDK11A	SOX11
SDF4	SOX11
ICMT	SOX11
GABRD	SOX11
PANK4	SOX11
PLCH2	SOX11
RERE	SOX11
THAP3	SOX11
FAM213B	SOX11
UBE2J2	SOX11
RNF223	SOX11
DFFB	SOX11
ENO1-AS1	S100A10
LINC00115	S100A10
SLC35E2	S100A10
MMP23A	S100A10
CDK11B	S100A10
KLHL21	S100A10
WASH7P	S100A10
TMEM52	S100A10
TAS1R3	S100A10
AGRN	S100A10
HES4	S100A10
FLJ42875	S100A10
CPSF3L	S100A10
SAMD11	S100A10
TNFRSF18	S100A10
SLC35E2B	S100A10
C1orf174	S100A10
CA6	S100A10
VAMP3	S100A10
SSU72	S100A10
ATAD3B	S100A10

ATAD3C	S100A10
GABRD	S100A10
RER1	S100A10
SDF4	S100A10
TTLL10	S100A10
PER3	S100A10
FAM138F	S100A10
CDK11A	S100A10
PUSL1	S100A10
RPL22	S100A10
RERE	S100A10
FAM213B	S100A10
UBE2J2	S100A10
GLTPD1	S100A10
OR4F3	S100A10
SKI	S100A10
WRAP73	S100A10
TP73	S100A10
LRRC47	S100A10
DFFB	S100A10
TP73-AS1	S100A10
ESPN	S100A10
CAMTA1	S100A10
SLC45A1	S100A10
DNAJC11	S100A10
MXRA8	S100A10
RNF223	S100A10
RNF207	S100A10
LINC00115	CCDC109B
SLC35E2	CCDC109B
MRPL20	CCDC109B
TMEM240	CCDC109B
CDK11B	CCDC109B
DDX11L1	CCDC109B
SAMD11	CCDC109B
KLHL21	CCDC109B
MEGF6	CCDC109B
RERE	CCDC109B
TMEM52	CCDC109B
ARHGEF16	CCDC109B
TAS1R3	CCDC109B
HES3	CCDC109B
FLJ42875	CCDC109B
TNFRSF25	CCDC109B
B3GALT6	CCDC109B
CPSF3L	CCDC109B
TNFRSF18	CCDC109B
VAMP3	CCDC109B
SSU72	CCDC109B
ACAP3	CCDC109B
ACTRT2	CCDC109B
ATAD3B	CCDC109B
ATAD3C	CCDC109B
CDK11A	CCDC109B
FAM41C	CCDC109B
RER1	CCDC109B
SDF4	CCDC109B
FAM138F	CCDC109B

DVL1	CCDC109B
GABRD	CCDC109B
TNFRSF9	CCDC109B
GLTPD1	CCDC109B
WRAP73	CCDC109B
DFFB	CCDC109B
ESPN	CCDC109B
DNAJC11	CCDC109B
UBE2J2	CCDC109B
OR4F3	CCDC109B
C1orf86	CCDC109B
MXRA8	CCDC109B
RNF223	CCDC109B
SLC35E2B	CCDC109B
CHD5	CCDC109B
RNF207	CCDC109B
GNB1	CCDC109B
UTS2	S100A6
ARNT	S100A6
USF1	S100A6
ARNT	SOX11
USF1	SOX11
ATF2	SOX11
GATA1	SOX11
MZF1	SOX11
CEBPB	SOX11
BPTF	ACVR2B
USF1	ACVR2B
MZF1	ACVR2B
NFIC	DCX
SOX9	DCX
IRF7	HFE
IRF1	HFE
PAX5	CLIC1
BACH2	CLIC1
HNF4A	CLIC1
RFX1	CLIC1
HNF1A	CLIC1
NFE2L1	ANXA1
BACH1	ANXA1
BACH2	ANXA1
POU3F1	ANXA1
CEBPB	ANXA1
ZNF238	RCOR2
MZF1	RCOR2
ARNT	RCOR2
USF1	RCOR2
SRF	RCOR2
BPTF	RCOR2
GATA1	TUBA1C
SP1	TUBA1C
TBP	TUBA1C
BACH1	LGALS3
RFX1	LGALS3
HIVEP2	LGALS3
FOXD1	LGALS3
TBP	ANXA2
BACH2	ANXA2

RREB1	MEX3B
GATA1	MEX3B
FOXC1	MEX3B
FOXO1	MEX3B
FOXO4	MEX3B
FOXF2	MEX3B
ATF2	MEX3B
STAT3	RAB34
MZF1	RAB34
ZNF238	RAB34
BACH1	RAB34
BACH2	RAB34
TFAP4	EMP3
FOXC1	EMP3
NR3C1	S100A6
MYC	SOX11
MYC	ACVR2B
NFE2	CLIC1
MAFK	CLIC1
JUN	CLIC1
FOS	CLIC1
FOSL1	CLIC1
JUNB	CLIC1
JUND	CLIC1
TCF3	RCOR2
TAL1	RCOR2
SREBF1	RCOR2
MYC	RCOR2
MAX	RCOR2
E2F4	RCOR2
E2F1	RCOR2
AHR	MEX3B
ARNT	MEX3B
CREB1	MEX3B
MAX	S100A6
CUX1	SOX11
TOPORS	ACVR2B
CUX1	LGALS3
MAX	SOX11
MAX	ACVR2B
SP1	S100A10
MIR196A2	ANXA1
NFE2L2	S100A6
RUNX1	LGALS3
RUNX2	LGALS3
SP1	S100A6
STAT1	S100A10
TPTEP1	RAB34
RP11-798G7.5	LGALS3
RP11-798G7.5	MEX3B
RP11-445H22.3	RAB34
ARL5B-AS1	TUBA1C
SNHG16	MEX3B
MIR4435-1HG	VAMP5
MIR4435-1HG	RAB34
SNHG11	RAB34
C20orf166-AS1	ANXA2
AC004840.9	S100A6

LINC00982	TUBA1C
DLGAP1-AS1	RAB34
RP11-218M22.1	ACVR2B
PCBP1-AS1	MEX3B
LINC00652	ANXA2
RP11-429J17.2	MEX3B
RP11-181G12.2	RAB34
AC092171.2	VAMP5
AC092171.2	ZNF74
AC092171.2	S100A13
LINC00910	RAB34
LINC00943	ANXA1
SPATA41	MEX3B
LINC00176	MEX3B
LINC00176	RAB34
LINC00173	LGALS3
CTBP1-AS2	ACVR2B
LINC00087	S100A6
RP11-351J23.1	S100A6
RP11-296O14.3	PIGB
RP11-296O14.3	S100A10
RP11-296O14.3	S100A6
RP11-296O14.3	RAB34
ATP1A1OS	S100A10
RP11-290F20.1	ZNF74
RP11-290F20.1	LGALS3
AC005042.4	LGALS3
CTD-2126E3.1	MEX3B
FAM201A	RAB34
FAM201A	MEX3B
RP4-610C12.4	LGALS3
RP4-610C12.4	RAB34
LINC00487	ANXA1
CTA-373H7.7	S100A6
CTA-373H7.7	CD58
CTD-2008L17.2	MEX3B
AC016747.3	RAB34
LINC00488	RAB34
AC005562.1	S100A13
AC114730.5	TUBA1C
MCM3AP-AS1	S100A6
LINC00152	RAB34
RP11-66D17.5	RAB34
TINCR	S100A6
TINCR	TUBA1C
RP11-54O7.3	RAB34
FAM95B1	MEX3B
AC002456.2	VAMP5
AC002456.2	S100A13
AC002456.2	S100A6
AC002456.2	RAB34
HCG14	CD58
RP3-369A17.4	ANXA2
RP3-369A17.4	S100A10
RP3-369A17.4	S100A6
RP3-369A17.4	MEX3B
RP4-798A10.4	RAB34
LINC00403	ANXA2

LINC00403	S100A6
LINC00403	TUBA1C
RP11-344B5.2	ACVR2B
RP11-561I11.3	MEX3B
RASAL2-AS1	RAB34
AC099850.1	MEX3B
AC015987.1	RAB34
LINC00853	BLVRB
RP11-134G8.8	S100A13
TSSC1-IT1	MEX3B
XXbac-BPG308K3.5	BLVRB
RP11-390P2.4	RAB34
AL589743.1	RCOR2
RP4-758J18.10	S100A6
AC078942.1	EMP3
RP11-63P12.6	S100A13
JPX	RAB34
RP11-120J1.1	RCOR2
LINC00475	RAB34
RP11-70P17.1	MEX3B
AC064875.2	RAB34
RP11-75C9.1	RCOR2
FGD5-AS1	LGALS3
LEPREL1-AS1	ANXA1
MIAT	CD58
AC073321.4	MEX3B
RP4-758J18.7	RAB34
AC007879.4	MEX3B
SATB2-AS1	BLVRB
SATB2-AS1	S100A10
RP11-57H12.3	RCOR2
RP11-57H12.3	MEX3B
RP11-57H12.3	ZNF74
RP11-50E11.3	TUBA1C
ZNF32-AS1	RAB34
TMEM191A	ACVR2B
TMEM191A	TUBA1C
RP11-776H12.1	RAB34
AC104655.3	ACVR2B
AC104655.3	VAMP5
AC104655.3	LGALS3
RP1-1J6.2	RAB34
FAM66C	BLVRB
AC109826.1	S100A13
RP11-182I10.3	LGALS3
DANCR	MEX3B
DANCR	RAB34
RP11-395B7.4	KIAA1549
RP3-340N1.2	KIAA1549
CTA-85E5.10	LGALS3
CTA-85E5.10	RAB34
AC011290.4	VAMP5
HCG15	KIAA1549
AC053503.11	MEX3B
RP11-169D4.1	RAB34
NCAM1-AS1	RAB34
RP1-28O10.1	VAMP5
RP11-165J3.6	ACVR2B

AC006042.6	MEX3B
FAM66A	RAB34
AC007383.3	RAB34
RP11-552D4.1	S100A13
RP11-222A11.1	RAB34
RP1-317E23.3	RCOR2
RP11-342M1.3	S100A10
RP11-342M1.3	S100A6
RP11-342M1.3	CD58
RP5-1125A11.1	RAB34
PCAT6	VAMP5
PCAT6	RAB34
AC068039.4	MEX3B
RP13-225O21.2	RAB34
RP3-510D11.1	S100A13
RP3-510D11.1	PIGB
RP11-556E13.1	S100A13
RP11-556E13.1	ACVR2B
RP11-157P1.5	RAB34
RP13-216E22.4	ANXA2
AC005550.4	RAB34
AC005550.4	TUBA1C
RP11-439K3.1	MEX3B
CCDC26	RAB34
ANKRD10-IT1	RCOR2
RP11-168O16.1	BLVRB
LINC00242	VAMP5
LINC00242	S100A6
RP4-580N22.2	RAB34
ACAP2-IT1	RAB34
ACAP2-IT1	TUBA1C
CTD-2554C21.3	S100A13
AC105053.3	ACVR2B
AC053503.4	VAMP5
ACVR2B-AS1	S100A13
SOS1-IT1	S100A6
AC018730.3	RAB34
RP11-398K22.12	MEX3B
RP11-398K22.12	S100A6
OGFR-AS1	MEX3B
Z83851.1	RCOR2
ALMS1-IT1	RAB34
CTA-126B4.7	ANXA2
ELOVL2-AS1	RAB34
LINC00856	ANXA1
RP11-420G6.4	BLVRB
RP11-420G6.4	VAMP5
RP11-420G6.4	ACVR2B
U73166.2	TUBA1C
CTC-228N24.1	RAB34
RP11-54O7.1	RAB34
AC074011.2	MEX3B
AC074011.2	S100A13
RP1-149A16.3	RAB34
AC093850.2	ANXA2
RP11-767N6.7	HFE
MORF4L2-AS1	MEX3B
RP11-38L15.3	TUBA1C

ITPR1-AS1	HFE
ITPR1-AS1	VAMP5
ITPR1-AS1	S100A10
ITPR1-AS1	ANXA2
AC007246.3	MEX3B
RP11-245P10.4	LGALS3
RP11-553A21.3	ZNF74
AP003774.6	S100A13
LINC00574	MEX3B
AF064860.7	HFE
LL0XNC01-116E7.2	MEX3B
DLX6-AS1	ZNF74
DLX6-AS1	S100A6
TRAF3IP2-AS1	S100A6
NEBL-AS1	ZNF74
RP11-67C2.2	MEX3B
RP1-85F18.5	RAB34
RP5-1007M22.2	MEX3B
GNG12-AS1	BLVRB
RP11-132A1.4	MEX3B
AC105053.4	RAB34
AC093673.5	MEX3B
AC093673.5	RAB34
KB-318B8.7	PIGB
KB-318B8.7	RAB34
RP11-1114A5.4	BLVRB
LINC00665	ZNF74
RP4-669P10.16	ZNF74
AC019068.2	ANXA2
RP3-510O8.4	S100A13
RP3-510O8.4	ACVR2B
RP3-510O8.4	RAB34
RP11-267N12.3	RAB34
RP11-40F8.2	TUBA1C
RP11-40F8.2	ANXA2
RP11-290F20.2	TUBA1C
RP11-290F20.2	S100A13
CTA-714B7.5	VAMP5
RP11-277L2.3	S100A13
AP000688.29	ANXA2
AC092295.7	RAB34
AC092295.7	VAMP5
AC092295.7	MEX3B
HCG21	RAB34
LINC00460	ACVR2B
RP1-122K4.2	RAB34
RP4-665J23.1	RAB34
MYCNOS	MEX3B
AC131097.3	MEX3B
AP000695.4	RCOR2
RP11-65J3.1	RAB34
Z83851.4	TUBA1C
AC074117.10	MEX3B
AC074117.10	RAB34
RP11-383C5.5	RAB34
RP11-613M10.6	MEX3B
RP11-94I2.4	LGALS3
RP11-94I2.4	TUBA1C

AC007970.1	S100A6
AC012146.7	TUBA1C
AL450992.2	HFE
RP11-262H14.3	MEX3B
AC073283.4	LGALS3
CTD-2666L21.1	KIAA1549
AC005152.2	RAB34
RP5-994D16.3	ACVR2B
RP11-480I12.7	ACVR2B
AC066593.1	KIAA1549
AC066593.1	LGALS3
AC066593.1	MEX3B
AC012074.2	CD58
CTB-114C7.3	CD58
LINC00601	LGALS3
HM13-IT1	MEX3B
RP11-477D19.2	LGALS3
AC011247.3	MEX3B
AC013402.2	CD58
RBMS3-AS3	MEX3B
ASH1L-AS1	LGALS3
RP11-733O18.1	ACVR2B
RP11-89F3.2	RAB34
RP1-182D15.2	RAB34
AC097662.2	LGALS3
RP11-89N17.4	LGALS3
RP11-162J8.3	ANXA2
RP11-162J8.3	S100A10
RP11-162J8.3	S100A6
AC005592.1	RAB34
BMS1P20	ZNF74
MIR600HG	MEX3B
NDUFA6-AS1	ZNF74
NDUFA6-AS1	RAB34
AC073254.1	RAB34
ZNF503-AS2	MEX3B
CNTFR-AS1	RAB34
RP11-145M4.3	ANXA2
HOXD-AS2	MEX3B
AP001092.4	MEX3B
RP11-312B8.1	TUBA1C
LINC00959	TUBA1C
RP11-76N22.2	RAB34
UNC5B-AS1	MEX3B
DGCR5	RAB34
RP11-443B7.2	BLVRB
RP11-443B7.2	S100A13
RP11-443B7.2	S100A10
RP11-443B7.2	S100A6
AP000251.3	RAB34
FRY-AS1	RAB34
RP11-400K9.4	LGALS3
AC002454.1	RAB34
AC093642.3	MEX3B
RP11-385J1.2	RAB34
RP11-338C15.3	ZNF74
RP11-338C15.3	MEX3B
AP001046.5	CD58

AC138035.2	MEX3B
RP11-432J22.2	VAMP5
AC105344.2	RAB34
RP11-262H14.1	MEX3B
AC129929.5	TUBA1C
AC129929.5	RAB34
PRKAG2-AS1	TUBA1C
RP11-71N10.1	RAB34
RP11-71N10.1	TUBA1C
RP11-71N10.1	MEX3B
RP11-499P20.2	LGALS3
RP4-584D14.5	CD58
RP11-430C7.4	ANXA1
BX255923.3	LGALS3
RP11-38P22.2	MEX3B
RP11-242C19.2	RCOR2
RP1-149A16.17	MEX3B
RP1-149A16.17	RAB34
SNHG3	BLVRB
RP11-18H7.1	MEX3B
SOX2-OT	ANXA2
RP11-285F7.2	TUBA1C
MIR1302-11	RAB34
RP11-649A16.1	RCOR2
LINC00883	MEX3B
RN7SL832P	RAB34
ATP1B3-AS1	S100A6
ATP1B3-AS1	HFE
ATP1B3-AS1	RAB34
LINC00698	RAB34
RP11-1398P2.1	PIGB
RP11-1398P2.1	ZNF74
CTD-2013N24.2	LGALS3
LIFR-AS1	VAMP5
USP2-AS1	HFE
RP11-15B17.1	ZNF74
RP11-540A21.2	MEX3B
DDX11-AS1	MEX3B
GS1-24F4.2	RAB34
RP11-796E2.4	LGALS3
SNHG6	MEX3B
CTC-228N24.3	HFE
RP11-115C21.2	S100A13
SBF2-AS1	MEX3B
SBF2-AS1	ZNF74
LINC00968	PIGB
LINC00968	RAB34
RP11-57A19.2	MEX3B
RP11-1149O23.3	S100A13
LINC00535	RAB34
STARD4-AS1	ANXA2
SOCS2-AS1	RAB34
RP11-588G21.2	RAB34
SPTY2D1-AS1	CD58
CTD-2366F13.1	HFE
DYNLL1-AS1	VAMP5
DYNLL1-AS1	RAB34
RP11-597D13.9	MEX3B

RP11-517B11.4	RCOR2
LINC01085	VAMP5
RP11-666A20.4	S100A13
RP11-362F19.1	ACVR2B
RP11-227H4.5	S100A13
USP46-AS1	RAB34
CTD-2135J3.3	S100A6
CTD-2135J3.3	MEX3B
AC010226.4	RAB34
RP11-21I10.2	MEX3B
CTD-2249K22.1	RCOR2
RP11-98D18.3	RAB34
RP11-98D18.3	ACVR2B
CTC-329D1.2	ZNF74
RP11-60A8.1	VAMP5
RP11-60A8.1	ANXA2
RP11-60A8.1	S100A10
RP11-63E5.6	VAMP5
RP11-122C5.1	TUBA1C
RP11-359B12.2	TUBA1C
CTC-321K16.1	RAB34
CTD-2154I11.2	RAB34
RP11-1E3.1	RAB34
RP11-1E3.1	TUBA1C
RP11-159K7.2	ANXA1
RP11-714G18.1	ACVR2B
RP11-92A5.2	HFE
RP11-517I3.1	TUBA1C
RP11-597D13.8	S100A13
RP11-46H11.3	RAB34
GMDS-AS1	MEX3B
RP11-1079K10.2	LGALS3
AE000661.37	RAB34
RP11-582J16.4	S100A10
RP11-540O11.1	S100A13
RP11-540O11.1	RAB34
CTD-2263F21.1	VAMP5
CTD-2263F21.1	S100A13
AC004069.2	LGALS3
RP11-792D21.2	BLVRB
Y_RNA	ACVR2B
CTB-118P15.2	ZNF74
CTB-118P15.2	TUBA1C
TUG1	RAB34
RP11-257P3.3	S100A13
RP11-150O12.6	S100A10
RP11-317J10.2	ANXA1
KB-1732A1.1	RAB34
RP11-567J20.2	HFE
RP11-567J20.2	TUBA1C
RP11-893F2.13	TUBA1C
KB-1047C11.2	HFE
RP11-10N23.2	MEX3B
AC226119.5	MEX3B
CTC-756D1.3	S100A13
RP11-281O15.4	RAB34
RP11-400K9.3	S100A10
KB-1507C5.4	TUBA1C

RP11-6I2.3	BLVRB
RP11-6I2.3	ZNF74
RP11-473O4.5	TUBA1C
RP11-760H22.2	RAB34
RP11-867G23.10	RAB34
RP4-791M13.3	RAB34
WAC-AS1	TUBA1C
WAC-AS1	RAB34
WAC-AS1	BLVRB
CTD-2523D13.2	S100A10
CTC-378H22.1	S100A13
RP11-326C3.2	KIAA1549
RP13-317D12.3	VAMP5
RP13-317D12.3	S100A10
RP13-317D12.3	S100A6
AF131215.2	MEX3B
RP11-1134I14.8	TUBA1C
RP11-1134I14.8	ANXA2
RP11-1134I14.8	S100A10
RP11-1134I14.8	S100A6
RP11-1134I14.8	ACVR2B
RP13-726E6.2	RAB34
RP11-234B24.2	RAB34
FAM85A	TUBA1C
LINC00925	S100A10
LINC00925	ACVR2B
LINC00925	CD58
LINC00925	VAMP5
LINC00925	S100A6
FAM222A-AS1	MEX3B
FAM222A-AS1	RAB34
FAM222A-AS1	LGALS3
RP11-881M11.4	ACVR2B
RP11-667M19.2	S100A13
RP11-705C15.3	LGALS3
RP11-966I7.2	BLVRB
RP11-783K16.13	RAB34
TMPO-AS1	MEX3B
RP11-996F15.2	TUBA1C
RP11-626P14.2	ZNF74
RP11-96H19.1	ACVR2B
RP11-977G19.11	CD58
RP11-977G19.11	S100A13
RP1-197B17.3	RAB34
RP11-571M6.8	ACVR2B
RP11-701H24.3	RAB34
RP11-328J6.1	TUBA1C
RP11-396F22.1	MEX3B
NOVA1-AS1	ZNF74
RP3-462E2.3	S100A13
RP3-462E2.3	LGALS3
RP3-462E2.3	ANXA2
RP11-809C9.2	LGALS3
RP11-298I3.4	RAB34
FAM181A-AS1	VAMP5
FAM181A-AS1	RAB34
CTD-2002H8.2	ANXA2
AC005041.17	RAB34

RP11-259K15.2	RAB34
RP11-300J18.3	TUBA1C
RP11-300J18.3	ZNF74
RP11-300J18.3	S100A13
RP11-300J18.3	ACVR2B
RP11-696D21.2	RAB34
RP11-8L8.2	MEX3B
RP1-261D10.2	MEX3B
RP1-261D10.2	RAB34
ADAM20P1	RAB34
RP11-133K1.6	S100A6
RP11-3D4.2	LGALS3
CTD-2184D3.6	S100A6
RP11-752G15.8	TUBA1C
RP11-752G15.8	ANXA2
RP11-154J22.1	RAB34
RP11-467H10.2	HFE
RP11-293M10.5	ANXA2
CTD-3094K11.1	RAB34
RP11-227D13.1	ZNF74
CASC7	MEX3B
CASC7	RAB34
RP11-158M2.4	ANXA1
RP11-554A11.9	RAB34
AC083843.1	MEX3B
CTA-204B4.2	RAB34
RP11-4O1.2	MEX3B
RP11-7O14.1	HFE
RP6-201G10.2	ANXA2
RP11-146F11.5	TUBA1C
RP1-239B22.5	MEX3B
SSSCA1-AS1	HFE
RP11-708J19.1	VAMP5
RP11-77H9.2	RAB34
RP11-395B7.7	HFE
RP11-395B7.7	RCOR2
RP11-395B7.7	MEX3B
CTD-2526A2.2	MEX3B
RP11-594N15.3	CD58
CASC14	LGALS3
RP4-561L24.3	S100A6
RP11-73K9.2	TUBA1C
RP11-390B4.5	LGALS3
RP11-1299A16.3	S100A13
RP11-482M8.1	S100A6
RP11-566E18.3	RAB34
AC124789.1	TUBA1C
RP11-434B12.1	RAB34
RP11-437L7.1	RAB34
ADPGK-AS1	S100A13
RP11-57H14.4	MEX3B
RP11-731J8.2	S100A6
RP1-228H13.5	RAB34
RP11-65L3.1	BLVRB
RP11-65L3.1	S100A6
RP11-504A18.1	RAB34
RP13-122B23.8	ACVR2B
AC004158.2	VAMP5

RP11-111K18.2	CD58
RP11-111K18.2	TUBA1C
RP11-209D14.2	HFE
RP11-1000B6.3	S100A13
KB-1460A1.5	TUBA1C
RP11-333O1.1	BLVRB
RBFADN	MEX3B
RP11-68I18.10	ACVR2B
RP11-68I18.10	MEX3B
RP11-317P15.5	TUBA1C
CTA-445C9.14	ANXA1
RP3-512B11.3	RAB34
RP4-714D9.5	VAMP5
CTD-3105H18.13	ZNF74
CTD-2568A17.1	VAMP5
CTD-2568A17.1	MEX3B
CTA-14H9.5	KIAA1549
RP11-698N11.4	TUBA1C
RP11-626G11.1	S100A13
RP11-64K12.10	S100A6
RP11-64K12.10	RAB34
RP1-168L15.5	MEX3B
RP11-399O19.9	ZNF74
RP11-981G7.1	S100A6
RP11-15N24.4	VAMP5
RP11-304L19.11	RAB34
RP11-223I10.1	ZNF74
RP1-265C24.8	VAMP5
RP11-483C6.1	LGALS3
RP11-483C6.1	TUBA1C
RP13-638C3.3	S100A13
RP11-473M20.9	RCOR2
RP11-95P2.1	S100A10
LINC00621	KIAA1549
LINC00621	MEX3B
RP11-353N14.2	RCOR2
RP11-156P1.3	S100A13
RP11-53I6.4	VAMP5
CTC-297N7.5	LGALS3
RP11-160O5.1	CD58
RP11-963H4.3	LGALS3
RP11-963H4.3	ANXA2
RP11-963H4.3	RAB34
RP11-649A18.12	BLVRB
RP11-649A18.12	S100A13
RP11-498C9.16	S100A6
RP11-110H1.4	MEX3B
RP11-676J15.1	LGALS3
CTC-297N7.9	LGALS3
RP11-283C24.1	TUBA1C
RP11-403A21.2	VAMP5
RP11-815I9.4	RAB34
AC145343.2	RAB34
RP11-649A18.7	S100A6
RP11-156L14.1	RAB34
RP11-403A21.1	ANXA2
RP11-649A18.5	S100A6
RP1-56K13.5	ANXA1

RP11-838N2.4	S100A13
CTC-548K16.2	TUBA1C
RP11-806H10.4	RAB34
RP11-806H10.4	TUBA1C
RP11-15A1.3	MEX3B
RP11-820I16.1	ANXA1
CTD-2105E13.13	ACVR2B
CTD-2105E13.13	MEX3B
ILF3-AS1	TUBA1C
RNF157-AS1	MEX3B
CTC-510F12.4	RAB34
RP11-15A1.2	MEX3B
RP1-193H18.2	MEX3B
CTD-2132N18.2	KIAA1549
CTD-2132N18.2	S100A6
CTD-2132N18.2	ZNF74
RP11-798G7.7	BLVRB
RP11-798G7.7	MEX3B
RP11-332H18.4	RAB34
RP11-47L3.1	ACVR2B
CTC-260E6.6	S100A13
CTC-260E6.6	BLVRB
RP11-635N19.1	VAMP5
RP11-873E20.1	BLVRB
RP11-13K12.5	S100A6
RP11-13K12.5	MEX3B
RP11-13K12.5	LGALS3
ZNF571-AS1	RAB34
RP11-703I16.1	TUBA1C
RP11-13K12.1	LGALS3
MIR497HG	VAMP5
RP11-686D22.4	RAB34
CTD-2189E23.1	TUBA1C
RP11-686D22.8	RAB34
AC009005.2	MEX3B
RP11-120K24.3	VAMP5
RP11-120K24.3	S100A6
CTD-2291D10.4	LGALS3
CTD-2291D10.4	ACVR2B
CTD-2291D10.4	RAB34
RP11-256I23.3	LGALS3
CTD-2525I3.3	S100A6
AC018766.6	RAB34
CTC-429C10.2	S100A10
CTC-241F20.4	VAMP5
CTD-2587H19.2	ZNF74
CTD-3099C6.11	S100A13
AP001462.6	ANXA2
AP001462.6	S100A10
RP11-15H20.7	ACVR2B
HCCAT3	LGALS3
HCCAT3	S100A6
ZSCAN16-AS1	VAMP5
CTD-3131K8.2	RAB34
AC008982.2	ANXA2
AC008982.2	MEX3B
AC008982.2	RAB34
RP11-13J10.1	RCOR2

AC006115.3	RAB34
CTD-3018O17.3	ZNF74
CTD-3018O17.3	TUBA1C
CTC-490E21.10	ACVR2B
RP11-571M6.18	S100A6
FAM226B	LGALS3
AF131215.9	MEX3B
AF131215.9	TUBA1C
MIR145	MEX3B
RP11-498E2.9	RAB34
RP11-498E2.9	ANXA1
RP3-430N8.10	RAB34
RP11-338N10.3	MEX3B
RP11-282O18.7	RAB34
RP3-430N8.11	RAB34
RP11-218C14.8	MEX3B
RP11-218C14.8	HFE
RP11-540B6.6	TUBA1C
CTD-3092A11.2	RCOR2
CTC-487M23.5	BLVRB
RP6-99M1.2	RCOR2
RP1-257I20.14	RAB34
RP11-130L8.1	LGALS3
RP11-118F19.1	S100A13
CTD-2267D19.6	ACVR2B
RP11-258C19.7	BLVRB
RP11-381K20.5	ACVR2B
SNORD109A	S100A13
RP11-348N5.7	ANXA2
RP11-359E10.1	EMP3
YTHDF3-AS1	S100A6
RP11-10C24.1	LGALS3
RP11-10C24.2	MEX3B
RP11-10C24.2	RAB34
RP11-230C9.2	MEX3B
RP11-326I11.3	RAB34
RP11-259K5.1	ACVR2B
RP11-435O5.4	RAB34
RP11-137H2.6	TUBA1C
CTD-2541M15.3	RAB34
CTD-2541M15.3	TUBA1C
RP11-379F4.8	MEX3B
CTD-2589H19.6	MEX3B
CTD-2235C13.3	TUBA1C
CTD-2235C13.3	S100A6
RP1-93I3.1	RAB34
RP11-242F4.2	LGALS3
RP11-572O6.1	MEX3B
RP11-572O6.1	RAB34
RP11-557L19.1	MEX3B
U91328.20	HFE
CTA-363E19.2	ANXA2
LA16c-380H5.5	TUBA1C
LA16c-380H5.5	VAMP5
RP3-355L5.5	MEX3B
AC005754.8	RAB34
RP4-555D20.4	RAB34
RP4-555D20.4	S100A13

KB-1836B5.4	LGALS3
CASC15	MEX3B
CASC15	LGALS3
RP3-500L14.2	HFE
RP11-157J24.2	RAB34
RP11-157J24.2	ANXA2
RP11-140K17.3	BLVRB
RP11-111M22.4	RAB34
XXbac-BPGBPG55C20.2	ACVR2B
XXbac-BPGBPG55C20.2	LGALS3
AC002044.4	S100A13
RP11-446N19.1	BLVRB
RP11-446N19.1	S100A10
RP11-441F2.5	CD58
RP11-51J9.6	HFE
CTC-487M23.7	TUBA1C
CTD-2081C10.7	S100A6
CTD-2081C10.7	BLVRB
RP11-38L15.8	MEX3B
RP11-182L21.6	S100A13
RP3-395M20.12	MEX3B
RP11-573G6.9	VAMP5
RP11-573G6.9	S100A6
LINC01023	TUBA1C
RP11-396C23.4	RAB34
RP11-365H22.2	MEX3B
RP11-440L14.4	TUBA1C
AC004471.10	TUBA1C
AC004471.10	RAB34
RP11-534C12.1	ACVR2B
RP11-534C12.1	MEX3B
RP11-367N14.3	RAB34
RP11-367N14.3	TUBA1C
RP11-5C23.1	ZNF74
RP11-5C23.1	HFE
RP1-74M1.3	RAB34
RP11-368I23.3	ACVR2B
RP11-368I23.3	KIAA1549
RP11-368I23.3	LGALS3
RP11-332H14.1	HFE
RP11-57G10.8	RAB34
RP11-299H21.1	MEX3B
RP11-440D17.3	MEX3B
RP11-62J1.4	MEX3B
RP11-329B9.5	ACVR2B
RP5-943J3.2	ZNF74
RP11-47A8.5	RAB34
CTA-390C10.10	MEX3B
CTA-390C10.10	RAB34
RP13-616I3.1	S100A10
RP11-196G18.24	MEX3B
KB-1572G7.2	RAB34
RP11-225B17.2	ACVR2B
RP11-508N22.13	RAB34
DGCR9	CD58
DGCR9	VAMP5
DGCR9	S100A6
DGCR9	RAB34

DGCR9	TUBA1C
RP11-67L2.2	TUBA1C
RP11-122G18.8	LGALS3
AP000569.9	KIAA1549
RP11-416N2.4	RAB34
RP5-973M2.2	MEX3B
XXbac-B444P24.14	MEX3B
XXbac-B444P24.14	RAB34
RP11-525A16.4	S100A6
RP11-127B20.2	HFE
RP11-104L21.3	RCOR2
DGCR10	RAB34
RP11-11N7.4	TUBA1C
RP11-1246C19.1	KIAA1549
RP11-1246C19.1	S100A6
CTB-119C2.1	BLVRB
KB-7G2.8	S100A13
KB-7G2.8	VAMP5
CTA-384D8.34	S100A13
CTA-268H5.14	S100A6
CTA-268H5.14	RAB34
RP11-314B1.2	RAB34
RP11-496H1.2	MEX3B
RP11-61L19.2	MEX3B
RP11-61L19.2	S100A13
RP11-61L19.2	RAB34
KB-1440D3.14	MEX3B
RP11-383I23.2	MEX3B
RP11-54O7.18	MEX3B
AC004067.5	LGALS3
RP11-218F10.3	LGALS3
LL09NC01-139C3.1	VAMP5
LL09NC01-139C3.1	ACVR2B
LL09NC01-139C3.1	S100A10
RP11-731C17.2	RAB34
AP000230.1	HFE
RP11-80H18.4	VAMP5
