# Supplementary material for: A Ferroptosis-Related Prognostic Risk Score Model to Predict Clinical Significance and Immunogenic Characteristics in Glioblastoma Multiforme
Source: Oxid Med Cell Longev. 2021 Nov 9;2021:9107857. doi: 10.1155/2021/9107857 (PMC8596022; doi:10.1155/2021/9107857)
Supplement: Supplementary 2 — Table S1: DEGs between GBM and normal brain tissue. Table S2: KEGG pathways enriched in ferroptosis-related genes. Table S3: GO enrichment analysis of molecular function (MF). Table S4: GO enrichment analysis of biological process (BP). Table S5: GO enrichment analysis of cellular component (CC). Table S6: cd-Ferr-Geneset1. Table S7: cd-Ferr-geneset2. Table S8: DEG.Subtype1. Table S9: DEG.Subtype2. Table S10: DEG.Subtype3. Table S11: DEG.Subtype4. Table S12: known ferroptosis genes. Table S13: a multifactor regulatory network of the ferroptosis key hub genes. Table S14: Lasso-logistic regression analysis of prognosis factors. Table S15: FRGPRS model applied for TCGA GBM and GSE4412 GBM dataset. [file 9107857.f2.zip › Table S15.pdf]

Table S15. FRGPRS model appled for TCGA GBM and GSE4412 GBM dataset

TCGA GBM				GSE4412 GBM			
Tumor_Sample_Barcode	risk.score	score.norm	risk	Sample.name	risk.score	score.norm	risk
TCGA-02-0047	66.073	0.504490872	Low risk	GSM99432	76.817	0.464947469	High risk
TCGA-02-0055	68.618	0.628722054	High risk	GSM99434	73.166	0.232473734	Low risk
TCGA-02-2483	62.098	0.310455921	Low risk	GSM99436	77.3	0.495702006	High risk
TCGA-02-2485	62.272	0.318949527	Low risk	GSM99438	78.725	0.58643744	High risk
TCGA-02-2486	72.897	0.837596407	High risk	GSM99440	77.599	0.514740528	High risk
TCGA-06-0125	63.373	0.372693547	Low risk	GSM99442	78.899	0.597516714	High risk
TCGA-06-0129	67.492	0.573757688	High risk	GSM99444	82.217	0.808787011	High risk
TCGA-06-0130	68.618	0.628722054	High risk	GSM99446	78.112	0.547405285	High risk
TCGA-06-0132	71.622	0.775358782	High risk	GSM99448	72.374	0.182043935	Low risk
TCGA-06-0138	66.073	0.504490872	Low risk	GSM99450	71.109	0.101496339	Low risk
TCGA-06-0141	71.617	0.775114712	High risk	GSM99452	77.624	0.516332378	High risk
TCGA-06-0157	67.806	0.589085229	High risk	GSM99454	72.708	0.203311047	Low risk
TCGA-06-0158	66.86	0.542907351	Low risk	GSM99456	75.542	0.383763133	High risk
TCGA-06-0168	69.405	0.667138534	High risk	GSM99458	71.736	0.14141993	Low risk
TCGA-06-0171	76.224	1	High risk	GSM99460	73.654	0.263546641	Low risk
TCGA-06-0174	64.16	0.411110026	Low risk	GSM99462	80.299	0.686660299	High risk
TCGA-06-0178	66.048	0.503270526	Low risk	GSM99464	72.354	0.180770455	Low risk
TCGA-06-0184	70.68	0.729376159	High risk	GSM99466	77.599	0.514740528	High risk
TCGA-06-0187	66.716	0.535878161	Low risk	GSM99468	75.079	0.354282076	Low risk
TCGA-06-0190	71.168	0.753197305	High risk	GSM99470	77.141	0.485577841	High risk
TCGA-06-0210	68.618	0.628722054	High risk	GSM99472	75.871	0.404711875	High risk
TCGA-06-0211	67.961	0.596651372	High risk	GSM99474	74.292	0.304170646	Low risk
TCGA-06-0219	70.023	0.697305477	High risk	GSM99476	72.997	0.22171283	Low risk
TCGA-06-0221	57.969	0.108903642	Low risk	GSM99478	76.981	0.475390003	High risk
TCGA-06-0238	65.43	0.473103583	Low risk	GSM99480	72.379	0.182362305	Low risk
TCGA-06-0644	71.323	0.760763448	High risk	GSM99482	75.253	0.36536135	Low risk
TCGA-06-0645	71.95	0.791369716	High risk	GSM99484	79.686	0.647628144	High risk
TCGA-06-0646	68.618	0.628722054	High risk	GSM99486	77.455	0.505571474	High risk
TCGA-06-0649	66.068	0.504246803	Low risk	GSM99488	79.083	0.609232728	High risk
TCGA-06-0686	63.861	0.396514693	Low risk	GSM99490	78.899	0.597516714	High risk
TCGA-06-0743	66.073	0.504490872	Low risk	GSM99492	77.599	0.514740528	High risk
TCGA-06-0744	62.885	0.348872401	Low risk	GSM99494	82.052	0.798280802	High risk
TCGA-06-0745	69.241	0.659133066	High risk	GSM99496	73.166	0.232473734	Low risk
TCGA-06-0747	68.753	0.63531192	High risk	GSM99498	73.809	0.27341611	Low risk
TCGA-06-0749	68.917	0.643317387	High risk	GSM99500	73.166	0.232473734	Low risk
TCGA-06-0750	71.483	0.76857366	High risk	GSM99502	69.834	0.020312003	Low risk
TCGA-06-0878	70.84	0.737186371	High risk	GSM99504	74.596	0.323527539	Low risk
TCGA-06-0882	71.293	0.759299033	High risk	GSM99506	69.834	0.020312003	Low risk
TCGA-06-1804	62.91	0.350092746	Low risk	GSM99508	71.109	0.101496339	Low risk
TCGA-06-2557	69.405	0.667138534	High risk	GSM99510	71.139	0.103406558	Low risk
TCGA-06-2558	63.368	0.372449478	Low risk	GSM99512	74.929	0.344730977	Low risk
TCGA-06-2559	64.803	0.442497315	Low risk	GSM99514	72.379	0.182362305	Low risk
TCGA-06-2561	68.773	0.636288197	High risk	GSM99516	74.441	0.313658071	Low risk
TCGA-06-2562	68.449	0.620472518	High risk	GSM99518	75.228	0.3637695	Low risk
TCGA-06-2563	67.811	0.589329298	High risk	GSM99520	72.862	0.213116842	Low risk
TCGA-06-2564	69.41	0.667382603	High risk	GSM99522	74.446	0.313976441	Low risk
TCGA-06-2565	64.648	0.434931173	Low risk	GSM99524	77.933	0.536007641	High risk
TCGA-06-2567	67.348	0.566728498	High risk	GSM99526	73.654	0.263546641	Low risk
TCGA-06-2569	55.738	0	Low risk	GSM99528	76.981	0.475390003	High risk
TCGA-06-2570	65.43	0.473103583	Low risk	GSM99530	73.649	0.263228271	Low risk
TCGA-06-5408	66.865	0.54315142	Low risk	GSM99532	71.736	0.14141993	Low risk
TCGA-06-5410	73.375	0.860929415	High risk	GSM99534	78.73	0.58675581	High risk
TCGA-06-5411	68.135	0.605144977	High risk	GSM99536	71.104	0.101177969	Low risk
TCGA-06-5412	70.68	0.729376159	High risk	GSM99538	77.295	0.495383636	High risk
TCGA-06-5413	64.344	0.42009177	Low risk	GSM99540	76.329	0.433874562	High risk
TCGA-06-5414	66.556	0.528067949	Low risk	GSM99542	81.27	0.748487743	High risk
TCGA-06-5415	65.59	0.480913795	Low risk	GSM99544	75.711	0.394524037	High risk
TCGA-06-5416	60.5	0.23245143	Low risk	GSM99546	76.03	0.414836039	High risk
TCGA-06-5417	57.814	0.101337499	Low risk	GSM99548	85.22	1	High risk
TCGA-06-5418	69.41	0.667382603	High risk	GSM99550	76.324	0.433556192	High risk
TCGA-06-5856	67.807	0.589134043	High risk	GSM99552	77.141	0.485577841	High risk
TCGA-06-5858	62.123	0.311676267	Low risk	GSM99554	74.137	0.294301178	Low risk
TCGA-06-5859	66.238	0.512545153	Low risk	GSM99556	79.512	0.63654887	High risk
TCGA-08-0386	63.528	0.38025969	Low risk	GSM99558	77.624	0.516332378	High risk
TCGA-12-0616	65.59	0.480913795	Low risk	GSM99560	77.455	0.505571474	High risk
TCGA-12-0618	61.615	0.286878844	Low risk	GSM99562	73.171	0.232792104	Low risk
TCGA-12-0619	71.323	0.760763448	High risk	GSM99564	77.46	0.505889844	High risk

TCGA-12-0821	61.645	0.288343259	Low risk	GSM99566	70.82	0.083094556	Low risk
TCGA-12-1597	70.815	0.735966026	High risk	GSM99568	75.084	0.354600446	Low risk
TCGA-12-3650	65.435	0.473347652	Low risk	GSM99570	75.716	0.394842407	High risk
TCGA-12-3652	66.387	0.519818413	Low risk	GSM99572	80.787	0.717733206	High risk
TCGA-12-3653	64.817	0.443180709	Low risk	GSM99574	71.109	0.101496339	Low risk
TCGA-12-5295	69.724	0.682710144	High risk	GSM99576	75.711	0.394524037	High risk
TCGA-12-5299	66.561	0.528312018	Low risk	GSM99578	74.62	0.325055715	Low risk
TCGA-14-0781	71.323	0.760763448	High risk	GSM99580	75.706	0.394205667	High risk
TCGA-14-0787	69.236	0.658888997	High risk	GSM99582	72.867	0.213435212	Low risk
TCGA-14-0789	69.405	0.667138534	High risk	GSM99584	72.384	0.182680675	Low risk
TCGA-14-0790	61.615	0.286878844	Low risk	GSM99586	78.386	0.564851958	High risk
TCGA-14-0817	67.343	0.566484428	High risk	GSM99588	78.894	0.597198344	High risk
TCGA-14-0871	58.099	0.115249439	Low risk	GSM99590	79.512	0.63654887	High risk
TCGA-14-1034	66.556	0.528067949	Low risk	GSM99592	75.103	0.355810252	Low risk
TCGA-14-1823	68.778	0.636532266	High risk	GSM99594	69.515	0	Low risk
TCGA-14-1825	60.34	0.224641218	Low risk	GSM99596	74.441	0.313658071	Low risk
TCGA-14-1829	68.135	0.605144977	High risk	GSM99598	73.161	0.232155365	Low risk
TCGA-14-2554	66.073	0.504490872	Low risk	GSM99600	79.517	0.63686724	High risk
TCGA-15-0742	67.483	0.573318364	High risk				
TCGA-15-1444	66.372	0.519086205	Low risk				
TCGA-16-0846	66.073	0.504490872	Low risk				
TCGA-16-1045	71.134	0.751537635	High risk				
TCGA-19-1390	63.672	0.38728888	Low risk				
TCGA-19-1787	67.503	0.57429464	High risk				
TCGA-19-2619	65.291	0.466318461	Low risk				
TCGA-19-2620	67.034	0.551400957	Low risk				
TCGA-19-2624	65.57	0.479937518	Low risk				
TCGA-19-2625	71.782	0.783168993	High risk				
TCGA-19-2629	69.077	0.651127599	High risk				
TCGA-19-4065	66.556	0.528067949	Low risk				
TCGA-19-5960	58.278	0.123987113	Low risk				
TCGA-26-1442	61.152	0.264278044	Low risk				
TCGA-26-5132	68.295	0.612955189	High risk				
TCGA-26-5133	63.832	0.395099092	Low risk				
TCGA-26-5134	64.947	0.449526506	Low risk				
TCGA-26-5135	68.135	0.605144977	High risk				
TCGA-26-5136	64.798	0.442253246	Low risk				
TCGA-26-5139	66.561	0.528312018	Low risk				
TCGA-27-1830	69.864	0.689544079	High risk				
TCGA-27-1831	69.241	0.659133066	High risk				
TCGA-27-1832	69.565	0.674948745	High risk				
TCGA-27-1834	68.29	0.61271112	High risk				
TCGA-27-1835	63.818	0.394415699	Low risk				
TCGA-27-1837	67.348	0.566728498	High risk				
TCGA-27-2519	70.965	0.743288099	High risk				
TCGA-27-2521	60.983	0.256028507	Low risk				
TCGA-27-2523	66.541	0.527335741	Low risk				
TCGA-27-2524	69.724	0.682710144	High risk				
TCGA-27-2526	66.547	0.527628624	Low risk				
TCGA-27-2528	66.696	0.534901884	Low risk				
TCGA-28-1747	66.073	0.504490872	Low risk				
TCGA-28-1753	68.135	0.605144977	High risk				
TCGA-28-2499	68.295	0.612955189	High risk				
TCGA-28-2509	66.073	0.504490872	Low risk				
TCGA-28-2510	66.377	0.519330274	Low risk				
TCGA-28-2513	71.781	0.78312018	High risk				
TCGA-28-2514	63.702	0.388753295	Low risk				
TCGA-28-5204	65.291	0.466318461	Low risk				
TCGA-28-5207	62.915	0.350336815	Low risk				
TCGA-28-5208	64.803	0.442497315	Low risk				
TCGA-28-5209	61.002	0.25695597	Low risk				
TCGA-28-5213	68.773	0.636288197	High risk				
TCGA-28-5215	67.647	0.581323831	High risk				
TCGA-28-5216	66.377	0.519330274	Low risk				
TCGA-28-5218	66.7	0.53509714	Low risk				
TCGA-28-5220	63.523	0.38001562	Low risk				
TCGA-32-1970	61.64	0.28809919	Low risk				
TCGA-32-1980	69.56	0.674704676	High risk				
TCGA-32-1982	65.131	0.45850825	Low risk				
TCGA-32-2615	68.778	0.636532266	High risk				
TCGA-32-2616	66.721	0.53612223	Low risk				
TCGA-32-2632	71.955	0.791613785	High risk				
TCGA-32-2634	68.439	0.61998438	High risk				
TCGA-32-2638	67.348	0.566728498	High risk				
TCGA-32-4213	70.685	0.729620228	High risk				
TCGA-32-5222	67.193	0.559162355	High risk				
TCGA-41-2571	68.753	0.63531192	High risk				
TCGA-41-2572	68.753	0.63531192	High risk				
TCGA-41-3915	68.13	0.604900908	High risk				
TCGA-41-4097	73.983	0.89060822	High risk				
TCGA-41-5651	60.828	0.248462365	Low risk				
TCGA-76-4925	66.851	0.542468027	Low risk				
TCGA-76-4926	64.016	0.404080836	Low risk				
TCGA-76-4927	67.348	0.566728498	High risk				
TCGA-76-4928	68.623	0.628966123	High risk				
TCGA-76-4929	67.961	0.596651372	High risk				
TCGA-76-4931	66.078	0.504734941	Low risk				
TCGA-76-4932	67.353	0.566972567	High risk				
