# Supplementary material for: A Ferroptosis-Related Prognostic Risk Score Model to Predict Clinical Significance and Immunogenic Characteristics in Glioblastoma Multiforme
Source: Oxid Med Cell Longev. 2021 Nov 9;2021:9107857. doi: 10.1155/2021/9107857 (PMC8596022; doi:10.1155/2021/9107857)
Supplement: Supplementary 2 — Table S1: DEGs between GBM and normal brain tissue. Table S2: KEGG pathways enriched in ferroptosis-related genes. Table S3: GO enrichment analysis of molecular function (MF). Table S4: GO enrichment analysis of biological process (BP). Table S5: GO enrichment analysis of cellular component (CC). Table S6: cd-Ferr-Geneset1. Table S7: cd-Ferr-geneset2. Table S8: DEG.Subtype1. Table S9: DEG.Subtype2. Table S10: DEG.Subtype3. Table S11: DEG.Subtype4. Table S12: known ferroptosis genes. Table S13: a multifactor regulatory network of the ferroptosis key hub genes. Table S14: Lasso-logistic regression analysis of prognosis factors. Table S15: FRGPRS model applied for TCGA GBM and GSE4412 GBM dataset. [file 9107857.f2.zip › Table S2.pdf]

Table S2. KEGG pathways enriched in ferroptosis-related genes

ID	Description	GeneRatio
hsa04216	Ferroptosis	14/103
hsa04140	Autophagy - animal	18/103
hsa05167	Kaposi sarcoma-associated herpesvirus infection	19/103
hsa04137	Mitophagy - animal	12/103
hsa05212	Pancreatic cancer	12/103
hsa04933	AGE-RAGE signaling pathway in diabetic complications	13/103
hsa04068	FoxO signaling pathway	14/103
hsa04066	HIF-1 signaling pathway	13/103
hsa05219	Bladder cancer	9/103
hsa01522	Endocrine resistance	12/103
hsa05210	Colorectal cancer	11/103
hsa05161	Hepatitis B	14/103
hsa05230	Central carbon metabolism in cancer	10/103
hsa05418	Fluid shear stress and atherosclerosis	13/103
hsa05223	Non-small cell lung cancer	10/103
hsa05206	MicroRNAs in cancer	18/103
hsa05225	Hepatocellular carcinoma	13/103
hsa05218	Melanoma	9/103
hsa05214	Glioma	9/103
hsa05220	Chronic myeloid leukemia	9/103
hsa04218	Cellular senescence	12/103
hsa05166	Human T-cell leukemia virus 1 infection	14/103
hsa04012	ErbB signaling pathway	9/103
hsa05235	PD-L1 expression and PD-1 checkpoint pathway in cancer	9/103
hsa05022	Pathways of neurodegeneration - multiple diseases	20/103
hsa05205	Proteoglycans in cancer	13/103
hsa05211	Renal cell carcinoma	8/103
hsa05131	Shigellosis	14/103
hsa04917	Prolactin signaling pathway	8/103
hsa05010	Alzheimer disease	17/103
hsa05231	Choline metabolism in cancer	9/103
hsa04150	mTOR signaling pathway	11/103
hsa05163	Human cytomegalovirus infection	13/103
hsa04217	Necroptosis	11/103
hsa04926	Relaxin signaling pathway	10/103
hsa04625	C-type lectin receptor signaling pathway	9/103
hsa01521	EGFR tyrosine kinase inhibitor resistance	8/103
hsa05213	Endometrial cancer	7/103
hsa04210	Apoptosis	10/103
hsa05162	Measles	10/103
hsa04668	TNF signaling pathway	9/103
hsa05017	Spinocerebellar ataxia	10/103
hsa04621	NOD-like receptor signaling pathway	11/103
hsa04722	Neurotrophin signaling pathway	9/103
hsa04664	Fc epsilon RI signaling pathway	7/103
hsa04657	IL-17 signaling pathway	8/103
hsa05160	Hepatitis C	10/103
hsa05215	Prostate cancer	8/103
hsa04380	Osteoclast differentiation	9/103
hsa05142	Chagas disease	8/103
hsa04136	Autophagy - other	5/103
hsa05169	Epstein-Barr virus infection	11/103
hsa04659	Th17 cell differentiation	8/103
hsa05216	Thyroid cancer	5/103
hsa04370	VEGF signaling pathway	6/103
hsa04978	Mineral absorption	6/103
hsa05224	Breast cancer	9/103
hsa05226	Gastric cancer	9/103
hsa04932	Non-alcoholic fatty liver disease	9/103
hsa04213	Longevity regulating pathway - multiple species	6/103
hsa04071	Sphingolipid signaling pathway	8/103
hsa04935	Growth hormone synthesis, secretion and action	8/103
hsa04211	Longevity regulating pathway	7/103
hsa05171	Coronavirus disease - COVID-19	11/103
hsa04912	GnRH signaling pathway	7/103
hsa05203	Viral carcinogenesis	10/103
hsa04930	Type II diabetes mellitus	5/103
hsa01524	Platinum drug resistance	6/103
hsa04115	p53 signaling pathway	6/103
hsa04910	Insulin signaling pathway	8/103
hsa04620	Toll-like receptor signaling pathway	7/103
hsa04660	T cell receptor signaling pathway	7/103
hsa05133	Pertussis	6/103
hsa05140	Leishmaniasis	6/103
hsa04931	Insulin resistance	7/103
hsa05145	Toxoplasmosis	7/103
hsa04726	Serotonergic synapse	7/103
hsa04921	Oxytocin signaling pathway	8/103
hsa04510	Focal adhesion	9/103
hsa00590	Arachidonic acid metabolism	5/103
hsa04010	MAPK signaling pathway	11/103
hsa04141	Protein processing in endoplasmic reticulum	8/103
hsa05221	Acute myeloid leukemia	5/103
hsa04151	PI3K-Akt signaling pathway	12/103
hsa04371	Apelin signaling pathway	7/103
hsa05135	Yersinia infection	7/103
hsa05120	Epithelial cell signaling in Helicobacter pylori infection	5/103
hsa05020	Prion disease	10/103
hsa05165	Human papillomavirus infection	11/103

hsa04662	B cell receptor signaling pathway	5/103
hsa05012	Parkinson disease	9/103
hsa04919	Thyroid hormone signaling pathway	6/103
hsa04630	JAK-STAT signaling pathway	7/103
hsa05170	Human immunodeficiency virus 1 infection	8/103
hsa00480	Glutathione metabolism	4/103
hsa05222	Small cell lung cancer	5/103
hsa05014	Amyotrophic lateral sclerosis	11/103
hsa05323	Rheumatoid arthritis	5/103
hsa04215	Apoptosis - multiple species	3/103
hsa04915	Estrogen signaling pathway	6/103
hsa04929	GnRH secretion	4/103
hsa04914	Progesterone-mediated oocyte maturation	5/103
hsa05321	Inflammatory bowel disease	4/103
hsa04014	Ras signaling pathway	8/103
hsa04920	Adipocytokine signaling pathway	4/103
hsa04145	Phagosome	6/103
hsa05132	Salmonella infection	8/103
hsa03320	PPAR signaling pathway	4/103
hsa04152	AMPK signaling pathway	5/103
hsa04530	Tight junction	6/103
hsa05164	Influenza A	6/103
hsa01210	2-Oxocarboxylic acid metabolism	2/103
hsa05144	Malaria	3/103
hsa04540	Gap junction	4/103
hsa04913	Ovarian steroidogenesis	3/103
hsa05152	Tuberculosis	6/103
hsa04658	Th1 and Th2 cell differentiation	4/103
hsa00220	Arginine biosynthesis	2/103
hsa04613	Neutrophil extracellular trap formation	6/103
hsa04550	Signaling pathways regulating pluripotency of stem cells	5/103
hsa04750	Inflammatory mediator regulation of TRP channels	4/103
hsa05130	Pathogenic Escherichia coli infection	6/103
hsa04072	Phospholipase D signaling pathway	5/103
hsa04730	Long-term depression	3/103
hsa05016	Huntington disease	8/103
hsa00140	Steroid hormone biosynthesis	3/103
hsa00790	Folate biosynthesis	2/103
hsa04934	Cushing syndrome	5/103
hsa04015	Rap1 signaling pathway	6/103
hsa04720	Long-term potentiation	3/103
hsa04725	Cholinergic synapse	4/103
hsa01523	Antifolate resistance	2/103
hsa04520	Adherens junction	3/103
hsa00760	Nicotinate and nicotinamide metabolism	2/103
hsa04110	Cell cycle	4/103
hsa00250	Alanine, aspartate and glutamate metabolism	2/103
hsa04960	Aldosterone-regulated sodium reabsorption	2/103
hsa05143	African trypanosomiasis	2/103
hsa04650	Natural killer cell mediated cytotoxicity	4/103
hsa04062	Chemokine signaling pathway	5/103
hsa04144	Endocytosis	6/103
hsa04727	GABAergic synapse	3/103
hsa04350	TGF-beta signaling pathway	3/103
hsa04723	Retrograde endocannabinoid signaling	4/103
hsa00564	Glycerophospholipid metabolism	3/103
hsa04640	Hematopoietic cell lineage	3/103
hsa00330	Arginine and proline metabolism	2/103
hsa04916	Melanogenesis	3/103
hsa05146	Amoebiasis	3/103
hsa04024	cAMP signaling pathway	5/103
hsa04974	Protein digestion and absorption	3/103
hsa04810	Regulation of actin cytoskeleton	5/103
hsa04064	NF-kappa B signaling pathway	3/103
hsa04310	Wnt signaling pathway	4/103
hsa04928	Parathyroid hormone synthesis, secretion and action	3/103
hsa04923	Regulation of lipolysis in adipocytes	2/103
hsa05134	Legionellosis	2/103
hsa04670	Leukocyte transendothelial migration	3/103
hsa04724	Glutamatergic synapse	3/103
hsa01200	Carbon metabolism	3/103
hsa04623	Cytosolic DNA-sensing pathway	2/103
hsa05217	Basal cell carcinoma	2/103
hsa00360	Phenylalanine metabolism	1/103
hsa00450	Selenocompound metabolism	1/103
hsa00910	Nitrogen metabolism	1/103
hsa04360	Axon guidance	4/103
hsa05031	Amphetamine addiction	2/103
hsa04622	RIG-I-like receptor signaling pathway	2/103
hsa04728	Dopaminergic synapse	3/103
hsa01230	Biosynthesis of amino acids	2/103
hsa04918	Thyroid hormone synthesis	2/103
hsa03060	Protein export	1/103
hsa04964	Proximal tubule bicarbonate reclamation	1/103
hsa05100	Bacterial invasion of epithelial cells	2/103
hsa04120	Ubiquitin mediated proteolysis	3/103
hsa00592	alpha-Linolenic acid metabolism	1/103
hsa05204	Chemical carcinogenesis	2/103
hsa01040	Biosynthesis of unsaturated fatty acids	1/103
hsa04966	Collecting duct acid secretion	1/103
hsa04392	Hippo signaling pathway - multiple species	1/103
hsa05410	Hypertrophic cardiomyopathy	2/103

hsa00020	Citrate cycle (TCA cycle)	1/103
hsa00030	Pentose phosphate pathway	1/103
hsa04710	Circadian rhythm	1/103
hsa04714	Thermogenesis	4/103
hsa04666	Fc gamma R-mediated phagocytosis	2/103
hsa00350	Tyrosine metabolism	1/103
hsa04922	Glucagon signaling pathway	2/103
hsa00860	Porphyrin and chlorophyll metabolism	1/103
hsa05332	Graft-versus-host disease	1/103
hsa05034	Alcoholism	3/103
hsa05202	Transcriptional misregulation in cancer	3/103
hsa04973	Carbohydrate digestion and absorption	1/103
hsa00565	Ether lipid metabolism	1/103
hsa04672	Intestinal immune network for IgA production	1/103
hsa05030	Cocaine addiction	1/103
hsa04611	Platelet activation	2/103
hsa00270	Cysteine and methionine metabolism	1/103
hsa05110	Vibrio cholerae infection	1/103
hsa04114	Oocyte meiosis	2/103
hsa03460	Fanconi anemia pathway	1/103
hsa00240	Pyrimidine metabolism	1/103
hsa01212	Fatty acid metabolism	1/103
hsa04060	Cytokine-cytokine receptor interaction	4/103
hsa05416	Viral myocarditis	1/103
hsa00561	Glycerolipid metabolism	1/103
hsa04390	Hippo signaling pathway	2/103
hsa00562	Inositol phosphate metabolism	1/103
hsa00980	Metabolism of xenobiotics by cytochrome P450	1/103
hsa04612	Antigen processing and presentation	1/103
hsa04721	Synaptic vesicle cycle	1/103
hsa00983	Drug metabolism - other enzymes	1/103
hsa04146	Peroxisome	1/103
hsa04512	ECM-receptor interaction	1/103
hsa04070	Phosphatidylinositol signaling system	1/103
hsa04713	Circadian entrainment	1/103
hsa04061	Viral protein interaction with cytokine and cytokine receptor	1/103
hsa05168	Herpes simplex virus 1 infection	5/103
hsa04142	Lysosome	1/103
hsa00230	Purine metabolism	1/103
hsa04020	Calcium signaling pathway	2/103
hsa00190	Oxidative phosphorylation	1/103
hsa04270	Vascular smooth muscle contraction	1/103
hsa04261	Adrenergic signaling in cardiomyocytes	1/103
hsa01240	Biosynthesis of cofactors	1/103
hsa03010	Ribosome	1/103
hsa04022	cGMP-PKG signaling pathway	1/103
hsa03013	RNA transport	1/103
hsa04080	Neuroactive ligand-receptor interaction	1/103

BgRatio	pvalue	p.adjust	qvalue
41/8087	3.16E-17	7.23E-15	3.36E-15
137/8087	6.93E-14	7.93E-12	3.68E-12
193/8087	2.64E-12	2.02E-10	9.37E-11
68/8087	3.82E-11	2.19E-09	1.02E-09
76/8087	1.50E-10	6.86E-09	3.18E-09
100/8087	3.05E-10	1.16E-08	5.41E-09
131/8087	8.61E-10	2.61E-08	1.21E-08
109/8087	9.13E-10	2.61E-08	1.21E-08
41/8087	1.55E-09	3.94E-08	1.83E-08
98/8087	3.11E-09	7.13E-08	3.31E-08
86/8087	9.21E-09	1.92E-07	8.91E-08
162/8087	1.40E-08	2.67E-07	1.24E-07
70/8087	1.52E-08	2.67E-07	1.24E-07
139/8087	1.86E-08	3.04E-07	1.41E-07
72/8087	2.01E-08	3.07E-07	1.42E-07
310/8087	5.57E-08	7.97E-07	3.70E-07
168/8087	1.78E-07	2.40E-06	1.12E-06
72/8087	2.71E-07	3.45E-06	1.60E-06
75/8087	3.88E-07	4.67E-06	2.17E-06
76/8087	4.35E-07	4.98E-06	2.31E-06
156/8087	5.85E-07	6.38E-06	2.96E-06
219/8087	6.14E-07	6.40E-06	2.97E-06
85/8087	1.14E-06	1.14E-05	5.29E-06
89/8087	1.69E-06	1.55E-05	7.18E-06
475/8087	1.73E-06	1.55E-05	7.18E-06
205/8087	1.76E-06	1.55E-05	7.18E-06
69/8087	2.32E-06	1.97E-05	9.13E-06
246/8087	2.47E-06	2.02E-05	9.39E-06
70/8087	2.59E-06	2.04E-05	9.49E-06
369/8087	3.50E-06	2.67E-05	1.24E-05
98/8087	3.81E-06	2.82E-05	1.31E-05
155/8087	3.94E-06	2.82E-05	1.31E-05
225/8087	4.94E-06	3.31E-05	1.53E-05
159/8087	5.04E-06	3.31E-05	1.53E-05
129/8087	5.05E-06	3.31E-05	1.53E-05
104/8087	6.25E-06	3.98E-05	1.85E-05
79/8087	6.51E-06	4.03E-05	1.87E-05
58/8087	7.82E-06	4.71E-05	2.19E-05
136/8087	8.11E-06	4.76E-05	2.21E-05
139/8087	9.85E-06	5.64E-05	2.62E-05
112/8087	1.15E-05	6.42E-05	2.98E-05
143/8087	1.27E-05	6.90E-05	3.20E-05
181/8087	1.74E-05	9.25E-05	4.29E-05
119/8087	1.88E-05	9.78E-05	4.54E-05
68/8087	2.27E-05	0.000115568	5.37E-05
94/8087	2.37E-05	0.00011818	5.49E-05
157/8087	2.86E-05	0.00013943	6.47E-05
97/8087	2.99E-05	0.00014249	6.62E-05
128/8087	3.37E-05	0.000157419	7.31E-05
102/8087	4.30E-05	0.000196975	9.14E-05
32/8087	4.66E-05	0.000209158	9.71E-05
202/8087	4.81E-05	0.000211877	9.84E-05
107/8087	6.07E-05	0.000262112	0.000121688
37/8087	9.59E-05	0.000393368	0.000182625
59/8087	9.62E-05	0.000393368	0.000182625
59/8087	9.62E-05	0.000393368	0.000182625
147/8087	9.96E-05	0.00039999	0.0001857
149/8087	0.000110475	0.000436184	0.000202503
150/8087	0.000116297	0.000451391	0.000209563
62/8087	0.000127261	0.000471625	0.000218957
119/8087	0.00012873	0.000471625	0.000218957
119/8087	0.00012873	0.000471625	0.000218957
89/8087	0.000129748	0.000471625	0.000218957
232/8087	0.000166752	0.000596661	0.000277007
93/8087	0.000171135	0.000602923	0.000279914
204/8087	0.000256172	0.000888839	0.000412653
46/8087	0.000275311	0.000940987	0.000436864
73/8087	0.000314782	0.00104471	0.000485018
73/8087	0.000314782	0.00104471	0.000485018
137/8087	0.000339852	0.00108885	0.000505511
104/8087	0.000342346	0.00108885	0.000505511
104/8087	0.000342346	0.00108885	0.000505511
76/8087	0.000392015	0.001229745	0.000570923
77/8087	0.000420808	0.001302229	0.000604574
108/8087	0.000431091	0.001316264	0.00061109
112/8087	0.000537353	0.001619129	0.000751699
115/8087	0.000629978	0.001873571	0.000869826
154/8087	0.00074253	0.002179992	0.001012085
201/8087	0.001009817	0.002927191	0.001358981
61/8087	0.00102756	0.002941391	0.001365573
294/8087	0.001235014	0.003491584	0.001621006
171/8087	0.001464139	0.004088876	0.001898306
67/8087	0.001570662	0.004333513	0.002011881
354/8087	0.00172668	0.004689581	0.00217719
137/8087	0.001761153	0.004689581	0.00217719
137/8087	0.001761153	0.004689581	0.00217719
70/8087	0.001910065	0.005027643	0.002334139
273/8087	0.002416604	0.006288663	0.002919582
331/8087	0.003145785	0.008094211	0.003757827

82/8087	0.003819026	0.009717299	0.004511364
249/8087	0.004347256	0.010833422	0.005029536
121/8087	0.004352292	0.010833422	0.005029536
162/8087	0.00450694	0.011097734	0.005152246
212/8087	0.00550894	0.013420716	0.006230716
57/8087	0.005845683	0.014091173	0.006541983
92/8087	0.006229648	0.014860305	0.006899062
364/8087	0.006416786	0.015148906	0.007033048
93/8087	0.006518349	0.015231653	0.007071464
32/8087	0.007612695	0.017609163	0.008175249
138/8087	0.008186645	0.018747418	0.008703697
64/8087	0.00878812	0.019773406	0.009180023
100/8087	0.008807369	0.019773406	0.009180023
65/8087	0.009274518	0.020510703	0.009522321
232/8087	0.009314905	0.020510703	0.009522321
69/8087	0.011394786	0.024851485	0.011537578
152/8087	0.012816567	0.027688621	0.01285475
249/8087	0.01386854	0.029681268	0.013779858
76/8087	0.015810062	0.033523186	0.015563511
120/8087	0.018284494	0.038414212	0.017834224
169/8087	0.020601552	0.042888686	0.019911548
171/8087	0.021690277	0.04474841	0.020774946
19/8087	0.023858774	0.048782672	0.022647896
50/8087	0.025525425	0.051457129	0.023889543
88/8087	0.025616213	0.051457129	0.023889543
51/8087	0.0268758	0.053445337	0.02481259
180/8087	0.027072747	0.053445337	0.02481259
92/8087	0.02954448	0.057826375	0.026846536
22/8087	0.031445615	0.061025812	0.02833191
190/8087	0.034021112	0.065469199	0.030394802
143/8087	0.035514162	0.067772859	0.031464301
98/8087	0.036073528	0.068271387	0.031695748
197/8087	0.039518175	0.074177557	0.034437754
148/8087	0.040250868	0.074938607	0.034791079
60/8087	0.040710129	0.074999727	0.034819455
306/8087	0.040938715	0.074999727	0.034819455
61/8087	0.042431515	0.077117594	0.035802698
26/8087	0.042816445	0.077204456	0.035843025
155/8087	0.04750191	0.084983885	0.039454711
210/8087	0.051162382	0.090823144	0.042165652
67/8087	0.053511332	0.094262269	0.043762304
113/8087	0.055775809	0.097501224	0.045266024
31/8087	0.058814818	0.102034798	0.047370787
71/8087	0.061594537	0.106053751	0.04923663
35/8087	0.072855563	0.124274342	0.057695742
124/8087	0.073262167	0.124274342	0.057695742
37/8087	0.080241373	0.133154162	0.061818296
37/8087	0.080241373	0.133154162	0.061818296
37/8087	0.080241373	0.133154162	0.061818296
131/8087	0.085682121	0.141159753	0.065534981
192/8087	0.097923051	0.160174133	0.074362617
252/8087	0.101909896	0.165513236	0.076841355
89/8087	0.104267354	0.168149465	0.078065254
94/8087	0.117755288	0.188573153	0.087547177
148/8087	0.119753647	0.190441564	0.088414608
98/8087	0.128991218	0.203717165	0.094577953
99/8087	0.13185855	0.206819232	0.096018122
51/8087	0.137311312	0.213001316	0.098888223
101/8087	0.137660239	0.213001316	0.098888223
102/8087	0.140593738	0.215161811	0.099891257
216/8087	0.140935684	0.215161811	0.099891257
103/8087	0.143548435	0.217699283	0.101069306
218/8087	0.144849131	0.218226652	0.101314143
104/8087	0.1465239	0.218327159	0.101360805
160/8087	0.146822631	0.218327159	0.101360805
106/8087	0.152535419	0.225358781	0.104625313
57/8087	0.16389871	0.239062449	0.110987393
57/8087	0.16389871	0.239062449	0.110987393
114/8087	0.177326635	0.255394965	0.118569945
114/8087	0.177326635	0.255394965	0.118569945
118/8087	0.190121846	0.270428196	0.125549289
63/8087	0.191307283	0.270428196	0.125549289
63/8087	0.191307283	0.270428196	0.125549289
17/8087	0.195977696	0.271993287	0.1262759
17/8087	0.195977696	0.271993287	0.1262759
17/8087	0.195977696	0.271993287	0.1262759
182/8087	0.2017906	0.27837378	0.129238114
69/8087	0.219273121	0.300679909	0.139593982
70/8087	0.223971661	0.305294704	0.141736452
132/8087	0.236552397	0.320535496	0.148812159
75/8087	0.247566232	0.331536065	0.153919295
75/8087	0.247566232	0.331536065	0.153919295
23/8087	0.255636018	0.338281285	0.157050838
23/8087	0.255636018	0.338281285	0.157050838
77/8087	0.257034688	0.338281285	0.157050838
140/8087	0.263934615	0.345377296	0.160345239
25/8087	0.274530994	0.357202259	0.16583511
83/8087	0.285458506	0.369322022	0.171461844
27/8087	0.292950878	0.374780732	0.173996111
27/8087	0.292950878	0.374780732	0.173996111
29/8087	0.310907501	0.395543432	0.183635425
90/8087	0.318487446	0.400081404	0.185742229

30/8087	0.319715707	0.400081404	0.185742229
30/8087	0.319715707	0.400081404	0.185742229
31/8087	0.328412404	0.408730655	0.189757739
231/8087	0.339470774	0.420209769	0.195087045
97/8087	0.351165696	0.432349163	0.200722893
36/8087	0.370271561	0.453434158	0.210511836
107/8087	0.396886602	0.483441659	0.224443151
42/8087	0.417104092	0.502720195	0.233393425
42/8087	0.417104092	0.502720195	0.233393425
187/8087	0.427284125	0.512293533	0.237837954
192/8087	0.444201286	0.529802575	0.245966721
47/8087	0.453483631	0.538071251	0.249805545
49/8087	0.467397579	0.545713234	0.25335342
49/8087	0.467397579	0.545713234	0.25335342
49/8087	0.467397579	0.545713234	0.25335342
124/8087	0.471008112	0.545713234	0.25335342
50/8087	0.474222417	0.545713234	0.25335342
50/8087	0.474222417	0.545713234	0.25335342
129/8087	0.491780216	0.563088347	0.261420009
54/8087	0.500666428	0.570411005	0.264819634
57/8087	0.519631058	0.585534962	0.271841099
57/8087	0.519631058	0.585534962	0.271841099
295/8087	0.521611931	0.585534962	0.271841099
60/8087	0.537882143	0.600853711	0.278952998
61/8087	0.543811898	0.60452876	0.280659181
157/8087	0.598263989	0.661847602	0.307270089
73/8087	0.609353904	0.670875212	0.311461257
78/8087	0.63382671	0.687897235	0.31936392
78/8087	0.63382671	0.687897235	0.31936392
78/8087	0.63382671	0.687897235	0.31936392
80/8087	0.643185094	0.694761258	0.322550618
83/8087	0.656780324	0.706115935	0.327822153
88/8087	0.678308186	0.725853152	0.336985375
97/8087	0.7137421	0.756698801	0.351305809
97/8087	0.7137421	0.756698801	0.351305809
100/8087	0.724671902	0.764745924	0.355041776
498/8087	0.76878108	0.807572785	0.374924621
128/8087	0.808687449	0.842836354	0.391296124
130/8087	0.813607391	0.842836354	0.391296124
240/8087	0.815472632	0.842836354	0.391296124
133/8087	0.820753305	0.842836354	0.391296124
133/8087	0.820753305	0.842836354	0.391296124
150/8087	0.856410315	0.875526617	0.406472941
156/8087	0.867237456	0.882221138	0.409580947
158/8087	0.870663656	0.882221138	0.409580947
167/8087	0.88502923	0.892826844	0.414504763
186/8087	0.910371243	0.9143641	0.424503673
341/8087	0.988509261	0.988509261	0.458926386

geneID	Count
ATG7/CYBB/FTH1/FTL/HMOX1/LPCAT3/MAP1LC3A/SAT1/SLC3A2/SLC40A1/STEAP3/TF/TFRC/TP53	14
ATG7/BECN1/DDIT4/EIF2S1/GABARAPL1/HIF1A/HRAS/LAMP2/MAPK3/MAPK8/MAPK9/NRAS/PIK3CA/PRKAA2/SQSTM1/ULK1/ULK2/WIPI1	18
BECN1/CDKN1A/GABARAPL1/HIF1A/HRAS/IL6/JUN/MAPK3/MAPK8/MAPK9/NRAS/PIK3CA/PTGS2/RB1/STAT3/TP53/UBC/VEGFA/ZFP36	19
BECN1/GABARAPL1/HIF1A/HRAS/JUN/MAPK8/MAPK9/NRAS/SQSTM1/TP53/UBC/ULK1	12
CDKN1A/CDKN2A/EGFR/MAPK3/MAPK8/MAPK9/PIK3CA/RB1/STAT3/TGFBR1/TP53/VEGFA	12
CYBB/HRAS/IL6/JUN/MAPK3/MAPK8/MAPK9/NOX4/NRAS/PIK3CA/STAT3/TGFBR1/VEGFA	13
CDKN1A/EGFR/GABARAPL1/HRAS/IL6/MAPK3/MAPK8/MAPK9/NRAS/PIK3CA/PRKAA2/SIRT1/STAT3/TGFBR1	14
CDKN1A/CYBB/EGFR/HIF1A/HMOX1/IL6/MAPK3/PIK3CA/STAT3/TF/TFRC/TLR4/VEGFA	13
CDKN1A/CDKN2A/EGFR/HRAS/MAPK3/NRAS/RB1/TP53/VEGFA	9
CDKN1A/CDKN2A/EGFR/HRAS/JUN/MAPK3/MAPK8/MAPK9/NRAS/PIK3CA/RB1/TP53	12
CDKN1A/EGFR/HRAS/JUN/MAPK3/MAPK8/MAPK9/NRAS/PIK3CA/TGFBR1/TP53	11
CDKN1A/HRAS/IL6/JUN/MAPK3/MAPK8/MAPK9/NRAS/PIK3CA/RB1/STAT3/TGFBR1/TLR4/TP53	14
EGFR/GLS2/HIF1A/HRAS/IDH1/MAPK3/NRAS/PIK3CA/SLC1A5/TP53	10
CAV1/HMOX1/JUN/MAP3K5/MAPK8/MAPK9/NCF2/NFE2L2/PIK3CA/PRKAA2/SQSTM1/TP53/VEGFA	13
CDKN1A/CDKN2A/EGFR/HRAS/MAPK3/NRAS/PIK3CA/RB1/STAT3/TP53	10
CD44/CDKN1A/CDKN2A/DDIT4/EGFR/GLS2/HMOX1/HRAS/MAPK3/NRAS/PIK3CA/PTGS2/SIRT1/SOCS1/STAT3/TP53/TP63/VEGFA	18
CDKN1A/CDKN2A/EGFR/HMOX1/HRAS/MAPK3/NFE2L2/NRAS/PIK3CA/RB1/TGFBR1/TP53/TXNRD1	13
CDKN1A/CDKN2A/EGFR/HRAS/MAPK3/NRAS/PIK3CA/RB1/TP53	9
CDKN1A/CDKN2A/EGFR/HRAS/MAPK3/NRAS/PIK3CA/RB1/TP53	9
CDKN1A/CDKN2A/HRAS/MAPK3/NRAS/PIK3CA/RB1/TGFBR1/TP53	9
CDKN1A/CDKN2A/HRAS/IL6/MAPK3/NRAS/PIK3CA/RB1/SIRT1/SQSTM1/TGFBR1/TP53	12
CDKN1A/CDKN2A/HRAS/IL6/JUN/MAPK3/MAPK8/MAPK9/NRAS/PIK3CA/RB1/TGFBR1/TP53/ZFP36	14
CDKN1A/EGFR/HRAS/JUN/MAPK3/MAPK8/MAPK9/NRAS/PIK3CA	9
EGFR/HIF1A/HRAS/JUN/MAPK3/NRAS/PIK3CA/STAT3/TLR4	9
BECN1/CYBB/EIF2S1/GABARAPL1/HRAS/HSPA5/IL6/MAP3K5/MAPK3/MAPK8/MAPK9/NOX4/NRAS/PTGS2/SQSTM1/UBC/ULK1/ULK2/WIPI1/XBP1	20
CAV1/CD44/CDKN1A/EGFR/HIF1A/HRAS/MAPK3/NRAS/PIK3CA/STAT3/TLR4/TP53/VEGFA	13
CDKN1A/HIF1A/HRAS/JUN/MAPK3/NRAS/PIK3CA/VEGFA	8
BECN1/CD44/EGFR/GABARAPL1/JUN/MAPK3/MAPK8/MAPK9/PIK3CA/SQSTM1/TLR4/TP53/UBC/WIPI1	14
HRAS/MAPK3/MAPK8/MAPK9/NRAS/PIK3CA/SOCS1/STAT3	8
BECN1/CYBB/EIF2S1/HRAS/IL6/MAP3K5/MAPK3/MAPK8/MAPK9/NOX4/NRAS/PIK3CA/PTGS2/ULK1/ULK2/WIPI1/XBP1	17
EGFR/HIF1A/HRAS/JUN/MAPK3/MAPK8/MAPK9/NRAS/PIK3CA	9
ATP6V1G2/DDIT4/HRAS/LPIN1/MAPK3/NRAS/PIK3CA/PRKAA2/SLC3A2/ULK1/ULK2	11
CDKN1A/CDKN2A/EGFR/HRAS/IL6/MAPK3/NRAS/PIK3CA/PTGS2/RB1/STAT3/TP53/VEGFA	13
CHMP6/CYBB/FTH1/FTL/IL33/MAPK8/MAPK9/SQSTM1/STAT3/TLR4/TNFAIP3	11
EGFR/HRAS/JUN/MAPK3/MAPK8/MAPK9/NRAS/PIK3CA/TGFBR1/VEGFA	10
HRAS/IL6/JUN/MAPK3/MAPK8/MAPK9/NRAS/PIK3CA/PTGS2	9
EGFR/HRAS/IL6/MAPK3/NRAS/PIK3CA/STAT3/VEGFA	8
CDKN1A/EGFR/HRAS/MAPK3/NRAS/PIK3CA/TP53	7
EIF2S1/HRAS/JUN/MAP3K5/MAPK3/MAPK8/MAPK9/NRAS/PIK3CA/TP53	10
EIF2S1/IL6/JUN/MAPK8/MAPK9/PIK3CA/STAT3/TLR4/TNFAIP3/TP53	10
IL6/JUN/MAP3K5/MAPK3/MAPK8/MAPK9/PIK3CA/PTGS2/TNFAIP3	9
BECN1/MAP3K5/MAPK8/MAPK9/PIK3CA/ULK1/ULK2/VLDLR/WIPI1/XBP1	10
CYBB/GABARAPL1/IL6/JUN/MAPK3/MAPK8/MAPK9/PANX1/TLR4/TNFAIP3/TXNIP	11
HRAS/JUN/MAP3K5/MAPK3/MAPK8/MAPK9/NRAS/PIK3CA/TP53	9
ALOX5/HRAS/MAPK3/MAPK8/MAPK9/NRAS/PIK3CA	7
ELAVL1/IL6/JUN/MAPK3/MAPK8/MAPK9/PTGS2/TNFAIP3	8
CDKN1A/EGFR/EIF2S1/HRAS/MAPK3/NRAS/PIK3CA/RB1/STAT3/TP53	10
CDKN1A/EGFR/HRAS/MAPK3/NRAS/PIK3CA/RB1/TP53	8
JUN/MAPK3/MAPK8/MAPK9/NCF2/PIK3CA/SOCS1/SQSTM1/TGFBR1	9
IL6/JUN/MAPK3/MAPK8/MAPK9/PIK3CA/TGFBR1/TLR4	8
ATG7/BECN1/GABARAPL1/ULK2/WIPI1	5
CD44/CDKN1A/IL6/JUN/MAPK8/MAPK9/PIK3CA/RB1/STAT3/TNFAIP3/TP53	11
HIF1A/IL6/JUN/MAPK3/MAPK8/MAPK9/STAT3/TGFBR1	8
CDKN1A/HRAS/MAPK3/NRAS/TP53	5
HRAS/MAPK3/NRAS/PIK3CA/PTGS2/VEGFA	6
FTH1/FTL/HMOX1/MT1G/SLC40A1/TF	6
CDKN1A/EGFR/HRAS/JUN/MAPK3/NRAS/PIK3CA/RB1/TP53	9
CDKN1A/EGFR/HRAS/MAPK3/NRAS/PIK3CA/RB1/TGFBR1/TP53	9
EIF2S1/IL6/JUN/MAP3K5/MAPK8/MAPK9/PIK3CA/PRKAA2/XBP1	9
CRYAB/HRAS/NRAS/PIK3CA/PRKAA2/SIRT1	6
HRAS/MAP3K5/MAPK3/MAPK8/MAPK9/NRAS/PIK3CA/TP53	8
HRAS/MAPK3/MAPK8/MAPK9/NRAS/PIK3CA/SOCS1/STAT3	8
HRAS/NRAS/PIK3CA/PRKAA2/SIRT1/TP53/ULK1	7
CYBB/EGFR/IL6/JUN/MAPK3/MAPK8/MAPK9/PIK3CA/RPL8/STAT3/TLR4	11
EGFR/HRAS/JUN/MAPK3/MAPK8/MAPK9/NRAS	7
CDKN1A/CDKN2A/HRAS/JUN/MAPK3/NRAS/PIK3CA/RB1/STAT3/TP53	10
MAPK3/MAPK8/MAPK9/PIK3CA/SOCS1	5
CDKN1A/CDKN2A/MAP3K5/MAPK3/PIK3CA/TP53	6
AIFM2/CDKN1A/CDKN2A/RRM2/STEAP3/TP53	6
HRAS/MAPK3/MAPK8/MAPK9/NRAS/PIK3CA/PRKAA2/SOCS1	8
IL6/JUN/MAPK3/MAPK8/MAPK9/PIK3CA/TLR4	7
HRAS/JUN/MAPK3/MAPK8/MAPK9/NRAS/PIK3CA	7
IL6/JUN/MAPK3/MAPK8/MAPK9/TLR4	6
CYBB/JUN/MAPK3/NCF2/PTGS2/TLR4	6
IL6/MAPK8/MAPK9/PIK3CA/PRKAA2/STAT3/TRIB3	7
ALOX5/MAPK3/MAPK8/MAPK9/SOCS1/STAT3/TLR4	7
ALOX12/ALOX15B/ALOX5/HRAS/MAPK3/NRAS/PTGS2	7
CDKN1A/EGFR/HRAS/JUN/MAPK3/NRAS/PRKAA2/PTGS2	8
CAV1/EGFR/HRAS/JUN/MAPK3/MAPK8/MAPK9/PIK3CA/VEGFA	9
AKR1C3/ALOX12/ALOX15B/ALOX5/PTGS2	5
EGFR/HRAS/JUN/MAP3K5/MAPK3/MAPK8/MAPK9/NRAS/TGFBR1/TP53/VEGFA	11
CRYAB/EIF2S1/HSPA5/MAP3K5/MAPK8/MAPK9/NFE2L2/XBP1	8
HRAS/MAPK3/NRAS/PIK3CA/STAT3	5
CDKN1A/DDIT4/EGFR/HRAS/IL6/MAPK3/NRAS/PIK3CA/PRKAA2/TLR4/TP53/VEGFA	12
BECN1/GABARAPL1/HRAS/MAPK3/NRAS/PRKAA2/TGFBR1	7
IL6/JUN/MAPK3/MAPK8/MAPK9/PIK3CA/TLR4	7
ATP6V1G2/EGFR/JUN/MAPK8/MAPK9	5
CAV1/CYBB/EIF2S1/HSPA5/IL6/MAPK3/MAPK8/MAPK9/NCF2/PIK3CA	10
ATP6V1G2/CDKN1A/EGFR/HRAS/MAPK3/NRAS/PIK3CA/PTGS2/RB1/TP53/VEGFA	11

HRAS/JUN/MAPK3/NRAS/PIK3CA	5
EIF2S1/HSPA5/MAP3K5/MAPK8/MAPK9/NFE2L2/TP53/UBC/XBP1	9
HIF1A/HRAS/MAPK3/NRAS/PIK3CA/TP53	6
CDKN1A/EGFR/HRAS/IL6/PIK3CA/SOCS1/STAT3	7
HRAS/JUN/MAPK3/MAPK8/MAPK9/NRAS/PIK3CA/TLR4	8
CHAC1/IDH1/PGD/RRM2	4
CDKN1A/PIK3CA/PTGS2/RB1/TP53	5
BECN1/EIF2S1/GABARAPL1/HSPA5/MAP3K5/SQSTM1/TP53/ULK1/ULK2/WIPI1/XBP1	11
ATP6V1G2/IL6/JUN/TLR4/VEGFA	5
BECN1/MAPK8/MAPK9	3
EGFR/HRAS/JUN/MAPK3/NRAS/PIK3CA	6
HRAS/MAPK3/NRAS/PIK3CA	4
AURKA/MAPK3/MAPK8/MAPK9/PIK3CA	5
IL6/JUN/STAT3/TLR4	4
EGFR/HRAS/MAPK3/MAPK8/MAPK9/NRAS/PIK3CA/VEGFA	8
MAPK8/MAPK9/PRKAA2/STAT3	4
ATP6V1G2/CYBB/LAMP2/NCF2/TFRC/TLR4	6
HRAS/IL6/JUN/MAPK3/MAPK8/MAPK9/PIK3CA/TLR4	8
FADS2/PLIN2/PLIN4/UBC	4
ELAVL1/PIK3CA/PRKAA2/SIRT1/ULK1	5
JUN/MAP3K5/MAPK8/MAPK9/NF2/PRKAA2	6
EIF2S1/IL33/IL6/MAPK3/PIK3CA/TLR4	6
GOT1/IDH1	2
HBA1/IL6/TLR4	3
EGFR/HRAS/MAPK3/NRAS	4
AKR1C3/ALOX5/PTGS2	3
IL6/LAMP2/MAPK3/MAPK8/MAPK9/TLR4	6
JUN/MAPK3/MAPK8/MAPK9	4
GLS2/GOT1	2
ATG7/CYBB/MAPK3/NCF2/PIK3CA/TLR4	6
HRAS/MAPK3/NRAS/PIK3CA/STAT3	5
ALOX12/MAPK8/MAPK9/PIK3CA	4
IL6/JUN/MAPK3/MAPK8/MAPK9/TLR4	6
EGFR/HRAS/MAPK3/NRAS/PIK3CA	5
HRAS/MAPK3/NRAS	3
BECN1/MAP3K5/MAPK8/MAPK9/TP53/ULK1/ULK2/WIPI1	8
AKR1C1/AKR1C2/AKR1C3	3
AKR1C3/GCH1	2
CDKN1A/CDKN2A/EGFR/MAPK3/RB1	5
EGFR/HRAS/MAPK3/NRAS/PIK3CA/VEGFA	6
HRAS/MAPK3/NRAS	3
HRAS/MAPK3/NRAS/PIK3CA	4
ALOX12/IL6	2
EGFR/MAPK3/TGFBR1	3
NNMT/SIRT1	2
CDKN1A/CDKN2A/RB1/TP53	4
GLS2/GOT1	2
MAPK3/PIK3CA	2
HBA1/IL6	2
HRAS/MAPK3/NRAS/PIK3CA	4
HRAS/MAPK3/NRAS/PIK3CA/STAT3	5
CAV1/CHMP6/EGFR/HRAS/TFRC/TGFBR1	6
GABARAPL1/GLS2/SLC38A1	3
HAMP/MAPK3/TGFBR1	3
MAPK3/MAPK8/MAPK9/PTGS2	4
LPCAT3/LPIN1/TAZ	3
CD44/IL6/TFRC	3
GOT1/SAT1	2
HRAS/MAPK3/NRAS	3
IL6/PIK3CA/TLR4	3
JUN/MAPK3/MAPK8/MAPK9/PIK3CA	5
DPP4/SLC1A5/SLC3A2	3
EGFR/HRAS/MAPK3/NRAS/PIK3CA	5
PTGS2/TLR4/TNFAIP3	3
JUN/MAPK8/MAPK9/TP53	4
CDKN1A/EGFR/MAPK3	3
PIK3CA/PTGS2	2
IL6/TLR4	2
CYBB/NCF2/PIK3CA	3
GLS2/MAPK3/SLC38A1	3
GOT1/IDH1/PGD	3
IL33/IL6	2
CDKN1A/TP53	2
GOT1	1
TXNRD1	1
CA9	1
HRAS/MAPK3/NRAS/PIK3CA	4
JUN/SIRT1	2
MAPK8/MAPK9	2
DRD4/MAPK8/MAPK9	3
GOT1/IDH1	2
DUOX1/HSPA5	2
HSPA5	1
GLS2	1
CAV1/PIK3CA	2
FBXW7/SOCS1/UBC	3
FADS2	1
AKR1C2/PTGS2	2
FADS2	1
ATP6V1G2	1
NF2	1
IL6/PRKAA2	2

IDH1	1
PGD	1
PRKAA2	1
HRAS/MAP3K5/NRAS/PRKAA2	4
MAPK3/PIK3CA	2
GOT1	1
PRKAA2/SIRT1	2
HMOX1	1
IL6	1
HRAS/MAPK3/NRAS	3
CDKN1A/IL6/TP53	3
PIK3CA	1
ENPP2	1
IL6	1
JUN	1
MAPK3/PIK3CA	2
GOT1	1
ATP6V1G2	1
AURKA/MAPK3	2
FANCD2	1
RRM2	1
FADS2	1
GDF15/IL33/IL6/TGFBR1	4
CAV1	1
LPIN1	1
NF2/TGFBR1	2
PIK3CA	1
AKR1C1	1
HSPA5	1
ATP6V1G2	1
RRM2	1
IDH1	1
CD44	1
PIK3CA	1
MAPK3	1
IL6	1
EIF2S1/IL6/PIK3CA/TP53/ZNF419	5
LAMP2	1
RRM2	1
EGFR/VEGFA	2
ATP6V1G2	1
MAPK3	1
MAPK3	1
GCH1	1
RPL8	1
MAPK3	1
EIF2S1	1
DRD4	1
