# Supplementary material for: A Ferroptosis-Related Prognostic Risk Score Model to Predict Clinical Significance and Immunogenic Characteristics in Glioblastoma Multiforme
Source: Oxid Med Cell Longev. 2021 Nov 9;2021:9107857. doi: 10.1155/2021/9107857 (PMC8596022; doi:10.1155/2021/9107857)
Supplement: Supplementary 2 — Table S1: DEGs between GBM and normal brain tissue. Table S2: KEGG pathways enriched in ferroptosis-related genes. Table S3: GO enrichment analysis of molecular function (MF). Table S4: GO enrichment analysis of biological process (BP). Table S5: GO enrichment analysis of cellular component (CC). Table S6: cd-Ferr-Geneset1. Table S7: cd-Ferr-geneset2. Table S8: DEG.Subtype1. Table S9: DEG.Subtype2. Table S10: DEG.Subtype3. Table S11: DEG.Subtype4. Table S12: known ferroptosis genes. Table S13: a multifactor regulatory network of the ferroptosis key hub genes. Table S14: Lasso-logistic regression analysis of prognosis factors. Table S15: FRGPRS model applied for TCGA GBM and GSE4412 GBM dataset. [file 9107857.f2.zip › Table S3.pdf]

Table S3. GO enrichment analysis of molecular function (MF)

ID	Description	GeneRati	BgRatio
GO:0031625	ubiquitin protein ligase binding	15/120	254/11509
GO:0044389	ubiquitin-like protein ligase binding	15/120	270/11509
GO:0016651	oxidoreductase activity, acting on NAD(P)H	8/120	79/11509
GO:0050664	oxidoreductase activity, acting on NAD(P)H, oxygen as acceptor	4/120	14/11509
GO:0016701	oxidoreductase activity, acting on single donors with incorporation of molecular oxygen	4/120	23/11509
GO:0016702	oxidoreductase activity, acting on single donors with incorporation of molecular oxygen, incorporation of two atoms of oxygen	4/120	23/11509
GO:0046982	protein heterodimerization activity	13/120	382/11509
GO:0016229	steroid dehydrogenase activity	4/120	28/11509
GO:0005506	iron ion binding	7/120	120/11509
GO:0004674	protein serine/threonine kinase activity	12/120	347/11509
GO:0004707	MAP kinase activity	3/120	13/11509
GO:0008106	alcohol dehydrogenase (NADP+) activity	3/120	13/11509
GO:0016722	oxidoreductase activity, oxidizing metal ions	3/120	13/11509

pvalue	p.adjust	qvalue	geneID	Count
5.69E-08	2.49E-05	2.16E-05	AURKA/BECN1/CDKN1A/EGFR/FBXW7/GABARAPL1/HIF1A/HSPA5/JUN/RB1/SQSTM1/TP53/TRIB3/TXNIP/UBC	15
1.27E-07	2.78E-05	2.41E-05	AURKA/BECN1/CDKN1A/EGFR/FBXW7/GABARAPL1/HIF1A/HSPA5/JUN/RB1/SQSTM1/TP53/TRIB3/TXNIP/UBC	15
1.55E-06	0.000226858	0.000196273	AKR1C1/AKR1C2/AKR1C3/CYBB/DUOX1/NCF2/NOX4/TXNRD1	8
1.04E-05	0.001136555	0.000983321	CYBB/DUOX1/NCF2/NOX4	4
8.54E-05	0.006233317	0.00539292	ALOX12/ALOX15B/ALOX5/PTGS2	4
8.54E-05	0.006233317	0.00539292	ALOX12/ALOX15B/ALOX5/PTGS2	4
0.000170063	0.009870205	0.008539471	ATF3/AURKA/CAV1/CYBB/EGFR/GABPB1/HIF1A/JUN/PANX1/TLR4/TP53/VEGFA/XBP1	13
0.000189647	0.009870205	0.008539471	AKR1C1/AKR1C2/AKR1C3/HSD17B11	4
0.00025281	0.009870205	0.008539471	ALOX12/ALOX15B/ALOX5/FTH1/FTL/RRM2/TF	7
0.000264317	0.009870205	0.008539471	AURKA/EGFR/MAP3K5/MAPK3/MAPK8/MAPK9/PIK3CA/PRKAA2/SQSTM1/TGFBR1/ULK1/ULK2	12
0.000292951	0.009870205	0.008539471	MAPK3/MAPK8/MAPK9	3
0.000292951	0.009870205	0.008539471	AKR1C1/AKR1C2/AKR1C3	3
0.000292951	0.009870205	0.008539471	FTH1/FTL/STEAP3	3
