# Supplementary material for: A Ferroptosis-Related Prognostic Risk Score Model to Predict Clinical Significance and Immunogenic Characteristics in Glioblastoma Multiforme
Source: Oxid Med Cell Longev. 2021 Nov 9;2021:9107857. doi: 10.1155/2021/9107857 (PMC8596022; doi:10.1155/2021/9107857)
Supplement: Supplementary 2 — Table S1: DEGs between GBM and normal brain tissue. Table S2: KEGG pathways enriched in ferroptosis-related genes. Table S3: GO enrichment analysis of molecular function (MF). Table S4: GO enrichment analysis of biological process (BP). Table S5: GO enrichment analysis of cellular component (CC). Table S6: cd-Ferr-Geneset1. Table S7: cd-Ferr-geneset2. Table S8: DEG.Subtype1. Table S9: DEG.Subtype2. Table S10: DEG.Subtype3. Table S11: DEG.Subtype4. Table S12: known ferroptosis genes. Table S13: a multifactor regulatory network of the ferroptosis key hub genes. Table S14: Lasso-logistic regression analysis of prognosis factors. Table S15: FRGPRS model applied for TCGA GBM and GSE4412 GBM dataset. [file 9107857.f2.zip › Table S4.pdf]

Table S4. GO enrichment analysis of biological process (BP)

ID	Description	GeneRatio	BgRatio	pvalue	p.adjust
GO:0042594	response to starvation	23/121	153/11512	1.41E-20	5.05E-17
GO:0006979	response to oxidative stress	30/121	374/11512	1.09E-18	1.94E-15
GO:0031669	cellular response to nutrient levels	23/121	191/11512	2.44E-18	2.90E-15
GO:0034599	cellular response to oxidative stress	25/121	249/11512	5.97E-18	5.33E-15
GO:0009267	cellular response to starvation	19/121	118/11512	1.05E-17	7.15E-15
GO:0071496	cellular response to external stimulus	26/121	284/11512	1.20E-17	7.15E-15
GO:0031668	cellular response to extracellular stimulus	23/121	218/11512	4.86E-17	2.48E-14
GO:0031667	response to nutrient levels	29/121	428/11512	4.28E-16	1.91E-13
GO:0009991	response to extracellular stimulus	29/121	456/11512	2.28E-15	9.05E-13
GO:0010035	response to inorganic substance	29/121	483/11512	1.03E-14	3.66E-12
GO:0072593	reactive oxygen species metabolic process	20/121	228/11512	2.26E-13	7.32E-11
GO:0071248	cellular response to metal ion	17/121	158/11512	5.92E-13	1.76E-10
GO:0010038	response to metal ion	22/121	315/11512	1.33E-12	3.65E-10
GO:0071241	cellular response to inorganic substance	17/121	182/11512	6.01E-12	1.53E-09
GO:0006914	autophagy	23/121	380/11512	7.52E-12	1.68E-09
GO:0061919	process utilizing autophagic mechanism	23/121	380/11512	7.52E-12	1.68E-09
GO:0097193	intrinsic apoptotic signaling pathway	19/121	245/11512	8.53E-12	1.79E-09
GO:0016236	macroautophagy	18/121	224/11512	1.75E-11	3.47E-09
GO:0071276	cellular response to cadmium ion	9/121	33/11512	3.62E-11	6.79E-09
GO:0010506	regulation of autophagy	18/121	259/11512	1.95E-10	3.47E-08
GO:0000302	response to reactive oxygen species	16/121	200/11512	2.73E-10	4.63E-08
GO:0046686	response to cadmium ion	10/121	58/11512	3.85E-10	6.25E-08
GO:0007568	aging	18/121	276/11512	5.48E-10	8.51E-08
GO:0043618	regulation of transcription from RNA polymerase II promoter in response to stress	12/121	103/11512	7.15E-10	1.06E-07
GO:0031331	positive regulation of cellular catabolic process	18/121	284/11512	8.70E-10	1.24E-07
GO:2000377	regulation of reactive oxygen species metabolic process	14/121	159/11512	1.09E-09	1.50E-07
GO:0043620	regulation of DNA-templated transcription in response to stress	12/121	109/11512	1.39E-09	1.81E-07
GO:0009636	response to toxic substance	22/121	451/11512	1.42E-09	1.81E-07
GO:2001233	regulation of apoptotic signaling pathway	19/121	336/11512	1.92E-09	2.36E-07
GO:0009896	positive regulation of catabolic process	19/121	337/11512	2.02E-09	2.40E-07
GO:0036003	positive regulation of transcription from RNA polymerase II promoter in response to stress	7/121	23/11512	2.54E-09	2.92E-07
GO:0034614	cellular response to reactive oxygen species	13/121	143/11512	3.02E-09	3.36E-07
GO:0070482	response to oxygen levels	19/121	348/11512	3.43E-09	3.71E-07
GO:0050678	regulation of epithelial cell proliferation	17/121	278/11512	4.60E-09	4.82E-07
GO:0046677	response to antibiotic	17/121	289/11512	8.24E-09	8.40E-07
GO:0050679	positive regulation of epithelial cell proliferation	13/121	161/11512	1.28E-08	1.27E-06
GO:0070997	neuron death	17/121	303/11512	1.67E-08	1.61E-06
GO:0055072	iron ion homeostasis	9/121	64/11512	1.97E-08	1.85E-06
GO:0035690	cellular response to drug	17/121	312/11512	2.57E-08	2.35E-06
GO:0051186	cofactor metabolic process	21/121	485/11512	2.87E-08	2.56E-06
GO:0001666	response to hypoxia	17/121	318/11512	3.40E-08	2.96E-06
GO:0006879	cellular iron ion homeostasis	8/121	49/11512	3.71E-08	3.15E-06
GO:2000147	positive regulation of cell motility	20/121	448/11512	3.87E-08	3.21E-06
GO:0034198	cellular response to amino acid starvation	7/121	34/11512	5.06E-08	3.99E-06
GO:0036293	response to decreased oxygen levels	17/121	327/11512	5.11E-08	3.99E-06
GO:0006826	iron ion transport	8/121	51/11512	5.15E-08	3.99E-06
GO:0050673	epithelial cell proliferation	17/121	330/11512	5.84E-08	4.36E-06
GO:0046394	carboxylic acid biosynthetic process	18/121	372/11512	5.96E-08	4.36E-06
GO:0051272	positive regulation of cellular component movement	20/121	460/11512	5.98E-08	4.36E-06
GO:0016053	organic acid biosynthetic process	18/121	373/11512	6.21E-08	4.43E-06
GO:0018105	peptidyl-serine phosphorylation	15/121	256/11512	7.04E-08	4.93E-06
GO:1990928	response to amino acid starvation	7/121	36/11512	7.72E-08	5.30E-06
GO:0071900	regulation of protein serine/threonine kinase activity	19/121	426/11512	8.95E-08	6.03E-06
GO:0040017	positive regulation of locomotion	20/121	472/11512	9.13E-08	6.03E-06
GO:0055076	transition metal ion homeostasis	10/121	101/11512	9.71E-08	6.30E-06
GO:0030335	positive regulation of cell migration	19/121	434/11512	1.20E-07	7.64E-06
GO:0042771	intrinsic apoptotic signaling pathway in response to DNA damage by p53 class mediator	7/121	39/11512	1.39E-07	8.67E-06
GO:0001525	angiogenesis	19/121	439/11512	1.43E-07	8.82E-06
GO:0018209	peptidyl-serine modification	15/121	271/11512	1.48E-07	8.98E-06
GO:2001242	regulation of intrinsic apoptotic signaling pathway	11/121	135/11512	1.63E-07	9.71E-06
GO:0006801	superoxide metabolic process	8/121	59/11512	1.67E-07	9.78E-06
GO:0016241	regulation of macroautophagy	11/121	136/11512	1.76E-07	1.01E-05
GO:0048871	multicellular organismal homeostasis	18/121	401/11512	1.85E-07	1.05E-05
GO:0071453	cellular response to oxygen levels	13/121	202/11512	1.89E-07	1.05E-05
GO:0046916	cellular transition metal ion homeostasis	9/121	83/11512	1.99E-07	1.09E-05
GO:0006984	ER-nucleus signaling pathway	7/121	42/11512	2.37E-07	1.28E-05
GO:0033559	unsaturated fatty acid metabolic process	9/121	85/11512	2.44E-07	1.30E-05
GO:0072330	monocarboxylic acid biosynthetic process	15/121	283/11512	2.60E-07	1.37E-05
GO:0071479	cellular response to ionizing radiation	8/121	63/11512	2.82E-07	1.43E-05
GO:0071214	cellular response to abiotic stimulus	15/121	285/11512	2.85E-07	1.43E-05
GO:0104004	cellular response to environmental stimulus	15/121	285/11512	2.85E-07	1.43E-05
GO:0006636	unsaturated fatty acid biosynthetic process	7/121	44/11512	3.30E-07	1.64E-05
GO:0000422	autophagy of mitochondrion	8/121	65/11512	3.61E-07	1.74E-05
GO:0061726	mitochondrion disassembly	8/121	65/11512	3.61E-07	1.74E-05
GO:0045765	regulation of angiogenesis	14/121	253/11512	3.99E-07	1.90E-05
GO:0051348	negative regulation of transferase activity	13/121	216/11512	4.10E-07	1.93E-05
GO:0050730	regulation of peptidyl-tyrosine phosphorylation	13/121	217/11512	4.32E-07	2.00E-05
GO:0042060	wound healing	19/121	473/11512	4.53E-07	2.07E-05
GO:0071480	cellular response to gamma radiation	6/121	29/11512	4.64E-07	2.10E-05
GO:1904019	epithelial cell apoptotic process	9/121	93/11512	5.32E-07	2.37E-05
GO:0048545	response to steroid hormone	16/121	344/11512	5.90E-07	2.60E-05
GO:0001935	endothelial cell proliferation	10/121	124/11512	6.74E-07	2.93E-05
GO:0010039	response to iron ion	6/121	31/11512	7.07E-07	3.04E-05
GO:0042542	response to hydrogen peroxide	10/121	125/11512	7.26E-07	3.08E-05
GO:0051090	regulation of DNA-binding transcription factor activity	16/121	350/11512	7.43E-07	3.12E-05
GO:0034976	response to endoplasmic reticulum stress	13/121	229/11512	7.99E-07	3.31E-05
GO:0006633	fatty acid biosynthetic process	10/121	127/11512	8.41E-07	3.45E-05
GO:1901214	regulation of neuron death	14/121	271/11512	9.15E-07	3.71E-05
GO:0043536	positive regulation of blood vessel endothelial cell migration	7/121	51/11512	9.40E-07	3.77E-05

GO:0071216	cellular response to biotic stimulus	12/121	196/11512	9.80E-07	3.88E-05
GO:0097237	cellular response to toxic substance	12/121	197/11512	1.03E-06	4.01E-05
GO:0042149	cellular response to glucose starvation	6/121	33/11512	1.05E-06	4.01E-05
GO:0042554	superoxide anion generation	6/121	33/11512	1.05E-06	4.01E-05
GO:0042326	negative regulation of phosphorylation	16/121	362/11512	1.16E-06	4.41E-05
GO:1901342	regulation of vasculature development	14/121	280/11512	1.35E-06	5.07E-05
GO:0016239	positive regulation of macroautophagy	7/121	54/11512	1.40E-06	5.20E-05
GO:1901654	response to ketone	11/121	168/11512	1.47E-06	5.41E-05
GO:1901522	positive regulation of transcription from RNA polymerase II promoter involved in cellular response to	5/121	20/11512	1.61E-06	5.86E-05
GO:0001933	negative regulation of protein phosphorylation	15/121	327/11512	1.63E-06	5.86E-05
GO:1902532	negative regulation of intracellular signal transduction	17/121	418/11512	1.65E-06	5.87E-05
GO:0043405	regulation of MAP kinase activity	14/121	289/11512	1.96E-06	6.93E-05
GO:0001938	positive regulation of endothelial cell proliferation	8/121	81/11512	2.00E-06	7.00E-05
GO:0010631	epithelial cell migration	13/121	249/11512	2.05E-06	7.09E-05
GO:0033002	muscle cell proliferation	11/121	175/11512	2.20E-06	7.46E-05
GO:0001936	regulation of endothelial cell proliferation	9/121	110/11512	2.21E-06	7.46E-05
GO:0006631	fatty acid metabolic process	14/121	292/11512	2.22E-06	7.46E-05
GO:0090132	epithelium migration	13/121	251/11512	2.24E-06	7.46E-05
GO:0006690	icosanoid metabolic process	8/121	83/11512	2.41E-06	7.89E-05
GO:0043535	regulation of blood vessel endothelial cell migration	8/121	83/11512	2.41E-06	7.89E-05
GO:0045766	positive regulation of angiogenesis	10/121	143/11512	2.49E-06	8.09E-05
GO:0010660	regulation of muscle cell apoptotic process	7/121	59/11512	2.58E-06	8.30E-05
GO:1903008	organelle disassembly	8/121	84/11512	2.64E-06	8.42E-05
GO:0071456	cellular response to hypoxia	11/121	179/11512	2.74E-06	8.65E-05
GO:0034605	cellular response to heat	9/121	113/11512	2.77E-06	8.67E-05
GO:2000379	positive regulation of reactive oxygen species metabolic process	8/121	85/11512	2.89E-06	8.86E-05
GO:0043542	endothelial cell migration	11/121	180/11512	2.89E-06	8.86E-05
GO:0090130	tissue migration	13/121	257/11512	2.91E-06	8.86E-05
GO:0048732	gland development	16/121	389/11512	2.96E-06	8.96E-05
GO:0006469	negative regulation of protein kinase activity	11/121	181/11512	3.05E-06	9.15E-05
GO:0022411	cellular component disassembly	17/121	439/11512	3.20E-06	9.52E-05
GO:0000041	transition metal ion transport	8/121	88/11512	3.76E-06	0.000110904
GO:0045936	negative regulation of phosphate metabolic process	17/121	446/11512	3.96E-06	0.000115839
GO:0010657	muscle cell apoptotic process	7/121	63/11512	4.05E-06	0.000117365
GO:0010563	negative regulation of phosphorus metabolic process	17/121	447/11512	4.08E-06	0.000117442
GO:0036294	cellular response to decreased oxygen levels	11/121	187/11512	4.19E-06	0.000119465
GO:2001234	negative regulation of apoptotic signaling pathway	11/121	188/11512	4.41E-06	0.000124777
GO:1901652	response to peptide	17/121	452/11512	4.74E-06	0.000133055
GO:0008630	intrinsic apoptotic signaling pathway in response to DNA damage	8/121	91/11512	4.84E-06	0.000133075
GO:0017015	regulation of transforming growth factor beta receptor signaling pathway	8/121	91/11512	4.84E-06	0.000133075
GO:2001235	positive regulation of apoptotic signaling pathway	10/121	154/11512	4.86E-06	0.000133075
GO:0001889	liver development	9/121	121/11512	4.89E-06	0.000133075
GO:0045860	positive regulation of protein kinase activity	17/121	454/11512	5.02E-06	0.000135779
GO:0018108	peptidyl-tyrosine phosphorylation	14/121	314/11512	5.16E-06	0.000138462
GO:1903844	regulation of cellular response to transforming growth factor beta stimulus	8/121	92/11512	5.26E-06	0.000140039
GO:0018212	peptidyl-tyrosine modification	14/121	316/11512	5.55E-06	0.000146763
GO:0051188	cofactor biosynthetic process	13/121	273/11512	5.63E-06	0.000147811
GO:0010508	positive regulation of autophagy	8/121	93/11512	5.70E-06	0.00014856
GO:0048660	regulation of smooth muscle cell proliferation	9/121	124/11512	5.98E-06	0.000153432
GO:0061008	hepaticobiliary system development	9/121	124/11512	5.98E-06	0.000153432
GO:0072332	intrinsic apoptotic signaling pathway by p53 class mediator	7/121	67/11512	6.14E-06	0.00015655
GO:0071236	cellular response to antibiotic	9/121	125/11512	6.38E-06	0.000161566
GO:0071478	cellular response to radiation	10/121	159/11512	6.47E-06	0.000162487
GO:0031100	animal organ regeneration	7/121	68/11512	6.79E-06	0.000167092
GO:0048659	smooth muscle cell proliferation	9/121	126/11512	6.82E-06	0.000167092
GO:0061041	regulation of wound healing	9/121	126/11512	6.82E-06	0.000167092
GO:1904018	positive regulation of vasculature development	10/121	160/11512	6.84E-06	0.000167092
GO:0007179	transforming growth factor beta receptor signaling pathway	10/121	161/11512	7.23E-06	0.000171819
GO:0019362	pyridine nucleotide metabolic process	10/121	161/11512	7.23E-06	0.000171819
GO:0046496	nicotinamide nucleotide metabolic process	10/121	161/11512	7.23E-06	0.000171819
GO:0033673	negative regulation of kinase activity	11/121	198/11512	7.24E-06	0.000171819
GO:0010634	positive regulation of epithelial cell migration	9/121	127/11512	7.27E-06	0.000171819
GO:1900407	regulation of cellular response to oxidative stress	7/121	69/11512	7.49E-06	0.00017581
GO:0055089	fatty acid homeostasis	4/121	13/11512	7.72E-06	0.000179958
GO:1903409	reactive oxygen species biosynthetic process	8/121	97/11512	7.82E-06	0.000181103
GO:1904035	regulation of epithelial cell apoptotic process	7/121	71/11512	9.07E-06	0.000208572
GO:1901653	cellular response to peptide	14/121	330/11512	9.12E-06	0.000208572
GO:0072524	pyridine-containing compound metabolic process	10/121	166/11512	9.47E-06	0.000215246
GO:0061418	regulation of transcription from RNA polymerase II promoter in response to hypoxia	7/121	72/11512	9.96E-06	0.000225004
GO:0071560	cellular response to transforming growth factor beta stimulus	11/121	205/11512	1.01E-05	0.000226185
GO:0036473	cell death in response to oxidative stress	7/121	73/11512	1.09E-05	0.000242123
GO:1902882	regulation of response to oxidative stress	7/121	73/11512	1.09E-05	0.000242123
GO:0032722	positive regulation of chemokine production	6/121	49/11512	1.15E-05	0.000253473
GO:2000060	positive regulation of ubiquitin-dependent protein catabolic process	7/121	74/11512	1.20E-05	0.000261858
GO:0071559	response to transforming growth factor beta	11/121	211/11512	1.32E-05	0.000287922
GO:0033674	positive regulation of kinase activity	17/121	489/11512	1.33E-05	0.000288666
GO:0045926	negative regulation of growth	11/121	212/11512	1.38E-05	0.000295969
GO:1904951	positive regulation of establishment of protein localization	15/121	390/11512	1.39E-05	0.000295969
GO:0043534	blood vessel endothelial cell migration	8/121	105/11512	1.41E-05	0.00029866
GO:2000378	negative regulation of reactive oxygen species metabolic process	6/121	51/11512	1.46E-05	0.000307608
GO:0051100	negative regulation of binding	9/121	139/11512	1.51E-05	0.000317575
GO:0006733	oxidoreduction coenzyme metabolic process	10/121	176/11512	1.58E-05	0.000330056
GO:0033572	transferrin transport	5/121	31/11512	1.61E-05	0.000334403
GO:0070059	intrinsic apoptotic signaling pathway in response to endoplasmic reticulum stress	6/121	52/11512	1.63E-05	0.000336795
GO:0043491	protein kinase B signaling	11/121	217/11512	1.72E-05	0.000352997
GO:0010332	response to gamma radiation	6/121	53/11512	1.83E-05	0.00037225
GO:1901800	positive regulation of proteasomal protein catabolic process	7/121	79/11512	1.85E-05	0.000374081
GO:0015682	ferric iron transport	5/121	32/11512	1.89E-05	0.000379767
GO:0072512	trivalent inorganic cation transport	5/121	32/11512	1.89E-05	0.000379767
GO:0019372	lipoxygenase pathway	4/121	16/11512	1.92E-05	0.000382089
GO:0031960	response to corticosteroid	9/121	144/11512	2.01E-05	0.000398254
GO:1903364	positive regulation of cellular protein catabolic process	8/121	111/11512	2.11E-05	0.00041657
GO:0048661	positive regulation of smooth muscle cell proliferation	7/121	81/11512	2.18E-05	0.000424153
GO:2001243	negative regulation of intrinsic apoptotic signaling pathway	7/121	81/11512	2.18E-05	0.000424153
GO:0071229	cellular response to acid chemical	10/121	183/11512	2.22E-05	0.000430314
GO:0009408	response to heat	9/121	146/11512	2.24E-05	0.000432613
GO:0043406	positive regulation of MAP kinase activity	11/121	225/11512	2.41E-05	0.000462544
GO:1903034	regulation of response to wounding	9/121	149/11512	2.64E-05	0.000503198
GO:0001676	long-chain fatty acid metabolic process	7/121	84/11512	2.76E-05	0.000524007
GO:0007623	circadian rhythm	10/121	188/11512	2.80E-05	0.000528484
GO:0031400	negative regulation of protein modification process	16/121	466/11512	2.86E-05	0.000536164
GO:0071375	cellular response to peptide hormone stimulus	12/121	274/11512	3.03E-05	0.000565677
GO:0051098	regulation of binding	13/121	320/11512	3.06E-05	0.000567229
GO:0030512	negative regulation of transforming growth factor beta receptor signaling pathway	6/121	58/11512	3.08E-05	0.000567229
GO:1903201	regulation of oxidative stress-induced cell death	6/121	58/11512	3.08E-05	0.000567229

GO:0033135	regulation of peptidyl-serine phosphorylation	8/121	118/11512	3.29E-05	0.000602646
GO:1903845	negative regulation of cellular response to transforming growth factor beta stimulus	6/121	59/11512	3.40E-05	0.000619677
GO:0010595	positive regulation of endothelial cell migration	7/121	87/11512	3.47E-05	0.000625422
GO:2001169	regulation of ATP biosynthetic process	7/121	87/11512	3.47E-05	0.000625422
GO:0032496	response to lipopolysaccharide	12/121	279/11512	3.62E-05	0.000648261
GO:0051896	regulation of protein kinase B signaling	10/121	194/11512	3.66E-05	0.000653564
GO:0009314	response to radiation	14/121	374/11512	3.69E-05	0.00065474
GO:0016999	antibiotic metabolic process	8/121	120/11512	3.72E-05	0.000656678
GO:1901216	positive regulation of neuron death	7/121	88/11512	3.74E-05	0.000657023
GO:0097164	ammonium ion metabolic process	9/121	156/11512	3.79E-05	0.000663151
GO:0036499	PERK-mediated unfolded protein response	4/121	19/11512	3.98E-05	0.000685828
GO:0060148	positive regulation of posttranscriptional gene silencing	4/121	19/11512	3.98E-05	0.000685828
GO:2000637	positive regulation of gene silencing by miRNA	4/121	19/11512	3.98E-05	0.000685828
GO:0010632	regulation of epithelial cell migration	10/121	196/11512	4.00E-05	0.000685828
GO:0032436	positive regulation of proteasomal ubiquitin-dependent protein catabolic process	6/121	61/11512	4.12E-05	0.000703947
GO:0043434	response to peptide hormone	14/121	378/11512	4.14E-05	0.000703947
GO:0051403	stress-activated MAPK cascade	11/121	239/11512	4.20E-05	0.000710454
GO:0071902	positive regulation of protein serine/threonine kinase activity	12/121	284/11512	4.30E-05	0.000720916
GO:0007569	cell aging	7/121	90/11512	4.32E-05	0.000720916
GO:0033138	positive regulation of peptidyl-serine phosphorylation	7/121	90/11512	4.32E-05	0.000720916
GO:0019216	regulation of lipid metabolic process	13/121	332/11512	4.49E-05	0.000744716
GO:0019359	nicotinamide nucleotide biosynthetic process	8/121	125/11512	4.98E-05	0.000815372
GO:0019363	pyridine nucleotide biosynthetic process	8/121	125/11512	4.98E-05	0.000815372
GO:1901568	fatty acid derivative metabolic process	8/121	125/11512	4.98E-05	0.000815372
GO:0002237	response to molecule of bacterial origin	12/121	289/11512	5.09E-05	0.000820927
GO:0006953	acute-phase response	5/121	39/11512	5.11E-05	0.000820927
GO:0008631	intrinsic apoptotic signaling pathway in response to oxidative stress	5/121	39/11512	5.11E-05	0.000820927
GO:0046456	icosanoid biosynthetic process	5/121	39/11512	5.11E-05	0.000820927
GO:0010212	response to ionizing radiation	8/121	126/11512	5.27E-05	0.000843737
GO:0051052	regulation of DNA metabolic process	13/121	339/11512	5.56E-05	0.000885717
GO:1903052	positive regulation of proteolysis involved in cellular protein catabolic process	7/121	94/11512	5.72E-05	0.000907415
GO:0019369	arachidonic acid metabolic process	5/121	40/11512	5.79E-05	0.000913901
GO:0072525	pyridine-containing compound biosynthetic process	8/121	128/11512	5.90E-05	0.000924163
GO:0001667	ameboidal-type cell migration	13/121	341/11512	5.91E-05	0.000924163
GO:0031099	regeneration	9/121	166/11512	6.16E-05	0.000960334
GO:0045862	positive regulation of proteolysis	12/121	296/11512	6.42E-05	0.000995413
GO:0002931	response to ischemia	5/121	41/11512	6.54E-05	0.001009801
GO:0043410	positive regulation of MAPK cascade	15/121	447/11512	6.72E-05	0.001033045
GO:0060249	anatomical structure homeostasis	13/121	347/11512	7.05E-05	0.001080312
GO:0051402	neuron apoptotic process	10/121	210/11512	7.16E-05	0.001086905
GO:0097305	response to alcohol	10/121	210/11512	7.16E-05	0.001086905
GO:0007005	mitochondrion organization	14/121	398/11512	7.24E-05	0.001094001
GO:0016049	cell growth	14/121	400/11512	7.64E-05	0.001149465
GO:0071222	cellular response to lipopolysaccharide	9/121	171/11512	7.76E-05	0.001162735
GO:0045732	positive regulation of protein catabolic process	9/121	172/11512	8.11E-05	0.001211135
GO:0043467	regulation of generation of precursor metabolites and energy	8/121	134/11512	8.16E-05	0.00121301
GO:0010507	negative regulation of autophagy	6/121	69/11512	8.31E-05	0.001230994
GO:0001101	response to acid chemical	12/121	305/11512	8.55E-05	0.001260187
GO:0090287	regulation of cellular response to growth factor stimulus	10/121	215/11512	8.71E-05	0.001279286
GO:0000045	autophagosome assembly	6/121	70/11512	9.02E-05	0.001313154
GO:0032642	regulation of chemokine production	6/121	70/11512	9.02E-05	0.001313154
GO:0051222	positive regulation of protein transport	13/121	356/11512	9.14E-05	0.001325989
GO:1902806	regulation of cell cycle G1/S phase transition	8/121	137/11512	9.53E-05	0.001377235
GO:0042752	regulation of circadian rhythm	7/121	102/11512	9.63E-05	0.00138041
GO:0051193	regulation of cofactor metabolic process	7/121	102/11512	9.63E-05	0.00138041
GO:0002791	regulation of peptide secretion	14/121	410/11512	9.93E-05	0.00141758
GO:0071219	cellular response to molecule of bacterial origin	9/121	177/11512	0.000101108	0.001437264
GO:1903203	regulation of oxidative stress-induced neuron death	4/121	24/11512	0.000104867	0.00147217
GO:1990776	response to angiotensin	4/121	24/11512	0.000104867	0.00147217
GO:0010594	regulation of endothelial cell migration	8/121	139/11512	0.000105533	0.00147217
GO:0001894	tissue homeostasis	9/121	178/11512	0.000105563	0.00147217
GO:1905037	autophagosome organization	6/121	72/11512	0.000105627	0.00147217
GO:0031098	stress-activated protein kinase signaling cascade	11/121	265/11512	0.000106469	0.001478141
GO:0044843	cell cycle G1/S phase transition	10/121	221/11512	0.000109472	0.001513934
GO:0030808	regulation of nucleotide biosynthetic process	7/121	105/11512	0.000115694	0.00158159
GO:0055088	lipid homeostasis	7/121	105/11512	0.000115694	0.00158159
GO:1900371	regulation of purine nucleotide biosynthetic process	7/121	105/11512	0.000115694	0.00158159
GO:0048511	rhythmic process	11/121	268/11512	0.000117638	0.001602039
GO:0071260	cellular response to mechanical stimulus	6/121	74/11512	0.00012312	0.001661022
GO:0036475	neuron death in response to oxidative stress	4/121	25/11512	0.000123832	0.001661022
GO:1902175	regulation of oxidative stress-induced intrinsic apoptotic signaling pathway	4/121	25/11512	0.000123832	0.001661022
GO:1902253	regulation of intrinsic apoptotic signaling pathway by p53 class mediator	4/121	25/11512	0.000123832	0.001661022
GO:0006995	cellular response to nitrogen starvation	3/121	10/11512	0.000128801	0.001702085
GO:0030647	aminoglycoside antibiotic metabolic process	3/121	10/11512	0.000128801	0.001702085
GO:0043562	cellular response to nitrogen levels	3/121	10/11512	0.000128801	0.001702085
GO:1990440	positive regulation of transcription from RNA polymerase II promoter in response to endoplasmic ret	3/121	10/11512	0.000128801	0.001702085
GO:0006110	regulation of glycolytic process	6/121	75/11512	0.000132689	0.001746986
GO:1903578	regulation of ATP metabolic process	7/121	108/11512	0.000138104	0.001811596
GO:0071407	cellular response to organic cyclic compound	15/121	477/11512	0.000138642	0.001811998
GO:0030811	regulation of nucleotide catabolic process	6/121	76/11512	0.000142838	0.001848695
GO:0032602	chemokine production	6/121	76/11512	0.000142838	0.001848695
GO:0007178	transmembrane receptor protein serine/threonine kinase signaling pathway	11/121	274/11512	0.000143004	0.001848695
GO:0042759	long-chain fatty acid biosynthetic process	4/121	26/11512	0.000145163	0.001869829
GO:0071901	negative regulation of protein serine/threonine kinase activity	7/121	109/11512	0.000146312	0.001877841
GO:0009612	response to mechanical stimulus	9/121	186/11512	0.000147501	0.001879588
GO:0043393	regulation of protein binding	9/121	186/11512	0.000147501	0.001879588
GO:0051897	positive regulation of protein kinase B signaling	8/121	146/11512	0.000148574	0.001886521
GO:0030099	myeloid cell differentiation	12/121	324/11512	0.000151203	0.001913089
GO:0090092	regulation of transmembrane receptor protein serine/threonine kinase signaling pathway	9/121	188/11512	0.000159923	0.002009403
GO:0006091	generation of precursor metabolites and energy	14/121	429/11512	0.000159941	0.002009403
GO:0016242	negative regulation of macroautophagy	4/121	27/11512	0.000169031	0.002108757
GO:0097421	liver regeneration	4/121	27/11512	0.000169031	0.002108757
GO:0090303	positive regulation of wound healing	5/121	50/11512	0.000171417	0.002131064
GO:0001819	positive regulation of cytokine production	13/121	380/11512	0.000175168	0.002162259
GO:0042532	negative regulation of tyrosine phosphorylation of STAT protein	3/121	11/11512	0.000175744	0.002162259
GO:0051024	positive regulation of immunoglobulin secretion	3/121	11/11512	0.000175744	0.002162259
GO:0048771	tissue remodeling	8/121	150/11512	0.000179066	0.002195562
GO:0051259	protein complex oligomerization	14/121	434/11512	0.000180432	0.002204725
GO:2001244	positive regulation of intrinsic apoptotic signaling pathway	5/121	51/11512	0.000188465	0.002295033
GO:0015804	neutral amino acid transport	4/121	28/11512	0.00019561	0.002365883
GO:1902895	positive regulation of pri-miRNA transcription by RNA polymerase II	4/121	28/11512	0.00019561	0.002365883
GO:1901655	cellular response to ketone	6/121	81/11512	0.000203188	0.002449243
GO:0050708	regulation of protein secretion	13/121	386/11512	0.000204313	0.002454513
GO:0031647	regulation of protein stability	10/121	239/11512	0.000207704	0.002486876
GO:0009411	response to UV	7/121	116/11512	0.000215522	0.002571843

GO:0043470	regulation of carbohydrate catabolic process	6/121	82/11512	0.000217368	0.00258523
GO:0002790	peptide secretion	15/121	499/11512	0.000226748	0.002687828
GO:0009895	negative regulation of catabolic process	10/121	242/11512	0.000229723	0.002693726
GO:0002702	positive regulation of production of molecular mediator of immune response	6/121	83/11512	0.000232316	0.002693726
GO:0032091	negative regulation of protein binding	6/121	83/11512	0.000232316	0.002693726
GO:0070301	cellular response to hydrogen peroxide	6/121	83/11512	0.000232316	0.002693726
GO:0050665	hydrogen peroxide biosynthetic process	3/121	12/11512	0.00023253	0.002693726
GO:0090399	replicative senescence	3/121	12/11512	0.00023253	0.002693726
GO:2000345	regulation of hepatocyte proliferation	3/121	12/11512	0.00023253	0.002693726
GO:0007584	response to nutrient	9/121	198/11512	0.000235916	0.002715315
GO:0009743	response to carbohydrate	9/121	198/11512	0.000235916	0.002715315
GO:0002793	positive regulation of peptide secretion	10/121	243/11512	0.000237485	0.002724582
GO:0072594	establishment of protein localization to organelle	14/121	446/11512	0.000239108	0.002730382
GO:2000058	regulation of ubiquitin-dependent protein catabolic process	7/121	118/11512	0.000239521	0.002730382
GO:0007050	cell cycle arrest	9/121	199/11512	0.000244936	0.002783222
GO:0019915	lipid storage	5/121	54/11512	0.000247441	0.002802757
GO:0032147	activation of protein kinase activity	11/121	292/11512	0.000248825	0.002808291
GO:0001558	regulation of cell growth	12/121	342/11512	0.000249503	0.002808291
GO:0046777	protein autophosphorylation	9/121	200/11512	0.000254241	0.002852616
GO:0032007	negative regulation of TOR signaling	4/121	30/11512	0.000257603	0.002881279
GO:0045453	bone resorption	5/121	55/11512	0.000269918	0.00300959
GO:0051592	response to calcium ion	7/121	121/11512	0.000279517	0.003106902
GO:0062014	negative regulation of small molecule metabolic process	6/121	86/11512	0.000282071	0.00312556
GO:0090398	cellular senescence	5/121	56/11512	0.000293914	0.003232606
GO:0046683	response to organophosphorus	7/121	122/11512	0.000293982	0.003232606
GO:0009266	response to temperature stimulus	9/121	204/11512	0.00029445	0.003232606
GO:0017000	antibiotic biosynthetic process	3/121	13/11512	0.000299974	0.003247903
GO:0045346	regulation of MHC class II biosynthetic process	3/121	13/11512	0.000299974	0.003247903
GO:0090343	positive regulation of cell aging	3/121	13/11512	0.000299974	0.003247903
GO:0051196	regulation of coenzyme metabolic process	6/121	87/11512	0.000300395	0.003247903
GO:0090101	negative regulation of transmembrane receptor protein serine/threonine kinase signaling pathway	6/121	87/11512	0.000300395	0.003247903
GO:0050707	regulation of cytokine secretion	8/121	163/11512	0.000315719	0.003403283
GO:0150076	neuroinflammatory response	5/121	57/11512	0.000319493	0.003433586
GO:0002700	regulation of production of molecular mediator of immune response	7/121	124/11512	0.00032472	0.003479278
GO:0006692	prostanoid metabolic process	4/121	32/11512	0.000332583	0.003521236
GO:0006693	prostaglandin metabolic process	4/121	32/11512	0.000332583	0.003521236
GO:0097300	programmed necrotic cell death	4/121	32/11512	0.000332583	0.003521236
GO:1905898	positive regulation of response to endoplasmic reticulum stress	4/121	32/11512	0.000332583	0.003521236
GO:1900542	regulation of purine nucleotide metabolic process	7/121	125/11512	0.000341027	0.003599957
GO:0050731	positive regulation of peptidyl-tyrosine phosphorylation	8/121	165/11512	0.000342803	0.003608029
GO:0031330	negative regulation of cellular catabolic process	9/121	209/11512	0.000351989	0.003693814
GO:0006275	regulation of DNA replication	6/121	90/11512	0.000361024	0.003777518
GO:0000082	G1/S transition of mitotic cell cycle	9/121	210/11512	0.000364544	0.003803199
GO:2001171	positive regulation of ATP biosynthetic process	4/121	33/11512	0.000375403	0.003873302
GO:2000045	regulation of G1/S transition of mitotic cell cycle	7/121	127/11512	0.000375614	0.003873302
GO:0002070	epithelial cell maturation	3/121	14/11512	0.000378863	0.003873302
GO:0043651	linoleic acid metabolic process	3/121	14/11512	0.000378863	0.003873302
GO:0045342	MHC class II biosynthetic process	3/121	14/11512	0.000378863	0.003873302
GO:1902165	regulation of intrinsic apoptotic signaling pathway in response to DNA damage by p53 class mediato	3/121	14/11512	0.000378863	0.003873302
GO:1902166	negative regulation of intrinsic apoptotic signaling pathway in response to DNA damage by p53 class	3/121	14/11512	0.000378863	0.003873302
GO:2000134	negative regulation of G1/S transition of mitotic cell cycle	6/121	91/11512	0.00038323	0.003906756
GO:0007507	heart development	14/121	468/11512	0.000390075	0.003965203
GO:0006140	regulation of nucleotide metabolic process	7/121	128/11512	0.000393931	0.003981715
GO:0051384	response to glucocorticoid	7/121	128/11512	0.000393931	0.003981715
GO:0002573	myeloid leukocyte differentiation	8/121	169/11512	0.000402669	0.00405128
GO:0042108	positive regulation of cytokine biosynthetic process	5/121	60/11512	0.00040642	0.00405128
GO:1903036	positive regulation of response to wounding	5/121	60/11512	0.00040642	0.00405128
GO:0042035	regulation of cytokine biosynthetic process	6/121	92/11512	0.000406491	0.00405128
GO:1902807	negative regulation of cell cycle G1/S phase transition	6/121	92/11512	0.000406491	0.00405128
GO:0042176	regulation of protein catabolic process	11/121	310/11512	0.00041459	0.004120497
GO:0009746	response to hexose	8/121	170/11512	0.000418894	0.004151704
GO:0009306	protein secretion	14/121	472/11512	0.00042488	0.004199371
GO:0002262	myeloid cell homeostasis	7/121	130/11512	0.00043271	0.00426494
GO:0006978	DNA damage response, signal transduction by p53 class mediator resulting in transcription of p21 cl	3/121	15/11512	0.000469955	0.004521083
GO:0010042	response to manganese ion	3/121	15/11512	0.000469955	0.004521083
GO:0030812	negative regulation of nucleotide catabolic process	3/121	15/11512	0.000469955	0.004521083
GO:0034755	iron ion transmembrane transport	3/121	15/11512	0.000469955	0.004521083
GO:0042772	DNA damage response, signal transduction resulting in transcription	3/121	15/11512	0.000469955	0.004521083
GO:0051198	negative regulation of coenzyme metabolic process	3/121	15/11512	0.000469955	0.004521083
GO:0002639	positive regulation of immunoglobulin production	4/121	35/11512	0.000472636	0.004521083
GO:0038083	peptidyl-tyrosine autophosphorylation	4/121	35/11512	0.000472636	0.004521083
GO:0045124	regulation of bone resorption	4/121	35/11512	0.000472636	0.004521083
GO:0090049	regulation of cell migration involved in sprouting angiogenesis	4/121	35/11512	0.000472636	0.004521083
GO:0090311	regulation of protein deacetylation	4/121	35/11512	0.000472636	0.004521083
GO:0007517	muscle organ development	11/121	317/11512	0.000500313	0.00477304
GO:0034284	response to monosaccharide	8/121	175/11512	0.000508198	0.004812716
GO:0070374	positive regulation of ERK1 and ERK2 cascade	8/121	175/11512	0.000508198	0.004812716
GO:0048608	reproductive structure development	12/121	370/11512	0.000508518	0.004812716
GO:0030330	DNA damage response, signal transduction by p53 class mediator	6/121	96/11512	0.000510779	0.004821319
GO:0002696	positive regulation of leukocyte activation	10/121	268/11512	0.000516626	0.00486365
GO:1901215	negative regulation of neuron death	8/121	176/11512	0.000527788	0.004955652
GO:0032434	regulation of proteasomal ubiquitin-dependent protein catabolic process	6/121	97/11512	0.000539843	0.005029136
GO:0045598	regulation of fat cell differentiation	6/121	97/11512	0.000539843	0.005029136
GO:0098754	detoxification	6/121	97/11512	0.000539843	0.005029136
GO:0009108	coenzyme biosynthetic process	9/121	222/11512	0.000546357	0.005063962
GO:0061458	reproductive system development	12/121	373/11512	0.00054642	0.005063962
GO:0042448	progesterone metabolic process	3/121	16/11512	0.000573983	0.005278273
GO:0072574	hepatocyte proliferation	3/121	16/11512	0.000573983	0.005278273
GO:0072575	epithelial cell proliferation involved in liver morphogenesis	3/121	16/11512	0.000573983	0.005278273
GO:1903524	positive regulation of blood circulation	5/121	65/11512	0.000589533	0.00540014
GO:0045637	regulation of myeloid cell differentiation	8/121	179/11512	0.000590262	0.00540014
GO:0032869	cellular response to insulin stimulus	8/121	180/11512	0.000612369	0.00558806
GO:0002521	leukocyte differentiation	13/121	433/11512	0.000614264	0.005591058
GO:0050714	positive regulation of protein secretion	9/121	226/11512	0.00062138	0.005627113
GO:0072331	signal transduction by p53 class mediator	9/121	226/11512	0.00062138	0.005627113
GO:0050871	positive regulation of B cell activation	5/121	66/11512	0.000632555	0.005713815
GO:0050999	regulation of nitric-oxide synthase activity	4/121	38/11512	0.00065032	0.005844689
GO:1902893	regulation of pri-miRNA transcription by RNA polymerase II	4/121	38/11512	0.00065032	0.005844689
GO:0042089	cytokine biosynthetic process	6/121	101/11512	0.00066919	0.005999168
GO:0006754	ATP biosynthetic process	7/121	140/11512	0.00067489	0.00600176
GO:0032006	regulation of TOR signaling	5/121	67/11512	0.000677889	0.00600176
GO:0034103	regulation of tissue remodeling	5/121	67/11512	0.000677889	0.00600176
GO:0034644	cellular response to UV	5/121	67/11512	0.000677889	0.00600176
GO:1905897	regulation of response to endoplasmic reticulum stress	5/121	67/11512	0.000677889	0.00600176
GO:0002440	production of molecular mediator of immune response	8/121	183/11512	0.000682739	0.006029732

GO:0016137	glycoside metabolic process	3/121	17/11512	0.000691651	0.006063417
GO:0072576	liver morphogenesis	3/121	17/11512	0.000691651	0.006063417
GO:2000269	regulation of fibroblast apoptotic process	3/121	17/11512	0.000691651	0.006063417
GO:0042107	cytokine metabolic process	6/121	102/11512	0.000704993	0.006162715
GO:0062012	regulation of small molecule metabolic process	12/121	384/11512	0.000706432	0.006162715
GO:1901615	organic hydroxy compound metabolic process	13/121	440/11512	0.000713345	0.006207842
GO:0042509	regulation of tyrosine phosphorylation of STAT protein	5/121	68/11512	0.000725613	0.006285669
GO:0050867	positive regulation of cell activation	10/121	280/11512	0.000725812	0.006285669
GO:0043281	regulation of cysteine-type endopeptidase activity involved in apoptotic process	8/121	185/11512	0.000733177	0.006333078
GO:0006732	coenzyme metabolic process	11/121	332/11512	0.000734836	0.006333078
GO:0030968	endoplasmic reticulum unfolded protein response	6/121	103/11512	0.000742263	0.006381382
GO:0006511	ubiquitin-dependent protein catabolic process	13/121	442/11512	0.000744018	0.006381382
GO:0051251	positive regulation of lymphocyte activation	9/121	232/11512	0.000749578	0.006413655
GO:0050663	cytokine secretion	8/121	186/11512	0.000759499	0.006482999
GO:0050864	regulation of B cell activation	6/121	104/11512	0.000781039	0.006650947
GO:0051205	protein insertion into membrane	4/121	40/11512	0.00079224	0.006714281
GO:0070265	necrotic cell death	4/121	40/11512	0.00079224	0.006714281
GO:2001252	positive regulation of chromosome organization	7/121	144/11512	0.000797586	0.006743567
GO:1905477	positive regulation of protein localization to membrane	6/121	105/11512	0.000821363	0.006894742
GO:0046716	muscle cell cellular homeostasis	3/121	18/11512	0.00082364	0.006894742
GO:0051023	regulation of immunoglobulin secretion	3/121	18/11512	0.00082364	0.006894742
GO:1902176	negative regulation of oxidative stress-induced intrinsic apoptotic signaling pathway	3/121	18/11512	0.00082364	0.006894742
GO:0007260	tyrosine phosphorylation of STAT protein	5/121	70/11512	0.000828547	0.006894742
GO:0048708	astrocyte differentiation	5/121	70/11512	0.000828547	0.006894742
GO:0061136	regulation of proteasomal protein catabolic process	7/121	145/11512	0.000830857	0.006894742
GO:0022407	regulation of cell-cell adhesion	11/121	337/11512	0.000830925	0.006894742
GO:0019941	modification-dependent protein catabolic process	13/121	448/11512	0.000842802	0.006963129
GO:1903039	positive regulation of leukocyte cell-cell adhesion	8/121	189/11512	0.000843069	0.006963129
GO:0006096	glycolytic process	6/121	106/11512	0.000863276	0.007011554
GO:0006757	ATP generation from ADP	6/121	106/11512	0.000863276	0.007011554
GO:0034101	erythrocyte homeostasis	6/121	106/11512	0.000863276	0.007011554
GO:0060968	regulation of gene silencing	6/121	106/11512	0.000863276	0.007011554
GO:0030810	positive regulation of nucleotide biosynthetic process	4/121	41/11512	0.00087082	0.007011554
GO:0051055	negative regulation of lipid biosynthetic process	4/121	41/11512	0.00087082	0.007011554
GO:0061912	selective autophagy	4/121	41/11512	0.00087082	0.007011554
GO:1900373	positive regulation of purine nucleotide biosynthetic process	4/121	41/11512	0.00087082	0.007011554
GO:1900408	negative regulation of cellular response to oxidative stress	4/121	41/11512	0.00087082	0.007011554
GO:1902475	L-alpha-amino acid transmembrane transport	4/121	41/11512	0.00087082	0.007011554
GO:1903202	negative regulation of oxidative stress-induced cell death	4/121	41/11512	0.00087082	0.007011554
GO:0051054	positive regulation of DNA metabolic process	8/121	190/11512	0.000872514	0.007011554
GO:0070372	regulation of ERK1 and ERK2 cascade	9/121	238/11512	0.000898617	0.007205094
GO:0006986	response to unfolded protein	7/121	147/11512	0.000900689	0.007205515
GO:0009206	purine ribonucleoside triphosphate biosynthetic process	7/121	148/11512	0.000937301	0.00748163
GO:0050865	regulation of cell activation	13/121	454/11512	0.000952416	0.00750332
GO:0007595	lactation	4/121	42/11512	0.000954739	0.00750332
GO:0042304	regulation of fatty acid biosynthetic process	4/121	42/11512	0.000954739	0.00750332
GO:0046850	regulation of bone remodeling	4/121	42/11512	0.000954739	0.00750332
GO:0050732	negative regulation of peptidyl-tyrosine phosphorylation	4/121	42/11512	0.000954739	0.00750332
GO:1902883	negative regulation of response to oxidative stress	4/121	42/11512	0.000954739	0.00750332
GO:1903580	positive regulation of ATP metabolic process	4/121	42/11512	0.000954739	0.00750332
GO:0006925	inflammatory cell apoptotic process	3/121	19/11512	0.000970602	0.007537804
GO:0046426	negative regulation of JAK-STAT cascade	3/121	19/11512	0.000970602	0.007537804
GO:0055093	response to hyperoxia	3/121	19/11512	0.000970602	0.007537804
GO:1902254	negative regulation of intrinsic apoptotic signaling pathway by p53 class mediator	3/121	19/11512	0.000970602	0.007537804
GO:1902644	tertiary alcohol metabolic process	3/121	19/11512	0.000970602	0.007537804
GO:0043632	modification-dependent macromolecule catabolic process	13/121	455/11512	0.000971802	0.007537804
GO:0009145	purine nucleoside triphosphate biosynthetic process	7/121	149/11512	0.000975076	0.007546792
GO:0042866	pyruvate biosynthetic process	6/121	109/11512	0.000998975	0.007711754
GO:0060964	regulation of gene silencing by miRNA	5/121	73/11512	0.001002874	0.007711754
GO:0098869	cellular oxidant detoxification	5/121	73/11512	0.001002874	0.007711754
GO:0042743	hydrogen peroxide metabolic process	4/121	43/11512	0.001044191	0.007995005
GO:0070849	response to epidermal growth factor	4/121	43/11512	0.001044191	0.007995005
GO:0022612	gland morphogenesis	6/121	110/11512	0.001047671	0.008004477
GO:0048145	regulation of fibroblast proliferation	5/121	74/11512	0.001066624	0.00811453
GO:1901570	fatty acid derivative biosynthetic process	5/121	74/11512	0.001066624	0.00811453
GO:0009201	ribonucleoside triphosphate biosynthetic process	7/121	152/11512	0.001095648	0.00829994
GO:1901796	regulation of signal transduction by p53 class mediator	7/121	152/11512	0.001095648	0.00829994
GO:0032675	regulation of interleukin-6 production	6/121	111/11512	0.001098171	0.008301432
GO:0052547	regulation of peptidase activity	11/121	350/11512	0.001130688	0.008359841
GO:0000423	mitophagy	3/121	20/11512	0.001133168	0.008359841
GO:0044346	fibroblast apoptotic process	3/121	20/11512	0.001133168	0.008359841
GO:0090050	positive regulation of cell migration involved in sprouting angiogenesis	3/121	20/11512	0.001133168	0.008359841
GO:1904893	negative regulation of STAT cascade	3/121	20/11512	0.001133168	0.008359841
GO:0006476	protein deacetylation	5/121	75/11512	0.001133334	0.008359841
GO:0019217	regulation of fatty acid metabolic process	5/121	75/11512	0.001133334	0.008359841
GO:0032755	positive regulation of interleukin-6 production	5/121	75/11512	0.001133334	0.008359841
GO:0048144	fibroblast proliferation	5/121	75/11512	0.001133334	0.008359841
GO:0060147	regulation of posttranscriptional gene silencing	5/121	75/11512	0.001133334	0.008359841
GO:0060966	regulation of gene silencing by RNA	5/121	75/11512	0.001133334	0.008359841
GO:0030522	intracellular receptor signaling pathway	9/121	246/11512	0.001134014	0.008359841
GO:0031396	regulation of protein ubiquitination	7/121	153/11512	0.001138342	0.008364764
GO:0061614	pri-miRNA transcription by RNA polymerase II	4/121	44/11512	0.001139371	0.008364764
GO:1903362	regulation of cellular protein catabolic process	8/121	199/11512	0.001176504	0.008619645
GO:0010821	regulation of mitochondrion organization	7/121	154/11512	0.001182333	0.008644599
GO:0046031	ADP metabolic process	6/121	113/11512	0.001204763	0.008772641
GO:0050715	positive regulation of cytokine secretion	6/121	113/11512	0.001204763	0.008772641
GO:1904705	regulation of vascular smooth muscle cell proliferation	4/121	45/11512	0.001240473	0.008995952
GO:1990874	vascular smooth muscle cell proliferation	4/121	45/11512	0.001240473	0.008995952
GO:0090288	negative regulation of cellular response to growth factor stimulus	6/121	114/11512	0.001260944	0.009125859
GO:0034612	response to tumor necrosis factor	9/121	250/11512	0.001269184	0.009166842
GO:0031349	positive regulation of defense response	12/121	411/11512	0.001272636	0.009166842
GO:0003018	vascular process in circulatory system	7/121	156/11512	0.001274314	0.009166842
GO:0048305	immunoglobulin secretion	3/121	21/11512	0.001311942	0.009324722
GO:0051195	negative regulation of cofactor metabolic process	3/121	21/11512	0.001311942	0.009324722
GO:0060571	morphogenesis of an epithelial fold	3/121	21/11512	0.001311942	0.009324722
GO:0071379	cellular response to prostaglandin stimulus	3/121	21/11512	0.001311942	0.009324722
GO:0089718	amino acid import across plasma membrane	3/121	21/11512	0.001311942	0.009324722
GO:2000108	positive regulation of leukocyte apoptotic process	3/121	21/11512	0.001311942	0.009324722
GO:0046890	regulation of lipid biosynthetic process	7/121	157/11512	0.00132236	0.009380082
GO:0061900	glial cell activation	4/121	46/11512	0.001347692	0.009540803
GO:0031058	positive regulation of histone modification	5/121	78/11512	0.001352064	0.009552799
GO:0001659	temperature homeostasis	7/121	158/11512	0.001371814	0.009673184
GO:2000116	regulation of cysteine-type endopeptidase activity	8/121	204/11512	0.001378788	0.009684722
GO:0070371	ERK1 and ERK2 cascade	9/121	253/11512	0.001378879	0.009684722
GO:0046434	organophosphate catabolic process	8/121	205/11512	0.001422382	0.009970644

qvalue
3.20E-17
1.23E-15
1.84E-15
3.38E-15
4.53E-15
4.53E-15
1.57E-14
1.21E-13
5.74E-13
2.32E-12
4.64E-11
1.11E-10
2.32E-10
9.70E-10
1.06E-09
1.06E-09
1.14E-09
2.20E-09
4.30E-09
2.20E-08
2.93E-08
3.96E-08
5.39E-08
6.73E-08
7.87E-08
9.52E-08
1.15E-07
1.15E-07
1.50E-07
1.52E-07
1.85E-07
2.13E-07
2.35E-07
3.06E-07
5.32E-07
8.05E-07
1.02E-06
1.17E-06
1.49E-06
1.62E-06
1.87E-06
2.00E-06
2.03E-06
2.53E-06
2.53E-06
2.53E-06
2.76E-06
2.76E-06
2.76E-06
2.81E-06
3.12E-06
3.36E-06
3.82E-06
3.82E-06
3.99E-06
4.84E-06
5.50E-06
5.59E-06
5.69E-06
6.15E-06
6.20E-06
6.42E-06
6.62E-06
6.68E-06
6.91E-06
8.11E-06
8.25E-06
8.66E-06
9.08E-06
9.08E-06
9.08E-06
1.04E-05
1.10E-05
1.10E-05
1.20E-05
1.22E-05
1.27E-05
1.31E-05
1.33E-05
1.50E-05
1.65E-05
1.86E-05
1.93E-05
1.95E-05
1.98E-05
2.10E-05
2.19E-05
2.35E-05
2.39E-05

2.46E-05
2.54E-05
2.54E-05
2.54E-05
2.79E-05
3.21E-05
3.30E-05
3.43E-05
3.72E-05
3.72E-05
3.72E-05
4.39E-05
4.44E-05
4.49E-05
4.73E-05
4.73E-05
4.73E-05
4.73E-05
5.00E-05
5.00E-05
5.13E-05
5.26E-05
5.33E-05
5.48E-05
5.49E-05
5.61E-05
5.61E-05
5.61E-05
5.68E-05
5.80E-05
6.03E-05
7.03E-05
7.34E-05
7.44E-05
7.44E-05
7.57E-05
7.91E-05
8.43E-05
8.43E-05
8.43E-05
8.43E-05
8.43E-05
8.60E-05
8.77E-05
8.87E-05
9.30E-05
9.37E-05
9.41E-05
9.72E-05
9.72E-05
9.92E-05
0.000102
0.000103
0.000106
0.000106
0.000106
0.000106
0.000109
0.000109
0.000109
0.000109
0.000109
0.000111
0.000114
0.000115
0.000132
0.000132
0.000136
0.000143
0.000143
0.000153
0.000153
0.000161
0.000166
0.000182
0.000183
0.000188
0.000188
0.000189
0.000195
0.000201
0.000209
0.000212
0.000213
0.000224
0.000236
0.000237
0.000241
0.000241
0.000242
0.000252
0.000264
0.000269
0.000269
0.000273
0.000274
0.000293
0.000319
0.000332
0.000335
0.00034
0.000358
0.000359
0.000359
0.000359

0.000382
0.000393
0.000396
0.000396
0.000411
0.000414
0.000415
0.000416
0.000416
0.00042
0.000435
0.000435
0.000435
0.000435
0.000446
0.000446
0.00045
0.000457
0.000457
0.000457
0.000472
0.000517
0.000517
0.000517
0.00052
0.00052
0.00052
0.00052
0.000535
0.000561
0.000575
0.000579
0.000586
0.000586
0.000609
0.000631
0.00064
0.000655
0.000685
0.000689
0.000689
0.000693
0.000728
0.000737
0.000768
0.000769
0.00078
0.000799
0.000811
0.000832
0.000832
0.00084
0.000873
0.000875
0.000875
0.000898
0.000911
0.000933
0.000933
0.000933
0.000933
0.000933
0.000937
0.000959
0.001002
0.001002
0.001002
0.001015
0.001053
0.001053
0.001053
0.001053
0.001079
0.001079
0.001079
0.001079
0.001107
0.001148
0.001148
0.001172
0.001172
0.001172
0.001185
0.00119
0.001191
0.001191
0.001195
0.001212
0.001273
0.001273
0.001336
0.001336
0.00135
0.00137
0.00137
0.00137
0.001391
0.001397
0.001454
0.001499
0.001499
0.001552
0.001555
0.001576
0.00163

0.001638
0.001703
0.001707
0.001707
0.001707
0.001707
0.001707
0.001707
0.001707
0.001721
0.001721
0.001727
0.00173
0.00173
0.001764
0.001776
0.00178
0.00178
0.001808
0.001826
0.001907
0.001969
0.001981
0.002049
0.002049
0.002049
0.002058
0.002058
0.002058
0.002058
0.002058
0.002157
0.002176
0.002205
0.002231
0.002231
0.002231
0.002231
0.002281
0.002286
0.002341
0.002394
0.00241
0.002455
0.002455
0.002455
0.002455
0.002455
0.002455
0.002455
0.002476
0.002513
0.002523
0.002523
0.002567
0.002567
0.002567
0.002567
0.002567
0.002611
0.002631
0.002661
0.002703
0.002865
0.002865
0.002865
0.002865
0.002865
0.002865
0.002865
0.002865
0.002865
0.002865
0.002865
0.003025
0.00305
0.00305
0.00305
0.003055
0.003082
0.00314
0.003187
0.003187
0.003187
0.003209
0.003209
0.003345
0.003345
0.003345
0.003422
0.003422
0.003541
0.003543
0.003566
0.003566
0.003621
0.003704
0.003704
0.003802
0.003803
0.003803
0.003803
0.003803
0.003803
0.003821

0.003842
0.003842
0.003842
0.003905
0.003905
0.003934
0.003983
0.003983
0.004013
0.004013
0.004044
0.004044
0.004064
0.004108
0.004215
0.004255
0.004255
0.004273
0.004369
0.004369
0.004369
0.004369
0.004369
0.004369
0.004369
0.004369
0.004413
0.004413
0.004443
0.004443
0.004443
0.004443
0.004443
0.004443
0.004443
0.004443
0.004443
0.004443
0.004443
0.004443
0.004566
0.004566
0.004741
0.004755
0.004755
0.004755
0.004755
0.004755
0.004755
0.004755
0.004777
0.004777
0.004777
0.004777
0.004777
0.004777
0.004782
0.004887
0.004887
0.004887
0.005066
0.005066
0.005072
0.005142
0.005142
0.00526
0.00526
0.005261
0.005298
0.005298
0.005298
0.005298
0.005298
0.005298
0.005298
0.005298
0.005298
0.005298
0.005298
0.005298
0.005301
0.005301
0.005462
0.005478
0.005559
0.005559
0.005701
0.005701
0.005783
0.005809
0.005809
0.005809
0.005909
0.005909
0.005909
0.005909
0.005909
0.005909
0.005944
0.006046
0.006054
0.00613
0.006137
0.006137
0.006318

geneID
AKR1C3/ATF3/ATG7/BECN1/CDKN1A/EIF2S1/GABARAPL1/HAMP/HSPA5/JUN/LAMP2/MAP3K5/MAPK3/MAPK8/NFE2L2/PRKAA2/SIRT1/TP53/ULK1/ULK2/WIPI1/XBP1/ZFP36
AKR1C3/ATG7/BECN1/CRYAB/CYBB/DUOX1/EGFR/EIF2S1/FBXW7/GCH1/HIF1A/HMOX1/IDH1/IL6/JUN/MAP3K5/MAPK3/MAPK8/MAPK9/NCF2/NFE2L2/NOX4/PRKAA2/PTGS2/SIRT1/T
AKR1C3/ATF3/ATG7/BECN1/CDKN1A/CYBB/EIF2S1/GABARAPL1/HMOX1/HSPA5/JUN/LAMP2/MAP3K5/MAPK3/MAPK8/NFE2L2/PRKAA2/PTGS2/SIRT1/TP53/ULK1/WIPI1/XBP1
AKR1C3/ATG7/BECN1/CYBB/EGFR/EIF2S1/FBXW7/GCH1/HIF1A/HMOX1/IL6/JUN/MAP3K5/MAPK3/MAPK8/MAPK9/NCF2/NFE2L2/NOX4/PRKAA2/SIRT1/TLR4/TNFAIP3/TP53/TXNRD1
AKR1C3/ATF3/ATG7/BECN1/CDKN1A/EIF2S1/GABARAPL1/HSPA5/JUN/LAMP2/MAP3K5/MAPK3/MAPK8/NFE2L2/PRKAA2/SIRT1/TP53/WIPI1/XBP1
AKR1C3/ATF3/ATG7/BECN1/CDKN1A/CYBB/EGFR/EIF2S1/GABARAPL1/GOT1/HMOX1/HSPA5/JUN/LAMP2/MAP3K5/MAPK3/MAPK8/NFE2L2/PRKAA2/PTGS2/SIRT1/TLR4/TP53/ULK1/W
AKR1C3/ATF3/ATG7/BECN1/CDKN1A/CYBB/EIF2S1/GABARAPL1/HMOX1/HSPA5/JUN/LAMP2/MAP3K5/MAPK3/MAPK8/NFE2L2/PRKAA2/PTGS2/SIRT1/TP53/ULK1/WIPI1/XBP1
AKR1C3/ATF3/ATG7/BECN1/CDKN1A/CYBB/EGFR/EIF2S1/GABARAPL1/GDF15/HAMP/HMOX1/HSPA5/JUN/LAMP2/MAP3K5/MAPK3/MAPK8/NFE2L2/PANX1/PRKAA2/PTGS2/SIRT1/TP
AKR1C3/ATF3/ATG7/BECN1/CDKN1A/CYBB/EGFR/EIF2S1/GABARAPL1/GDF15/HAMP/HMOX1/HSPA5/JUN/LAMP2/MAP3K5/MAPK3/MAPK8/NFE2L2/PANX1/PRKAA2/PTGS2/SIRT1/TP
AKR1C3/ATG7/BECN1/CAV1/CRYAB/CYBB/EGFR/EIF2S1/GCH1/GOT1/HAMP/HIF1A/HMOX1/HSPA5/IL6/JUN/MAP3K5/MAPK3/MAPK8/MAPK9/MT1G/NFE2L2/PRKAA2/PTGS2/SIRT1/T
AKR1C3/ALOX12/BECN1/CAV1/CDKN1A/CRYAB/CYBB/DDIT4/DUOX1/EGFR/GCH1/GLS2/HIF1A/NCF2/NFE2L2/NOX4/PTGS2/STAT3/TLR4/TP53
AKR1C3/BECN1/CYBB/EGFR/EIF2S1/HMOX1/HSPA5/JUN/MAPK3/MAPK8/MAPK9/MT1G/NFE2L2/PRKAA2/PTGS2/TF/TFR2
AKR1C3/BECN1/CAV1/CYBB/EGFR/EIF2S1/GOT1/HAMP/HIF1A/HMOX1/HSPA5/JUN/MAPK3/MAPK8/MAPK9/MT1G/NFE2L2/PRKAA2/PTGS2/TF/TFR2/TXNIP
AKR1C3/BECN1/CYBB/EGFR/EIF2S1/HMOX1/HSPA5/JUN/MAPK3/MAPK8/MAPK9/MT1G/NFE2L2/PRKAA2/PTGS2/TF/TFR2
ATG7/ATP6V1G2/BECN1/CHMP6/FBXW7/GABARAPL1/HIF1A/HMOX1/LAMP2/MAPK3/MAPK8/MTDH/PIK3CA/PRKAA2/SIRT1/SQSTM1/STAT3/TP53/TRIB3/ULK1/ULK2/WIPI1/XBP1
ATG7/ATP6V1G2/BECN1/CHMP6/FBXW7/GABARAPL1/HIF1A/HMOX1/LAMP2/MAPK3/MAPK8/MTDH/PIK3CA/PRKAA2/SIRT1/SQSTM1/STAT3/TP53/TRIB3/ULK1/ULK2/WIPI1/XBP1
BECN1/CAV1/CD44/CDKN1A/CHAC1/DDIT4/FBXW7/HIF1A/HMOX1/HRAS/MAP3K5/MUC1/NFE2L2/PTGS2/SIRT1/TP53/TP63/TRIB3/XBP1
ATG7/ATP6V1G2/BECN1/CHMP6/GABARAPL1/HIF1A/HMOX1/LAMP2/MAPK3/MAPK8/PIK3CA/PRKAA2/SIRT1/SQSTM1/TP53/ULK1/ULK2/WIPI1
AKR1C3/CYBB/EGFR/HMOX1/JUN/MAPK3/MAPK8/MAPK9/MT1G
ATG7/ATP6V1G2/BECN1/FBXW7/HIF1A/HMOX1/MAPK3/MAPK8/MTDH/PIK3CA/PRKAA2/SIRT1/STAT3/TP53/TRIB3/ULK1/ULK2/XBP1
AKR1C3/BECN1/CRYAB/EGFR/GCH1/HMOX1/IL6/JUN/MAP3K5/MAPK3/MAPK8/MAPK9/NFE2L2/SIRT1/TNFAIP3/TXNIP
AKR1C3/CYBB/EGFR/GOT1/HMOX1/JUN/MAPK3/MAPK8/MAPK9/MT1G
ALOX12/ATG7/BECN1/CDKN1A/CDKN2A/CRYAB/EIF2S1/HAMP/HRAS/JUN/MAPK3/NFE2L2/NOX4/PTGS2/SIRT1/STAT3/TP53/TP63
ATF3/BACH1/CA9/HIF1A/HMOX1/HSPA5/JUN/MUC1/NFE2L2/TP53/UBC/VEGFA
AURKA/BECN1/CAV1/FBXW7/HIF1A/HMOX1/IL33/IL6/MAPK3/MAPK9/MTDH/NFE2L2/PRKAA2/SIRT1/TNFAIP3/TRIB3/ULK1/ZFP36
AKR1C3/BECN1/CAV1/CDKN1A/CRYAB/EGFR/GCH1/HIF1A/NFE2L2/NOX4/PTGS2/STAT3/TLR4/TP53
ATF3/BACH1/CA9/HIF1A/HMOX1/HSPA5/JUN/MUC1/NFE2L2/TP53/UBC/VEGFA
BECN1/CDKN1A/CRYAB/CYBB/DRD4/DUOX1/GCH1/HAMP/HMOX1/IL6/JUN/MAP3K5/MAPK3/MT1G/NFE2L2/PTGS2/RGS4/SIRT1/STAT3/TNFAIP3/TXNIP/TXNRD1
ATF3/BECN1/CAV1/CD44/FBXW7/HIF1A/HMOX1/MAPK8/MAPK9/MUC1/NFE2L2/PTGS2/RB1/SIRT1/TGFBR1/TNFAIP3/TP53/TP63/XBP1
ATG7/AURKA/BECN1/CAV1/FBXW7/HIF1A/HMOX1/IL33/IL6/MAPK3/MAPK9/MTDH/NFE2L2/PRKAA2/SIRT1/TNFAIP3/TRIB3/ULK1/ZFP36
ATF3/HIF1A/HSPA5/MUC1/NFE2L2/TP53/VEGFA
AKR1C3/BECN1/EGFR/GCH1/IL6/JUN/MAP3K5/MAPK3/MAPK8/MAPK9/NFE2L2/SIRT1/TNFAIP3
ATG7/BACH1/BECN1/CA9/CAV1/CDKN1A/CRYAB/CYBB/DDIT4/DPP4/HIF1A/HMOX1/NFE2L2/NOX4/PTGS2/SIRT1/TP53/UBC/VEGFA
CAV1/EGFR/FBXW7/HIF1A/HMOX1/HRAS/JUN/NRAS/RB1/SIRT1/STAT3/TGFBR1/TNFAIP3/TP63/VEGFA/XBP1/ZFP36
BECN1/CRYAB/CYBB/DRD4/HAMP/HMOX1/HSPA5/IL6/JUN/MAP3K5/NFE2L2/RGS4/SIRT1/STAT3/TNFAIP3/TP53/TXNIP
EGFR/HIF1A/HMOX1/HRAS/JUN/NRAS/SIRT1/STAT3/TGFBR1/TNFAIP3/TP63/VEGFA/XBP1
ATG7/DDIT4/EIF2S1/FBXW7/HIF1A/HMOX1/HRAS/HSPA5/JUN/MAP3K5/PIK3CA/RB1/SIRT1/STAT3/TLR4/TP53/TP63
FTH1/FTL/HAMP/HIF1A/HMOX1/STEAP3/TF/TFR2/TFRC
ATG7/BECN1/CYBB/DDIT4/EGFR/HMOX1/HSPA5/IL6/MAP3K5/MAPK3/NFE2L2/PRKAA2/PTGS2/SIRT1/TFRC/TNFAIP3/TP53
AKR1C1/AKR1C2/AKR1C3/CHAC1/CYBB/DDIT4/DUOX1/EGFR/GCH1/GOT1/HIF1A/HMOX1/IDH1/NFE2L2/NNMT/PGD/PRKAA2/PTGS2/SLC2A6/STAT3/TP53
BACH1/BECN1/CA9/CAV1/CRYAB/CYBB/DDIT4/DPP4/HIF1A/HMOX1/NFE2L2/NOX4/PTGS2/SIRT1/TP53/UBC/VEGFA
FTH1/FTL/HAMP/HIF1A/HMOX1/TF/TFR2/TFRC
ALOX12/CAV1/DUOX1/EGFR/ENPP2/HIF1A/HMOX1/HRAS/HSPA5/IL6/JUN/MAPK3/NFE2L2/NOX4/PTGS2/SIRT1/STAT3/TGFBR1/VEGFA/XBP1
ATF3/BECN1/CDKN1A/EIF2S1/MAP3K5/MAPK3/MAPK8
BACH1/BECN1/CA9/CAV1/CRYAB/CYBB/DDIT4/DPP4/HIF1A/HMOX1/NFE2L2/NOX4/PTGS2/SIRT1/TP53/UBC/VEGFA
ATP6V1G2/FTH1/FTL/HAMP/STEAP3/TF/TFR2/TFRC
CAV1/EGFR/FBXW7/HIF1A/HMOX1/HRAS/JUN/NRAS/RB1/SIRT1/STAT3/TGFBR1/TNFAIP3/TP63/VEGFA/XBP1/ZFP36
AKR1C3/ALOX12/ALOX15B/ALOX5/DDIT4/FADS2/GCH1/GLS2/GOT1/HIF1A/PRKAA2/PTGS2/SIRT1/SLC2A6/STAT3/TP53/TRIB3/XBP1
ALOX12/CAV1/DUOX1/EGFR/ENPP2/HIF1A/HMOX1/HRAS/HSPA5/IL6/JUN/MAPK3/NFE2L2/NOX4/PTGS2/SIRT1/STAT3/TGFBR1/VEGFA/XBP1
AKR1C3/ALOX12/ALOX15B/ALOX5/DDIT4/FADS2/GCH1/GLS2/GOT1/HIF1A/PRKAA2/PTGS2/SIRT1/SLC2A6/STAT3/TP53/TRIB3/XBP1
AURKA/CAV1/CD44/DDIT4/EGFR/IL6/MAPK3/MAPK8/MAPK9/PIK3CA/PRKAA2/PTGS2/TGFBR1/ULK1/VEGFA
ATF3/BECN1/CDKN1A/EIF2S1/MAP3K5/MAPK3/MAPK8
CAV1/CDKN1A/CDKN2A/DRD4/EGFR/GDF15/HRAS/MAP3K5/MAPK3/NOX4/RB1/RGS4/SIRT1/TGFBR1/TLR4/TNFAIP3/TRIB3/UBC/VEGFA
ALOX12/CAV1/DUOX1/EGFR/ENPP2/HIF1A/HMOX1/HRAS/HSPA5/IL6/JUN/MAPK3/NFE2L2/NOX4/PTGS2/SIRT1/STAT3/TGFBR1/VEGFA/XBP1
FTH1/FTL/HAMP/HIF1A/HMOX1/MT1G/STEAP3/TF/TFR2/TFRC
ALOX12/CAV1/EGFR/ENPP2/HIF1A/HMOX1/HRAS/HSPA5/IL6/JUN/MAPK3/NFE2L2/NOX4/PTGS2/SIRT1/STAT3/TGFBR1/VEGFA/XBP1
CD44/CDKN1A/DDIT4/MUC1/SIRT1/TP53/TP63
CAV1/CYBB/ENPP2/FBXW7/HIF1A/HMOX1/IL6/JUN/MTDH/NFE2L2/PIK3CA/PTGS2/SAT1/SIRT1/STAT3/TGFBR1/TNFAIP3/VEGFA/XBP1
AURKA/CAV1/CD44/DDIT4/EGFR/IL6/MAPK3/MAPK8/MAPK9/PIK3CA/PRKAA2/PTGS2/TGFBR1/ULK1/VEGFA
BECN1/CAV1/CD44/FBXW7/HIF1A/MUC1/NFE2L2/PTGS2/SIRT1/TP53/XBP1
ALOX12/CYBB/DUOX1/EGFR/GCH1/NCF2/NFE2L2/NOX4
ATP6V1G2/BECN1/HIF1A/HMOX1/MAPK3/MAPK8/PIK3CA/PRKAA2/SIRT1/TP53/ULK1
ALOX12/CAV1/EGFR/HAMP/IL6/LPIN1/NOX4/PIK3CA/PRKAA2/PTGS2/RB1/STAT3/TF/TFRC/TLR4/TNFAIP3/TP63/VEGFA
ATG7/BACH1/CA9/CAV1/CYBB/HIF1A/HMOX1/NFE2L2/PTGS2/SIRT1/TP53/UBC/VEGFA
FTH1/FTL/HAMP/HIF1A/HMOX1/MT1G/TF/TFR2/TFRC
ATF3/EIF2S1/FBXW7/HSPA5/NFE2L2/TP53/XBP1
AKR1C2/AKR1C3/ALOX12/ALOX15B/ALOX5/FADS2/MAPK3/PTGS2/SIRT1
AKR1C3/ALOX12/ALOX15B/ALOX5/DDIT4/FADS2/HIF1A/PRKAA2/PTGS2/SIRT1/SLC2A6/STAT3/TP53/TRIB3/XBP1
CDKN1A/CRYAB/HAMP/HRAS/HSPA5/NOX4/SIRT1/TP53
CDKN1A/CRYAB/EGFR/EIF2S1/GOT1/HAMP/HRAS/HSPA5/MAPK3/MAPK8/NOX4/PTGS2/SIRT1/TLR4/TP53
CDKN1A/CRYAB/EGFR/EIF2S1/GOT1/HAMP/HRAS/HSPA5/MAPK3/MAPK8/NOX4/PTGS2/SIRT1/TLR4/TP53
AKR1C3/ALOX12/ALOX15B/ALOX5/FADS2/PTGS2/SIRT1
ATG7/BECN1/FBXW7/GABARAPL1/HIF1A/SQSTM1/TP53/WIPI1
ATG7/BECN1/FBXW7/GABARAPL1/HIF1A/SQSTM1/TP53/WIPI1
CYBB/ENPP2/FBXW7/HIF1A/HMOX1/IL6/MTDH/NFE2L2/PTGS2/SIRT1/STAT3/TNFAIP3/VEGFA/XBP1
CAV1/CDKN1A/CDKN2A/CHMP6/NF2/RB1/RGS4/SIRT1/SOCS1/TNFAIP3/TP53/TRIB3/ZFP36
CAV1/CD44/CHMP6/EGFR/ENPP2/FBXW7/IL6/NF2/NOX4/SOCS1/STAT3/TP53/VEGFA
ALOX12/CAV1/CD44/CDKN1A/DUOX1/EGFR/HIF1A/HMOX1/HRAS/IL6/LOX/MAP3K5/MAPK3/NFE2L2/PIK3CA/TGFBR1/TLR4/TNFAIP3/XBP1
CDKN1A/CRYAB/HRAS/HSPA5/NOX4/TP53
AKR1C3/EIF2S1/HMOX1/IL6/MAP3K5/NFE2L2/RB1/TNFAIP3/ZFP36
AKR1C3/CAV1/CDKN1A/CYBB/DDIT4/EGFR/GOT1/IDH1/IL6/LOX/PTGS2/RB1/SIRT1/TP63/TXNIP/ZFP36
CAV1/HIF1A/HMOX1/JUN/NRAS/SIRT1/STAT3/TGFBR1/VEGFA/XBP1
BECN1/HAMP/HIF1A/HMOX1/TF/TFR2
BECN1/CRYAB/HMOX1/IL6/JUN/MAP3K5/NFE2L2/SIRT1/TNFAIP3/TXNIP
CAV1/CDKN2A/HMOX1/IL6/JUN/MAPK3/MAPK8/MAPK9/MTDH/RB1/SIRT1/STAT3/TLR4/TNFAIP3/UBC/VEGFA
ATF3/CAV1/CHAC1/EIF2S1/HSPA5/JUN/MAP3K5/NFE2L2/SIRT1/TP53/TRIB3/WIPI1/XBP1
AKR1C3/ALOX12/ALOX15B/ALOX5/FADS2/PRKAA2/PTGS2/SIRT1/TRIB3/XBP1
ATG7/DDIT4/EIF2S1/FBXW7/HIF1A/HMOX1/HRAS/JUN/MAP3K5/PIK3CA/SIRT1/STAT3/TLR4/TP53
ALOX12/HIF1A/HMOX1/NFE2L2/PTGS2/SIRT1/VEGFA

HAMP/HSPA5/IL6/MAPK3/MAPK8/MTDH/TLR4/TNFAIP3/TP53/TXNIP/XBP1/ZFP36
BECN1/CYBB/DUOX1/GCH1/HMOX1/IL6/MAP3K5/NFE2L2/PTGS2/SIRT1/TNFAIP3/TXNRD1
BECN1/HSPA5/NFE2L2/PRKAA2/TP53/XBP1
ALOX12/CYBB/DUOX1/EGFR/NCF2/NOX4
ATF3/CAV1/CDKN1A/CDKN2A/CHMP6/DDIT4/JUN/NF2/RB1/RGS4/SIRT1/SOCS1/STAT3/TLR4/TNFAIP3/TRIB3
CYBB/ENPP2/FBXW7/HIF1A/HMOX1/IL6/MTDH/NFE2L2/PTGS2/SIRT1/STAT3/TNFAIP3/VEGFA/XBP1
BECN1/HIF1A/HMOX1/MAPK3/PRKAA2/SIRT1/ULK1
AKR1C2/AKR1C3/CA9/CAV1/CDKN1A/CYBB/DDIT4/EGFR/PRKAA2/SIRT1/TXNIP
HIF1A/NFE2L2/TP53/VEGFA/XBP1
ATF3/CAV1/CDKN1A/CDKN2A/CHMP6/DDIT4/JUN/NF2/RB1/RGS4/SIRT1/SOCS1/TLR4/TNFAIP3/TRIB3
ATF3/CAV1/CD44/DDIT4/HIF1A/MUC1/NF2/NFE2L2/PRKAA2/PTGS2/RGS4/SIRT1/SOCS1/TLR4/TNFAIP3/TRIB3/XBP1
CAV1/DRD4/EGFR/GDF15/HRAS/MAP3K5/MAPK3/NOX4/RGS4/TGFBR1/TLR4/TRIB3/UBC/VEGFA
HIF1A/HMOX1/JUN/NRAS/SIRT1/STAT3/TGFBR1/VEGFA
ALOX12/DPP4/ENPP2/FBXW7/HIF1A/HMOX1/JUN/NFE2L2/PIK3CA/PTGS2/SIRT1/TGFBR1/VEGFA
ALOX12/CDKN1A/EGFR/HMOX1/IL6/JUN/MAP3K5/PTGS2/STAT3/TGFBR1/TNFAIP3
CAV1/HIF1A/HMOX1/JUN/NRAS/SIRT1/STAT3/TGFBR1/VEGFA
AKR1C2/AKR1C3/ALOX12/ALOX15B/ALOX5/CAV1/FADS2/LPIN1/MAPK3/PRKAA2/PTGS2/SIRT1/TRIB3/XBP1
ALOX12/DPP4/ENPP2/FBXW7/HIF1A/HMOX1/JUN/NFE2L2/PIK3CA/PTGS2/SIRT1/TGFBR1/VEGFA
AKR1C2/AKR1C3/ALOX12/ALOX15B/ALOX5/MAPK3/PTGS2/SIRT1
ALOX12/FBXW7/HIF1A/HMOX1/NFE2L2/PTGS2/SIRT1/VEGFA
CYBB/HIF1A/HMOX1/MTDH/NFE2L2/PTGS2/SIRT1/STAT3/VEGFA/XBP1
ALOX12/CDKN2A/HMOX1/MAP3K5/NFE2L2/SIRT1/TP53
ATG7/BECN1/FBXW7/GABARAPL1/HIF1A/SQSTM1/TP53/WIPI1
BACH1/CA9/CYBB/HIF1A/HMOX1/NFE2L2/PTGS2/SIRT1/TP53/UBC/VEGFA
CDKN1A/CRYAB/EIF2S1/HMOX1/HSBP1/HSPA5/MAPK3/PTGS2/SIRT1
AKR1C3/CDKN1A/EGFR/NFE2L2/NOX4/PTGS2/TLR4/TP53
ALOX12/DPP4/FBXW7/HIF1A/HMOX1/NFE2L2/PIK3CA/PTGS2/SIRT1/TGFBR1/VEGFA
ALOX12/DPP4/ENPP2/FBXW7/HIF1A/HMOX1/JUN/NFE2L2/PIK3CA/PTGS2/SIRT1/TGFBR1/VEGFA
ALOX15B/AURKA/CAV1/EGFR/FBXW7/HAMP/HIF1A/HMOX1/JUN/MAPK3/PIK3CA/TGFBR1/TNFAIP3/TP63/VEGFA/XBP1
CAV1/CDKN1A/CDKN2A/CHMP6/NF2/RB1/RGS4/SIRT1/SOCS1/TNFAIP3/TRIB3
ATG7/BECN1/CAPG/CAV1/CD44/DDIT4/DPP4/FBXW7/GABARAPL1/HIF1A/IL6/LAMP2/LPIN1/SQSTM1/TGFBR1/TP53/WIPI1
ATP6V1G2/FTH1/FTL/HAMP/STEAP3/TF/TFR2/TFRC
ATF3/CAV1/CDKN1A/CDKN2A/CHMP6/DDIT4/JUN/NF2/RB1/RGS4/SIRT1/SOCS1/STAT3/TLR4/TNFAIP3/TP53/TRIB3
ALOX12/CDKN2A/HMOX1/MAP3K5/NFE2L2/SIRT1/TP53
ATF3/CAV1/CDKN1A/CDKN2A/CHMP6/DDIT4/JUN/NF2/RB1/RGS4/SIRT1/SOCS1/STAT3/TLR4/TNFAIP3/TP53/TRIB3
BACH1/CA9/CYBB/HIF1A/HMOX1/NFE2L2/PTGS2/SIRT1/TP53/UBC/VEGFA
CD44/HIF1A/HMOX1/MUC1/NFE2L2/PTGS2/RB1/SIRT1/TGFBR1/TNFAIP3/XBP1
ATP6V1G2/CAV1/CYBB/GDF15/GOT1/LPIN1/NFE2L2/PIK3CA/PTGS2/SIRT1/SOCS1/STAT3/TLR4/TNFAIP3/TP53/TRIB3/XBP1
CD44/CDKN1A/DDIT4/HMOX1/MUC1/SIRT1/TP53/TP63
CAV1/GOT1/HSPA5/LOX/SIRT1/TGFBR1/TP53/UBC
ATF3/BECN1/CAV1/FBXW7/MAPK8/MAPK9/SIRT1/TGFBR1/TP53/TP63
AURKA/EGFR/FBXW7/HAMP/HMOX1/JUN/PIK3CA/TNFAIP3/XBP1
CDKN1A/DRD4/EGFR/FBXW7/GDF15/HRAS/MAP3K5/MAPK3/NOX4/PIK3CA/SIRT1/SOCS1/TGFBR1/TLR4/UBC/VEGFA/VLDLR
CAV1/CD44/CHMP6/EGFR/ENPP2/FBXW7/IL6/MAPK3/NF2/NOX4/SOCS1/STAT3/TP53/VEGFA
CAV1/GOT1/HSPA5/LOX/SIRT1/TGFBR1/TP53/UBC
CAV1/CD44/CHMP6/EGFR/ENPP2/FBXW7/IL6/MAPK3/NF2/NOX4/SOCS1/STAT3/TP53/VEGFA
CHAC1/CYBB/DDIT4/DUOX1/GCH1/HIF1A/NFE2L2/NNMT/PRKAA2/PTGS2/SLC2A6/STAT3/TP53
BECN1/HIF1A/HMOX1/MAPK3/MTDH/PRKAA2/SIRT1/ULK1
ALOX12/CDKN1A/EGFR/HMOX1/IL6/JUN/MAP3K5/PTGS2/TNFAIP3
AURKA/EGFR/FBXW7/HAMP/HMOX1/JUN/PIK3CA/TNFAIP3/XBP1
CD44/CDKN1A/DDIT4/MUC1/SIRT1/TP53/TP63
BECN1/CYBB/HSPA5/IL6/MAP3K5/NFE2L2/SIRT1/TNFAIP3/TP53
CDKN1A/CRYAB/EIF2S1/HAMP/HRAS/HSPA5/NOX4/PTGS2/SIRT1/TP53
AURKA/CDKN1A/EGFR/HAMP/HMOX1/LPIN1/NNMT
ALOX12/CDKN1A/EGFR/HMOX1/IL6/JUN/MAP3K5/PTGS2/TNFAIP3
ALOX12/CAV1/CDKN1A/DUOX1/HRAS/NFE2L2/TLR4/TNFAIP3/XBP1
CYBB/HIF1A/HMOX1/MTDH/NFE2L2/PTGS2/SIRT1/STAT3/VEGFA/XBP1
CAV1/GDF15/GOT1/HSPA5/JUN/LOX/SIRT1/TGFBR1/TP53/UBC
DDIT4/HIF1A/IDH1/NNMT/PGD/PRKAA2/PTGS2/SLC2A6/STAT3/TP53
DDIT4/HIF1A/IDH1/NNMT/PGD/PRKAA2/PTGS2/SLC2A6/STAT3/TP53
CAV1/CDKN1A/CDKN2A/CHMP6/NF2/RB1/RGS4/SIRT1/SOCS1/TNFAIP3/TRIB3
ALOX12/ENPP2/HIF1A/HMOX1/JUN/NFE2L2/PTGS2/SIRT1/VEGFA
ATG7/FBXW7/GCH1/HIF1A/NFE2L2/SIRT1/TLR4
GOT1/PRKAA2/SIRT1/XBP1
CAV1/CYBB/DUOX1/GCH1/NOX4/PTGS2/STAT3/TLR4
AKR1C3/EIF2S1/HMOX1/IL6/NFE2L2/TNFAIP3/ZFP36
ATP6V1G2/CAV1/GDF15/GOT1/LPIN1/NFE2L2/PIK3CA/SIRT1/SOCS1/STAT3/TLR4/TP53/TRIB3/XBP1
DDIT4/HIF1A/IDH1/NNMT/PGD/PRKAA2/PTGS2/SLC2A6/STAT3/TP53
BACH1/CA9/HIF1A/NFE2L2/TP53/UBC/VEGFA
CAV1/GDF15/GOT1/HSPA5/JUN/LOX/NOX4/SIRT1/TGFBR1/TP53/UBC
ATG7/FBXW7/HIF1A/MAP3K5/NFE2L2/SIRT1/TLR4
ATG7/FBXW7/GCH1/HIF1A/NFE2L2/SIRT1/TLR4
ALOX15B/HIF1A/HMOX1/IL33/IL6/TLR4
AURKA/CAV1/FBXW7/IL33/MAPK9/NFE2L2/TRIB3
CAV1/GDF15/GOT1/HSPA5/JUN/LOX/NOX4/SIRT1/TGFBR1/TP53/UBC
CDKN1A/DRD4/EGFR/FBXW7/GDF15/HRAS/MAP3K5/MAPK3/NOX4/PIK3CA/SIRT1/SOCS1/TGFBR1/TLR4/UBC/VEGFA/VLDLR
ALOX15B/CDKN1A/CDKN2A/CRYAB/GDF15/HIF1A/MT1G/RGS4/SIRT1/TP53/ULK2
ALOX15B/EGFR/FBXW7/HIF1A/HRAS/IL33/IL6/MAPK3/MAPK8/PANX1/PTGS2/TLR4/TP53/TP63/XBP1
ALOX12/FBXW7/HIF1A/HMOX1/NFE2L2/PTGS2/SIRT1/VEGFA
BECN1/CAV1/CRYAB/HIF1A/STAT3/TP53
AURKA/CAV1/EIF2S1/FBXW7/HMOX1/HSPA5/JUN/MAPK3/MAPK8
DDIT4/HIF1A/IDH1/NNMT/PGD/PRKAA2/PTGS2/SLC2A6/STAT3/TP53
ATP6V1G2/STEAP3/TF/TFR2/TFRC
CHAC1/MAP3K5/SIRT1/TP53/TRIB3/XBP1
AKR1C2/AKR1C3/EGFR/GDF15/LOX/MTDH/NOX4/PIK3CA/SIRT1/TGFBR1/TRIB3
CDKN1A/CRYAB/HRAS/HSPA5/NOX4/TP53
AURKA/CAV1/FBXW7/IL33/MAPK9/NFE2L2/TRIB3
ATP6V1G2/STEAP3/TF/TFR2/TFRC
ATP6V1G2/STEAP3/TF/TFR2/TFRC
ALOX12/ALOX15B/ALOX5/PTGS2
AKR1C3/CDKN1A/CYBB/DDIT4/EGFR/GOT1/IL6/PTGS2/ZFP36
AURKA/CAV1/FBXW7/IL33/MAPK9/NFE2L2/TNFAIP3/TRIB3
ALOX12/EGFR/HMOX1/IL6/JUN/MAP3K5/PTGS2
CD44/HIF1A/MUC1/NFE2L2/PTGS2/SIRT1/XBP1
AKR1C1/AKR1C2/AKR1C3/CYBB/EGFR/HAMP/PRKAA2/SOCS1/VEGFA/XBP1
CDKN1A/CRYAB/EIF2S1/HMOX1/HSBP1/HSPA5/MAPK3/PTGS2/SIRT1
DRD4/EGFR/GDF15/HRAS/MAP3K5/MAPK3/NOX4/TGFBR1/TLR4/UBC/VEGFA
ALOX12/CAV1/CDKN1A/DUOX1/HRAS/NFE2L2/TLR4/TNFAIP3/XBP1
AKR1C3/ALOX12/ALOX15B/ALOX5/FADS2/MAPK3/PTGS2
ATG7/DRD4/EGFR/FBXW7/JUN/MAPK8/MAPK9/PRKAA2/SIRT1/TP53
ATF3/CAV1/CDKN1A/CDKN2A/CHMP6/DDIT4/JUN/NF2/PRKAA2/RB1/RGS4/SIRT1/SOCS1/TLR4/TNFAIP3/TRIB3
ATP6V1G2/CAV1/GDF15/GOT1/LPIN1/NFE2L2/PIK3CA/SIRT1/SOCS1/STAT3/TRIB3/XBP1
AURKA/CAV1/EIF2S1/FBXW7/HMOX1/HSPA5/JUN/LOX/MAPK3/MAPK8/RB1/TGFBR1/TRIB3
CAV1/HSPA5/SIRT1/TGFBR1/TP53/UBC
ATG7/FBXW7/HIF1A/NFE2L2/SIRT1/TLR4

CAV1/CD44/DDIT4/EGFR/IL6/PIK3CA/PTGS2/VEGFA
CAV1/HSPA5/SIRT1/TGFBR1/TP53/UBC
ALOX12/HIF1A/HMOX1/NFE2L2/PTGS2/SIRT1/VEGFA
DDIT4/HIF1A/PRKAA2/SLC2A6/STAT3/TAZ/TP53
GCH1/HAMP/IL6/JUN/MAPK3/MAPK8/MTDH/PTGS2/TLR4/TNFAIP3/XBP1/ZFP36
AKR1C2/AKR1C3/EGFR/GDF15/MTDH/NOX4/PIK3CA/SIRT1/TGFBR1/TRIB3
CDKN1A/CRYAB/EGFR/EIF2S1/HAMP/HIF1A/HRAS/HSPA5/JUN/MAPK8/NOX4/PTGS2/SIRT1/TP53
AKR1C1/AKR1C2/AKR1C3/CYBB/DUOX1/EGFR/IDH1/STAT3
DDIT4/EIF2S1/FBXW7/JUN/MAP3K5/TLR4/TP53
AKR1C1/AKR1C2/AKR1C3/DRD4/ENPP2/GCH1/LPCAT3/LPIN1/SAT1
ATF3/EIF2S1/HSPA5/NFE2L2
EGFR/STAT3/TP53/ZFP36
EGFR/STAT3/TP53/ZFP36
ALOX12/ENPP2/FBXW7/HIF1A/HMOX1/JUN/NFE2L2/PTGS2/SIRT1/VEGFA
AURKA/CAV1/IL33/MAPK9/NFE2L2/TRIB3
ATP6V1G2/CAV1/CYBB/GDF15/GOT1/LPIN1/NFE2L2/PIK3CA/PTGS2/SIRT1/SOCS1/STAT3/TRIB3/XBP1
CRYAB/EGFR/HRAS/MAP3K5/MAPK3/MAPK8/MAPK9/TLR4/UBC/VEGFA/ZFP36
DRD4/EGFR/GDF15/HRAS/MAP3K5/MAPK3/NOX4/SIRT1/TGFBR1/TLR4/UBC/VEGFA
CDKN1A/CDKN2A/HRAS/NOX4/SIRT1/TP53/TP63
CAV1/CD44/EGFR/IL6/PIK3CA/PTGS2/VEGFA
AKR1C3/CAV1/FBXW7/IDH1/PLIN2/PRKAA2/PTGS2/RB1/SIRT1/SOCS1/TAZ/TRIB3/TXNRD1
DDIT4/HIF1A/NNMT/PRKAA2/PTGS2/SLC2A6/STAT3/TP53
DDIT4/HIF1A/NNMT/PRKAA2/PTGS2/SLC2A6/STAT3/TP53
AKR1C2/AKR1C3/ALOX12/ALOX15B/ALOX5/MAPK3/PTGS2/SIRT1
GCH1/HAMP/IL6/JUN/MAPK3/MAPK8/MTDH/PTGS2/TLR4/TNFAIP3/XBP1/ZFP36
HAMP/IL6/PTGS2/STAT3/TFR2
FBXW7/HIF1A/MAP3K5/NFE2L2/SIRT1
AKR1C3/ALOX12/ALOX5/PTGS2/SIRT1
CDKN1A/CRYAB/HAMP/HRAS/HSPA5/NOX4/SIRT1/TP53
ATG7/CDKN1A/EGFR/FBXW7/HRAS/IL6/JUN/MAPK3/NOX4/PIK3CA/SIRT1/TFRC/TP53
AURKA/CAV1/FBXW7/IL33/MAPK9/NFE2L2/TRIB3
AKR1C3/ALOX12/ALOX15B/MAPK3/PTGS2
DDIT4/HIF1A/NNMT/PRKAA2/PTGS2/SLC2A6/STAT3/TP53
ALOX12/DPP4/ENPP2/FBXW7/HIF1A/HMOX1/JUN/NFE2L2/PIK3CA/PTGS2/SIRT1/TGFBR1/VEGFA
AURKA/CDKN1A/EGFR/HAMP/HMOX1/JUN/LPIN1/NNMT/ULK1
ALOX12/AURKA/CAV1/FBXW7/IL33/MAP3K5/MAPK3/MAPK9/NFE2L2/SIRT1/STAT3/TRIB3
CAV1/MAP3K5/PANX1/SQSTM1/TP53
CD44/DRD4/EGFR/FBXW7/GDF15/HRAS/IL6/JUN/MAP3K5/MAPK3/NOX4/TGFBR1/TLR4/UBC/VEGFA
EGFR/HIF1A/IL6/LAMP2/LOX/MAPK3/NOX4/RB1/TF/TFRC/TLR4/TNFAIP3/VEGFA
FBXW7/HIF1A/HMOX1/HRAS/HSPA5/JUN/PIK3CA/RB1/TP53/TP63
AKR1C2/AKR1C3/CDKN1A/CYBB/DRD4/HAMP/PRKAA2/RGS4/STAT3/TGFBR1
ATG7/BECN1/FBXW7/GABARAPL1/GABPB1/HIF1A/JUN/MAPK8/SQSTM1/STAT3/TAZ/TP53/TP63/WIPI1
AURKA/CDKN1A/CDKN2A/CRYAB/EGFR/HAMP/RB1/RGS4/SIRT1/TGFBR1/TP53/ULK1/ULK2/VEGFA
HAMP/IL6/MAPK3/MAPK8/MTDH/TLR4/TNFAIP3/XBP1/ZFP36
ATG7/AURKA/CAV1/FBXW7/IL33/MAPK9/NFE2L2/TNFAIP3/TRIB3
CISD1/DDIT4/HIF1A/PIK3CA/PRKAA2/SLC2A6/STAT3/TP53
ATG7/BECN1/HMOX1/PIK3CA/STAT3/TP53
AKR1C1/AKR1C2/AKR1C3/ATG7/CYBB/EGFR/HAMP/PRKAA2/PTGS2/SOCS1/VEGFA/XBP1
CAV1/GOT1/HIF1A/HSPA5/LOX/SIRT1/TGFBR1/TP53/UBC/VEGFA
ATG7/BECN1/GABARAPL1/ULK1/ULK2/WIPI1
ALOX15B/HIF1A/HMOX1/IL33/IL6/TLR4
ALOX15B/EGFR/FBXW7/HIF1A/HRAS/IL33/IL6/MAPK3/PANX1/PTGS2/TLR4/TP53/XBP1
AURKA/CDKN1A/CDKN2A/EGFR/FBXW7/MUC1/RB1/TP53
ATG7/DRD4/FBXW7/MAPK8/MAPK9/PRKAA2/TP53
DDIT4/HIF1A/NFE2L2/PRKAA2/SLC2A6/STAT3/TP53
ALOX15B/DPP4/DRD4/EGFR/HIF1A/IL33/IL6/MAPK3/PANX1/SOCS1/TFR2/TLR4/TNFAIP3/XBP1
HAMP/IL6/MAPK3/MAPK8/MTDH/TLR4/TNFAIP3/XBP1/ZFP36
ATG7/FBXW7/HIF1A/TLR4
CAV1/CYBB/NFE2L2/PTGS2
ALOX12/FBXW7/HIF1A/HMOX1/NFE2L2/PTGS2/SIRT1/VEGFA
EGFR/IL6/NOX4/RB1/TF/TFRC/TLR4/TNFAIP3/VEGFA
ATG7/BECN1/GABARAPL1/ULK1/ULK2/WIPI1
CRYAB/EGFR/HRAS/MAP3K5/MAPK3/MAPK8/MAPK9/TLR4/UBC/VEGFA/ZFP36
AURKA/BACH1/CDKN1A/CDKN2A/EGFR/FBXW7/MUC1/RB1/RRM2/TP53
DDIT4/HIF1A/PRKAA2/SLC2A6/STAT3/TAZ/TP53
AKR1C1/CAV1/FBXW7/GOT1/PRKAA2/SIRT1/XBP1
DDIT4/HIF1A/PRKAA2/SLC2A6/STAT3/TAZ/TP53
ATG7/DRD4/EGFR/FBXW7/HSPA5/JUN/MAPK8/MAPK9/PRKAA2/SIRT1/TP53
EGFR/GOT1/MAPK3/MAPK8/PTGS2/TLR4
ATG7/FBXW7/HIF1A/TLR4
FBXW7/HIF1A/NFE2L2/SIRT1
CD44/MUC1/SIRT1/TP53
ATG7/BECN1/GABARAPL1
AKR1C1/AKR1C2/AKR1C3
ATG7/BECN1/GABARAPL1
ATF3/HSPA5/TP53
DDIT4/HIF1A/PRKAA2/SLC2A6/STAT3/TP53
DDIT4/HIF1A/PRKAA2/SLC2A6/STAT3/TAZ/TP53
AKR1C3/ATG7/CAV1/DDIT4/EGFR/HAMP/HSPA5/MAPK3/NOX4/PTGS2/RB1/SIRT1/STAT3/TP63/ZFP36
DDIT4/HIF1A/PRKAA2/SLC2A6/STAT3/TP53
ALOX15B/HIF1A/HMOX1/IL33/IL6/TLR4
CAV1/GDF15/GOT1/HSPA5/JUN/LOX/MAPK3/SIRT1/TGFBR1/TP53/UBC
ALOX12/ALOX15B/ALOX5/PTGS2
CAV1/CDKN1A/CDKN2A/RB1/RGS4/SIRT1/TNFAIP3
EGFR/GOT1/JUN/MAPK3/MAPK8/MAPK9/PTGS2/TLR4/TXNIP
AURKA/CAV1/EIF2S1/HSPA5/LOX/MAPK3/MAPK8/TGFBR1/TRIB3
AKR1C2/AKR1C3/EGFR/GDF15/MTDH/NOX4/PIK3CA/TGFBR1
FBXW7/HIF1A/JUN/LOX/MT1G/RB1/SIRT1/STAT3/TFRC/TLR4/VEGFA/ZFP36
CAV1/GDF15/GOT1/HSPA5/LOX/SIRT1/TGFBR1/TP53/UBC
CISD1/CYBB/DDIT4/HIF1A/IDH1/NCF2/NOX4/PIK3CA/PRKAA2/SLC2A6/STAT3/TAZ/TP53/TXNRD1
BECN1/HMOX1/PIK3CA/TP53
AURKA/EGFR/HAMP/HMOX1
DUOX1/HRAS/NFE2L2/TLR4/XBP1
ALOX15B/CYBB/HIF1A/HMOX1/HRAS/IL33/IL6/MAPK3/PANX1/PTGS2/STAT3/TLR4/XBP1
CAV1/NF2/SOCS1
IL33/IL6/XBP1
CAV1/EGFR/HIF1A/IL6/NOX4/TFRC/TNFAIP3/TP53
AKR1C1/CAV1/CRYAB/ELAVL1/GCH1/HMOX1/HRAS/PANX1/RRM2/SLC1A5/SQSTM1/TNFAIP3/TP53/TP63
BECN1/CAV1/FBXW7/SIRT1/TP53
RGS4/SLC1A5/SLC38A1/SLC3A2
HIF1A/JUN/STAT3/TP53
AKR1C2/AKR1C3/DDIT4/EGFR/PRKAA2/SIRT1
ALOX15B/DPP4/DRD4/EGFR/HIF1A/IL33/IL6/MAPK3/PANX1/SOCS1/TLR4/TNFAIP3/XBP1
AURKA/CDKN1A/CRYAB/FBXW7/LAMP2/NF2/SIRT1/TF/TP53/XBP1
CDKN1A/EGFR/EIF2S1/MAPK8/PTGS2/SIRT1/TP53

DDIT4/HIF1A/PRKAA2/SLC2A6/STAT3/TP53
ALOX15B/DPP4/DRD4/EGFR/HIF1A/IL33/IL6/MAPK3/PANX1/SOCS1/STEAP3/TFR2/TLR4/TNFAIP3/XBP1
ATG7/BECN1/DDIT4/EGFR/ELAVL1/HMOX1/PIK3CA/STAT3/TP53/ZFP36
IL33/IL6/MAPK3/TFRC/TLR4/XBP1
AURKA/CAV1/EIF2S1/HSPA5/MAPK3/MAPK8
BECN1/IL6/MAP3K5/NFE2L2/SIRT1/TNFAIP3
CYBB/DUOX1/STAT3
CDKN1A/CDKN2A/TP53
FBXW7/TNFAIP3/XBP1
AKR1C3/BECN1/CYBB/EGFR/HAMP/HMOX1/PANX1/PTGS2/XBP1
ATG7/GOT1/HIF1A/NOX4/PIK3CA/PRKAA2/PTGS2/TXNIP/XBP1
ALOX15B/EGFR/HIF1A/IL33/IL6/MAPK3/PANX1/TFR2/TLR4/XBP1
CDKN1A/FBXW7/HSPA5/IDH1/LAMP2/MAPK8/PTGS2/RPL8/STAT3/TNFAIP3/TP53/TP63/TXNIP/UBC
AURKA/CAV1/FBXW7/IL33/MAPK9/NFE2L2/TRIB3
AURKA/CDKN1A/CDKN2A/HRAS/MUC1/PRKAA2/RB1/TGFBR1/TP53
CAV1/FBXW7/IL6/PLIN2/SIRT1
DRD4/EGFR/GDF15/MAP3K5/MAPK3/PIK3CA/SOCS1/TGFBR1/TLR4/UBC/VEGFA
CDKN1A/CDKN2A/CRYAB/EGFR/HAMP/RB1/RGS4/SIRT1/TGFBR1/TP53/ULK2/VEGFA
AURKA/CAV1/EGFR/EIF2S1/JUN/MAPK3/ULK1/ULK2/VEGFA
DDIT4/HIF1A/PRKAA2/SIRT1
EGFR/IL6/NOX4/TFRC/TNFAIP3
AKR1C3/CAV1/EGFR/HSPA5/JUN/PRKAA2/TXNIP
AKR1C3/DDIT4/SIRT1/STAT3/TP53/TRIB3
CDKN1A/CDKN2A/HRAS/SIRT1/TP53
AKR1C1/DUOX1/HSPA5/JUN/NOX4/PANX1/PTGS2
CDKN1A/CRYAB/EIF2S1/HMOX1/HSBP1/HSPA5/MAPK3/PTGS2/SIRT1
CYBB/DUOX1/STAT3
SIRT1/TLR4/XBP1
CDKN2A/SIRT1/TP53
DDIT4/HIF1A/PRKAA2/SLC2A6/STAT3/TP53
CAV1/HSPA5/SIRT1/TGFBR1/TP53/UBC
ALOX15B/IL33/MAPK3/PANX1/SOCS1/TLR4/TNFAIP3/XBP1
EGFR/IL33/IL6/JUN/PTGS2
HMOX1/IL33/IL6/MAPK3/TFRC/TLR4/XBP1
AKR1C2/AKR1C3/PTGS2/SIRT1
AKR1C2/AKR1C3/PTGS2/SIRT1
CAV1/MAP3K5/TLR4/TP53
CAV1/NFE2L2/SIRT1/XBP1
DDIT4/HIF1A/PRKAA2/SLC2A6/STAT3/TAZ/TP53
CD44/ENPP2/FBXW7/IL6/NOX4/STAT3/TP53/VEGFA
ATG7/BECN1/DDIT4/ELAVL1/HMOX1/PIK3CA/STAT3/TP53/ZFP36
ATG7/EGFR/FBXW7/HRAS/JUN/TP53
AURKA/BACH1/CDKN1A/CDKN2A/EGFR/MUC1/RB1/RRM2/TP53
HIF1A/PRKAA2/STAT3/TAZ
AURKA/CDKN1A/CDKN2A/EGFR/MUC1/RB1/TP53
CDKN1A/HIF1A/XBP1
ALOX12/ALOX15B/FADS2
SIRT1/TLR4/XBP1
CD44/MUC1/SIRT1
CD44/MUC1/SIRT1
AURKA/CDKN1A/CDKN2A/MUC1/RB1/TP53
CDKN1A/EGFR/HAMP/HIF1A/JUN/LOX/MAPK3/NOX4/RB1/RGS4/TAZ/TGFBR1/TP53/VEGFA
DDIT4/HIF1A/PRKAA2/SLC2A6/STAT3/TAZ/TP53
CDKN1A/DDIT4/EGFR/GOT1/IL6/PTGS2/ZFP36
FBXW7/JUN/MT1G/RB1/SIRT1/TFRC/TLR4/VEGFA
CYBB/HMOX1/IL6/STAT3/TLR4
DUOX1/HRAS/NFE2L2/TLR4/XBP1
CYBB/HMOX1/IL6/STAT3/TLR4/ZFP36
AURKA/CDKN1A/CDKN2A/MUC1/RB1/TP53
ATG7/AURKA/CAV1/CHMP6/EGFR/FBXW7/IL33/MAPK9/NFE2L2/TNFAIP3/TRIB3
ATG7/HIF1A/NOX4/PIK3CA/PRKAA2/PTGS2/TXNIP/XBP1
ALOX15B/DPP4/DRD4/EGFR/HIF1A/IL33/IL6/MAPK3/PANX1/SOCS1/STEAP3/TLR4/TNFAIP3/XBP1
HIF1A/HMOX1/IL6/RB1/STAT3/VEGFA/ZFP36
CDKN1A/MUC1/TP53
EIF2S1/HSPA5/PTGS2
DDIT4/STAT3/TP53
HAMP/STEAP3/TF
CDKN1A/MUC1/TP53
DDIT4/STAT3/TP53
IL33/IL6/TFRC/XBP1
CAV1/EGFR/MAPK3/VEGFA
EGFR/IL6/TFRC/TNFAIP3
FBXW7/HMOX1/PTGS2/VEGFA
MAPK8/PRKAA2/TP53/VEGFA
ATF3/CAV1/CRYAB/HAMP/LOX/RB1/RGS4/SIRT1/TAZ/TGFBR1/XBP1
ATG7/HIF1A/NOX4/PIK3CA/PRKAA2/PTGS2/TXNIP/XBP1
CD44/EGFR/FBXW7/HRAS/JUN/MAPK3/NOX4/TLR4
AKR1C3/ALOX15B/EGFR/HIF1A/HSPA5/IDH1/MAPK3/PTGS2/SIRT1/TGFBR1/TP63/VEGFA
AURKA/CD44/CDKN1A/MUC1/SIRT1/TP53
CAV1/CDKN1A/DPP4/IL33/IL6/PIK3CA/SOCS1/TFRC/TLR4/XBP1
ATG7/HIF1A/HMOX1/HRAS/JUN/PIK3CA/SIRT1/STAT3
AURKA/CAV1/IL33/MAPK9/NFE2L2/TRIB3
IL6/PTGS2/SIRT1/TRIB3/XBP1/ZFP36
DUOX1/GCH1/MT1G/NFE2L2/PTGS2/TXNRD1
DDIT4/GCH1/HIF1A/NNMT/PRKAA2/PTGS2/SLC2A6/STAT3/TP53
AKR1C3/ALOX15B/EGFR/HIF1A/HSPA5/IDH1/MAPK3/PTGS2/SIRT1/TGFBR1/TP63/VEGFA
AKR1C1/AKR1C2/AKR1C3
FBXW7/TNFAIP3/XBP1
FBXW7/TNFAIP3/XBP1
CAV1/EGFR/GCH1/PTGS2/RGS4
FBXW7/HIF1A/JUN/LOX/RB1/STAT3/TLR4/ZFP36
ATP6V1G2/GOT1/LPIN1/PIK3CA/SIRT1/SOCS1/TRIB3/XBP1
FBXW7/IL6/JUN/MT1G/RB1/SIRT1/SOCS1/STAT3/TFRC/TLR4/TP53/VEGFA/XBP1
ALOX15B/EGFR/HIF1A/IL33/IL6/MAPK3/PANX1/TLR4/XBP1
AURKA/CD44/CDKN1A/DDIT4/MUC1/PRKAA2/SIRT1/TP53/TP63
CDKN1A/IL6/TFRC/TLR4/XBP1
CAV1/EGFR/GCH1/HIF1A
HIF1A/JUN/STAT3/TP53
CYBB/HMOX1/IL6/STAT3/TLR4/ZFP36
DDIT4/HIF1A/PRKAA2/SLC2A6/STAT3/TAZ/TP53
DDIT4/HIF1A/PIK3CA/PRKAA2/SIRT1
EGFR/IL6/TFRC/TNFAIP3/TP53
CDKN1A/EIF2S1/PTGS2/SIRT1/TP53
CAV1/HSPA5/NFE2L2/SIRT1/XBP1
HMOX1/IL33/IL6/MAPK3/SIRT1/TFRC/TLR4/XBP1

AKR1C1/AKR1C2/AKR1C3
FBXW7/TNFAIP3/XBP1
PIK3CA/TP53/TP63
CYBB/HMOX1/IL6/STAT3/TLR4/ZFP36
AKR1C3/CAV1/DDIT4/HIF1A/PRKAA2/PTGS2/SIRT1/SLC2A6/STAT3/TAZ/TP53/TRIB3
AKR1C1/AKR1C2/AKR1C3/ALOX12/DRD4/DUOX1/GCH1/GOT1/HIF1A/PRKAA2/SIRT1/TP53/VLDLR
CAV1/IL6/NF2/SOCS1/STAT3
CAV1/CDKN1A/DPP4/IL33/IL6/PIK3CA/SOCS1/TFRC/TLR4/XBP1
ALOX12/CD44/CRYAB/MAP3K5/PTGS2/SIRT1/TP63/VEGFA
DDIT4/GCH1/HIF1A/IDH1/NNMT/PGD/PRKAA2/PTGS2/SLC2A6/STAT3/TP53
ATF3/EIF2S1/HSPA5/NFE2L2/WIPI1/XBP1
AURKA/CAV1/FBXW7/HSPA5/IL33/MAPK9/NFE2L2/SIRT1/SQSTM1/TNFAIP3/TRIB3/UBC/XBP1
CAV1/CDKN1A/DPP4/IL6/PIK3CA/SOCS1/TFRC/TLR4/XBP1
ALOX15B/IL33/MAPK3/PANX1/SOCS1/TLR4/TNFAIP3/XBP1
CDKN1A/IL6/TFRC/TLR4/TNFAIP3/XBP1
EGFR/MAPK8/TP53/TP63
CAV1/MAP3K5/TLR4/TP53
BECN1/MAPK3/MUC1/RB1/SIRT1/TP53/VEGFA
EGFR/HRAS/MAPK8/SQSTM1/TP53/TP63
HIF1A/LAMP2/LOX
IL33/IL6/XBP1
HIF1A/NFE2L2/SIRT1
CAV1/IL6/NF2/SOCS1/STAT3
EGFR/IL6/MAPK3/STAT3/TLR4
AURKA/CAV1/FBXW7/IL33/MAPK9/NFE2L2/TRIB3
ALOX12/CAV1/CD44/DPP4/IL6/NF2/PIK3CA/SOCS1/TFRC/VEGFA/XBP1
AURKA/CAV1/FBXW7/HSPA5/IL33/MAPK9/NFE2L2/SIRT1/SQSTM1/TNFAIP3/TRIB3/UBC/XBP1
CAV1/CD44/DPP4/IL6/PIK3CA/SOCS1/TFRC/XBP1
DDIT4/HIF1A/PRKAA2/SLC2A6/STAT3/TP53
DDIT4/HIF1A/PRKAA2/SLC2A6/STAT3/TP53
HIF1A/HMOX1/RB1/STAT3/VEGFA/ZFP36
EGFR/ELAVL1/SIRT1/STAT3/TP53/ZFP36
HIF1A/PRKAA2/STAT3/TAZ
AKR1C3/FBXW7/SIRT1/TRIB3
BECN1/MAPK3/SQSTM1/TP53
HIF1A/PRKAA2/STAT3/TAZ
ATG7/HIF1A/NFE2L2/SIRT1
RGS4/SLC1A5/SLC38A1/SLC3A2
ATG7/HIF1A/NFE2L2/SIRT1
EGFR/HRAS/IL6/JUN/MAPK3/NOX4/SIRT1/TFRC
ATF3/CD44/EGFR/FBXW7/HRAS/JUN/MAPK3/NOX4/TLR4
ATF3/CHAC1/EIF2S1/HSPA5/NFE2L2/WIPI1/XBP1
DDIT4/HIF1A/PRKAA2/SLC2A6/STAT3/TAZ/TP53
ALOX12/CAV1/CDKN1A/DPP4/HMOX1/IL33/IL6/PIK3CA/SOCS1/TFRC/TLR4/TNFAIP3/XBP1
CAV1/HIF1A/VEGFA/XBP1
PRKAA2/PTGS2/SIRT1/TRIB3
EGFR/IL6/TFRC/TNFAIP3
CAV1/CHMP6/NF2/SOCS1
ATG7/HIF1A/NFE2L2/SIRT1
HIF1A/PRKAA2/STAT3/TAZ
CDKN2A/IL6/SIRT1
CAV1/NF2/SOCS1
ATG7/CAV1/CDKN1A
CD44/MUC1/SIRT1
AKR1C1/AKR1C2/AKR1C3
AURKA/CAV1/FBXW7/HSPA5/IL33/MAPK9/NFE2L2/SIRT1/SQSTM1/TNFAIP3/TRIB3/UBC/XBP1
DDIT4/HIF1A/PRKAA2/SLC2A6/STAT3/TAZ/TP53
DDIT4/HIF1A/PRKAA2/SLC2A6/STAT3/TP53
EGFR/ELAVL1/STAT3/TP53/ZFP36
DUOX1/GCH1/NFE2L2/PTGS2/TXNRD1
CYBB/DUOX1/EGFR/STAT3
BECN1/EGFR/MAPK3/ZFP36
CAV1/EGFR/FBXW7/TNFAIP3/TP63/XBP1
CDKN1A/EGFR/FTH1/JUN/TP53
AKR1C3/ALOX12/ALOX5/PTGS2/SIRT1
DDIT4/HIF1A/PRKAA2/SLC2A6/STAT3/TAZ/TP53
AURKA/CD44/MUC1/PRKAA2/SIRT1/TP53/TP63
IL33/IL6/STAT3/TLR4/TNFAIP3/XBP1
ALOX12/CAV1/CD44/CRYAB/MAP3K5/MAPK3/PTGS2/SIRT1/STAT3/TP63/VEGFA
BECN1/SQSTM1/TP53
PIK3CA/TP53/TP63
HMOX1/PTGS2/VEGFA
CAV1/NF2/SOCS1
MAPK8/PRKAA2/SIRT1/TP53/VEGFA
CAV1/PRKAA2/PTGS2/SIRT1/TRIB3
IL33/IL6/STAT3/TLR4/XBP1
CDKN1A/EGFR/FTH1/JUN/TP53
EGFR/ELAVL1/STAT3/TP53/ZFP36
EGFR/ELAVL1/STAT3/TP53/ZFP36
AKR1C3/ALOX15B/RB1/SIRT1/STAT3/TLR4/TNFAIP3/TP63/UBC
CAV1/FBXW7/HSPA5/MAPK9/TGFBR1/TNFAIP3/TRIB3
HIF1A/JUN/STAT3/TP53
AURKA/CAV1/FBXW7/IL33/MAPK9/NFE2L2/TNFAIP3/TRIB3
ATG7/FBXW7/HIF1A/MAPK8/SQSTM1/TP53/TP63
DDIT4/HIF1A/PRKAA2/SLC2A6/STAT3/TP53
ALOX15B/IL33/MAPK3/PANX1/TLR4/XBP1
CDKN1A/HMOX1/JUN/MAP3K5
CDKN1A/HMOX1/JUN/MAP3K5
CAV1/HSPA5/SIRT1/TGFBR1/TP53/UBC
GCH1/HAMP/MAP3K5/MAPK3/NFE2L2/PTGS2/SIRT1/TNFAIP3/ZFP36
CAV1/EGFR/HRAS/IL33/IL6/MAPK3/MUC1/NRAS/PTGS2/TLR4/TNFAIP3/UBC
ALOX12/CAV1/EGFR/GCH1/HMOX1/PTGS2/VEGFA
IL33/IL6/XBP1
DDIT4/STAT3/TP53
EGFR/HIF1A/TP63
AKR1C2/AKR1C3/PRKAA2
RGS4/SLC1A5/SLC3A2
CDKN2A/SIRT1/TP53
AKR1C3/FBXW7/IDH1/PRKAA2/PTGS2/SIRT1/TRIB3
EGFR/IL33/IL6/JUN
MAPK3/MUC1/SIRT1/TP53/VEGFA
CAV1/LPIN1/PTGS2/RB1/STAT3/TLR4/VEGFA
ALOX12/CD44/CRYAB/MAP3K5/PTGS2/SIRT1/TP63/VEGFA
ATF3/CD44/EGFR/FBXW7/HRAS/JUN/MAPK3/NOX4/TLR4
DDIT4/ENPP2/HIF1A/IDH1/PRKAA2/SLC2A6/STAT3/TP53

Count
23
30
23
25
19
26
23
29
29
29
20
17
22
17
23
23
19
18
9
18
16
10
18
12
18
14
12
22
19
19
7
13
19
17
17
13
17
9
17
21
17
8
20
7
17
8
17
18
20
18
15
7
19
20
10
19
7
19
15
11
8
11
18
13
9
7
9
15
8
15
15
7
8
8
14
13
13
19
6
9
16
10
6
10
16
13
10
14
7

12
12
6
6
16
14
7
11
5
15
17
14
8
13
11
9
14
13
8
8
10
7
8
11
9
8
11
13
16
11
17
8
17
7
17
11
11
17
8
8
10
9
17
14
8
14
13
8
9
9
7
9
10
7
9
9
10
10
10
10
11
9
7
4
8
7
14
10
7
11
7
7
6
7
11
17
11
15
8
6
9
10
5
6
11
6
7
5
5
4
9
8
7
7
10
9
11
9
7
10
16
12
13
6
6

8
6
7
7
12
10
14
8
7
9
4
4
4
10
6
14
11
12
7
7
13
8
8
8
12
5
5
5
8
13
7
5
8
13
9
12
5
15
13
10
10
14
14
9
9
8
6
12
10
6
6
13
8
7
7
14
9
4
4
8
9
6
11
10
7
7
7
11
6
4
4
4
3
3
3
3
6
7
15
6
6
11
4
7
9
9
8
12
9
14
4
4
5
13
3
3
8
14
5
4
4
6
13
10
7

6
15
10
6
6
6
3
3
3
9
9
10
14
7
9
5
11
12
9
4
5
7
6
5
7
9
3
3
3
6
6
8
5
7
4
4
4
4
7
8
9
6
9
4
7
3
3
3
3
3
6
14
7
7
8
5
5
6
6
11
8
14
7
3
3
3
3
3
3
4
4
4
4
4
11
8
8
12
6
10
8
6
6
6
9
12
3
3
3
5
8
8
13
9
9
5
4
4
6
7
5
5
5
5
8

3
3
3
6
12
13
5
10
8
11
6
13
9
8
6
4
4
7
6
3
3
3
5
5
7
11
13
8
6
6
6
6
4
4
4
4
4
4
4
8
9
7
7
13
4
4
4
4
4
4
3
3
3
3
3
13
7
6
5
5
4
4
6
5
5
7
7
6
11
3
3
3
3
5
5
5
5
5
5
9
7
4
8
7
6
6
4
4
6
9
12
7
3
3
3
3
3
7
4
5
7
8
9
8
