# Supplementary material for: A Ferroptosis-Related Prognostic Risk Score Model to Predict Clinical Significance and Immunogenic Characteristics in Glioblastoma Multiforme
Source: Oxid Med Cell Longev. 2021 Nov 9;2021:9107857. doi: 10.1155/2021/9107857 (PMC8596022; doi:10.1155/2021/9107857)
Supplement: Supplementary 2 — Table S1: DEGs between GBM and normal brain tissue. Table S2: KEGG pathways enriched in ferroptosis-related genes. Table S3: GO enrichment analysis of molecular function (MF). Table S4: GO enrichment analysis of biological process (BP). Table S5: GO enrichment analysis of cellular component (CC). Table S6: cd-Ferr-Geneset1. Table S7: cd-Ferr-geneset2. Table S8: DEG.Subtype1. Table S9: DEG.Subtype2. Table S10: DEG.Subtype3. Table S11: DEG.Subtype4. Table S12: known ferroptosis genes. Table S13: a multifactor regulatory network of the ferroptosis key hub genes. Table S14: Lasso-logistic regression analysis of prognosis factors. Table S15: FRGPRS model applied for TCGA GBM and GSE4412 GBM dataset. [file 9107857.f2.zip › Table S5.pdf]

Table S5. GO enrichment analysis of cellular component (CC)

ID	Description	GeneRatio	BgRatio	pvalue	p.adjust	qvalue
GO:0005767	secondary lysosome	5/122	11/11816	4.75E-08	1.47E-05	1.24E-05
GO:0000407	phagophore assembly site	6/122	26/11816	2.08E-07	3.22E-05	2.72E-05
GO:0005776	autophagosome	8/122	65/11816	3.16E-07	3.26E-05	2.75E-05
GO:0043020	NADPH oxidase complex	4/122	10/11816	2.17E-06	0.000167277	0.00014132
GO:0061695	transferase complex, transferring phosphorus-containing groups	10/122	214/11816	7.25E-05	0.004482479	0.003786935
GO:0000421	autophagosome membrane	4/122	25/11816	0.000115697	0.005958374	0.005033816
GO:1902911	protein kinase complex	6/122	92/11816	0.000370292	0.014995783	0.012668895
GO:0045177	apical part of cell	11/122	313/11816	0.00038824	0.014995783	0.012668895
GO:0034045	phagophore assembly site membrane	3/122	15/11816	0.000446282	0.015322355	0.012944793
GO:0042470	melanosome	6/122	101/11816	0.0006104	0.017146691	0.014486048
GO:0048770	pigment granule	6/122	101/11816	0.0006104	0.017146691	0.014486048
GO:0036464	cytoplasmic ribonucleoprotein granule	7/122	157/11816	0.001195245	0.030063092	0.025398218
GO:0030139	endocytic vesicle	9/122	255/11816	0.001290136	0.030063092	0.025398218
GO:0016324	apical plasma membrane	9/122	257/11816	0.001362082	0.030063092	0.025398218
GO:1990204	oxidoreductase complex	5/122	82/11816	0.001564515	0.030170593	0.025489038
GO:0010494	cytoplasmic stress granule	4/122	49/11816	0.001601153	0.030170593	0.025489038
GO:0010008	endosome membrane	11/122	374/11816	0.001671352	0.030170593	0.025489038
GO:0035770	ribonucleoprotein granule	7/122	168/11816	0.001765524	0.030170593	0.025489038
GO:0005942	phosphatidylinositol 3-kinase complex	3/122	24/11816	0.00185515	0.030170593	0.025489038
GO:0044440	endosomal part	11/122	412/11816	0.003537151	0.05464899	0.046169135
GO:0005770	late endosome	7/122	192/11816	0.003731518	0.054906616	0.046386785
GO:0005901	caveola	4/122	73/11816	0.006805657	0.095588542	0.080756118
GO:1902554	serine/threonine protein kinase complex	4/122	76/11816	0.007835133	0.101203002	0.085499385
GO:0043235	receptor complex	9/122	336/11816	0.007991628	0.101203002	0.085499385
GO:0045121	membrane raft	8/122	282/11816	0.008808222	0.101203002	0.085499385
GO:0098857	membrane microdomain	8/122	283/11816	0.008987992	0.101203002	0.085499385
GO:0098797	plasma membrane protein complex	11/122	469/11816	0.009144206	0.101203002	0.085499385
GO:0000790	nuclear chromatin	8/122	284/11816	0.009170499	0.101203002	0.085499385
GO:0019898	extrinsic component of membrane	7/122	231/11816	0.009999326	0.106544545	0.090012084
GO:0098589	membrane region	8/122	291/11816	0.010526788	0.108425918	0.091601525
GO:0097038	perinuclear endoplasmic reticulum	2/122	17/11816	0.012994627	0.129527089	0.109428438
GO:0016605	PML body	4/122	89/11816	0.01346617	0.1300327	0.109855594
GO:0000785	chromatin	10/122	434/11816	0.01430181	0.133556616	0.112832705
GO:0005774	vacuolar membrane	8/122	309/11816	0.01469555	0.133556616	0.112832705
GO:0070820	tertiary granule	5/122	142/11816	0.015785278	0.139272177	0.117661386
GO:0090575	RNA polymerase II transcription factor complex	5/122	143/11816	0.016225885	0.139272177	0.117661386

GO:0031258	lamellipodium membrane	2/122	20/11816	0.017792602	0.148592275	0.125535289
GO:0044853	plasma membrane raft	4/122	100/11816	0.019846491	0.161383306	0.136341542
GO:0098802	plasma membrane receptor complex	5/122	156/11816	0.022714472	0.179968506	0.152042887
GO:0005912	adherens junction	10/122	473/11816	0.024428924	0.188713438	0.159430872
GO:0005811	lipid droplet	3/122	62/11816	0.026122814	0.18997532	0.160496949
GO:0030027	lamellipodium	5/122	163/11816	0.026818535	0.18997532	0.160496949
GO:0005769	early endosome	7/122	282/11816	0.026838311	0.18997532	0.160496949
GO:0005719	nuclear euchromatin	2/122	25/11816	0.027170037	0.18997532	0.160496949
GO:0045335	phagocytic vesicle	4/122	111/11816	0.027826297	0.18997532	0.160496949
GO:0101002	ficolin-1-rich granule	5/122	166/11816	0.028713348	0.18997532	0.160496949
GO:0070161	anchoring junction	10/122	487/11816	0.029096881	0.18997532	0.160496949
GO:0005905	clathrin-coated pit	3/122	65/11816	0.029510729	0.18997532	0.160496949
GO:1904813	ficolin-1-rich granule lumen	4/122	115/11816	0.031139479	0.196369365	0.165898834
GO:0005925	focal adhesion	8/122	362/11816	0.033818373	0.201005286	0.169815401
GO:0031965	nuclear membrane	6/122	234/11816	0.034269548	0.201005286	0.169815401
GO:0005924	cell-substrate adherens junction	8/122	365/11816	0.035250844	0.201005286	0.169815401
GO:0044798	nuclear transcription factor complex	5/122	176/11816	0.035634088	0.201005286	0.169815401
GO:0009925	basal plasma membrane	2/122	29/11816	0.03580347	0.201005286	0.169815401
GO:0030055	cell-substrate junction	8/122	369/11816	0.037224328	0.201005286	0.169815401
GO:0044454	nuclear chromosome part	9/122	438/11816	0.037421685	0.201005286	0.169815401
GO:0031983	vesicle lumen	7/122	303/11816	0.037466324	0.201005286	0.169815401
GO:0005635	nuclear envelope	8/122	370/11816	0.037729148	0.201005286	0.169815401
GO:0044437	vacuolar part	9/122	442/11816	0.039290173	0.203167144	0.171641805
GO:0016323	basolateral plasma membrane	5/122	181/11816	0.039449931	0.203167144	0.171641805
GO:0000791	euchromatin	2/122	32/11816	0.042877452	0.217198895	0.183496256

geneID	Count
FTH1/FTL/LAMP2/NCF2/SQSTM1	5
ATG7/BECN1/SQSTM1/ULK1/ULK2/WIPI1	6
BECN1/FTH1/FTL/GABARAPL1/LAMP2/SQSTM1/ULK1/WIPI1	8
CYBB/DUOX1/NCF2/NOX4	4
BECN1/CDKN1A/MAP3K5/PIK3CA/PRKAA2/RB1/SOCS1/TGFBR1/TP53/ULK1	10
GABARAPL1/LAMP2/ULK1/WIPI1	4
CDKN1A/MAP3K5/PRKAA2/RB1/TGFBR1/ULK1	6
CD44/DPP4/DUOX1/EGFR/HAMP/MTDH/MUC1/NF2/NOX4/SLC3A2/TF	11
ULK1/ULK2/WIPI1	3
ATP6V1G2/CAPG/HSPA5/SLC1A5/SLC3A2/TFRC	6
ATP6V1G2/CAPG/HSPA5/SLC1A5/SLC3A2/TFRC	6
EIF2S1/ELAVL1/GABPB1/PRKAA2/SOCS1/SQSTM1/ZFP36	7
BECN1/CAV1/CYBB/DPP4/EGFR/LAMP2/NCF2/TF/UBC	9
CD44/DPP4/DUOX1/EGFR/MTDH/MUC1/NOX4/SLC3A2/TF	9
CYBB/DUOX1/NCF2/NOX4/RRM2	5
EIF2S1/ELAVL1/PRKAA2/ZFP36	4
BECN1/CAV1/CHMP6/EGFR/LAMP2/STEAP3/TF/TFRC/TLR4/UBC/WIPI1	11
EIF2S1/ELAVL1/GABPB1/PRKAA2/SOCS1/SQSTM1/ZFP36	7
BECN1/PIK3CA/SOCS1	3
BECN1/CAV1/CHMP6/EGFR/LAMP2/STEAP3/TF/TFRC/TLR4/UBC/WIPI1	11
CHMP6/EGFR/LAMP2/MAPK3/SQSTM1/STEAP3/TF	7
CAV1/HMOX1/MAPK3/PTGS2	4
CDKN1A/RB1/TGFBR1/ULK1	4
CD44/EGFR/IL6/TF/TFR2/TFRC/TGFBR1/TLR4/VLDLR	9
CAV1/DPP4/EGFR/HMOX1/LAMP2/MAPK3/PTGS2/TGFBR1	8
CAV1/DPP4/EGFR/HMOX1/LAMP2/MAPK3/PTGS2/TGFBR1	8
CYBB/DUOX1/EGFR/IL6/NCF2/NOX4/SLC3A2/TF/TFR2/TFRC/TGFBR1	11
HIF1A/JUN/MUC1/RB1/SIRT1/STAT3/TP53/TP63	8
ALOX15B/BECN1/PIK3CA/SOCS1/TF/ULK1/WIPI1	7
CAV1/DPP4/EGFR/HMOX1/LAMP2/MAPK3/PTGS2/TGFBR1	8
CYBB/NOX4	2
RB1/SIRT1/SQSTM1/TP53	4
CDKN2A/HIF1A/JUN/MUC1/NFE2L2/RB1/SIRT1/STAT3/TP53/TP63	10
ATP6V1G2/DPP4/GABARAPL1/LAMP2/SLC2A6/ULK1/VLDLR/WIPI1	8
CYBB/FTH1/IDH1/LAMP2/NRAS	5
HIF1A/JUN/RB1/STAT3/TP53	5

CD44/DPP4	2
CAV1/HMOX1/MAPK3/PTGS2	4
IL6/TF/TFR2/TFRC/TGFBR1	5
CAV1/CD44/DPP4/EGFR/HSPA5/MAPK3/NF2/NOX4/RPL8/VEGFA	10
CAV1/HSD17B11/PLIN2	3
CAPG/CD44/DPP4/NF2/PIK3CA	5
CAV1/EGFR/MAPK3/NF2/TF/TFRC/TLR4	7
JUN/SIRT1	2
BECN1/CYBB/LAMP2/NCF2	4
ALOX5/ATG7/FTH1/IDH1/LAMP2	5
CAV1/CD44/DPP4/EGFR/HSPA5/MAPK3/NF2/NOX4/RPL8/VEGFA	10
TF/TFRC/VLDLR	3
ALOX5/ATG7/FTH1/IDH1	4
CAV1/CD44/DPP4/EGFR/HSPA5/MAPK3/NOX4/RPL8	8
ALOX5/EGFR/GCH1/LPIN1/MTDH/SIRT1	6
CAV1/CD44/DPP4/EGFR/HSPA5/MAPK3/NOX4/RPL8	8
HIF1A/JUN/RB1/STAT3/TP53	5
EGFR/TF	2
CAV1/CD44/DPP4/EGFR/HSPA5/MAPK3/NOX4/RPL8	8
AURKA/HIF1A/JUN/MUC1/RB1/SIRT1/STAT3/TP53/TP63	9
ALOX5/ATG7/EGFR/FTL/IDH1/TF/VEGFA	7
ALOX5/CYBB/EGFR/GCH1/LPIN1/MAPK3/MTDH/SIRT1	8
ATP6V1G2/DPP4/FTL/GABARAPL1/LAMP2/SLC2A6/ULK1/VLDLR/WIPI1	9
CA9/CD44/EGFR/TF/TFRC	5
JUN/SIRT1	2
