# Supplementary material for: A Ferroptosis-Related Prognostic Risk Score Model to Predict Clinical Significance and Immunogenic Characteristics in Glioblastoma Multiforme
Source: Oxid Med Cell Longev. 2021 Nov 9;2021:9107857. doi: 10.1155/2021/9107857 (PMC8596022; doi:10.1155/2021/9107857)
Supplement: Supplementary 2 — Table S1: DEGs between GBM and normal brain tissue. Table S2: KEGG pathways enriched in ferroptosis-related genes. Table S3: GO enrichment analysis of molecular function (MF). Table S4: GO enrichment analysis of biological process (BP). Table S5: GO enrichment analysis of cellular component (CC). Table S6: cd-Ferr-Geneset1. Table S7: cd-Ferr-geneset2. Table S8: DEG.Subtype1. Table S9: DEG.Subtype2. Table S10: DEG.Subtype3. Table S11: DEG.Subtype4. Table S12: known ferroptosis genes. Table S13: a multifactor regulatory network of the ferroptosis key hub genes. Table S14: Lasso-logistic regression analysis of prognosis factors. Table S15: FRGPRS model applied for TCGA GBM and GSE4412 GBM dataset. [file 9107857.f2.zip › Table S6.pdf]

cd-Ferr-Geneset1
CDO1
P3H3
FAXDC2
CYP46A1
EGLN2
EGLN3
CYP4F8
CYP2U1
CYGB
CYP2R1
DNAJC24
TPH2
ALKBH2
ISCA2
CYP4F22
TYW5
CYP1A1
CYP1A2
CYP1B1
CYP2A6
CYP2A7
CYP3A7
CYP2A13
CYP2B6
CYP2C19
CYP2C8
CYP2C9
CYP2C18
CYP2D7
CYP2D6
CYP2E1
CYP2F1
CYP2J2
CYP3A4
CYP3A5
CYP4A11
CYP4B1
CYP7A1
CYP8B1
CYP11A1
CYP11B1
CYP11B2
CYP17A1
CYP19A1
CYP21A2
CYP24A1
CYP26A1
CYP27A1
CYP27B1
CYP51A1
CYP4Z2P
CYP4Z1
ALKBH3
FDX1
FECH
CYP2G1P
PHF8
JMJD6
ETHE1
ISCU
HAAO
ALOX12
FXN
ALOX5
ALOX12B
ALOX15
ALOX15B
FTH1
FTL
CYP4X1
FBXL5
NFU1
P4HA3
CYP4A22
CYP4V2
CYP2S1
HBA1
HBA2
HBQ1
HBZ
AOX1
CYP27C1
CYP26C1
FTH1P19
AGMO
LCN2
CYP4F3
LTF
MELTF

ACO2
P4HA1
PAH
CYP39A1
PHF2
PHYH
PLOD1
PLOD2
FTHL17
ACP5
EGLN1
P4HTM
PPEF2
PPEF1
TET2
CYP2W1
P3H2
TMLHE
OGFOD1
ADI1
MIOX
HIF1AN
KDM3A
CYP26B1
PTGIS
CYP20A1
CYP4F11
ALOXE3
ABCE1
RRM2
MSMO1
SC5D
SCD
P3H1
CYP3A43
CYP4F12
SNCA
TBXAS1
TF
TH
TPH1
XDH
FTO
FA2H
OGFOD2
RIOX1
OGFOD3
SCD5
TET1
KDM7A
CALR
DOHH
BBOX1
CYP4F2
ALKBH1
P4HA2
PLOD3
CH25H
ALKBH8
FTMT
CYP7B1
HEPH
ABCB6
TCIRG1
PGRMC2
SLC46A1
CLTC
C1orf194
ATP6V1G3
CP
ATP6V0E2
DNM2
ABCB7
LMTK2
SLC39A14
ATP6V0A2
ATP6V0D2
ATP6V1C2
SCARA5
FLVCR1
SLC40A1
HFE
HPX
HRG
HEPHL1
IFNG
IREB2
REP15
ARHGAP1
MIR210
SLC11A2
ATP6V0A4

SLC22A17
SLC25A37
ATP6V1D
ATP6V1H
ATP6V1A
ATP6V1B1
ATP6V1B2
ATP6V0C
ATP6V1C1
ATP6V1E1
ATP6V0B
ATP6V1G2
ATP6V0A1
ATP6AP1
ATP7A
STEAP3
FLVCR2
SLC48A1
B2M
MCOLN1
TTYH1
HAMP
NECTIN1
SLC39A8
SLC11A1
TFR2
TFRC
NOX5
SLC25A28
ATP6V0E1
ATP6V1E2
ATP6V0D1
RAB11B
ATP6V1F
ASIC3
MMGT1
SFXN1
ATP6V1G1
GLRX3
TMEM199
HJV
ERFE
TMPRSS6
ALAS2
ATP13A2
GDF2
HIF1A
HMOX1
HMOX2
SMAD4
MYC
NUBP1
NEDD8
ACO1
BOLA2
CAND1
TTC7A
SKP1
BMP6
BOLA2B
SOD1
SRI
CYBRD1
NCOA4
NDFIP1
CCDC115
CUL1
ABCG2
CPOX
ABAT
DRD2
ALAD
G6PD
APBB1
PDX1
MDM2
NFYA
CCND1
BCL2
SLC6A3
TFAP2A
TFF1
C1QA
MAP1LC3A
CCNB1
BTBD9
EPAS1
EPB42
STEAP2
STEAP1
EIF2AK1
NEO1
BDH2
RHAG
STEAP4

PICALM
HYAL2
MAP1LC3C
ACSL6
CYBB
MAP1LC3B
SAT1
VDAC2
GCLC
VDAC3
ACSL5
ATG7
ACSL1
ATG5
SLC3A2
TP53
LPCAT3
PCBP1
PCBP2
ACSL4
SLC7A11
ACSL3
GCLM
PRNP
SAT2
GSS
GPX4
