# Supplementary material for: A Ferroptosis-Related Prognostic Risk Score Model to Predict Clinical Significance and Immunogenic Characteristics in Glioblastoma Multiforme
Source: Oxid Med Cell Longev. 2021 Nov 9;2021:9107857. doi: 10.1155/2021/9107857 (PMC8596022; doi:10.1155/2021/9107857)
Supplement: Supplementary 2 — Table S1: DEGs between GBM and normal brain tissue. Table S2: KEGG pathways enriched in ferroptosis-related genes. Table S3: GO enrichment analysis of molecular function (MF). Table S4: GO enrichment analysis of biological process (BP). Table S5: GO enrichment analysis of cellular component (CC). Table S6: cd-Ferr-Geneset1. Table S7: cd-Ferr-geneset2. Table S8: DEG.Subtype1. Table S9: DEG.Subtype2. Table S10: DEG.Subtype3. Table S11: DEG.Subtype4. Table S12: known ferroptosis genes. Table S13: a multifactor regulatory network of the ferroptosis key hub genes. Table S14: Lasso-logistic regression analysis of prognosis factors. Table S15: FRGPRS model applied for TCGA GBM and GSE4412 GBM dataset. [file 9107857.f2.zip › Table S9.pdf]

Table S9. DEG.Subtype2

ID	baseMean	log2FoldChange	lfcSE	stat	pvalue	padj
AATK	537.808193	-1.229425505	0.240370904	-5.114701832	3.14E-07	9.45E-06
ABAT	5451.876906	-0.672888653	0.147633806	-4.557822291	5.17E-06	8.74E-05
ABCA13	62.50684162	1.406377631	0.348918943	4.030671472	5.56E-05	0.00057723
ABCA2	3376.924344	-0.641381963	0.168157852	-3.814166017	0.000136644	0.00118047
ABCA8	496.5615424	0.582988117	0.209842672	2.778215278	0.005465839	0.02123684
ABCC3	1490.228041	0.814556631	0.278672914	2.922984581	0.003466937	0.01476694
ABCG4	35.31303096	-1.071207285	0.228971922	-4.678334679	2.89E-06	5.61E-05
ABLIM2	168.1335329	-0.633962537	0.166408262	-3.809681866	0.000139146	0.00119758
ACADL	43.73882994	-1.314190846	0.317964901	-4.133131809	3.58E-05	0.00040836
ACAN	207.9386464	-1.181679999	0.351334249	-3.363406789	0.000769868	0.00457072
ACAP1	39.8144755	0.874605372	0.212042218	4.124675649	3.71E-05	0.00041982
ACBD7	313.7987766	-1.000292108	0.224042344	-4.464745762	8.02E-06	0.00012494
ACP5	187.3549224	1.054633536	0.266446388	3.958145362	7.55E-05	0.00073204
ACPP	31.92115004	1.125170827	0.250233606	4.496481696	6.91E-06	0.00011081
ACSL1	1311.862408	0.798791128	0.16425363	4.863156626	1.16E-06	2.69E-05
ACSS3	583.8768874	0.86588169	0.218041997	3.97116932	7.15E-05	0.00070105
ACVR1C	31.29429426	-0.882279218	0.294142299	-2.999497929	0.00270425	0.01215411
ACVR2B	274.2793504	-1.063427927	0.151102583	-7.037787861	1.95E-12	5.07E-10
ADAM11	138.0778594	-1.187425514	0.206637471	-5.746419117	9.12E-09	5.55E-07
ADAM12	765.1181713	0.84504969	0.219787411	3.844850291	0.000120626	0.00107243
ADAM19	673.5258138	0.825523877	0.229306229	3.600093557	0.000318103	0.0023091
ADAM8	248.0216458	0.896044489	0.23948363	3.741568842	0.000182875	0.00149007
ADAMTS14	176.5771666	1.0666962	0.255157303	4.180543489	2.91E-05	0.00034591
ADAMTS17	49.60385607	-1.200008429	0.252337379	-4.75557143	1.98E-06	4.11E-05
ADAMTS18	50.35419421	1.124935176	0.261775675	4.297325089	1.73E-05	0.00023233
ADAMTS1	967.6561641	1.009187123	0.20813086	4.848810615	1.24E-06	2.84E-05
ADAMTS7	182.1106742	-0.920614552	0.225466163	-4.083160599	4.44E-05	0.00048757
ADAMTS8	100.6946504	-0.894910194	0.239389653	-3.738299396	0.000185269	0.00150601
ADAMTSL1	136.2004805	0.805040651	0.315522549	2.551452044	0.010727509	0.03580693
ADAMTSL3	35.11141611	-0.798793438	0.238483244	-3.349474051	0.000809651	0.00474347
ADAP1	330.461852	-0.653802243	0.213651253	-3.060137655	0.002212353	0.01043757
ADARB2	32.35145241	-1.16753798	0.293342297	-3.980121482	6.89E-05	0.00068345
ADCY1	620.4142836	-0.711696914	0.239044905	-2.977251972	0.002908449	0.0128784
ADCY2	1740.385694	-1.321658604	0.214898267	-6.150159432	7.74E-10	6.96E-08
ADCY5	177.713448	-1.303403162	0.232813109	-5.598495586	2.16E-08	1.11E-06
ADORA3	1555.430899	0.591552193	0.186703098	3.16841124	0.001532745	0.00783859
ADPRHL1	39.77037751	-0.689535776	0.173953474	-3.963909211	7.37E-05	0.00071862
ADRA2A	72.27443382	1.495584964	0.278803414	5.364299322	8.13E-08	3.20E-06
AFAP1L2	624.9215399	-0.915984194	0.202777546	-4.517187484	6.27E-06	0.00010158
AFF2	70.76481703	-1.957713178	0.361605739	-5.413943887	6.17E-08	2.53E-06
AFF3	350.4838174	-0.863511162	0.201857011	-4.277835877	1.89E-05	0.00024806
AGAP2	2935.60921	-2.951140756	0.329804083	-8.948163188	3.61E-19	1.24E-15
AGAP8	53.27835331	-0.606258439	0.194621436	-3.115065078	0.001839043	0.0090389
AGBL4	30.82032267	-0.637627722	0.238834476	-2.6697474	0.007590833	0.02740426
AGPAT9	29.40547793	0.956066017	0.230964738	4.139445812	3.48E-05	0.00039893
AHR	983.6114585	0.686486409	0.211830033	3.240741641	0.001192192	0.00640146
AIFM3	134.0596337	-1.107803634	0.33011292	-3.355832399	0.000791265	0.00466955
AIM1	223.7893861	1.119228928	0.250842211	4.46188432	8.12E-06	0.00012605
AJAP1	36.08135976	-0.743879402	0.248823675	-2.989584505	0.002793572	0.01247409
AK5	380.2998381	-1.49337145	0.313675707	-4.760876968	1.93E-06	4.03E-05
AKAP12	3258.00459	0.624197185	0.205247972	3.041185638	0.002356485	0.01095236
AKR1C2	50.63291694	-1.181284543	0.309771299	-3.813408629	0.000137063	0.00118335
AKR7L	15.83395901	-0.711853974	0.178063962	-3.997743094	6.39E-05	0.00064427
AKT3	831.0232635	-0.595518686	0.141643864	-4.204338054	2.62E-05	0.00031946
ALCAM	2140.495126	-0.66790421	0.154873981	-4.312565656	1.61E-05	0.00021923
ALDH3B1	312.3703784	0.628088558	0.159096361	3.947849945	7.89E-05	0.00076156
ALDOC	8324.329755	-0.983501641	0.213122253	-4.614729925	3.94E-06	7.10E-05
ALOX15B	112.5959407	1.360480941	0.328837239	4.137247187	3.51E-05	0.00040144
ALOX5	547.5266994	0.694257523	0.190113432	3.651806796	0.000260402	0.00197229
ALOX5AP	1683.425201	0.852322269	0.19352143	4.404278476	1.06E-05	0.00015639
ALPK2	74.20206969	1.102277759	0.347267579	3.174145315	0.001502784	0.00772267
ALS2CL	68.26983203	0.76770149	0.246935793	3.108911349	0.001877781	0.00918258
ALX3	66.62906967	-0.844331889	0.209767413	-4.025086055	5.70E-05	0.000588

AMH	104.1028944	-0.999390192	0.273381759	-3.655657919	0.000256523	0.00194827
AMICA1	64.75956105	0.877423868	0.25653142	3.420336839	0.000625436	0.00389821
AMOT	1011.211749	-0.657325354	0.173409583	-3.790594175	0.000150287	0.00126731
AMOTL2	1277.48577	-1.151292455	0.177894894	-6.47175662	9.69E-11	1.29E-08
AMPH	433.4287241	-0.720146361	0.20704603	-3.47819449	0.000504804	0.00331288
AMZ1	60.16017937	-0.747550129	0.228087866	-3.277465569	0.001047435	0.00579839
ANG	156.001888	0.596836675	0.152984991	3.901276012	9.57E-05	0.00088438
ANKLE1	35.80513998	-0.680962974	0.216142135	-3.150533213	0.001629727	0.00822453
ANKRD13B	488.5458286	-1.026924564	0.131789097	-7.792181508	6.59E-15	4.77E-12
ANKRD20A3	50.55067297	-0.903749933	0.255511484	-3.537022749	0.000404665	0.0027845
ANKRD24	112.6770514	-0.840688698	0.217905208	-3.85804776	0.000114296	0.00102472
ANKRD29	45.41212233	0.831133672	0.231680408	3.58741458	0.000333973	0.00239433
ANKRD36B	21.29141127	-0.721710417	0.197713988	-3.650274939	0.00026196	0.00198082
ANKRD6	611.1334203	-0.721574457	0.131359168	-5.493141183	3.95E-08	1.82E-06
ANKS1B	343.900948	-1.106803653	0.2486275	-4.451654184	8.52E-06	0.00013132
ANO3	35.17030941	-1.803411113	0.281156233	-6.414266871	1.42E-10	1.74E-08
ANO5	146.7688807	-1.24494564	0.2637784	-4.719664844	2.36E-06	4.75E-05
ANXA2	6022.805593	0.699544966	0.178223565	3.925098053	8.67E-05	0.00081999
ANXA2P1	68.89676002	0.7783378	0.170175444	4.573737436	4.79E-06	8.27E-05
ANXA2P2	3059.954182	0.750799633	0.183490856	4.091755025	4.28E-05	0.000474
AOAH	244.4052055	0.603183586	0.210222059	2.869268757	0.00411422	0.01691156
AP3B2	500.9229044	-0.795141189	0.164923799	-4.821264076	1.43E-06	3.18E-05
APBA1	518.2306158	-0.889462955	0.155231953	-5.729896056	1.00E-08	6.01E-07
APBA2	2297.488917	-0.611663136	0.129335705	-4.729267423	2.25E-06	4.56E-05
APC2	4475.926681	-0.600260874	0.173328145	-3.46314716	0.000533896	0.00346699
APOA1	6.069903189	0.942716483	0.216848492	4.347350883	1.38E-05	0.0001933
APOBEC3B	88.40233552	0.628836674	0.197625419	3.181962517	0.001462807	0.0075681
APOBEC3C	272.84461	0.747139157	0.160942699	4.642268095	3.45E-06	6.44E-05
APOBEC3D	33.1087164	0.965978448	0.182961302	5.279687226	1.29E-07	4.69E-06
APOBEC3F	96.46245168	0.705266795	0.161971325	4.354269455	1.34E-05	0.00018942
APOBEC3G	282.987984	0.863618874	0.176212419	4.901010253	9.53E-07	2.29E-05
APOC1	3351.82592	0.758131032	0.203052193	3.733675671	0.000188705	0.00153078
APOL1	720.5459304	1.433412355	0.224287575	6.390957472	1.65E-10	1.96E-08
APOL3	287.6948927	0.646782238	0.15667249	4.128243826	3.66E-05	0.00041575
APOL6	990.6563918	0.69129315	0.170734518	4.048936068	5.15E-05	0.00054425
AQP9	176.9859297	0.830068297	0.313938218	2.644049847	0.008192059	0.02906396
ARHGAP15	130.7628414	0.59011571	0.165083007	3.574660536	0.000350683	0.00248855
ARHGAP18	608.320089	0.823296507	0.133762376	6.154918381	7.51E-10	6.85E-08
ARHGAP28	34.96488015	-1.228928782	0.291603308	-4.214385607	2.50E-05	0.00030858
ARHGAP29	712.5763189	0.590650418	0.13811689	4.276453228	1.90E-05	0.00024913
ARHGAP33	1130.014515	-0.720378929	0.155950048	-4.61929277	3.85E-06	7.02E-05
ARHGAP36	69.42322005	-1.089813214	0.302650312	-3.600899022	0.000317119	0.00230543
ARHGAP39	527.9928486	-0.641306273	0.113993872	-5.625796017	1.85E-08	9.81E-07
ARHGAP9	239.2820888	0.61983923	0.160673967	3.857745241	0.000114438	0.00102532
ARHGDIG	157.6933017	-1.153910943	0.312446882	-3.693142775	0.0002215	0.00174008
ARHGEF16	19.50921994	-1.549099821	0.297901561	-5.200039293	1.99E-07	6.58E-06
ARHGEF35	11.23668047	0.878943014	0.242640904	3.622402491	0.000291879	0.00215727
ARHGEF4	1618.6509	-0.615800288	0.153461375	-4.01273798	6.00E-05	0.00061365
ARHGEF5	17.25558868	1.143870803	0.309499037	3.695878391	0.000219128	0.00172435
ARHGEF9	1297.863432	-0.580937626	0.117617086	-4.939228169	7.84E-07	1.96E-05
ARL11	92.20802267	0.806997822	0.185710368	4.345464548	1.39E-05	0.00019418
ARPC1B	2197.794728	0.731779986	0.148164457	4.93897119	7.85E-07	1.96E-05
ARPP21	203.375943	-1.126854249	0.230826642	-4.881820578	1.05E-06	2.48E-05
ARSI	212.1563662	1.473906879	0.309008227	4.76979818	1.84E-06	3.90E-05
ARSJ	326.9335761	0.932939732	0.228557751	4.081855581	4.47E-05	0.00048953
ARVCF	676.0715437	-0.741898717	0.145448135	-5.100778469	3.38E-07	1.01E-05
ASCL1	1047.406083	-1.324819513	0.228729999	-5.792067161	6.95E-09	4.43E-07
ASGR2	12.5771924	0.942174135	0.244799057	3.848765385	0.000118715	0.00105813
ASPA	71.36448578	-0.846178733	0.244551494	-3.460124985	0.000539925	0.00348847
ASPN	89.33634779	0.783415781	0.231907898	3.378133244	0.000729797	0.00438579
ASRGL1	725.9150459	-0.734308389	0.131463037	-5.585664261	2.33E-08	1.17E-06
ASS1	616.1237322	1.127040692	0.241378268	4.669188734	3.02E-06	5.83E-05
ASXL3	52.32164833	-1.178124928	0.222361668	-5.298237492	1.17E-07	4.30E-06
ATAD3C	64.90504023	0.712733845	0.241892914	2.946485008	0.00321408	0.01395777
ATCAY	1812.086565	-1.891371575	0.321967018	-5.874426477	4.24E-09	2.93E-07
ATG9B	77.06973629	-0.828232645	0.252929669	-3.274557107	0.001058277	0.005849
ATP10B	210.872256	-1.06258419	0.309296116	-3.43549154	0.00059148	0.00372538
ATP1A3	2268.538409	-1.298487128	0.292765475	-4.435246776	9.20E-06	0.00013908
ATP2A1	19.51844902	-0.798976337	0.217561396	-3.672417779	0.000240266	0.00185449
ATP2B2	866.3104064	-1.049524652	0.213781977	-4.909322422	9.14E-07	2.24E-05
ATP2B3	63.54379686	-2.24439205	0.349978212	-6.41294792	1.43E-10	1.74E-08
ATP2C2	18.97124547	-1.448902644	0.267487585	-5.416709877	6.07E-08	2.51E-06
ATP6V1G2	804.4714841	-0.976857875	0.192991677	-5.061658059	4.16E-07	1.18E-05
ATP8A1	578.8572274	-0.890615796	0.199540707	-4.463328849	8.07E-06	0.00012548

ATP8B1	81.88610754	0.722416317	0.177682905	4.06576152	4.79E-05	0.00051555
ATP8B4	144.5599968	0.738950262	0.172462926	4.284690514	1.83E-05	0.00024259
AVIL	196.5587207	-0.894259471	0.251821385	-3.551165726	0.000383529	0.00268061
AXIN2	333.140482	-0.598186531	0.12107362	-4.940684267	7.78E-07	1.95E-05
AZGP1	703.596773	0.898656438	0.329069495	2.730901681	0.006316131	0.02379485
AZI1	290.6739697	-0.679116539	0.133157882	-5.100085183	3.40E-07	1.01E-05
B2M	54208.70856	0.726453625	0.140221439	5.180760024	2.21E-07	7.07E-06
B3GALT2	181.1623897	-1.159379172	0.228463557	-5.074678819	3.88E-07	1.13E-05
B3GNT5	625.4253277	0.671476972	0.139357868	4.818364258	1.45E-06	3.20E-05
B4GALNT1	980.5710219	-2.17709097	0.30028762	-7.250019071	4.17E-13	1.55E-10
B4GALT1	826.5463311	1.040812116	0.169758198	6.131144945	8.72E-10	7.70E-08
BACE2	539.3067411	0.621328286	0.171417324	3.624652814	0.00028935	0.00214549
BACH2	206.5700794	-0.876062104	0.190067582	-4.609213698	4.04E-06	7.23E-05
BAIAP2L1	19.23075116	0.804821357	0.249613643	3.224268305	0.00126295	0.00670035
BAMBI	379.2832263	-0.615670469	0.20547962	-2.996260495	0.002733128	0.01226788
BASP1	1203.77833	-1.029813487	0.217882753	-4.726457119	2.28E-06	4.60E-05
BATF	34.1489404	0.602141756	0.221408739	2.719593444	0.006536222	0.0244771
BATF2	229.5694881	0.962657428	0.210871997	4.565126907	4.99E-06	8.57E-05
BATF3	189.8767546	0.810636958	0.208823363	3.88192655	0.000103632	0.00094637
BCAN	34491.61567	-0.828561326	0.226516362	-3.657843157	0.000254347	0.00193495
BCAS1	1629.054602	-1.758088137	0.332756883	-5.283401268	1.27E-07	4.63E-06
BCAS4	931.5313981	0.719615082	0.157758765	4.561490346	5.08E-06	8.66E-05
BCL11A	128.3193951	-1.344938071	0.233769511	-5.753265537	8.75E-09	5.35E-07
BCL11B	54.52619798	-1.159441181	0.255755028	-4.533405233	5.80E-06	9.58E-05
BCL2A1	163.550132	0.670386609	0.247990101	2.703279712	0.006865894	0.02542746
BCL3	439.5768994	0.858688582	0.180676959	4.752618079	2.01E-06	4.15E-05
BCL7A	564.3444214	-0.972283089	0.17376906	-5.595260108	2.20E-08	1.12E-06
BCOR	526.408461	-0.735905135	0.153300631	-4.800405133	1.58E-06	3.45E-05
BDKRB2	175.5655179	0.707052696	0.277353043	2.549287682	0.010794322	0.03597759
BEX5	138.6022279	-0.667981597	0.253654267	-2.63343331	0.008452643	0.0297888
BMP4	91.21754145	-0.607760395	0.208252502	-2.918382205	0.003518528	0.01493441
BMP6	77.06830252	-0.61526424	0.22518631	-2.732245317	0.006290428	0.02372114
BOK	412.9491731	-0.587588084	0.220584315	-2.663779988	0.007726808	0.0278102
BRSK2	403.5014579	-1.272102461	0.22020277	-5.776959401	7.61E-09	4.76E-07
BSN	509.9856965	-1.240307585	0.222988137	-5.562213317	2.66E-08	1.31E-06
BST2	2269.011515	0.899511683	0.213569743	4.211793626	2.53E-05	0.00031158
BTBD11	252.1611622	0.868995892	0.28756474	3.021913925	0.00251182	0.01151104
BTK	278.1971115	0.640648754	0.168763537	3.796132547	0.000146971	0.00124643
BTN3A1	816.9204032	0.591497175	0.124567476	4.748407806	2.05E-06	4.20E-05
BZRAP1	491.3237047	-0.985557979	0.2114357	-4.661265714	3.14E-06	6.00E-05
C10orf55	13.78701271	0.682150176	0.215987297	3.158288402	0.001586985	0.00804127
C11orf52	12.70242885	-0.610337233	0.247288778	-2.468115371	0.013582653	0.0430305
C11orf65	6.139482637	-0.600034763	0.203759384	-2.944820266	0.003231423	0.01401981
C11orf87	104.7362048	-0.876997091	0.308653998	-2.841359889	0.004492159	0.01812756
C11orf95	736.4371169	-0.631441019	0.136980336	-4.609720173	4.03E-06	7.23E-05
C14orf132	2150.864589	-0.690966507	0.174977845	-3.948879967	7.85E-05	0.00075935
C14orf23	92.3515428	0.769686014	0.252258751	3.051176663	0.002279464	0.01068096
C15orf48	53.15211541	0.914819644	0.272761094	3.35392277	0.000796746	0.00469384
C16orf54	35.35153048	0.613804636	0.16939951	3.62341448	0.000290739	0.00215
C17orf51	543.3865403	-0.689228762	0.120839393	-5.703676139	1.17E-08	6.77E-07
C17orf96	165.9800236	-0.791706524	0.17122188	-4.623863049	3.77E-06	6.89E-05
C19orf57	165.4410653	-0.719004727	0.160410416	-4.482282053	7.38E-06	0.00011749
C1orf106	331.3434772	-1.076074348	0.191584311	-5.616714341	1.95E-08	1.03E-06
C1orf162	385.1446642	0.679719099	0.174631669	3.892301448	9.93E-05	0.00091224
C1orf198	2267.392801	-0.620871119	0.145063391	-4.279998648	1.87E-05	0.00024636
C1orf220	8.400082879	-0.776640143	0.204509204	-3.797580398	0.000146115	0.00124279
C1orf95	155.7427184	-0.960039453	0.235393372	-4.078447267	4.53E-05	0.00049401
C1QA	6175.895195	0.683906164	0.19420584	3.521553028	0.000429027	0.00291281
C1QB	9859.203409	0.599140424	0.192722032	3.108832016	0.001878285	0.00918258
C1QC	8069.807506	0.684519054	0.18605473	3.679127393	0.000234033	0.00181452
C1QL1	2811.980677	-1.394322478	0.26189831	-5.323907885	1.02E-07	3.85E-06
C1QTNF1	1863.10285	0.823259569	0.235237342	3.499697635	0.000465786	0.00310269
C1QTNF4	56.5805739	-1.213908261	0.218563204	-5.554037635	2.79E-08	1.36E-06
C1R	5934.911773	0.897464733	0.204162502	4.39583529	1.10E-05	0.00016087
C1RL	872.6162862	0.6580124	0.182639559	3.602792324	0.000314817	0.0022944
C1S	4744.353213	0.950425397	0.221964482	4.281880555	1.85E-05	0.00024476
C20orf112	652.9143471	-0.73409552	0.144362012	-5.085101737	3.67E-07	1.08E-05
C2	928.3655513	0.619193535	0.211992263	2.920830825	0.003490993	0.01484704
C2CD4C	115.3410491	-0.897260739	0.203716656	-4.404454491	1.06E-05	0.00015639
C2orf27A	63.88239551	-0.82231603	0.1957335	-4.201202294	2.66E-05	0.00032192
C2orf88	590.7677204	-0.605338395	0.219912413	-2.752634049	0.005911794	0.02262461
C3	22026.58519	0.764011991	0.203969505	3.745716747	0.000179879	0.00147001
C4orf21	116.6699911	-0.690642131	0.162868115	-4.24049931	2.23E-05	0.00028211
C5AR1	618.3334064	0.974181092	0.212808637	4.577732877	4.70E-06	8.16E-05

C5orf30	392.109661	-0.607134493	0.123746862	-4.906261745	9.28E-07	2.26E-05
C6orf141	84.30483938	0.94643247	0.340650289	2.778311074	0.005464228	0.02123657
C7orf13	75.64260971	-0.844284601	0.275715905	-3.062154146	0.002197503	0.01039602
C7orf41	4393.554434	-0.73347095	0.187594035	-3.909884176	9.23E-05	0.00086331
C8orf4	1034.644512	0.847702144	0.200384134	4.230385554	2.33E-05	0.0002924
C8orf56	61.22966793	0.782857205	0.217779859	3.594718125	0.000324743	0.00234722
C9orf117	17.16599722	-1.38099011	0.26607292	-5.190269313	2.10E-07	6.83E-06
CA10	370.7453754	-2.479369656	0.393377616	-6.302772587	2.92E-10	2.98E-08
CA12	2043.05079	1.520142389	0.226610221	6.708181054	1.97E-11	3.48E-09
CA4	85.56472519	-0.646572297	0.250345028	-2.582724737	0.00980235	0.0333498
CA9	488.6382966	1.148916588	0.340658715	3.372632308	0.000744533	0.00445582
CABLES1	290.5983972	-0.791390242	0.166652229	-4.748752809	2.05E-06	4.20E-05
CABP1	102.3342679	-1.693886975	0.292383298	-5.793378038	6.90E-09	4.43E-07
CABP7	176.7313783	-0.773674819	0.290963244	-2.659012208	0.007837012	0.02813066
CABYR	47.05900738	-0.647961898	0.208457647	-3.108362333	0.001881273	0.00919241
CACNA1A	283.2253875	-0.730345383	0.236101931	-3.09334778	0.001979121	0.009587
CACNA1E	144.3437748	-1.449224937	0.267681851	-5.413982798	6.16E-08	2.53E-06
CACNA1G	291.8289793	-0.773231824	0.248667311	-3.109503294	0.001874022	0.00917477
CACNA1H	207.2743113	-0.760667233	0.218746083	-3.477398195	0.000506305	0.00331723
CACNA1I	40.30615021	-1.755751526	0.310595773	-5.652850687	1.58E-08	8.55E-07
CACNA2D2	86.56242253	-1.239723928	0.279377288	-4.437454225	9.10E-06	0.00013812
CACNA2D4	84.08618688	0.790173291	0.172248512	4.587402704	4.49E-06	7.88E-05
CACNB1	390.7972282	-0.68956052	0.150240159	-4.589721711	4.44E-06	7.81E-05
CACNG4	2519.961688	-0.761813638	0.253875429	-3.000737967	0.002693262	0.01212056
CADM2	698.7520541	-1.02550375	0.225368562	-4.550340746	5.36E-06	8.98E-05
CADM3	2824.513332	-1.389060532	0.272876059	-5.090444868	3.57E-07	1.05E-05
CALHM2	403.4563066	0.671155031	0.125537656	5.346244718	8.98E-08	3.45E-06
CALN1	132.8131642	-1.132920001	0.304979329	-3.714743563	0.00020341	0.00162657
CALU	6513.865411	0.701147725	0.145199563	4.828855618	1.37E-06	3.08E-05
CAMK1G	83.17673571	-0.912041713	0.341284703	-2.672377946	0.007531577	0.02724036
CAMK2A	922.1096327	-1.666041255	0.336041391	-4.957845367	7.13E-07	1.83E-05
CAMK4	29.13826663	-0.90709049	0.215876978	-4.201886178	2.65E-05	0.00032152
CAMKK1	336.9677676	-1.095754589	0.201059813	-5.449893618	5.04E-08	2.17E-06
CAMKV	350.0404446	-2.302424773	0.296226558	-7.772512997	7.69E-15	5.29E-12
CAPN3	527.1078739	-0.840026768	0.24759996	-3.392677313	0.000692131	0.00421092
CARD16	150.5950002	0.96196803	0.20603333	4.668992294	3.03E-06	5.83E-05
CARD6	203.5794721	0.58585367	0.160178134	3.657513402	0.000254674	0.00193637
CARNS1	492.8379991	-1.177137855	0.33368758	-3.527664576	0.000419243	0.00285908
CASKIN1	228.6716218	-1.058837347	0.203223458	-5.210212243	1.89E-07	6.31E-06
CASP1	431.447986	0.79143252	0.165892931	4.770742877	1.84E-06	3.90E-05
CASP4	518.5916019	0.651459344	0.156740016	4.156305192	3.23E-05	0.00037781
CASP7	353.0931869	0.673123459	0.134754286	4.995191482	5.88E-07	1.58E-05
CASP8	261.8027723	0.582512585	0.123155216	4.72990593	2.25E-06	4.55E-05
CASS4	56.61902576	0.619174944	0.199814767	3.098744661	0.001943424	0.00945736
CAV1	2559.385901	0.616780356	0.2346651	2.628342928	0.008580197	0.03013694
CAV2	764.1995973	0.613077571	0.213939052	2.865664622	0.004161348	0.01704419
CBFA2T3	44.87542284	-0.606968463	0.198344374	-3.060174837	0.002212078	0.01043757
CBS	1663.425155	-0.610817421	0.146696965	-4.163804088	3.13E-05	0.00036752
CBX2	308.6472748	-1.074878109	0.209408489	-5.132925189	2.85E-07	8.69E-06
CCDC102B	255.0092636	0.642791533	0.134869773	4.766016277	1.88E-06	3.95E-05
CCDC109B	723.5266122	0.707330122	0.16067496	4.402242404	1.07E-05	0.00015736
CCDC114	14.08667768	-0.764960988	0.213731784	-3.579069869	0.000344819	0.00245748
CCDC136	386.1525255	-0.966844659	0.176302641	-5.484005525	4.16E-08	1.88E-06
CCDC141	18.56079543	-0.58673674	0.242679327	-2.417745041	0.015617015	0.04787734
CCDC150	39.43471261	-0.684295101	0.218952831	-3.125308302	0.001776188	0.00878329
CCDC64	51.18235327	-0.866700768	0.269286369	-3.218509613	0.001288587	0.00681011
CCDC78	76.05213386	-0.62178385	0.236532243	-2.628748798	0.008569964	0.03013694
CCDC85A	71.01069356	-1.007670259	0.237590888	-4.241199092	2.22E-05	0.000282
CCL2	1737.722758	1.170598683	0.277560035	4.21746122	2.47E-05	0.00030542
CCL8	33.85053773	0.944500057	0.279240878	3.382384646	0.000718595	0.00433171
CCNB3	15.15288578	-0.930892864	0.228721213	-4.069989191	4.70E-05	0.00050907
CCND1	1794.009252	-0.999804669	0.171555837	-5.827867388	5.61E-09	3.70E-07
CCNI2	24.13548101	-0.622222928	0.179458434	-3.467225896	0.00052586	0.00342493
CCR1	481.4022146	0.614440448	0.18413229	3.336951108	0.000847028	0.0049102
CCR2	34.8067116	1.192650131	0.292322506	4.079912111	4.51E-05	0.00049155
CCR5	147.8646427	0.666381086	0.20317054	3.279910002	0.001038402	0.00576673
CD109	475.4121485	0.619912437	0.221992414	2.792493789	0.005230347	0.02050129
CD14	3926.985587	0.632302953	0.201180425	3.142964597	0.001672461	0.00839096
CD163	4250.816608	1.118141045	0.27913235	4.005773764	6.18E-05	0.00062689
CD200	562.7554079	-0.606950199	0.150667351	-4.028412218	5.62E-05	0.00058193
CD209	77.49409536	1.098654167	0.363630763	3.021345496	0.00251654	0.01152884
CD274	50.17208707	1.3304399	0.248278499	5.358659351	8.38E-08	3.29E-06
CD27	19.58801175	0.630959249	0.235908154	2.674597035	0.007481912	0.02712613
CD28	19.11733644	0.824504012	0.238881203	3.451523186	0.000557432	0.00357974

CD2	53.75129209	0.65927349	0.273657842	2.409116015	0.015991214	0.04880707
CD300C	77.21374124	0.710639869	0.175642093	4.045954227	5.21E-05	0.00054869
CD300LB	14.7019326	1.077565614	0.201832662	5.33890602	9.35E-08	3.58E-06
CD300LF	88.37353936	0.622001212	0.194684725	3.194915322	0.001398719	0.00728583
CD33	188.1169079	0.731896324	0.180622013	4.052088174	5.08E-05	0.00053987
CD36	230.9631581	0.954777663	0.301519668	3.166551849	0.001542578	0.00787133
CD3E	50.17778739	0.659178857	0.256400814	2.570892222	0.010143689	0.03433287
CD40	215.2084635	0.664709484	0.158805881	4.185672982	2.84E-05	0.00033995
CD48	172.5495254	0.939384615	0.224108674	4.191647727	2.77E-05	0.00033286
CD52	97.35124796	0.737310542	0.238770945	3.087940802	0.002015486	0.00973232
CD53	1775.757234	0.590312908	0.170473188	3.462790331	0.000534605	0.00346876
CD58	413.7790385	0.732812206	0.153933014	4.760591563	1.93E-06	4.03E-05
CD63	20792.46702	0.672529767	0.141401621	4.75616732	1.97E-06	4.10E-05
CD68	3950.578924	0.660676836	0.163747915	4.034719061	5.47E-05	0.00057073
CD69	94.53959029	0.750459451	0.219597354	3.417433933	0.000632144	0.0039329
CD72	79.59352567	0.772319184	0.206629089	3.737707924	0.000185705	0.00150778
CD74	45158.18644	0.642767535	0.177277857	3.62576323	0.000288109	0.0021386
CD79B	21.0524628	0.821033888	0.24778452	3.313499513	0.000921363	0.00524392
CD80	10.25793066	0.844722444	0.218246603	3.870495275	0.000108614	0.00098339
CD86	465.4041849	0.643018598	0.171165496	3.756706883	0.000172164	0.00141875
CD8B	17.66380246	0.915057723	0.292444899	3.128991914	0.001754071	0.0087052
CD93	1609.5943	0.647131315	0.167788968	3.856816838	0.000114873	0.00102788
CD96	17.67710319	1.029320756	0.242649221	4.24201138	2.22E-05	0.0002815
CDC25A	241.0325076	-0.711489454	0.180976603	-3.931389158	8.45E-05	0.00080269
CDC42EP3	317.1857324	0.705064254	0.162973041	4.326263103	1.52E-05	0.00020788
CDC42EP5	32.24961214	0.686520569	0.224154444	3.062712286	0.002193408	0.01038022
CDCP1	269.5252801	1.15232454	0.211642271	5.444680466	5.19E-08	2.19E-06
CDH20	390.4559774	-0.814620278	0.2191063	-3.717922654	0.000200868	0.00161374
CDH22	109.6487016	-1.582431085	0.316299213	-5.002956123	5.65E-07	1.53E-05
CDH3	135.960851	-1.135880319	0.283332704	-4.008998272	6.10E-05	0.00061977
CDH5	813.0618182	0.632961354	0.145438989	4.352074768	1.35E-05	0.00019054
CDH6	453.3328248	0.667818966	0.172349092	3.874804093	0.000106711	0.00096971
CDH8	42.1097868	-1.574609655	0.294460281	-5.347443296	8.92E-08	3.44E-06
CDHR1	207.0472164	-1.093101176	0.237597913	-4.600634589	4.21E-06	7.50E-05
CDK4	20333.85724	-2.681957732	0.342537147	-7.82968433	4.89E-15	4.04E-12
CDK5R1	834.1861867	-1.193126307	0.191668857	-6.224935681	4.82E-10	4.67E-08
CDKL2	34.47903581	-0.892377427	0.279331579	-3.194688659	0.001399818	0.0072888
CDKN1A	3733.813044	0.879646754	0.177848391	4.946048433	7.57E-07	1.92E-05
CDKN1C	354.1151612	-0.824942698	0.191579858	-4.305999105	1.66E-05	0.00022451
CEACAM19	66.8748643	-0.595716658	0.204339873	-2.91532264	0.00355321	0.01505056
CEACAM21	37.36937354	0.608611942	0.200921474	3.029103506	0.002452806	0.01129325
CEBPB	787.8063913	0.748988179	0.150998877	4.960223514	7.04E-07	1.82E-05
CEBPD	1705.553892	0.588869314	0.171989116	3.423875465	0.000617349	0.0038618
CECR1	1167.160605	0.622109265	0.176170369	3.531293428	0.000413533	0.00282574
CECR6	244.0976649	-0.714683975	0.160591564	-4.450320792	8.57E-06	0.00013199
CELF3	360.5852381	-1.610653797	0.313009733	-5.14569876	2.67E-07	8.28E-06
CELF4	208.8421525	-2.070364031	0.329934309	-6.275079536	3.49E-10	3.48E-08
CELF5	312.1412195	-1.563080005	0.308851332	-5.060946299	4.17E-07	1.18E-05
CELF6	188.6830672	-0.664090943	0.19261605	-3.447744575	0.000565288	0.00361501
CELSR3	533.2277687	-1.234201687	0.239498063	-5.153284639	2.56E-07	7.99E-06
CENPJ	219.4464952	-0.812783561	0.179440235	-4.529550234	5.91E-06	9.73E-05
CENPV	546.4880732	-0.86877389	0.313137937	-2.774412767	0.005530146	0.02144431
CES3	43.33415644	0.855704807	0.223527911	3.828178783	0.000129095	0.00113449
CFB	629.8340982	1.298221917	0.272374057	4.766320011	1.88E-06	3.95E-05
CFI	1151.69945	0.637293673	0.216283931	2.946560428	0.003213296	0.01395777
CH25H	213.0097758	0.894351732	0.238006303	3.757680875	0.000171495	0.00141494
CHADL	361.9745832	-0.745627376	0.220376582	-3.383423816	0.000715881	0.00431914
CHCHD2	10492.411	0.706140704	0.23749101	2.973336569	0.002945812	0.01300618
CHD5	180.7399531	-1.714673603	0.354966162	-4.830526923	1.36E-06	3.06E-05
CHD7	1115.97488	-1.156513188	0.185882673	-6.221737453	4.92E-10	4.73E-08
CHGA	294.5775107	-2.182235291	0.377664021	-5.778245139	7.55E-09	4.74E-07
CHGB	468.4996659	-0.671368879	0.273963917	-2.450574099	0.01426286	0.0445597
CHI3L1	94501.02402	1.060650041	0.343064248	3.091695057	0.001990172	0.00963099
CHI3L2	7401.157708	1.059405899	0.315503894	3.357821943	0.000785592	0.00464404
CHIT1	155.8658894	1.35294834	0.381192317	3.549253956	0.000386324	0.00269575
CHL1	3778.652318	0.72204185	0.222745027	3.241562161	0.001188765	0.00638805
CHML	242.1127875	-0.651641163	0.144874493	-4.497970275	6.86E-06	0.00011017
CHODL	226.6602046	1.0007444	0.375914912	2.662156694	0.007764173	0.02792018
CHRDL1	544.7274874	-0.923401934	0.268044485	-3.444957774	0.000571149	0.00364233
CHRM1	92.17712506	-2.049458657	0.331384262	-6.184538289	6.23E-10	5.79E-08
CHRNA1	319.0735693	2.984202201	0.381270097	7.827003013	5.00E-15	4.04E-12
CHRNA9	139.0813909	1.662897381	0.303369834	5.481419682	4.22E-08	1.90E-06
CHRNB2	237.5546837	-1.061858841	0.235066203	-4.517275667	6.26E-06	0.00010158
CHST2	1462.745042	0.874127862	0.143230162	6.1029594	1.04E-09	8.79E-08

CHST8	326.4206398	1.252938228	0.362321556	3.458083594	0.000544033	0.00350708
CHST9	462.1426462	-0.712006058	0.265829196	-2.678434382	0.007396722	0.02689397
CHSY3	98.52712018	0.854598484	0.205236913	4.163960921	3.13E-05	0.00036752
CIT	841.5839446	-0.986116393	0.150899714	-6.534912289	6.36E-11	9.52E-09
CKMT1B	132.2418921	-1.645983114	0.313223373	-5.25498177	1.48E-07	5.20E-06
CLCF1	191.3306955	0.898888418	0.224637948	4.001498517	6.29E-05	0.00063646
CLDN23	51.30883965	1.195128047	0.229262986	5.212913205	1.86E-07	6.28E-06
CLDN7	27.97469026	0.700143116	0.170267492	4.112018721	3.92E-05	0.00044098
CLEC2B	220.6556976	0.596182459	0.233828441	2.549657585	0.010782877	0.03596557
CLEC4A	80.39241834	0.714857364	0.179740941	3.977153784	6.97E-05	0.00068756
CLEC4E	66.18871862	0.793886517	0.270755859	2.932112052	0.003366653	0.0144426
CLEC5A	381.3679045	0.87051267	0.232412402	3.745551717	0.000179998	0.00147011
CLEC7A	279.6166336	0.848970842	0.187135573	4.536662002	5.72E-06	9.46E-05
CLGN	112.4376172	-1.351880218	0.315376001	-4.286566559	1.81E-05	0.00024151
CLIC1	5308.689966	0.740015986	0.140687951	5.259981251	1.44E-07	5.09E-06
CLIC5	35.90513625	-0.632923603	0.221382417	-2.858960579	0.004250316	0.01733121
CLSTN2	198.2305447	-0.698501441	0.243993377	-2.862788532	0.004199307	0.01715372
CLSTN3	1315.757715	-0.617748251	0.119097306	-5.186920422	2.14E-07	6.92E-06
CLVS1	103.7208177	0.795285211	0.318670889	2.49563182	0.012573303	0.04042846
CMTM5	449.4509498	-1.144276089	0.202173447	-5.659873273	1.51E-08	8.27E-07
CNIH2	780.449792	-0.782452666	0.171187587	-4.570732487	4.86E-06	8.38E-05
CNKSR1	21.61725773	-1.843157687	0.248239999	-7.424902098	1.13E-13	5.01E-11
CNN1	95.86171199	1.131246119	0.238796258	4.737285785	2.17E-06	4.42E-05
CNN2	896.5338462	0.950327186	0.20856599	4.556482027	5.20E-06	8.78E-05
CNNM1	40.95004051	-1.648456988	0.332835226	-4.952772002	7.32E-07	1.87E-05
CNP	8492.121244	-0.816517007	0.140307765	-5.819471265	5.90E-09	3.83E-07
CNR1	1722.413671	0.873921111	0.215817432	4.049353676	5.14E-05	0.00054412
CNTFR	904.6705882	-1.364183728	0.215081315	-6.342641743	2.26E-10	2.49E-08
CNTN1	1393.363367	-0.815203007	0.251013313	-3.247648487	0.001163629	0.00628488
CNTN2	1505.499812	-0.816747606	0.274984582	-2.970157815	0.002976468	0.01312048
CNTN4	45.48301072	-1.214527455	0.281441934	-4.315374892	1.59E-05	0.00021753
CNTNAP2	254.02535	-1.986894108	0.280110229	-7.093257953	1.31E-12	4.10E-10
CNTNAP3	94.64761735	1.249179047	0.246837124	5.060742188	4.18E-07	1.18E-05
COL11A1	737.0420321	-1.256572989	0.376401015	-3.338388948	0.000842657	0.00489517
COL11A2	193.2603648	-1.702657548	0.262069961	-6.496958079	8.20E-11	1.15E-08
COL12A1	485.25239	0.987470973	0.314029275	3.144518848	0.001663602	0.00834956
COL15A1	190.764865	0.929294866	0.292050489	3.18196648	0.001462787	0.0075681
COL1A1	8549.979548	1.334802036	0.321880061	4.14689258	3.37E-05	0.0003891
COL1A2	10310.29509	1.699781509	0.286721622	5.928333894	3.06E-09	2.20E-07
COL20A1	1142.447088	-2.588555882	0.392819613	-6.589680853	4.41E-11	6.97E-09
COL23A1	134.7984165	1.607553939	0.334696672	4.803017397	1.56E-06	3.41E-05
COL25A1	50.25061558	0.91436556	0.356280406	2.566421127	0.010275399	0.03465932
COL3A1	8931.54185	1.114089679	0.311806143	3.573020305	0.000352887	0.00249817
COL4A4	63.81443957	-0.846298969	0.263744042	-3.208788951	0.001332953	0.00700691
COL5A2	2850.249659	0.678094966	0.209123186	3.242562341	0.0011846	0.00637064
COL6A1	10571.05409	0.750374787	0.229428331	3.270628272	0.001073089	0.00590006
COL6A2	6805.594084	0.796006156	0.315585993	2.522311428	0.011658644	0.03813793
COL6A3	1814.120486	1.600014134	0.376464386	4.250107558	2.14E-05	0.00027481
COL7A1	205.6759526	0.755193058	0.264484831	2.855335997	0.004299132	0.01749398
COL8A1	297.4239693	1.002233372	0.31581213	3.173511331	0.00150607	0.00773667
COL8A2	457.5655454	1.273227395	0.205555886	6.194069266	5.86E-10	5.49E-08
COL9A1	146.7830793	-1.374970413	0.286440218	-4.800200267	1.59E-06	3.45E-05
COLQ	93.47913666	-0.663428584	0.189528682	-3.500412578	0.000464539	0.00309587
COPZ2	336.3706094	0.774293043	0.196900929	3.932399137	8.41E-05	0.00079987
CORO6	37.47265735	-0.668403925	0.243887986	-2.740618491	0.006132367	0.02326175
COX7A1	235.4961059	0.760295283	0.269503896	2.821091992	0.004786048	0.01909701
CP	1368.063339	0.930842935	0.270200975	3.445002129	0.000571055	0.00364233
CPAMD8	34.26674154	-0.890413702	0.292314135	-3.046085001	0.002318423	0.01081564
CPD	2060.366447	0.632107755	0.137231348	4.606146939	4.10E-06	7.33E-05
CPLX1	280.6106598	-0.958124929	0.251999933	-3.802084059	0.000143484	0.00122963
CPLX2	947.8639117	-2.060816062	0.352873921	-5.840091712	5.22E-09	3.45E-07
CPNE4	338.5358209	1.849137661	0.340035156	5.438077884	5.39E-08	2.26E-06
CPNE7	38.19487788	-1.11224294	0.331786391	-3.352286199	0.000801471	0.00470958
CPVL	1570.373908	0.94278464	0.183612302	5.134648558	2.83E-07	8.65E-06
CPXM1	1654.803865	-0.595039363	0.20830327	-2.856601156	0.004282035	0.01743965
CPXM2	69.30995792	-1.180968464	0.355308067	-3.323787363	0.000888039	0.0051091
CPZ	81.20395688	1.708066164	0.313075619	5.455762319	4.88E-08	2.13E-06
CRABP2	163.5951537	0.813483695	0.187510187	4.338344011	1.44E-05	0.00019856
CRB1	751.494906	-0.931423118	0.198808269	-4.685032078	2.80E-06	5.47E-05
CREB3L1	348.6761809	0.702641429	0.275150487	2.553662315	0.010659658	0.03562366
CREG2	61.42083585	-1.315199286	0.333486985	-3.943779952	8.02E-05	0.00076991
CREM	682.5607395	0.639938811	0.129394157	4.945654641	7.59E-07	1.92E-05
CRHBP	35.10520382	-0.901917291	0.296115608	-3.045828273	0.002320403	0.01082121
CRHR1	29.96595657	-1.693947225	0.298834788	-5.668507461	1.44E-08	7.96E-07

CRIM1	1041.090838	0.644373784	0.150893526	4.27038721	1.95E-05	0.00025528
CRIP1	235.4019262	0.693495586	0.207153622	3.347735745	0.000814747	0.00476387
CRISPLD2	287.5457942	0.747375204	0.185972081	4.018749471	5.85E-05	0.00060223
CRLF1	170.0014148	-1.608422575	0.378000154	-4.255084445	2.09E-05	0.00026932
CRMP1	4558.791326	-1.088294893	0.161774581	-6.727230472	1.73E-11	3.13E-09
CROCC	433.3944699	-0.644545888	0.18118897	-3.557313044	0.000374668	0.0026307
CRTAC1	191.9384786	-0.822817062	0.297733222	-2.763605143	0.005716667	0.02203719
CRYM	190.3353011	-1.795044913	0.378943015	-4.736978496	2.17E-06	4.42E-05
CSDC2	340.3731909	-1.451730475	0.266642594	-5.444480768	5.20E-08	2.19E-06
CSF2RB	143.7431202	0.856118346	0.206201977	4.151843547	3.30E-05	0.0003827
CSMD1	155.4563153	-1.116661421	0.22782047	-4.901497309	9.51E-07	2.29E-05
CSMD2	439.6697405	-0.7000662	0.21568862	-3.245726174	0.001171515	0.00632003
CSMD3	53.65948496	-2.835838473	0.324193475	-8.747364421	2.18E-18	3.76E-15
CST7	35.16741587	0.924825463	0.230580473	4.010857687	6.05E-05	0.00061764
CSTA	70.22597204	1.1220124	0.20897487	5.369125974	7.91E-08	3.13E-06
CTBS	447.6338845	0.585760976	0.132912777	4.407108091	1.05E-05	0.00015486
CTDSP2	6964.874036	-0.887336128	0.203575634	-4.3587541	1.31E-05	0.00018671
CTGF	2976.136744	0.790947037	0.194176589	4.073338819	4.63E-05	0.00050259
CTNNA3	18.44533949	-1.648787463	0.296970555	-5.552023372	2.82E-08	1.37E-06
CTNND2	5849.22254	-0.70136648	0.153474154	-4.569932218	4.88E-06	8.40E-05
CTRL	24.09333649	-0.596828505	0.155692623	-3.833376904	0.000126396	0.00111719
CTSB	29045.13976	0.77610915	0.145064974	5.350079549	8.79E-08	3.41E-06
CTSC	2074.412977	0.83422325	0.180137586	4.6310338	3.64E-06	6.73E-05
CTSS	1841.734461	0.900660105	0.182978044	4.922230479	8.56E-07	2.11E-05
CTSW	26.14462848	0.743227099	0.240423388	3.091326114	0.001992647	0.00963895
CTSZ	3153.589838	0.776653053	0.176851135	4.391563868	1.13E-05	0.00016337
CTTNBP2	501.6316101	-0.78004891	0.167447464	-4.658469544	3.19E-06	6.04E-05
CUX2	58.94285311	-2.474147378	0.349430785	-7.080507743	1.44E-12	4.30E-10
CXCL10	461.0515415	0.906126335	0.299822761	3.022206621	0.002509392	0.01150972
CXCL11	119.0461866	1.452408568	0.316523045	4.58863451	4.46E-06	7.84E-05
CXCL1	66.11204085	1.186845432	0.311465962	3.810514071	0.000138678	0.00119447
CXCL14	2265.99398	1.790250309	0.304164696	5.885792576	3.96E-09	2.78E-07
CXCL2	147.4452265	1.214703816	0.28670867	4.23671812	2.27E-05	0.0002861
CXCL5	118.6053889	1.019450166	0.384479294	2.651508626	0.008013307	0.02858453
CXCR2	52.24872224	0.745755422	0.242253456	3.078409836	0.002081085	0.0099617
CXorf57	234.9860204	-1.356054146	0.263249494	-5.151212734	2.59E-07	8.06E-06
CYBB	1861.968194	0.809911082	0.176323415	4.593326889	4.36E-06	7.71E-05
CYFIP2	1902.206579	-0.633197184	0.13460785	-4.704013796	2.55E-06	5.09E-05
CYP19A1	93.81410946	0.824041131	0.241416862	3.413353662	0.000641686	0.0039743
CYP1B1	507.4557484	0.886302048	0.295662866	2.997677929	0.00272045	0.01221889
CYP21A2	44.10341468	-1.117858372	0.231944219	-4.819513823	1.44E-06	3.19E-05
CYP26B1	107.1568141	-1.258367855	0.277872186	-4.528585144	5.94E-06	9.76E-05
CYP27B1	150.9644034	-2.591377201	0.355102782	-7.297541261	2.93E-13	1.12E-10
CYP2E1	26.3087273	-0.610162628	0.250146909	-2.439217138	0.014719121	0.04562264
CYP2S1	70.99602242	0.600679657	0.165966036	3.619292659	0.000295409	0.00217985
CYP46A1	334.375784	-0.770889806	0.183529789	-4.200352484	2.66E-05	0.00032228
CYP4X1	24.83777822	-0.72811888	0.266359891	-2.733590547	0.00626479	0.02365972
CYR61	2592.689387	0.876987747	0.233939337	3.748782738	0.000177695	0.00145475
CYS1	36.86481219	-0.909921599	0.224196921	-4.058582044	4.94E-05	0.00052917
CYTIP	130.1807828	0.746632195	0.182513193	4.090839597	4.30E-05	0.00047473
DAAM2	2801.366038	-1.720650681	0.265258576	-6.486691989	8.77E-11	1.21E-08
DAB2	965.8666687	0.643361793	0.156577964	4.108891036	3.98E-05	0.00044591
DACH1	95.13640201	-1.299367126	0.238972609	-5.437305697	5.41E-08	2.26E-06
DACH2	28.12708388	-2.405107749	0.342165655	-7.029074121	2.08E-12	5.30E-10
DARC	155.1035629	-1.187400292	0.282836747	-4.198182541	2.69E-05	0.00032437
DBN1	3552.88359	-0.643135336	0.12514548	-5.139101597	2.76E-07	8.50E-06
DCBLD2	1177.554824	0.877173854	0.183879739	4.770367091	1.84E-06	3.90E-05
DCC	64.40650496	-0.832335938	0.332659443	-2.502066167	0.012347085	0.03989281
DCN	4973.557597	1.503777619	0.258170836	5.824738549	5.72E-09	3.75E-07
DCTN2	5316.989944	-0.596759998	0.172716329	-3.455145222	0.000549996	0.00353859
DCX	1105.591911	-1.484663855	0.345570779	-4.296265612	1.74E-05	0.00023321
DDIT4	2981.170228	0.610150392	0.174168093	3.503227156	0.000459657	0.00307078
DDN	224.7353147	-2.11943415	0.312368815	-6.785037584	1.16E-11	2.24E-09
DDX25	152.241598	-0.847036947	0.262029459	-3.232601977	0.001226683	0.00654326
DENND2D	139.7396573	0.749157207	0.177638452	4.217314425	2.47E-05	0.00030542
DEPDC7	52.32295887	-0.643297774	0.178198687	-3.610002881	0.000306194	0.00224498
DGCR5	208.0714004	-0.75244114	0.189174101	-3.977506093	6.96E-05	0.00068703
DGCR9	43.77395077	-1.01951081	0.20025178	-5.091144816	3.56E-07	1.05E-05
DGKB	325.0935552	-1.185870373	0.222337067	-5.333660225	9.63E-08	3.68E-06
DGKI	68.77362536	-1.31786143	0.201905157	-6.527131098	6.70E-11	9.92E-09
DHRS3	1755.733735	0.704236664	0.145101197	4.853417346	1.21E-06	2.78E-05
DIRAS3	691.1869103	0.806895994	0.229490319	3.516035008	0.000438043	0.00296525
DIRC3	33.40383882	-0.786294392	0.221848743	-3.544281492	0.000393685	0.00272393
DISP2	256.8873763	-0.8034212	0.198266417	-4.052230391	5.07E-05	0.00053987

DKK1	206.1892803	2.408703399	0.448397333	5.371805806	7.80E-08	3.09E-06
DLEC1	118.5867783	-0.683323395	0.275473273	-2.480543351	0.013118231	0.0418288
DLG2	397.3281804	-0.895581881	0.186933419	-4.790913723	1.66E-06	3.58E-05
DLGAP3	119.9836457	-1.500810858	0.264685466	-5.670167239	1.43E-08	7.92E-07
DLL1	493.9245403	-1.651791236	0.204597574	-8.073366665	6.84E-16	9.41E-13
DLL3	1524.052686	-1.753021574	0.314060032	-5.581804098	2.38E-08	1.19E-06
DLX1	202.9713441	-1.636810175	0.307550927	-5.322078495	1.03E-07	3.88E-06
DLX2	49.37131726	-1.958002429	0.328026413	-5.969038927	2.39E-09	1.78E-07
DLX5	180.7037371	-3.512625614	0.398552361	-8.81346082	1.21E-18	2.78E-15
DMRTA2	309.0474417	0.805332449	0.301601208	2.670189734	0.00758084	0.02738255
DNAH10	34.00255506	-0.742507887	0.16998427	-4.368097645	1.25E-05	0.00017948
DNAH17	81.24402313	-0.68991804	0.204473306	-3.374122771	0.000740513	0.0044405
DNAJB1	5270.816885	0.642980402	0.124185202	5.177592775	2.25E-07	7.14E-06
DNASE1L1	396.0651532	0.62011405	0.127110475	4.878544044	1.07E-06	2.51E-05
DNASE1L2	28.89416625	-0.789887443	0.216827633	-3.642927937	0.000269554	0.00202822
DNER	6166.849829	-0.654629146	0.187349516	-3.494159799	0.000475556	0.00315403
DNM1	1504.054283	-1.190152788	0.209488403	-5.681234709	1.34E-08	7.54E-07
DNM3	493.8878497	-1.395826897	0.204175922	-6.836393247	8.12E-12	1.69E-09
DOC2A	129.6067544	-1.053677221	0.279676559	-3.767484936	0.0001649	0.00137204
DOCK2	507.2865117	0.586393262	0.181412976	3.232366688	0.001227694	0.00654612
DOK2	68.94588071	0.907149362	0.2248437	4.034577628	5.47E-05	0.00057073
DOK6	96.99888226	-1.480466768	0.275438457	-5.374945767	7.66E-08	3.05E-06
DPEP2	63.48927454	0.623588321	0.198122571	3.147487523	0.001646801	0.00829246
DPP10	197.2576686	-1.699126154	0.339187342	-5.009403191	5.46E-07	1.50E-05
DPP4	235.5917014	2.980888297	0.230566629	12.9285331	3.11E-38	4.28E-34
DPY19L2	104.8897804	-0.732534588	0.23410686	-3.129060756	0.00175366	0.0087052
DPYD	1087.548868	1.19537749	0.184135246	6.491845067	8.48E-11	1.18E-08
DPYSL4	828.34088	-1.145872529	0.178529726	-6.418385088	1.38E-10	1.71E-08
DRAM1	452.3747942	0.628102437	0.144129133	4.357914497	1.31E-05	0.00018707
DRD4	12.76507154	-0.957790422	0.244321042	-3.920212577	8.85E-05	0.00083507
DRP2	230.0526082	-0.971389077	0.180167054	-5.391602142	6.98E-08	2.81E-06
DSC2	117.2947568	0.712010037	0.233576945	3.048289025	0.002301485	0.0107585
DSCAM	471.5723869	-1.441206901	0.226792692	-6.354732539	2.09E-10	2.34E-08
DSCAML1	271.3808787	-1.844643125	0.296605494	-6.21918056	5.00E-10	4.78E-08
DSE	673.9890287	0.658005272	0.146920737	4.478641243	7.51E-06	0.00011891
DTX3	3174.568912	-1.074009137	0.198730258	-5.404356376	6.50E-08	2.65E-06
DTX4	770.916134	-1.529182943	0.174819461	-8.747212312	2.19E-18	3.76E-15
DUSP1	3510.440771	0.827330105	0.205471335	4.026498903	5.66E-05	0.00058536
DUSP23	303.4737471	0.598513526	0.159253536	3.758243253	0.00017111	0.00141261
DUSP26	280.5482781	-1.178173485	0.233504941	-5.045604082	4.52E-07	1.26E-05
DUSP4	245.271103	1.183405986	0.224304764	5.275884314	1.32E-07	4.76E-06
DUSP5	435.1951313	0.681505754	0.192861906	3.533646271	0.000409869	0.00280488
DUSP8	362.8367811	-0.941259086	0.181375845	-5.189550397	2.11E-07	6.84E-06
DYNC1I1	344.1047417	-1.881018359	0.277276783	-6.783901409	1.17E-11	2.24E-09
DYNLT3	841.2277885	0.71489877	0.136308686	5.244704423	1.57E-07	5.41E-06
E2F2	196.3583033	-0.747222635	0.204988467	-3.645193533	0.000267191	0.00201594
EBI3	124.6107006	0.679786753	0.180388034	3.76846922	0.000164252	0.00136799
ECHDC3	44.60641318	0.61428205	0.239036306	2.569827407	0.010174919	0.03440804
EDA2R	215.036123	0.650530588	0.225054515	2.890546711	0.003845724	0.01600389
EFCAB1	55.9091217	-2.033100968	0.407830923	-4.985156472	6.19E-07	1.64E-05
EFEMP1	7842.694627	0.801759455	0.225629979	3.553426099	0.000380248	0.00266309
EFHD1	1414.453341	-0.850297199	0.19929247	-4.266579652	1.98E-05	0.00025866
EFNA3	199.4621678	-0.661199494	0.159039654	-4.15745054	3.22E-05	0.00037693
EFNB2	1175.781487	0.596678247	0.155849003	3.82856633	0.000128892	0.00113415
EFNB3	1025.615899	-1.039836576	0.200591048	-5.183863323	2.17E-07	7.00E-06
EGFR	12114.70936	0.950367416	0.345216602	2.752959767	0.005905915	0.0226084
EGR3	436.1313065	0.590908549	0.2347174	2.517531928	0.011818026	0.03855848
EHD3	681.5361261	-0.728139349	0.151008876	-4.821831472	1.42E-06	3.17E-05
ELAVL2	155.496207	-2.111278868	0.307729338	-6.860830629	6.85E-12	1.50E-09
ELAVL3	1086.134618	-0.898789177	0.216066631	-4.15977781	3.19E-05	0.00037345
ELAVL4	157.0380212	-1.432247604	0.306350514	-4.675192422	2.94E-06	5.68E-05
ELF4	259.4927772	0.627140896	0.159008805	3.944063963	8.01E-05	0.00076991
ELFN1	225.9264976	-1.166417899	0.257895118	-4.52283823	6.10E-06	9.94E-05
ELFN2	386.8083412	-2.454359397	0.322656916	-7.606715601	2.81E-14	1.61E-11
ELL2	1388.492279	0.649908859	0.147677707	4.40085963	1.08E-05	0.00015803
ELMO1	1443.239329	-0.65411856	0.171114946	-3.822685132	0.000132006	0.00114979
ELOVL2	2163.268796	0.805533405	0.261655465	3.078603401	0.002079733	0.0099587
EMB	260.7003952	0.636545311	0.201643424	3.156786854	0.001595179	0.00807387
EMILIN1	1897.434963	0.580342337	0.214315696	2.70788537	0.006771341	0.02516532
EMR1	74.35471661	0.812625224	0.28395935	2.861766034	0.004212878	0.01719894
EN2	117.462069	-0.670314126	0.26224671	-2.556043987	0.010586973	0.03541515
ENHO	1136.352993	-0.890471739	0.212929349	-4.182005637	2.89E-05	0.00034429
ENO3	74.56193618	-0.649016339	0.188119804	-3.450016022	0.000560553	0.0035914
EPB41L4B	115.5413128	-0.790382025	0.269209407	-2.935937616	0.003325412	0.01429962

EPHA10	44.36297768	-2.172588053	0.306021919	-7.099452409	1.25E-12	4.01E-10
EPHA2	361.1695902	0.774807511	0.182001519	4.257148594	2.07E-05	0.00026755
EPHA5	162.7402143	0.594555723	0.235078624	2.529178168	0.011432997	0.03761412
EPHA7	84.24756271	-0.80497277	0.326883735	-2.462565996	0.013794678	0.04342083
EPHB1	949.5433494	-1.804842041	0.279071518	-6.467310088	9.98E-11	1.31E-08
EPHB6	269.7804311	-0.797173989	0.246236376	-3.237433884	0.001206099	0.00646352
EPSTI1	202.6775468	0.645513748	0.190210148	3.393687224	0.000689584	0.00419728
ERAP2	730.4975359	1.187457239	0.257273684	4.615541002	3.92E-06	7.08E-05
ERAS	8.500215355	-0.989798463	0.212357428	-4.661002309	3.15E-06	6.00E-05
ERBB3	556.9258688	-1.881675876	0.272321799	-6.909751207	4.86E-12	1.13E-09
ERBB4	317.3784314	-1.156265485	0.225820659	-5.120282132	3.05E-07	9.23E-06
ERMN	1054.521633	-0.749117966	0.311455097	-2.405219804	0.016162741	0.04921054
ERRFI1	1150.154092	1.128246844	0.181952684	6.200770562	5.62E-10	5.30E-08
ESR2	40.0106788	2.411683177	0.29696994	8.120967329	4.62E-16	7.07E-13
ETNK2	547.234162	-0.854354195	0.225938888	-3.7813508	0.00015598	0.0013089
ETV5	2328.600001	0.581076145	0.148623787	3.909711595	9.24E-05	0.00086334
ETV7	62.10216912	1.177882579	0.25077928	4.696889547	2.64E-06	5.22E-05
EVI2B	573.7613334	0.646100015	0.176489115	3.660849073	0.000251381	0.00191875
EXTL1	141.1877174	-0.584836155	0.219582123	-2.663405137	0.007735422	0.02783134
EYA1	197.3605737	-1.724081796	0.33178139	-5.196439123	2.03E-07	6.69E-06
EYA4	278.5307833	0.858535906	0.355242239	2.416761896	0.015659257	0.04797478
EYS	6.34594623	-0.787307912	0.232497467	-3.386307482	0.0007084	0.00428527
F13A1	2649.742211	1.52851703	0.363107701	4.209541762	2.56E-05	0.00031386
F2RL3	29.24899791	0.620236102	0.225678676	2.748315055	0.005990242	0.02287343
FA2H	240.0216727	-1.614899739	0.31096353	-5.193212661	2.07E-07	6.76E-06
FAAH	163.2793255	-1.129841377	0.179806376	-6.283655802	3.31E-10	3.32E-08
FABP7	8028.988915	0.785777384	0.250439601	3.137592387	0.001703416	0.00851522
FADS2	9955.543125	-0.701590657	0.164884416	-4.255045285	2.09E-05	0.00026932
FAIM2	2719.053442	-0.957009344	0.167971905	-5.697436986	1.22E-08	6.97E-07
FAM107A	12862.59713	-0.621733937	0.203470055	-3.055653257	0.002245708	0.01054793
FAM117B	280.1926485	-0.626426067	0.123945212	-5.054056194	4.33E-07	1.22E-05
FAM129A	1030.254948	0.779280924	0.193322829	4.030982413	5.55E-05	0.00057723
FAM131C	31.34002082	-1.26316244	0.269497365	-4.68710497	2.77E-06	5.44E-05
FAM133A	33.28292675	-1.201177934	0.269439179	-4.45806708	8.27E-06	0.00012788
FAM155B	77.98964953	-0.969430956	0.248980496	-3.893601992	9.88E-05	0.00090797
FAM171A2	281.3137306	-1.123127116	0.156716753	-7.166605328	7.69E-13	2.65E-10
FAM177B	16.62712601	1.063044362	0.260805728	4.076000821	4.58E-05	0.00049834
FAM178B	36.69167411	-0.811898844	0.219609184	-3.697016805	0.000218148	0.00171935
FAM182B	300.8703863	-0.939127864	0.187634851	-5.005082261	5.58E-07	1.52E-05
FAM189A2	439.9371102	-0.991980974	0.22085206	-4.491608428	7.07E-06	0.00011325
FAM19A2	61.18508676	-1.329535575	0.257206852	-5.169129685	2.35E-07	7.42E-06
FAM19A5	787.8242008	-0.590002979	0.183564943	-3.214137566	0.00130837	0.00689085
FAM20A	291.2500164	0.633426769	0.246453268	2.570169895	0.010164865	0.03438763
FAM26F	163.9568798	0.701093629	0.217705511	3.220376122	0.001280225	0.00677372
FAM46A	729.2687851	0.886999148	0.136153291	6.514709579	7.28E-11	1.07E-08
FAM46B	120.7596989	0.755181561	0.265044998	2.84925793	0.004382134	0.01774777
FAM57B	259.2242513	-1.289377732	0.227579422	-5.665616508	1.46E-08	8.06E-07
FAM66A	8.215607363	-0.800899882	0.190776898	-4.198096786	2.69E-05	0.00032437
FAM66C	77.9278889	-0.609693888	0.114873315	-5.307532792	1.11E-07	4.11E-06
FAM66D	46.0376443	-1.127417433	0.296973724	-3.796354163	0.00014684	0.00124643
FAM78B	113.622356	1.20784881	0.19263984	6.269984479	3.61E-10	3.57E-08
FAM89A	277.1133353	-0.758487491	0.214377896	-3.538086268	0.000403038	0.00277469
FAM95B1	205.9772594	-0.787526899	0.276495591	-2.848243962	0.004396121	0.01779395
FAP	170.8418066	1.03296672	0.283802165	3.639742218	0.000272911	0.00204788
FAS	420.0417087	0.706637112	0.171372311	4.123403052	3.73E-05	0.00042146
FBLIM1	653.2811464	0.733103037	0.223993521	3.272876075	0.001064591	0.00587682
FBLL1	143.1375634	-0.698468025	0.272171774	-2.566276493	0.010279685	0.03466528
FBLN1	1882.850934	0.964543064	0.256911781	3.754374595	0.000173775	0.00142861
FBLN7	311.5173743	1.119199515	0.216232218	5.175914687	2.27E-07	7.19E-06
FBN1	782.4588225	0.732920878	0.203879258	3.594877114	0.000324545	0.00234702
FBN3	214.3678606	-0.995293391	0.370296656	-2.687827114	0.007191862	0.02630899
FBP1	223.4033715	0.600882728	0.198255272	3.030853723	0.002438634	0.01123928
FBXL16	1127.793025	-1.480355629	0.230513137	-6.422001129	1.34E-10	1.68E-08
FBXL2	176.9046578	-0.724436834	0.247471893	-2.927349951	0.00341864	0.01462005
FBXO32	889.201493	0.665659807	0.203408035	3.272534481	0.001065879	0.00587817
FBXO39	19.26621212	1.057845339	0.243311748	4.347695283	1.38E-05	0.0001932
FBXO41	514.1043658	-0.597047744	0.167173896	-3.571417294	0.000355055	0.00250964
FCER1G	1454.626818	0.677550549	0.172209781	3.93444869	8.34E-05	0.00079473
FCGBP	10172.60192	0.870490461	0.235531366	3.695857913	0.000219146	0.00172435
FCGR1B	416.2369334	0.634422372	0.193908591	3.271760004	0.001068803	0.00589061
FCGR2A	1941.509052	0.807067729	0.183940004	4.387668324	1.15E-05	0.00016597
FCGR2B	356.9441311	1.240732019	0.284962739	4.354014918	1.34E-05	0.00018945
FCGR2C	100.1331078	1.050924084	0.237209438	4.430363701	9.41E-06	0.00014154
FCGR3A	5023.484197	0.614458525	0.194071234	3.166149414	0.001544714	0.0078793

FCRLA	34.86382981	-1.193962123	0.300683839	-3.970822403	7.16E-05	0.00070157
FERMT1	498.1668131	-1.638263833	0.298632485	-5.485886212	4.11E-08	1.87E-06
FERMT3	619.5099588	0.701107172	0.159800996	4.387376727	1.15E-05	0.00016602
FGF12	316.5175569	-0.638206383	0.256034005	-2.492662577	0.012678927	0.04070152
FGF13	136.7649342	-1.655534136	0.294840768	-5.61501094	1.97E-08	1.03E-06
FGFBP2	135.8313759	-1.480064201	0.332637897	-4.449475586	8.61E-06	0.00013236
FGFBP3	237.8184175	-0.633738585	0.194631381	-3.25609663	0.001129553	0.00613453
FGFR3	1767.293348	-0.783930156	0.238962297	-3.280560012	0.001036012	0.00575668
FGFR4	39.71466276	-0.975840654	0.240838382	-4.051848563	5.08E-05	0.00054001
FGL2	712.5678703	0.650167507	0.173936526	3.737958459	0.000185521	0.00150716
FGR	272.8151072	0.593121715	0.154897067	3.829134572	0.000128595	0.00113247
FHL2	279.4370362	0.777708585	0.239073676	3.253008021	0.001141903	0.00618939
FHOD3	381.3644739	-0.802552984	0.163517304	-4.908061509	9.20E-07	2.24E-05
FLRT1	172.4199672	-1.539746461	0.228401039	-6.741416195	1.57E-11	2.88E-09
FLT3LG	155.1363717	0.613824139	0.144748571	4.240623139	2.23E-05	0.00028211
FMO3	27.35574816	0.730309356	0.224004578	3.26024299	0.001113168	0.00605511
FNDC3B	996.6286704	0.65050377	0.127932267	5.084751391	3.68E-07	1.08E-05
FNDC5	262.5640853	-0.631980495	0.225077841	-2.807830799	0.004987642	0.01972412
FOSL1	216.6326504	0.915284329	0.239145519	3.827311225	0.000129551	0.00113704
FOSL2	995.8723428	0.614491741	0.17276711	3.556763451	0.000375452	0.00263486
FOXD3	41.45677496	-1.061863674	0.31595832	-3.36077137	0.000777251	0.00460462
FOXH1	6.824901885	-0.59984529	0.213116566	-2.814634734	0.00488327	0.01941738
FOXL1	38.6219611	0.734116055	0.207167756	3.543582605	0.00039473	0.00272841
FOXN4	22.4392064	-1.806135458	0.325268742	-5.552748307	2.81E-08	1.37E-06
FPR1	1186.2896	0.845168769	0.232136244	3.640830722	0.00027176	0.00204146
FPR2	54.94570586	1.022625465	0.312942015	3.267779382	0.001083948	0.00593841
FPR3	309.6350705	0.739945352	0.222983396	3.318387677	0.000905387	0.00517439
FREM1	167.2873335	-0.841639602	0.314866939	-2.673000872	0.007517606	0.02721831
FRRS1	27.41794494	0.973340036	0.214505332	4.537603001	5.69E-06	9.45E-05
FRS3	183.6610806	-0.612139282	0.096148641	-6.366593151	1.93E-10	2.24E-08
FSD1L	125.833723	-0.639239601	0.164905977	-3.876388299	0.000106018	0.00096496
FST	74.04254644	1.228861289	0.325717314	3.772784673	0.000161436	0.00134892
FTL	64978.46836	0.634951155	0.158015566	4.018282314	5.86E-05	0.00060298
FUCA1	741.1745277	0.581855305	0.143785689	4.046684413	5.19E-05	0.00054782
FUT9	396.2340591	-1.294121696	0.264044519	-4.901149627	9.53E-07	2.29E-05
FXYD1	307.4802124	-0.748385941	0.283967739	-2.635461136	0.008402305	0.02964936
FXYD5	735.430517	0.68214148	0.141438297	4.822890934	1.41E-06	3.16E-05
FXYD7	160.7816595	-1.266114952	0.329253997	-3.845404963	0.000120354	0.00107135
FZD1	849.7781028	0.632028342	0.186962942	3.380500617	0.000723539	0.0043577
FZD6	317.4215165	0.700009763	0.197470439	3.544883819	0.000392786	0.00272393
FZD7	1072.403703	0.836492079	0.180980872	4.621991658	3.80E-06	6.94E-05
G0S2	194.3730233	0.653791763	0.252356608	2.590745561	0.009576827	0.03272816
GABBR1	2424.058595	-1.110185987	0.174742593	-6.353264914	2.11E-10	2.34E-08
GABRA2	89.25420783	-0.994657133	0.327314574	-3.038841568	0.002374897	0.01101562
GABRA3	127.0051194	-2.113388178	0.332536024	-6.355366109	2.08E-10	2.34E-08
GABRB2	128.488152	-1.131048777	0.350864148	-3.223608866	0.001265862	0.00671194
GABRB3	322.1101689	-1.671532714	0.320022161	-5.223178008	1.76E-07	5.99E-06
GABRD	133.4221094	-0.723242372	0.277436569	-2.606874696	0.009137279	0.0315898
GABRG1	90.78149873	-1.955276447	0.363779408	-5.374895893	7.66E-08	3.05E-06
GAD1	470.2924699	-1.010199153	0.251281961	-4.020181754	5.82E-05	0.00059948
GADD45A	1994.634983	0.609879533	0.158942161	3.837116141	0.000124488	0.00110458
GADD45B	1478.807626	0.690172926	0.187031455	3.690143589	0.000224128	0.00175352
GADD45G	543.0130151	-1.484473976	0.241746849	-6.140613548	8.22E-10	7.30E-08
GAL3ST1	136.6994353	-1.682381562	0.264591938	-6.3584007	2.04E-10	2.32E-08
GALNT13	596.4774299	-1.572970716	0.330154705	-4.764344388	1.89E-06	3.97E-05
GALNT14	56.87584193	-1.119701114	0.251898033	-4.445056998	8.79E-06	0.00013436
GAPT	97.60846638	0.802112507	0.186368362	4.303909194	1.68E-05	0.00022641
GARNL3	170.1309066	-0.6983193	0.133307764	-5.238399308	1.62E-07	5.59E-06
GATA3	25.78440065	0.893285762	0.321586661	2.777745069	0.005473755	0.02126159
GATS	1355.501651	-0.663169203	0.132445022	-5.007128196	5.52E-07	1.50E-05
GATSL1	34.40503496	-0.629395368	0.174217695	-3.612694846	0.000303031	0.00222892
GBP1	1877.657503	0.94098081	0.205237324	4.584842523	4.54E-06	7.94E-05
GBP2	1461.341704	0.682069841	0.18081153	3.772269616	0.000161769	0.00135089
GBP4	566.2016831	0.739252558	0.238314577	3.1020031	0.001922159	0.00936381
GBP5	128.5163208	1.393101931	0.232272786	5.997697598	2.00E-09	1.56E-07
GCH1	106.444904	0.715254881	0.172751421	4.140370462	3.47E-05	0.00039768
GCHFR	94.30139446	0.7809356	0.199971613	3.905232283	9.41E-05	0.00087356
GCNT1	150.6529354	1.313545652	0.232508203	5.649459392	1.61E-08	8.69E-07
GDA	200.5060484	-1.169976393	0.356721164	-3.279806499	0.001038783	0.00576673
GDAP1	658.4628418	-0.586287824	0.121031354	-4.844098681	1.27E-06	2.90E-05
GDAP1L1	405.26204	-1.455732573	0.25947634	-5.61027095	2.02E-08	1.05E-06
GDF15	325.389094	0.879086416	0.25587795	3.435569248	0.00059131	0.00372538
GDF5	26.35422872	0.711939548	0.242793802	2.93228057	0.003364826	0.01444103
GDPD1	139.8546895	-0.881074554	0.158177306	-5.570170438	2.54E-08	1.26E-06

GFAP	312457.4738	-0.672777184	0.186621065	-3.605044184	0.0003121	0.00227858
GFPT2	1260.361395	0.969543336	0.198369183	4.887570355	1.02E-06	2.42E-05
GFRA1	212.9265523	-2.401880807	0.340278147	-7.058580832	1.68E-12	4.63E-10
GFRA2	132.2003295	-1.152432152	0.258142019	-4.464333849	8.03E-06	0.00012504
GIMAP5	346.0833031	0.598081574	0.154644088	3.867471302	0.00010997	0.00099435
GIPC2	10.38165639	0.940087463	0.248654599	3.780696064	0.000156391	0.00131155
GJB1	307.0570205	-0.920254244	0.328860146	-2.798314893	0.005137	0.02021487
GJC2	115.6922282	-0.709053674	0.233730429	-3.033638694	0.002416236	0.01116596
GLCCI1	939.0156554	-1.109163905	0.190462968	-5.823514752	5.76E-09	3.76E-07
GLI1	65.52102806	-1.133193271	0.320538608	-3.535278573	0.000407345	0.00279355
GLI2	278.1707951	-0.642179754	0.207790957	-3.090508662	0.00199814	0.00966212
GLRX	991.0178925	0.670670833	0.160392103	4.18144548	2.90E-05	0.00034484
GLS2	35.43605285	-1.109048043	0.280080404	-3.959748793	7.50E-05	0.0007292
GLT1D1	50.80220374	-0.964507301	0.29051691	-3.319969574	0.000900273	0.00516016
GMFG	508.1094211	0.626142013	0.164720894	3.801230049	0.00014398	0.00123168
GNA15	231.735613	0.793724901	0.177735509	4.465764365	7.98E-06	0.00012449
GNAI1	589.4249495	-0.844552324	0.195452864	-4.321002542	1.55E-05	0.00021248
GNAL	48.7694286	-1.805446724	0.258344405	-6.988526522	2.78E-12	6.95E-10
GNAO1	2358.540653	-0.893336491	0.166826202	-5.354893186	8.56E-08	3.33E-06
GNG2	2247.732944	-0.635483503	0.157130758	-4.044297312	5.25E-05	0.00055132
GNG3	335.6927012	-1.012432442	0.286423133	-3.534743965	0.00040817	0.00279604
GNG4	1263.860089	-0.917483292	0.20509852	-4.473378414	7.70E-06	0.00012137
GNMT	12.87731458	-0.743247227	0.204353978	-3.637057793	0.00027577	0.00206258
GOLGA2B	67.54970814	-0.611021942	0.230515752	-2.650673271	0.008033151	0.02864047
GOLGA6L9	132.5293761	-0.587046067	0.169587956	-3.461602347	0.00053697	0.00347591
GOLGA7B	285.9959584	-1.281401511	0.24348304	-5.26279576	1.42E-07	5.05E-06
GPC2	393.3387418	-1.670948405	0.235830399	-7.085381756	1.39E-12	4.24E-10
GPC3	88.04326103	-1.494448817	0.319134551	-4.682817364	2.83E-06	5.51E-05
GPD1	886.7231483	-1.253353338	0.306223464	-4.092936976	4.26E-05	0.00047197
GPR123	400.2109878	-1.032712354	0.22121297	-4.668407799	3.04E-06	5.84E-05
GPR132	77.87571071	0.692467108	0.170945349	4.050809869	5.10E-05	0.00054157
GPR153	398.8508367	-0.645107248	0.241777087	-2.668190179	0.007626108	0.02750275
GPR17	868.2638585	-2.116232823	0.418509093	-5.056599383	4.27E-07	1.21E-05
GPR173	231.3646952	-0.662666772	0.138590296	-4.7814803	1.74E-06	3.72E-05
GPR179	23.30772792	-1.656861066	0.262692618	-6.307223556	2.84E-10	2.94E-08
GPR183	288.5671968	0.647979461	0.211635167	3.061775927	0.002200281	0.01039844
GPR39	105.9785957	0.666382462	0.225387676	2.956605581	0.003110457	0.01358062
GPR62	50.01358517	-0.915246437	0.280340617	-3.264765725	0.001095546	0.00597579
GPR65	228.3832981	0.817460682	0.185320782	4.411057805	1.03E-05	0.00015271
GPR83	23.24463213	-2.040954606	0.313536337	-6.509467526	7.54E-11	1.08E-08
GPR84	85.79366761	0.877649254	0.220186387	3.985937851	6.72E-05	0.00067126
GPR85	207.9952847	-0.680374589	0.152175567	-4.470984433	7.79E-06	0.0001219
GPR98	755.7315356	-0.855241503	0.215936024	-3.96062448	7.48E-05	0.00072704
GPRIN1	536.5349189	-0.783081746	0.173447681	-4.514800904	6.34E-06	0.00010261
GPSM1	1290.744532	-0.663976575	0.13661668	-4.860142798	1.17E-06	2.72E-05
GPT2	2196.703275	-0.61475898	0.120326189	-5.109103727	3.24E-07	9.71E-06
GPX8	453.9960506	0.901536729	0.205666319	4.383492315	1.17E-05	0.00016883
GRB10	1920.572662	0.821781092	0.18017383	4.561045803	5.09E-06	8.66E-05
GRHL1	40.69590735	-1.272824114	0.214424651	-5.93599713	2.92E-09	2.12E-07
GRHL3	72.17986424	-1.17678175	0.258760944	-4.547756443	5.42E-06	9.07E-05
GRIA2	1228.639832	-0.707260514	0.231381533	-3.056685225	0.002237992	0.01053326
GRID2IP	11.22068681	-1.311586915	0.20973263	-6.253614006	4.01E-10	3.91E-08
GRIK1	337.3678219	0.63389086	0.263471877	2.405914694	0.016132031	0.04913878
GRIK2	562.7047201	-1.155133025	0.243189352	-4.749932574	2.03E-06	4.19E-05
GRIK4	263.25288	-0.613197365	0.156526178	-3.917538742	8.95E-05	0.00084323
GRIN1	347.2832391	-2.270671607	0.440015441	-5.160436198	2.46E-07	7.74E-06
GRIN2A	164.6684658	-0.782919267	0.316609859	-2.472820241	0.013405155	0.04255634
GRIN2D	180.4098902	-0.851464551	0.202248189	-4.209998395	2.55E-05	0.00031351
GRIN3A	36.98353107	-0.973478468	0.229767823	-4.236791971	2.27E-05	0.0002861
GRM1	42.96864721	-1.50083603	0.283138978	-5.300704416	1.15E-07	4.26E-06
GRM2	34.65712947	-1.265233979	0.268927054	-4.704747848	2.54E-06	5.08E-05
GRM3	292.6393174	-0.702411882	0.231584621	-3.033067905	0.002420811	0.01118335
GRM5	125.9047524	-1.363104685	0.31992855	-4.26065346	2.04E-05	0.00026438
GRM6	22.30461813	-0.747703725	0.203411262	-3.675822649	0.000237084	0.00183301
GSDMB	66.07729516	-0.8897731	0.194481955	-4.575093351	4.76E-06	8.24E-05
GSDMD	796.3232929	0.610799109	0.174439976	3.501485865	0.000462672	0.00308792
GSTM5	310.6389464	-0.941555949	0.350295808	-2.687888148	0.007190548	0.02630899
GSTO1	1694.6062	0.614068269	0.166235752	3.693960296	0.000220788	0.00173628
GUSB	1651.224566	0.668491977	0.117745866	5.677413577	1.37E-08	7.68E-07
GXYLT2	50.28630558	1.149902515	0.212353753	5.415032696	6.13E-08	2.52E-06
H19	3260.288883	-1.289621284	0.436299796	-2.955814548	0.003118445	0.01361118
H2AFY2	251.004494	-1.064481765	0.26409177	-4.030726761	5.56E-05	0.00057723
H2BFXP	25.16999839	-1.01455614	0.200184022	-5.068117485	4.02E-07	1.16E-05
HAMP	318.9579153	0.787423332	0.286006328	2.753167516	0.005902169	0.02260035

HAS3	126.5717923	0.733813413	0.219546123	3.342411162	0.000830539	0.00483498
HCG22	58.60705194	-1.654473476	0.29498593	-5.608652162	2.04E-08	1.05E-06
HCK	628.3806522	0.672917847	0.181995181	3.697448707	0.000217777	0.00171751
HCLS1	1547.906553	0.593224309	0.176828856	3.354793573	0.000794242	0.0046831
HCN2	581.7416115	-0.60910173	0.234125645	-2.601601931	0.009278949	0.03197218
HCN3	163.9988122	-0.602620919	0.150882874	-3.99396501	6.50E-05	0.00065319
HCN4	31.6906076	-1.241202905	0.274623302	-4.519656175	6.19E-06	0.00010064
HCP5	483.9964432	1.012440345	0.212321601	4.768428363	1.86E-06	3.92E-05
HCST	206.8708315	0.582307681	0.178158476	3.268481486	0.001081263	0.00592842
HDAC11	594.1975936	-0.599269752	0.127694459	-4.692997304	2.69E-06	5.31E-05
HECW1	67.12554913	-1.830863452	0.32711813	-5.596948887	2.18E-08	1.11E-06
HEPH	508.8181337	0.659686738	0.221421466	2.979326029	0.002888832	0.0128039
HES6	2433.738202	-1.153816457	0.20014858	-5.764799616	8.18E-09	5.07E-07
HEXB	1954.918751	0.67328398	0.114468689	5.881817854	4.06E-09	2.82E-07
HEY2	321.7523218	-0.606060898	0.163496889	-3.706865028	0.000209841	0.00166734
HFE	143.0040861	0.739437448	0.170132245	4.346251051	1.38E-05	0.00019408
HHATL	241.6478854	-0.802245945	0.316830635	-2.532097143	0.011338257	0.03738311
HHIP	49.27283209	-1.110212943	0.289692556	-3.832383407	0.000126908	0.00112027
HIF3A	459.2699266	-0.827832892	0.27862124	-2.971176541	0.002966612	0.01308123
HIPK2	4314.384803	-0.765520528	0.139097224	-5.50349251	3.72E-08	1.72E-06
HIST1H1C	531.6195452	0.754570617	0.223737219	3.372575295	0.000744687	0.00445582
HIST1H1E	8.947102074	-0.980167584	0.284848027	-3.441019394	0.000579527	0.00367874
HIST1H4E	8.978304854	-0.732652205	0.238130991	-3.076677265	0.002093218	0.01001281
HIST1H4J	75.18698517	0.736908275	0.232496062	3.169551642	0.001526743	0.00781661
HK3	157.4403334	1.00682766	0.244034012	4.125767761	3.69E-05	0.00041887
HKDC1	80.99017215	0.953694706	0.241825759	3.943726715	8.02E-05	0.00076991
HLA-A	22413.01027	0.940084623	0.153381275	6.129070341	8.84E-10	7.75E-08
HLA-B	26309.22572	1.104711189	0.173614706	6.363004683	1.98E-10	2.27E-08
HLA-C	18134.87465	0.995343725	0.177403248	5.610628541	2.02E-08	1.05E-06
HLA-DMA	1668.268418	0.647792781	0.163044877	3.97309498	7.09E-05	0.00069689
HLA-DMB	2258.539372	0.672998607	0.181371997	3.710598211	0.00020677	0.00164579
HLA-DOA	950.0154101	0.631430878	0.189881664	3.325391535	0.000882944	0.00508839
HLA-DPA1	7232.934218	0.784345642	0.183039331	4.28512079	1.83E-05	0.00024259
HLA-DPB1	3414.12542	0.764561053	0.181045402	4.223034911	2.41E-05	0.00029992
HLA-DQA1	1310.400814	0.810720667	0.244649929	3.313798903	0.000920377	0.00524047
HLA-DQA2	207.1412143	0.991707211	0.309179326	3.20754697	0.001338722	0.00703455
HLA-DQB1	1547.7339	0.887228919	0.250704209	3.53894704	0.000401726	0.00276843
HLA-DRA	18800.25003	0.762584325	0.196143421	3.887891431	0.000101119	0.00092526
HLA-DRB1	4506.851277	0.90603757	0.202111399	4.482862305	7.36E-06	0.00011731
HLA-DRB5	1506.378901	1.063655106	0.267881082	3.970624189	7.17E-05	0.00070165
HLA-F	821.5852519	0.667039639	0.184592572	3.613577894	0.000302001	0.00222491
HLA-G	22.03252592	0.753091689	0.24648028	3.055383135	0.002247732	0.01055383
HLA-H	1553.709138	0.974716461	0.152654663	6.385107687	1.71E-10	2.00E-08
HMGA2	60.85733859	0.969590933	0.362746738	2.672914272	0.007519546	0.02721831
HMGN5	146.0927473	-0.716424676	0.147359552	-4.861745771	1.16E-06	2.70E-05
HOMER2	109.0181216	0.767526618	0.151495422	5.066335404	4.06E-07	1.17E-05
HOXA11	52.9785746	-1.918528377	0.346032105	-5.544365249	2.95E-08	1.40E-06
HOXA2	84.81203336	-1.739406349	0.338765561	-5.134543031	2.83E-07	8.65E-06
HOXA3	118.2301597	-0.906498428	0.285530251	-3.174789449	0.001499453	0.00771419
HOXD8	126.0523045	-1.849469874	0.319284421	-5.792546557	6.93E-09	4.43E-07
HOXD9	112.1914745	-1.122931068	0.294988893	-3.806689326	0.000140839	0.00120913
HP	438.4765454	1.366916511	0.392138153	3.485803409	0.000490661	0.00324171
HPCA	347.2853191	-1.28369527	0.27849665	-4.609374194	4.04E-06	7.23E-05
HPCAL4	586.4123199	-1.636607353	0.284357319	-5.755460618	8.64E-09	5.31E-07
HPN	20.66625076	-0.650829346	0.266717887	-2.440141355	0.014681516	0.04554712
HR	403.3191534	-1.459162564	0.207248182	-7.040653147	1.91E-12	5.06E-10
HRASLS	191.3366491	-0.730496461	0.250360941	-2.917773271	0.003525406	0.01495581
HS6ST3	117.950915	-1.047340588	0.30727265	-3.408505727	0.000653197	0.00402746
HSD3B7	407.5009485	0.648795759	0.150388276	4.314137895	1.60E-05	0.00021811
HSPA12A	442.3522664	-0.859560788	0.177712879	-4.836795117	1.32E-06	2.98E-05
HSPA1B	1420.108087	0.588618843	0.205307396	2.867012363	0.004143668	0.01698188
HSPA7	168.9521473	0.882235626	0.233185147	3.78341261	0.000154693	0.00129889
HTR1D	30.26742949	1.000384732	0.300976037	3.323801925	0.000887992	0.0051091
HTR7	20.04320292	0.646684289	0.204100911	3.168453715	0.001532521	0.00783859
HTRA1	9779.646436	0.662072781	0.132529297	4.99567111	5.86E-07	1.58E-05
HUNK	211.3974255	-0.614614973	0.213245845	-2.882189681	0.003949219	0.01635545
IBSP	376.4904164	0.88370554	0.349815358	2.526205669	0.011530196	0.03784368
ICAM1	1206.540916	0.718341093	0.218579885	3.28640073	0.001014765	0.0056677
ICAM3	234.2562371	0.802937682	0.113062805	7.101696113	1.23E-12	4.01E-10
IER3	433.4766793	0.683014671	0.203080206	3.363275445	0.000770235	0.00457092
IFI27	1294.429319	1.011548371	0.248194179	4.075632935	4.59E-05	0.00049844
IFI30	3363.163154	0.761471887	0.191418348	3.978050675	6.95E-05	0.00068702
IFITM1	1301.061784	0.992090845	0.223121885	4.446407596	8.73E-06	0.00013382
IFITM3	7180.031823	0.583267545	0.176852016	3.298054259	0.000973573	0.00548099

IGDCC3	240.6824233	-0.761594029	0.265356866	-2.870074709	0.004103748	0.0168786
IGF2	1022.297826	-1.062108886	0.279166444	-3.804572174	0.000142049	0.00121876
IGFBP1	48.64223833	0.644220158	0.230110111	2.79961691	0.005116329	0.02015772
IGFBP2	11138.43095	0.614044322	0.203953953	3.010700759	0.002606456	0.01182104
IGFBP3	7739.512242	1.432984568	0.257686909	5.560952134	2.68E-08	1.31E-06
IGFBP6	299.1875098	1.032708234	0.283131197	3.647454763	0.000264851	0.00199938
IGFBP7	16214.87336	0.607133206	0.167624507	3.621983542	0.000292353	0.00215961
IGLON5	480.3288239	-1.339855789	0.26972792	-4.967434554	6.78E-07	1.77E-05
IGSF9	56.32184491	-1.224672122	0.269969248	-4.536339338	5.72E-06	9.47E-05
IGSF9B	105.6412183	-1.205093363	0.240656764	-5.007519178	5.51E-07	1.50E-05
IL10	33.5957788	1.230142683	0.274059933	4.488590027	7.17E-06	0.00011465
IL10RA	721.4521195	0.843229221	0.191115031	4.412155425	1.02E-05	0.00015211
IL11	39.14182256	1.213886362	0.318856169	3.807002903	0.000140661	0.00120835
IL12RB1	51.13841252	0.651099807	0.186066202	3.499291106	0.000466497	0.00310592
IL15	39.40712767	0.997628175	0.214985133	4.640451936	3.48E-06	6.48E-05
IL15RA	69.155264	0.814159752	0.149432192	5.448355824	5.08E-08	2.17E-06
IL17D	658.7127791	-0.716733427	0.178713621	-4.010513711	6.06E-05	0.00061772
IL17RD	1051.926827	-0.588597503	0.158793457	-3.706686129	0.000209989	0.00166755
IL18	365.2262042	0.719109834	0.182587385	3.938442046	8.20E-05	0.00078433
IL18R1	21.67751943	1.31788415	0.266133727	4.951962176	7.35E-07	1.87E-05
IL1B	316.484827	0.711103314	0.253094907	2.809631062	0.004959832	0.01963106
IL1R1	395.8788498	1.117278968	0.244226011	4.574774661	4.77E-06	8.24E-05
IL1R2	71.07829962	1.802754994	0.343461648	5.248781055	1.53E-07	5.36E-06
IL1RAP	1733.167088	0.731117879	0.212580096	3.439258393	0.00058331	0.00369081
IL4R	568.6433139	0.670276997	0.144872108	4.626680759	3.72E-06	6.83E-05
IL6	94.51658458	1.181187123	0.335986595	3.515578123	0.000438798	0.0029689
INA	346.0685791	-2.306070237	0.341708618	-6.748645234	1.49E-11	2.78E-09
INHBA	28.39483777	1.610082939	0.294927888	5.459242763	4.78E-08	2.10E-06
INMT	95.40626371	0.780087053	0.293796466	2.655195494	0.007926246	0.02838433
INSM1	486.6590282	-0.84969191	0.307416189	-2.763979064	0.00571012	0.02203464
INTS4L1	26.52738011	-0.595822064	0.208702415	-2.854888206	0.004305198	0.01751349
IQCA1	111.1740185	-1.636730186	0.315088218	-5.194514089	2.05E-07	6.73E-06
IQGAP1	3310.384748	0.679543723	0.129497442	5.247545533	1.54E-07	5.38E-06
IQSEC3	95.68249618	-1.608021176	0.306590082	-5.244857122	1.56E-07	5.41E-06
IRAK3	238.034695	0.597095602	0.176674274	3.379640903	0.000725806	0.00436753
IRF1	842.08019	0.818727103	0.157159569	5.20952754	1.89E-07	6.31E-06
IRF6	15.34408309	0.776691353	0.263582359	2.946674259	0.003212114	0.01395777
IRF8	412.5344052	0.648121354	0.174245213	3.719593461	0.000199544	0.00160498
IRX3	77.19263109	1.180957149	0.281874301	4.189658807	2.79E-05	0.00033491
ISM1	29.16925191	-0.756222427	0.254274376	-2.97404103	0.002939058	0.01298469
ITGA11	157.7086092	0.834998272	0.19718935	4.234499835	2.29E-05	0.00028868
ITGA5	1851.746974	0.661950331	0.166359196	3.979042609	6.92E-05	0.00068506
ITGB1	5336.461093	0.641412662	0.114361534	5.60863991	2.04E-08	1.05E-06
ITGB2	3307.152473	0.702772498	0.175277503	4.009484886	6.09E-05	0.00061958
ITGB3	202.8926412	0.631923686	0.208444246	3.031619723	0.002432454	0.01121831
ITK	41.04739032	0.959175496	0.258421099	3.71167641	0.000205891	0.00164069
ITPR3	248.4919725	0.633638335	0.236003866	2.684864219	0.007255928	0.02648702
JAK3	241.978119	0.602626295	0.220335061	2.73504494	0.006237176	0.02359429
JAKMIP1	109.6591296	-1.286703199	0.270762462	-4.752147656	2.01E-06	4.15E-05
JAKMIP3	51.41966562	-0.975361448	0.234724237	-4.155350392	3.25E-05	0.00037847
JPH1	79.15184217	1.297000604	0.256260741	5.06125363	4.17E-07	1.18E-05
JPH3	226.2052823	-2.439642117	0.325142455	-7.503302261	6.22E-14	3.17E-11
JPH4	728.3795788	-1.376085006	0.239727591	-5.740202861	9.46E-09	5.71E-07
JUNB	2844.552758	0.597041277	0.172256952	3.465992337	0.000528278	0.00343743
KAZALD1	63.50932531	-0.759873358	0.291276788	-2.608767298	0.009086901	0.03147595
KBTBD11	994.5524246	-0.597188115	0.159329328	-3.74813678	0.000178153	0.00145764
KCNA2	56.7203486	-0.59150934	0.192775198	-3.068389226	0.002152161	0.01022369
KCNA5	28.2173786	-1.373521776	0.321773313	-4.268600653	1.97E-05	0.00025708
KCNA6	523.651528	-1.046317512	0.202001389	-5.179754052	2.22E-07	7.09E-06
KCNAB1	117.7205622	-0.608880863	0.176975683	-3.440477539	0.000580689	0.00368269
KCNB1	55.58517176	-1.521239789	0.26557399	-5.72812039	1.02E-08	6.05E-07
KCNC1	228.5531435	-0.706948332	0.209839595	-3.368993977	0.000754431	0.00450628
KCND2	725.6325699	-1.110060762	0.260715845	-4.257741841	2.07E-05	0.00026709
KCNE1L	209.2703272	-0.905705968	0.367508678	-2.464447839	0.013722454	0.04325433
KCNE4	688.8868693	0.845059984	0.210372997	4.01696033	5.90E-05	0.00060546
KCNH2	937.041075	-0.647846209	0.17361137	-3.731588594	0.000190276	0.00154216
KCNH3	167.8003047	-0.652305469	0.200719469	-3.24983656	0.001154714	0.00624653
KCNH4	14.82730377	-0.715237757	0.287586549	-2.487034808	0.01288128	0.0412165
KCNH8	105.2495616	-1.81459409	0.298976216	-6.069359348	1.28E-09	1.05E-07
KCNIP2	227.6108695	-1.464881212	0.242372356	-6.04392859	1.50E-09	1.21E-07
KCNIP3	361.7118448	-1.490780422	0.192431812	-7.747058072	9.40E-15	6.16E-12
KCNJ10	2917.192813	-0.762411965	0.188995777	-4.034015886	5.48E-05	0.00057166
KCNJ11	116.3272688	-0.765696967	0.228183897	-3.355613507	0.000791892	0.00467124
KCNJ15	16.42220654	0.783470025	0.272267815	2.877571207	0.004007495	0.01655196

KCNJ4	104.2426189	-0.739028873	0.273999595	-2.697189655	0.006992743	0.02581388
KCNJ5	82.06360022	0.860561203	0.218640745	3.935959887	8.29E-05	0.00079108
KCNJ9	147.7331209	-1.680885775	0.260870784	-6.44336536	1.17E-10	1.50E-08
KCNK1	162.1849123	-1.082041594	0.282092529	-3.835768345	0.000125172	0.0011078
KCNK2	221.2854987	-0.812036789	0.284693835	-2.852316035	0.004340193	0.017609
KCNK3	101.433178	-0.875207643	0.247904385	-3.530424208	0.000414894	0.00283223
KCNK4	19.88320392	-1.16532295	0.257452736	-4.526356828	6.00E-06	9.83E-05
KCNMB1	232.8202482	0.610647735	0.184962559	3.301466731	0.000961807	0.00542253
KCNN1	117.9905609	-1.061238849	0.213797921	-4.963747275	6.91E-07	1.80E-05
KCNN3	169.4738337	-0.881490517	0.177786721	-4.958134729	7.12E-07	1.83E-05
KCNN4	101.1492961	1.195621953	0.279437951	4.278667051	1.88E-05	0.00024754
KCNQ1OT1	56.02235252	-1.017875914	0.230282173	-4.420124668	9.86E-06	0.00014772
KCNQ5	159.1844326	-1.074713301	0.273246896	-3.933121721	8.38E-05	0.00079857
KCNS1	70.40390275	-1.156292473	0.30244856	-3.823104569	0.000131782	0.00114856
KCNS3	122.5594401	-0.900976079	0.221712541	-4.063712749	4.83E-05	0.00051847
KCNT2	142.0221058	-0.682552298	0.215255704	-3.17089064	0.001519723	0.00779517
KCTD14	133.496657	0.886326099	0.222666158	3.980515528	6.88E-05	0.00068329
KCTD16	36.5130943	-1.555634072	0.252991348	-6.148961545	7.80E-10	6.97E-08
KCTD4	49.56247773	-1.157177225	0.313730088	-3.68844835	0.000225626	0.00176124
KDELR3	246.792698	1.295495081	0.233612456	5.545488039	2.93E-08	1.40E-06
KDM4D	61.50164742	-0.640996041	0.109537538	-5.851839028	4.86E-09	3.26E-07
KDM5B	1238.786961	-0.668765839	0.138211601	-4.838709865	1.31E-06	2.95E-05
KDM6B	642.9463517	-0.597899435	0.140997742	-4.240489441	2.23E-05	0.00028211
KIAA0319	98.9531007	-0.652817631	0.230378301	-2.833676728	0.004601586	0.01848424
KIAA0513	796.6763488	-0.637951672	0.165924093	-3.844840491	0.000120631	0.00107243
KIAA0895L	743.3059529	-0.623471585	0.155508523	-4.009243823	6.09E-05	0.00061958
KIAA1045	211.8159345	-1.444707215	0.289491284	-4.990503324	6.02E-07	1.61E-05
KIAA1107	134.9616259	-0.695883858	0.17977276	-3.870908237	0.000108431	0.00098237
KIAA1199	432.5894745	1.054290288	0.239795104	4.396629747	1.10E-05	0.00016045
KIAA1211	641.551345	-0.841732775	0.180619747	-4.660247792	3.16E-06	6.01E-05
KIAA1244	147.7562828	-0.761576982	0.242792651	-3.136738197	0.001708386	0.00853387
KIAA1324L	222.0593386	-0.870663797	0.181780473	-4.78964424	1.67E-06	3.60E-05
KIAA1549	836.7908578	-0.977215608	0.164106953	-5.954748339	2.60E-09	1.92E-07
KIAA1644	132.1174419	-1.650323932	0.316700477	-5.210992885	1.88E-07	6.30E-06
KIAA1755	415.5043283	-0.787888545	0.173402772	-4.54369059	5.53E-06	9.20E-05
KIAA1875	22.37958387	-0.79553549	0.224998222	-3.535741229	0.000406633	0.00279246
KIF18B	365.7136068	-0.631785756	0.201405841	-3.136879019	0.001707566	0.00853287
KIF1A	5150.628719	-1.452326671	0.184544304	-7.869799486	3.55E-15	3.49E-12
KIF21B	972.6412614	-1.368133846	0.228048003	-5.999323947	1.98E-09	1.55E-07
KIF26A	134.8464695	-1.611810498	0.267796531	-6.018787816	1.76E-09	1.39E-07
KIF5A	1626.660361	-2.027977903	0.273647642	-7.410909485	1.25E-13	5.39E-11
KIF5C	2361.598213	-1.040811134	0.158398995	-6.570819048	5.00E-11	7.83E-09
KIF6	64.66017244	-1.31150197	0.292682216	-4.480975941	7.43E-06	0.00011808
KIRREL3	191.997616	-0.83970843	0.266861076	-3.146612623	0.001651736	0.0083105
KIT	315.0069614	-1.25116762	0.250005331	-5.004563765	5.60E-07	1.52E-05
KLF5	64.25859798	0.672685715	0.217952809	3.086382408	0.002026081	0.00976977
KLHDC7B	28.22396101	0.710061063	0.217184528	3.26939064	0.001077794	0.0059185
KLHDC8A	2669.687975	0.824896696	0.206503942	3.994580869	6.48E-05	0.00065209
KLHL23	245.1470968	-0.584339114	0.149481996	-3.909093597	9.26E-05	0.00086379
KLHL32	169.2711131	-0.853973942	0.241560219	-3.535242453	0.000407401	0.00279355
KLHL35	41.45922385	-2.084240481	0.31830307	-6.547974797	5.83E-11	8.82E-09
KLRK1	51.63832731	-0.843880676	0.301534161	-2.798623791	0.005132089	0.02020824
KMO	55.44209874	0.628986118	0.236453675	2.660081806	0.007812167	0.0280488
KNDC1	388.4418571	-0.681058507	0.229548939	-2.966942517	0.003007772	0.01322459
KRT18	41.22254754	0.925126343	0.249635501	3.705908574	0.000210634	0.00166831
KSR1	771.3644763	-0.582357625	0.142576447	-4.084528948	4.42E-05	0.00048586
KY	67.18364096	-1.57419054	0.328679225	-4.789443389	1.67E-06	3.60E-05
KYNU	90.92102994	0.773448915	0.182005785	4.249584242	2.14E-05	0.0002752
L1CAM	535.3060949	-1.776334254	0.331598061	-5.356889752	8.47E-08	3.31E-06
LAIR1	954.7746609	0.735046082	0.175230685	4.194733839	2.73E-05	0.00032893
LAMA3	43.08681859	0.852563622	0.294870157	2.891318783	0.003836288	0.01597554
LAMB3	175.3514759	0.876323228	0.20163961	4.345987507	1.39E-05	0.00019411
LANCL3	14.54483062	1.002340849	0.223313895	4.488484014	7.17E-06	0.00011465
LAPTM5	9782.319737	0.705466984	0.17289763	4.080258275	4.50E-05	0.00049134
LCK	29.46418666	0.642909349	0.268071084	2.398279365	0.016472297	0.04988027
LCP1	1460.679153	0.613796837	0.161170509	3.808369418	0.000139886	0.00120273
LDB3	69.7641662	-0.907711388	0.263791562	-3.441017524	0.000579531	0.00367874
LEPREL2	354.7768363	0.774876134	0.183449843	4.223912769	2.40E-05	0.00029929
LGALS1	8221.953027	0.809645489	0.167267607	4.840420113	1.30E-06	2.95E-05
LGALS3	4497.479126	1.333535651	0.23060492	5.782771902	7.35E-09	4.66E-07
LGALS3BP	8786.698094	0.674022308	0.132263606	5.096052711	3.47E-07	1.02E-05
LGI3	484.530797	-1.509775925	0.286921073	-5.261990373	1.43E-07	5.05E-06
LGI4	398.0237959	-0.828067351	0.235909545	-3.510105328	0.000447929	0.00300996
LGR5	85.94831548	-2.424683593	0.324900054	-7.462859931	8.47E-14	4.02E-11

LHFPL4	746.0509195	-0.918915309	0.256614557	-3.580916526	0.000342391	0.00244524
LHX2	520.4825941	-0.619257026	0.185384487	-3.340392911	0.000836599	0.00486408
LHX6	54.45488729	-0.708066652	0.244361696	-2.8976172	0.003760092	0.01574753
LIF	572.4049261	1.255485358	0.302008404	4.157120601	3.22E-05	0.00037715
LILRA5	31.44388141	1.096678482	0.299692211	3.659349293	0.000252856	0.00192894
LILRA6	79.64887079	0.884075046	0.247100868	3.577790131	0.000346511	0.00246571
LILRB2	129.6066506	0.84573457	0.235825143	3.586278204	0.000335431	0.00240177
LILRB3	59.61905313	0.864956057	0.21372009	4.04714436	5.18E-05	0.00054759
LIMS2	391.3198919	-0.992170645	0.207179922	-4.788932416	1.68E-06	3.60E-05
LINGO1	1544.638665	-0.68142033	0.220171953	-3.09494611	0.001968487	0.00954893
LIPE	219.2287731	-0.713265986	0.179552551	-3.972463675	7.11E-05	0.00069824
LIPG	333.690286	0.605067347	0.224831751	2.69120062	0.007119537	0.02612078
LNPEP	79.42705785	0.626686701	0.13500644	4.641902269	3.45E-06	6.45E-05
LOX	1047.137512	1.390416241	0.245021111	5.674679366	1.39E-08	7.77E-07
LOXL1	372.4481945	1.353512414	0.244621562	5.53308712	3.15E-08	1.47E-06
LPAR4	50.71662157	-1.831275865	0.276453929	-6.624162915	3.49E-11	5.79E-09
LPHN3	1331.169823	-0.609624432	0.15926507	-3.82773469	0.000129328	0.00113581
LPIN3	73.91518031	0.681853299	0.261361317	2.608853165	0.009084621	0.03147595
LPPR1	787.1774553	-1.659563281	0.329026298	-5.043862115	4.56E-07	1.27E-05
LRP6	746.7746311	-0.631389085	0.126801643	-4.979344682	6.38E-07	1.69E-05
LRRC10B	53.02127572	-1.30720882	0.280891289	-4.65378911	3.26E-06	6.16E-05
LRRC16B	130.6936751	-0.795159337	0.226006043	-3.51831007	0.000434305	0.00294574
LRRC25	284.768891	0.64063563	0.181141394	3.536660588	0.00040522	0.00278693
LRRC2	191.287902	0.787247652	0.282951277	2.782272834	0.005397964	0.02104441
LRRC37B	222.80456	-0.661407116	0.102829526	-6.432073959	1.26E-10	1.59E-08
LRRC3B	177.9023102	-0.797731219	0.247397154	-3.224496348	0.001261945	0.0066976
LRRC4	550.2003442	-0.967187867	0.174399726	-5.545810716	2.93E-08	1.40E-06
LRRC4C	390.6764993	-0.656871427	0.168305241	-3.902857822	9.51E-05	0.0008798
LRRC55	1040.053378	-0.580231431	0.238669483	-2.431108595	0.015052702	0.04643696
LRRC7	33.49781215	-1.656423506	0.307586679	-5.38522511	7.24E-08	2.90E-06
LRRC8E	29.13840457	1.73356397	0.313724632	5.525750276	3.28E-08	1.53E-06
LRRN2	1079.114898	-1.065594048	0.233492023	-4.563727847	5.03E-06	8.61E-05
LRRTM1	114.4397965	-1.638197222	0.244779163	-6.692551783	2.19E-11	3.82E-09
LRRTM4	125.4685182	-1.867582111	0.272327205	-6.857860985	6.99E-12	1.50E-09
LSAMP	1461.479926	-1.052099643	0.154328683	-6.817265736	9.28E-12	1.91E-09
LTA	6.795620685	-0.607857261	0.219711335	-2.766617666	0.005664114	0.02189594
LTBP2	532.0477062	1.085064993	0.20554964	5.278846485	1.30E-07	4.70E-06
LUM	718.2181261	1.366432159	0.272816412	5.008614209	5.48E-07	1.50E-05
LUZP2	613.3386004	-1.736308006	0.354404343	-4.899228917	9.62E-07	2.31E-05
LY6H	383.3004011	-1.137525221	0.315060308	-3.610499933	0.000305607	0.00224188
LY75	168.4292301	0.86311315	0.198295163	4.352668696	1.34E-05	0.00019038
LY86	560.0862484	0.814013506	0.187324584	4.345470776	1.39E-05	0.00019418
LY96	255.635948	1.040429083	0.219874191	4.731929094	2.22E-06	4.52E-05
LYN	1014.147916	0.619811583	0.144555225	4.287714826	1.81E-05	0.00024096
LYZ	1267.37514	0.845339711	0.245448165	3.444066121	0.000573036	0.0036476
LZTS1	1275.011703	0.632454575	0.209204867	3.023135089	0.002501705	0.01148381
MACROD2	66.19026387	-0.715511567	0.257821182	-2.775224136	0.005516368	0.02140295
MAFF	467.0353743	0.591919956	0.168943022	3.503666205	0.0004589	0.0030687
MAG	1306.948231	-1.102444411	0.367186969	-3.002406142	0.002678546	0.01207407
MAGEE1	199.3994861	-0.994677037	0.198061491	-5.022061751	5.11E-07	1.42E-05
MAGEL2	78.61039854	-2.013067793	0.227549007	-8.846743909	9.01E-19	2.48E-15
MAGI1	767.5466779	-0.652008469	0.158604692	-4.110902777	3.94E-05	0.00044276
MAN1A1	322.6344206	0.779327174	0.197742418	3.941122917	8.11E-05	0.00077685
MAN1C1	1734.142461	0.710806454	0.191299487	3.715673596	0.000202663	0.00162437
MAN2A1	372.4113121	0.710384209	0.157374446	4.513974338	6.36E-06	0.0001028
MAN2B1	2066.641814	0.635535985	0.114476018	5.551695436	2.83E-08	1.37E-06
MAP1A	4309.033003	-0.684156891	0.148127187	-4.61871252	3.86E-06	7.03E-05
MAP2	7183.496175	-0.617701062	0.168144694	-3.673628042	0.000239131	0.00184676
MAP3K8	190.2710464	0.649921852	0.205517782	3.162363111	0.001564943	0.00796183
MAP3K9	29.49537306	-0.948765147	0.247988481	-3.825843621	0.000130325	0.00114238
MAP7D2	85.9116808	-1.057139492	0.314148309	-3.365096871	0.000765168	0.00455067
MAPK13	122.0517759	0.778103131	0.191358165	4.066213386	4.78E-05	0.00051553
MAPK8IP1	3741.934144	-0.598938573	0.143550582	-4.172317273	3.02E-05	0.00035587
MAPK8IP2	713.9511227	-0.802034631	0.18803975	-4.265239828	2.00E-05	0.0002595
MAPK8IP3	1939.492112	-0.641977847	0.148359668	-4.327172308	1.51E-05	0.00020723
MAPT	3676.907241	-0.786239558	0.17271463	-4.552246423	5.31E-06	8.92E-05
4-Mar	57.21347201	-1.149654503	0.284534743	-4.04047144	5.33E-05	0.00055869
9-Mar	2786.982409	-1.970751904	0.336755364	-5.852176725	4.85E-09	3.26E-07
MARCKSL1	10395.58231	-0.689830576	0.151337538	-4.558225184	5.16E-06	8.74E-05
MAST1	427.6128006	-1.32116467	0.218859705	-6.036582519	1.57E-09	1.26E-07
MATN1	7.446834338	-0.703951161	0.198598162	-3.544600591	0.000393208	0.00272393
MBD6	1456.041371	-0.847055664	0.194941979	-4.345168088	1.39E-05	0.00019424
MBP	21868.22731	-1.155968532	0.340267039	-3.397239223	0.000680694	0.00415604
MCF2L2	72.73325997	-1.653150582	0.24970214	-6.620490239	3.58E-11	5.87E-09

MCM10	155.0612486	-0.627908931	0.206718119	-3.037512787	0.002385393	0.01105312
MCOLN2	23.32578202	0.939747863	0.248978619	3.774411896	0.000160386	0.00134178
MDFI	1072.731457	-0.886662753	0.21982372	-4.033517198	5.49E-05	0.00057244
MDM4	1314.28568	-0.920409148	0.184607204	-4.985770472	6.17E-07	1.64E-05
MEFV	16.42444728	0.774786007	0.212209685	3.651039814	0.000261181	0.00197685
MEG3	1066.008533	-0.840775445	0.318937497	-2.636176218	0.008384618	0.02961733
MEGF11	364.1703156	-0.890284533	0.230278871	-3.866114715	0.000110583	0.00099826
MEGF6	157.692759	-1.391998369	0.282642902	-4.924936594	8.44E-07	2.08E-05
MET	485.3173855	1.804404398	0.32094518	5.622157643	1.89E-08	9.98E-07
METTL1	996.2529222	-1.261025552	0.273578884	-4.609367267	4.04E-06	7.23E-05
METTL7B	3190.953034	0.699696765	0.263880066	2.651571127	0.008011824	0.02858453
MEX3A	974.2799585	-1.29949946	0.200954876	-6.466623188	1.00E-10	1.31E-08
MEX3B	164.7138944	-1.37489197	0.225884328	-6.086708101	1.15E-09	9.56E-08
MFSD4	214.7833923	-0.934901926	0.205005023	-4.56038546	5.11E-06	8.68E-05
MFSD7	76.2738822	0.825349193	0.192654262	4.284095177	1.83E-05	0.00024259
MIAT	802.7892508	-1.286736256	0.252347817	-5.099058395	3.41E-07	1.01E-05
MICAL3	1273.820882	-0.595383446	0.140328337	-4.242788442	2.21E-05	0.00028111
MIR155HG	44.74325517	0.716996978	0.217906451	3.290388952	0.00100049	0.00560394
MIR17HG	31.1544758	-1.010542356	0.225363957	-4.484046036	7.32E-06	0.00011679
MKRN3	134.2267966	-1.09141666	0.191984917	-5.68490836	1.31E-08	7.41E-07
MLKL	104.5775241	0.66370749	0.141167808	4.701549887	2.58E-06	5.13E-05
MLLT11	2582.571459	-0.603423975	0.154442419	-3.907112945	9.34E-05	0.00086854
MMD2	177.0941383	-1.267155541	0.263727035	-4.804799557	1.55E-06	3.40E-05
MME	61.15988972	2.127145969	0.3690814	5.763351848	8.25E-09	5.09E-07
MMP15	660.8956105	-0.779041788	0.184173471	-4.229934867	2.34E-05	0.00029272
MMP17	206.1018181	-1.341145194	0.252154432	-5.318745281	1.04E-07	3.92E-06
MMRN1	167.5059888	0.596806604	0.173781478	3.434235988	0.000594227	0.00374096
MN1	230.8342338	-1.042241249	0.227400439	-4.583286012	4.58E-06	7.99E-05
MNDA	334.7731641	0.74598071	0.191045388	3.904730279	9.43E-05	0.00087419
MOBP	947.8838128	-1.21750673	0.385357804	-3.1594189	0.001580841	0.00802195
MOG	1036.227164	-1.086396277	0.363357015	-2.98988662	0.00279081	0.0124658
MOXD1	1608.053057	0.956254109	0.291389175	3.281707734	0.001031805	0.00573957
MPEG1	333.6660657	0.6786052	0.207808294	3.265534719	0.001092576	0.00596678
MPP2	688.0316562	-0.601843267	0.135295779	-4.448352119	8.65E-06	0.00013291
MPP4	10.92863199	0.672581471	0.277592573	2.422908743	0.015396794	0.04730759
MPPED1	44.05170051	-1.889779675	0.355417768	-5.317065844	1.05E-07	3.93E-06
MPZL2	126.5488569	0.659375291	0.179884592	3.665546255	0.000246811	0.00189016
MR1	244.2205046	0.70853222	0.133141819	5.321635426	1.03E-07	3.88E-06
MRC1	299.9083612	1.2090827	0.333065189	3.630168327	0.000283236	0.00210925
MRC2	4356.705287	0.625202175	0.176462956	3.542965556	0.000395654	0.00273343
MREG	786.2627724	0.823826895	0.198228535	4.155945031	3.24E-05	0.00037781
MS4A14	64.50522747	0.864442334	0.203719304	4.243301036	2.20E-05	0.00028092
MS4A4A	692.0667088	0.798649041	0.237911027	3.356923177	0.00078815	0.00465316
MS4A6A	1845.427328	0.642034616	0.210818365	3.045439684	0.002323404	0.01083153
MS4A7	1018.343007	0.610297297	0.201074807	3.035175341	0.002403959	0.01112417
MSH5	294.7443833	-0.810988159	0.181354343	-4.471843044	7.75E-06	0.00012174
MSR1	1820.931128	0.785856608	0.193335157	4.064737212	4.81E-05	0.00051701
MST1	163.2177874	-0.643221091	0.20693623	-3.108305839	0.001881632	0.00919241
MST1R	6.517656246	-1.18720524	0.243768785	-4.870210278	1.11E-06	2.61E-05
MSTN	406.0271072	-1.259066167	0.344183195	-3.658127954	0.000254064	0.00193387
MT1E	1114.217491	0.778581598	0.20505807	3.796883481	0.000146527	0.00124499
MT1L	85.66475605	1.116597746	0.231656862	4.820050391	1.44E-06	3.19E-05
MT1M	374.2265871	0.646238013	0.22255468	2.903726914	0.003687496	0.01550484
MT1X	2975.175	0.861119716	0.216179451	3.983356007	6.79E-05	0.00067713
MT2A	10864.89719	1.434634972	0.198984249	7.209791629	5.60E-13	2.03E-10
MT3	17288.70189	1.109462025	0.241173872	4.600257956	4.22E-06	7.50E-05
MTMR7	106.6262432	-0.908054392	0.187610084	-4.840115045	1.30E-06	2.95E-05
MTSS1	1576.915061	-0.751828216	0.16254139	-4.625457034	3.74E-06	6.86E-05
MTUS2	17.89479787	-1.449654206	0.279273074	-5.190812655	2.09E-07	6.83E-06
MUM1L1	37.56276547	-1.435175195	0.324071917	-4.428570079	9.49E-06	0.00014252
MURC	21.63043588	-1.006518124	0.203991334	-4.934121983	8.05E-07	2.00E-05
MVP	1753.990968	0.729462806	0.143846027	5.071136263	3.95E-07	1.14E-05
MXRA8	1287.088351	0.649473757	0.191858015	3.385179183	0.000711318	0.00429915
MYADM	1396.579081	0.640310799	0.139153278	4.601478355	4.20E-06	7.48E-05
MYADML2	12.3351707	-0.63445185	0.205407551	-3.088746487	0.002010029	0.00971279
MYB	27.89648471	-1.069300194	0.279522544	-3.825452425	0.000130532	0.00114274
MYCN	258.1449604	-1.045860451	0.237626733	-4.401274379	1.08E-05	0.00015789
MYH14	585.3785613	-1.002122715	0.229738663	-4.362011611	1.29E-05	0.00018436
MYH15	27.98633816	-0.643556604	0.24749679	-2.60026243	0.009315249	0.03205713
MYH3	47.32687589	-0.735025059	0.195188326	-3.765722434	0.000166068	0.00138009
MYH7B	23.30798329	-1.342639663	0.258901303	-5.185913121	2.15E-07	6.94E-06
MYL12A	2400.904587	0.662131082	0.132613291	4.992946604	5.95E-07	1.60E-05
MYL12B	3959.875551	0.620718322	0.122864876	5.052040429	4.37E-07	1.23E-05
MYL9	1401.205211	0.685643601	0.185749189	3.691233351	0.000223169	0.00174702

MYLK3	17.75499822	-0.960581571	0.274737658	-3.496359313	0.000471653	0.00313267
MYO16	160.8166236	-0.750221692	0.215728033	-3.477627285	0.000505873	0.00331673
MYO1E	734.2136261	0.721717663	0.122664414	5.88367597	4.01E-09	2.80E-07
MYO1G	158.0095888	0.732585977	0.219244846	3.341405689	0.000833553	0.00484842
MYO7B	69.78468241	-1.709758191	0.328039346	-5.212052191	1.87E-07	6.28E-06
MYOF	1080.451945	0.676532886	0.168620042	4.012173638	6.02E-05	0.00061466
MYOM1	80.20074919	-0.786202958	0.219040401	-3.589305693	0.00033156	0.00238024
MYOT	40.86920214	-1.292628982	0.319601538	-4.044501752	5.24E-05	0.00055126
MYOZ3	65.72712605	-1.00741557	0.223583171	-4.505775479	6.61E-06	0.00010644
MYT1	370.7003251	-1.637468505	0.347063864	-4.718061061	2.38E-06	4.78E-05
MYT1L	146.2302841	-2.195024242	0.383326359	-5.726254372	1.03E-08	6.08E-07
NAALADL1	40.2660025	0.662402266	0.160082267	4.137886587	3.51E-05	0.00040065
NALCN	286.9970803	-0.634701713	0.211330443	-3.003361479	0.002670151	0.01204807
NAMPT	7779.740296	0.938692358	0.225975914	4.153948709	3.27E-05	0.00038016
NAP1L2	216.5433697	-1.112277239	0.205764864	-5.405574186	6.46E-08	2.64E-06
NAP1L3	718.0648113	-0.697102793	0.131978627	-5.281936992	1.28E-07	4.65E-06
NAPB	814.7702656	-0.87778688	0.196240889	-4.47300706	7.71E-06	0.00012144
NAV1	2439.748546	-0.580271183	0.148381298	-3.910676004	9.20E-05	0.00086165
NAV3	248.8360446	-0.604358221	0.212244573	-2.847461358	0.004406944	0.01782203
NBEA	370.1055989	-0.758088265	0.157139512	-4.82430075	1.40E-06	3.14E-05
NCAM1	7914.454181	-0.854734109	0.137687286	-6.207792556	5.37E-10	5.10E-08
NCAM2	663.099797	-0.885375433	0.190948243	-4.636729919	3.54E-06	6.59E-05
NCF1	206.3962424	0.591986798	0.179940778	3.28989796	0.001002237	0.0056091
NCF1B	46.78530066	0.688682796	0.205992888	3.343235788	0.000828075	0.00482471
NCF1C	62.40379301	0.596893988	0.19950697	2.991845281	0.002772968	0.01239656
NCF2	387.4346238	0.580424463	0.165114105	3.515293032	0.000439269	0.00296917
NCF4	268.4171436	0.630179075	0.167770016	3.756207983	0.000172507	0.00142073
NDRG1	7191.498577	0.717265914	0.216118324	3.318857472	0.000903866	0.00517429
NECAB2	146.1127357	-1.631335738	0.235522172	-6.926463552	4.31E-12	1.02E-09
NEDD9	1325.3287	0.635677521	0.147417608	4.312086812	1.62E-05	0.00021949
NEFH	145.6053406	-0.998744312	0.256078456	-3.900149692	9.61E-05	0.00088706
NEFL	696.9133246	-1.15624434	0.400105049	-2.889851915	0.003854233	0.01602952
NEFM	244.3664282	-2.183958779	0.402137465	-5.430876172	5.61E-08	2.33E-06
NEGR1	214.0551035	-0.959981421	0.279233713	-3.437913751	0.000586214	0.00370408
NEK10	12.30665523	0.946906616	0.227902751	4.154871367	3.25E-05	0.00037894
NET1	451.2364478	-0.682397036	0.181957942	-3.750300913	0.000176622	0.00144856
NETO1	112.0288456	-2.766804302	0.350242911	-7.899672532	2.80E-15	2.96E-12
NEU4	515.1555452	-1.523381161	0.26516997	-5.744923381	9.20E-09	5.58E-07
NEURL1B	998.3173876	-0.652640408	0.154641522	-4.220343933	2.44E-05	0.00030279
NEURL	136.7397381	-1.192019775	0.250778135	-4.753284303	2.00E-06	4.14E-05
NEXN	139.0916314	0.618584116	0.179242216	3.451107275	0.000558292	0.00358359
NFE2L3	281.2514414	0.797178856	0.19023379	4.190521854	2.78E-05	0.00033422
NFKBIZ	329.3378795	0.963817977	0.222022913	4.341074377	1.42E-05	0.0001973
NGEF	459.8895775	-0.860398477	0.282675428	-3.04376819	0.002336351	0.01086612
NHLH1	129.707201	-1.793041024	0.361629007	-4.958233414	7.11E-07	1.83E-05
NINL	206.4392619	-0.964528074	0.21302905	-4.527683317	5.96E-06	9.79E-05
NKAIN1	278.2084134	-1.752021903	0.29845788	-5.870248433	4.35E-09	2.98E-07
NKAIN2	136.2406528	-1.22192523	0.310955375	-3.929583884	8.51E-05	0.00080706
NKAIN4	2257.84134	-1.636376399	0.252623543	-6.47752928	9.32E-11	1.27E-08
NKD1	157.3161019	-1.210055964	0.184489127	-6.558955436	5.42E-11	8.38E-09
NKPD1	11.98447854	-0.679595977	0.189997494	-3.576868111	0.000347736	0.00247315
NKX2-2	459.1980274	-1.174953912	0.217596663	-5.399687179	6.68E-08	2.71E-06
NLGN2	2628.342043	-0.69171485	0.12064429	-5.733506757	9.84E-09	5.91E-07
NLRC5	584.8366725	0.608394955	0.155831801	3.904177146	9.45E-05	0.00087561
NMNAT2	422.0838508	-1.099628448	0.245096364	-4.48651474	7.24E-06	0.00011558
NNAT	2035.555979	-1.49779235	0.417249869	-3.589677223	0.000331088	0.00237809
NNMT	2443.707667	1.585796187	0.290875472	5.45180443	4.99E-08	2.16E-06
NOD2	75.63051847	0.674685902	0.199545196	3.381118239	0.000721915	0.00434982
NOG	55.0294586	-0.706247183	0.210584787	-3.35374266	0.000797265	0.00469387
NOL4	203.1135479	-0.76065708	0.190969034	-3.983143574	6.80E-05	0.00067724
NOS2	632.3971203	2.682460278	0.379197112	7.074052494	1.50E-12	4.41E-10
NOTCH1	3394.007618	-0.621805382	0.145279929	-4.280050144	1.87E-05	0.00024636
NPAS2	499.8147617	0.64431909	0.185390794	3.475464323	0.00050997	0.00333861
NPM2	37.61095933	-1.811529698	0.298464078	-6.069506628	1.28E-09	1.05E-07
NPNT	1176.705885	0.801706411	0.278313795	2.880584519	0.003969385	0.01641427
NPPA	189.1545747	-1.946689181	0.365088246	-5.33210588	9.71E-08	3.70E-06
NPTX2	1352.19983	1.228080427	0.356296407	3.446794311	0.00056728	0.00362606
NPW	17.65567904	-1.147039514	0.279348633	-4.106121811	4.02E-05	0.00045018
NPY1R	38.16905175	-0.817634834	0.315643096	-2.590377688	0.009587068	0.03275502
NR4A3	166.1335561	0.672960376	0.188498581	3.57010844	0.000356833	0.00252091
NRG3	97.09180268	-0.734974863	0.272544062	-2.696719411	0.007002625	0.02584342
NRGN	1176.71354	-0.870747906	0.266993632	-3.261305901	0.001109003	0.00603484
NRIP2	63.80519157	-0.669039667	0.156798163	-4.266884595	1.98E-05	0.00025866
NRP1	1830.655114	1.119168424	0.147258139	7.600044599	2.96E-14	1.63E-11

NRP2	1987.993645	0.701392762	0.165153483	4.246914744	2.17E-05	0.00027772
NRSN1	273.0815329	-0.908378951	0.237428831	-3.825899941	0.000130295	0.00114238
NRXN1	1079.29875	-0.770719308	0.224293387	-3.436210568	0.000589912	0.0037218
NRXN2	2594.543691	-0.960278007	0.171689843	-5.593097358	2.23E-08	1.13E-06
NT5E	1545.067284	0.925049171	0.190037682	4.867714443	1.13E-06	2.63E-05
NTM	830.5648041	-0.643141458	0.164522421	-3.909141717	9.26E-05	0.00086379
NTN4	217.8814818	-0.93750804	0.261674641	-3.582724085	0.00034003	0.00243005
NTNG2	204.2842268	-0.713222765	0.203964048	-3.49680628	0.000470864	0.00312899
NTRK2	12656.95658	-0.902221825	0.22302944	-4.045303734	5.23E-05	0.0005498
NTSR2	217.9297104	-0.860857297	0.323868381	-2.658046749	0.007859499	0.02818932
NUDT10	259.2416746	-0.613634387	0.182767452	-3.35745988	0.000786622	0.00464813
NXN	426.7006355	-0.868886071	0.158877766	-5.468896561	4.53E-08	2.02E-06
NXPH1	534.1467589	-0.943354296	0.283886313	-3.323000277	0.000890548	0.00511584
NXPH3	415.9052652	-0.681837601	0.203845092	-3.344881134	0.000823178	0.00480228
OAS1	978.561347	1.159268597	0.247958166	4.6752588	2.94E-06	5.68E-05
OBSCN	90.77865331	-1.067677203	0.233984729	-4.563020875	5.04E-06	8.63E-05
OCIAD2	1339.996407	0.996814051	0.243653923	4.091106104	4.29E-05	0.00047473
ODF3B	51.47459574	1.013727501	0.228817071	4.430296647	9.41E-06	0.00014154
ODF3L2	5.223295808	-0.85747944	0.210177155	-4.07979374	4.51E-05	0.00049155
OLFM1	2495.266072	-1.142377517	0.234104677	-4.879772301	1.06E-06	2.50E-05
OLIG1	5626.208402	-1.039313422	0.224487905	-4.629707882	3.66E-06	6.76E-05
OLIG2	3181.242263	-0.947186955	0.22790508	-4.156058988	3.24E-05	0.00037781
OMG	762.1887696	-1.029570572	0.207809964	-4.954385012	7.26E-07	1.86E-05
ONECUT2	77.43683237	-1.321049058	0.31444419	-4.201219479	2.65E-05	0.00032192
OPCML	580.7609569	-1.1770007	0.294834836	-3.992067959	6.55E-05	0.00065748
OS9	14127.44282	-1.166215057	0.22585446	-5.163568862	2.42E-07	7.63E-06
OSBPL3	920.5121405	0.791428053	0.15087219	5.245685466	1.56E-07	5.41E-06
OSM	133.7635892	0.928666302	0.262703249	3.535039273	0.000407715	0.00279431
OTOS	62.31309645	-1.198914551	0.376611546	-3.183424843	0.001455439	0.00753565
OTUD7A	33.05302638	-0.873536116	0.208566082	-4.188294215	2.81E-05	0.00033634
P2RX7	360.3102147	-0.791252248	0.190696832	-4.14926792	3.34E-05	0.00038605
P2RY6	90.97141606	0.580607882	0.197636149	2.937761556	0.003305912	0.01424866
P2RY8	59.64943755	0.672652971	0.217997704	3.085596592	0.002031442	0.00978876
P4HA2	478.1742246	0.90320407	0.184592219	4.892969347	9.93E-07	2.37E-05
P4HA3	25.91592706	0.745728669	0.28677049	2.600437266	0.009310504	0.03205683
PABPC5	75.19267221	-1.662090697	0.36437359	-4.561501559	5.08E-06	8.66E-05
PACSIN1	373.5097571	-2.022651297	0.36544008	-5.534837057	3.12E-08	1.46E-06
PAIP2B	220.3184399	-1.296264824	0.211918346	-6.116812669	9.55E-10	8.21E-08
PALM	1522.052887	-0.797020846	0.163164878	-4.884757391	1.04E-06	2.45E-05
PAPPA	47.04950255	2.054819075	0.276517858	7.431053796	1.08E-13	4.94E-11
PAQR6	1558.304711	-0.640897173	0.180238329	-3.555831757	0.000376785	0.00264153
PARVB	451.8340758	0.665279462	0.144502625	4.603926475	4.15E-06	7.40E-05
PBX4	24.07687634	-0.692290261	0.182082765	-3.802063633	0.000143496	0.00122963
PCBP3	78.24507975	-1.008385583	0.226409875	-4.453805668	8.44E-06	0.0001303
PCDH15	220.1806268	-1.821119068	0.384515829	-4.736135506	2.18E-06	4.43E-05
PCDH20	72.94107858	-0.813510198	0.274766714	-2.960730525	0.003069103	0.0134457
PCDHA1	120.6051198	-0.810857256	0.257550645	-3.148341	0.001642	0.00827131
PCDHA2	59.17293488	-1.550323262	0.329654824	-4.702868422	2.57E-06	5.11E-05
PCDHA7	53.75121567	-0.818254566	0.295195803	-2.771904472	0.005572939	0.02157985
PCDHB11	20.78884014	-1.038802622	0.287802986	-3.609422664	0.000306879	0.00224652
PCDHB12	47.34612817	-0.817757514	0.227141919	-3.600205181	0.000317966	0.0023091
PCDHB13	50.1583477	-0.717283096	0.180319214	-3.977851722	6.95E-05	0.00068702
PCDHB15	99.54956318	-0.986362721	0.242252198	-4.071635792	4.67E-05	0.00050588
PCDHB18	13.38321728	-1.182834367	0.244434419	-4.839066338	1.30E-06	2.95E-05
PCDHB19P	14.59205673	-1.033118794	0.180674369	-5.718125938	1.08E-08	6.31E-07
PCDHB3	159.1881113	-0.87666561	0.300033149	-2.921895839	0.003479079	0.01480491
PCDHB4	164.0554817	-0.799314569	0.278315409	-2.871973828	0.004079167	0.01681267
PCDHGA11	35.89824111	-0.897794625	0.265236025	-3.384889463	0.000712069	0.0043018
PCDHGA12	69.43076177	-1.086261506	0.284546101	-3.817523781	0.000134798	0.00116893
PCDHGA1	70.0956571	-1.092541998	0.317132754	-3.445062001	0.000570928	0.00364233
PCDHGA3	33.9763384	-0.657656503	0.248674137	-2.64465179	0.008177502	0.02902728
PCDHGA5	30.38373011	-0.880018597	0.262809318	-3.348506071	0.000812485	0.00475805
PCDHGA6	47.55060178	-0.625854555	0.204711957	-3.057244743	0.002233818	0.01052802
PCDHGA7	54.76626784	-0.927371716	0.340345395	-2.724795843	0.006434126	0.02419302
PCDHGA8	6.06437368	-0.849006039	0.229893883	-3.693034488	0.000221594	0.00174008
PCDHGB1	257.6535575	-1.748072706	0.359797343	-4.858492535	1.18E-06	2.73E-05
PCDHGB3	79.93978353	-1.270527514	0.358382494	-3.545171808	0.000392357	0.00272393
PCDHGB4	14.4894944	-0.818730049	0.240939104	-3.398078753	0.000678609	0.00414883
PCLO	142.0238809	-1.250356504	0.297347727	-4.205031322	2.61E-05	0.00031905
PCOLCE2	276.7942159	0.807161317	0.261490938	3.086765926	0.002023469	0.00976402
PCOLCE	1804.498522	1.255746932	0.301334285	4.167288602	3.08E-05	0.00036288
PCP4L1	76.20560815	-0.85846124	0.316195649	-2.714968538	0.006628207	0.02474027
PCSK1	487.6454539	0.721633914	0.267647735	2.696207813	0.00701339	0.02585552
PCSK2	253.1424814	-2.489619663	0.374206326	-6.653066739	2.87E-11	4.88E-09

PCSK5	160.4097595	0.60991286	0.179878038	3.390702209	0.000697138	0.00423577
PCSK6	352.2389399	-0.897043817	0.298843505	-3.001717625	0.002684611	0.01209242
PCYT1B	519.5701837	-0.684885021	0.13802307	-4.962105382	6.97E-07	1.80E-05
PDCD1LG2	150.7169306	1.238257426	0.195655504	6.328763581	2.47E-10	2.70E-08
PDE10A	118.0261252	-1.287821525	0.25912843	-4.969819496	6.70E-07	1.75E-05
PDE11A	13.23036317	-1.323386788	0.24181412	-5.472744061	4.43E-08	1.99E-06
PDE2A	435.0286608	-1.430707704	0.226732582	-6.310110757	2.79E-10	2.93E-08
PDE4C	48.99061473	-1.0386004	0.287980228	-3.606498984	0.000310356	0.00226825
PDGFD	356.7110132	1.091351079	0.244068038	4.471503477	7.77E-06	0.00012174
PDGFRA	5974.361336	-1.65794695	0.335262699	-4.945217447	7.61E-07	1.92E-05
PDIA2	66.34672375	-1.33447772	0.293580999	-4.545518018	5.48E-06	9.15E-05
PDIA5	266.8715744	0.681490343	0.147442907	4.622062589	3.80E-06	6.94E-05
PDPN	5656.743601	0.680401138	0.205773772	3.306549374	0.000944527	0.00534261
PDYN	149.5515011	-1.894881007	0.381799762	-4.963023015	6.94E-07	1.80E-05
PDZD4	1719.66944	-0.814596715	0.153164534	-5.318442175	1.05E-07	3.92E-06
PDZD7	20.18892536	-0.803191983	0.233199818	-3.444222173	0.000572705	0.00364718
PDZK1	23.76070488	0.834547052	0.19278115	4.328986795	1.50E-05	0.0002062
PDZRN4	104.5702322	-1.227123532	0.265083288	-4.629199911	3.67E-06	6.77E-05
PERP	209.3003597	0.70282539	0.217463992	3.231916166	0.001229631	0.00655391
PEX5L	56.6122288	-0.963282213	0.301679457	-3.193065323	0.001407711	0.00732435
PFN2	8430.756015	-0.62775591	0.170698849	-3.677563809	0.000235472	0.00182362
PHACTR1	128.4122254	-0.89531471	0.195392947	-4.58212398	4.60E-06	8.03E-05
PHACTR3	446.6621286	-1.954486481	0.245389434	-7.964835523	1.65E-15	2.07E-12
PHF16	273.2801501	-0.581916657	0.142505566	-4.083466176	4.44E-05	0.00048731
PHGDH	3507.107913	-0.683358684	0.144464231	-4.73029675	2.24E-06	4.55E-05
PHYHIP	618.5054472	-1.56891515	0.26247584	-5.977369766	2.27E-09	1.70E-07
PHYHIPL	2040.828829	-0.822831707	0.172602742	-4.767199518	1.87E-06	3.94E-05
PIF1	103.5801434	-0.594916998	0.195146709	-3.048562808	0.002299388	0.01075236
PIK3AP1	615.0246502	0.671706494	0.180927906	3.712564347	0.00020517	0.00163662
PIK3CG	98.28315136	0.6149931	0.192841081	3.189118714	0.001427073	0.0074055
PIK3R5	314.9846842	0.690087236	0.16557624	4.167791447	3.08E-05	0.00036239
PIP4K2C	1146.808369	-0.605504982	0.170411081	-3.553201924	0.000380572	0.00266401
PIP5KL1	14.14507296	-0.66545448	0.199582711	-3.334229081	0.000855362	0.00494808
PITPNM3	212.1741769	-0.981540262	0.209225954	-4.691293039	2.71E-06	5.34E-05
PITX1	130.4729719	0.809465403	0.318456829	2.541837164	0.011027153	0.03661175
PKDCC	175.5777711	-0.607154919	0.169357931	-3.585039775	0.000337027	0.00241194
PKIB	469.1130559	0.845144492	0.232655548	3.632599777	0.00028058	0.00209287
PKNOX2	476.7578205	-0.605661269	0.148896812	-4.067657721	4.75E-05	0.00051338
PKP4	1306.549631	-0.58386857	0.134006808	-4.357006763	1.32E-05	0.00018745
PLA2G2A	1376.950364	2.591764808	0.448299398	5.781325644	7.41E-09	4.68E-07
PLA2G5	670.8530284	0.91986137	0.301141295	3.054583966	0.002253729	0.01057478
PLA2R1	77.38029178	1.58802928	0.310173639	5.119807356	3.06E-07	9.23E-06
PLAU	1012.510176	0.872855935	0.217448243	4.014085944	5.97E-05	0.00061106
PLAUR	694.9534326	1.046207764	0.178846551	5.849750863	4.92E-09	3.29E-07
PLBD1	252.2187987	0.704088431	0.196128658	3.589931425	0.000330765	0.00237702
PLCB1	493.7996434	-0.648824364	0.193275238	-3.356996844	0.00078794	0.00465316
PLCB4	106.0369691	-1.177888107	0.241847032	-4.870384794	1.11E-06	2.61E-05
PLCH2	116.1610786	-1.447130791	0.308465941	-4.69137949	2.71E-06	5.34E-05
PLCL1	207.6970595	-1.041166658	0.187926407	-5.540289282	3.02E-08	1.42E-06
PLCXD2	83.88988135	-1.638845325	0.235791291	-6.95040651	3.64E-12	8.79E-10
PLEK2	65.79485318	0.805270577	0.189848869	4.241640105	2.22E-05	0.0002817
PLEK	612.7872763	0.691235717	0.18076302	3.823988539	0.00013131	0.00114663
PLEKHA6	547.8781843	-0.788118964	0.213926417	-3.684065655	0.000229543	0.00178574
PLEKHG3	423.1817017	-0.778375465	0.166140496	-4.685043597	2.80E-06	5.47E-05
PLEKHG4	64.81689172	0.684484212	0.255631598	2.677619737	0.007414735	0.026931
PLEKHG4B	47.83839974	-1.043051003	0.363931203	-2.86606643	0.00415607	0.01702764
PLEKHH1	769.439725	-0.996354233	0.201488186	-4.944975956	7.62E-07	1.92E-05
PLEKHH2	257.4069616	-0.582783176	0.217626872	-2.677900806	0.007408515	0.02692263
PLIN1	28.60506829	-0.870434169	0.250851606	-3.469916666	0.00052062	0.00339241
PLIN2	1579.914497	0.587070142	0.207464328	2.829740166	0.004658582	0.01867504
PLIN4	42.6713584	-1.214463208	0.245799991	-4.940859452	7.78E-07	1.95E-05
PLIN5	50.80856768	-1.544738473	0.252305873	-6.122483226	9.21E-10	8.02E-08
PLOD2	2198.939497	0.690021683	0.151382102	4.558145739	5.16E-06	8.74E-05
PLP1	21150.21192	-1.05800209	0.285364704	-3.707543635	0.000209279	0.00166384
PLP2	1712.963547	0.868642645	0.186367609	4.660909962	3.15E-06	6.00E-05
PLS3	3357.214374	0.693671256	0.167038873	4.152753447	3.28E-05	0.00038182
PLSCR1	1296.734288	0.62417935	0.147900157	4.220275093	2.44E-05	0.00030279
PLXNB1	3359.090427	-1.044841401	0.139911914	-7.467851541	8.15E-14	4.01E-11
PMAIP1	112.0979845	0.871415163	0.253344818	3.439640761	0.000582487	0.00368899
PMP2	19357.96779	-0.631623478	0.188723204	-3.346824681	0.000817429	0.00477481
PNCK	130.5997766	-1.328410192	0.221916566	-5.986079442	2.15E-09	1.63E-07
PNMA3	111.5722657	-1.572343164	0.259700784	-6.054441341	1.41E-09	1.14E-07
PNMA6A	169.6767996	-0.784866639	0.207746476	-3.778002169	0.000158092	0.00132339
PNMAL2	310.310754	-0.791068576	0.172150256	-4.595221618	4.32E-06	7.65E-05

PODN	348.7055835	-0.971683424	0.225995035	-4.299578627	1.71E-05	0.00023043
PODNL1	100.4635766	1.718588769	0.281941625	6.095548206	1.09E-09	9.10E-08
PODXL2	1626.281087	-0.614860369	0.181453939	-3.388520376	0.000702708	0.00425833
POPDC3	49.6849052	-0.763985995	0.297027415	-2.572105999	0.010108194	0.03425485
POSTN	4798.818951	2.190516818	0.402158464	5.446899698	5.13E-08	2.18E-06
POU2F1	62.33032234	-0.623486485	0.139250065	-4.477459218	7.55E-06	0.00011935
PPFIA4	407.3433027	-0.850937058	0.202616066	-4.199751158	2.67E-05	0.00032286
PPIC	421.5878409	0.604174919	0.139574112	4.328703301	1.50E-05	0.00020621
PPM1E	393.840187	-0.740336014	0.166758062	-4.439581536	9.01E-06	0.00013691
PPP1R15B	1764.108011	-0.684408647	0.178494835	-3.834333063	0.000125905	0.00111357
PPP1R16B	521.2833904	-1.319771848	0.259760653	-5.0807227	3.76E-07	1.10E-05
PPP1R1A	162.6622122	-1.929458096	0.342113934	-5.639811497	1.70E-08	9.15E-07
PPP1R3B	570.4658108	0.732796805	0.147967869	4.952404925	7.33E-07	1.87E-05
PPP2R2C	399.0957867	-1.213523178	0.310564726	-3.907472664	9.33E-05	0.00086784
PPP2R3B	297.8505973	-1.010693891	0.181380746	-5.572222587	2.52E-08	1.25E-06
PPP4R4	43.74452888	-1.409583902	0.311388797	-4.526764979	5.99E-06	9.82E-05
PQLC3	347.7330894	0.742258903	0.148944934	4.983445124	6.25E-07	1.65E-05
PRCD	28.19369245	-0.747688167	0.246056931	-3.03867956	0.002376175	0.01101783
PRDM1	205.3454128	0.699288158	0.140795228	4.966703545	6.81E-07	1.77E-05
PRDM8	119.3416007	-1.01550149	0.28651408	-3.544333629	0.000393607	0.00272393
PRDX4	2759.086389	0.707425883	0.148604678	4.760455017	1.93E-06	4.03E-05
PRF1	134.4335244	1.034313969	0.261610881	3.95363513	7.70E-05	0.00074546
PRIMA1	330.4411039	-0.7735749	0.191977983	-4.029498007	5.59E-05	0.00057968
PRKCDBP	170.0184749	1.000102105	0.224270878	4.459348955	8.22E-06	0.00012741
PRKCG	73.78654322	-1.621467992	0.355175599	-4.565257287	4.99E-06	8.57E-05
PRKCZ	448.2089619	-1.312899494	0.220660304	-5.94986715	2.68E-09	1.95E-07
PRLR	14.10345137	1.355112021	0.288704137	4.693774166	2.68E-06	5.30E-05
PRMT8	38.50022304	-1.17130139	0.294288074	-3.980118446	6.89E-05	0.00068345
PRODH	546.6841307	-0.855738397	0.237989091	-3.595704293	0.000323515	0.00234204
PROM1	579.9108571	-1.169326804	0.248021779	-4.714613412	2.42E-06	4.85E-05
PROM2	28.55931866	-0.964152887	0.228803758	-4.213885706	2.51E-05	0.00030899
PRPH	161.5259958	-0.886187042	0.233134116	-3.801189879	0.000144003	0.00123168
PRR18	150.7582005	-0.676099706	0.200904637	-3.36527676	0.000764669	0.00454967
PRR24	504.8047123	0.733906746	0.152802159	4.802986748	1.56E-06	3.41E-05
PRRT1	226.8021176	-0.656791701	0.159953226	-4.106148504	4.02E-05	0.00045018
PRRT2	531.3495168	-0.761477393	0.211395943	-3.602138155	0.000315611	0.00229811
PRRT4	94.16025928	-0.880384437	0.289169703	-3.044525159	0.002330479	0.01085349
PRSS12	90.31987382	-1.338782029	0.316602225	-4.228593246	2.35E-05	0.00029394
PRSS35	155.216931	-1.171391832	0.310689377	-3.770298953	0.000163052	0.00135995
PRSS3	37.24318403	-0.671532055	0.264460482	-2.539252934	0.011108948	0.03682113
PSD	556.2317255	-1.104224306	0.241337156	-4.575442597	4.75E-06	8.24E-05
PSMB8	2048.436368	0.749331201	0.146297982	5.121951705	3.02E-07	9.17E-06
PSMB9	974.6962841	0.680205184	0.171547422	3.965114591	7.34E-05	0.00071551
PSPH	1878.389684	0.641511483	0.258340986	2.483196701	0.013020917	0.0415956
PSTPIP2	83.29893727	0.930832952	0.168165574	5.535217055	3.11E-08	1.46E-06
PTCH1	835.633899	-1.432795503	0.209060446	-6.853498741	7.21E-12	1.53E-09
PTCHD2	293.9560481	-0.758533524	0.23994038	-3.161341679	0.001570442	0.00798685
PTER	107.4604708	-0.625390481	0.239855199	-2.607366794	0.009124156	0.03156527
PTGDS	3933.586139	-0.672504621	0.257735834	-2.609278698	0.009073331	0.03146062
PTGER2	12.07539713	0.991265092	0.272313613	3.640159894	0.000272469	0.00204567
PTGES	110.5093186	1.1257239	0.275811335	4.081499773	4.47E-05	0.00048989
PTGS1	1072.083425	0.930482248	0.187763161	4.955616653	7.21E-07	1.85E-05
PTGS2	266.9378277	0.69311355	0.284110425	2.439592104	0.014703854	0.04558559
PTK6	18.50970184	-0.749962184	0.220423711	-3.402366208	0.000668051	0.00410067
PTPN14	63.46000833	0.841356513	0.199591928	4.215383473	2.49E-05	0.00030749
PTPN22	39.04197313	1.272413752	0.206862122	6.151023399	7.70E-10	6.96E-08
PTPN5	183.5698164	-1.354363527	0.338641472	-3.999402436	6.35E-05	0.00064118
PTPN7	137.5163583	0.624598903	0.165747552	3.768374833	0.000164314	0.00136799
PTPRC	707.4557855	0.723296605	0.186925625	3.869435267	0.000109088	0.00098703
PTPRD	1000.47865	-0.645640439	0.173075219	-3.730403711	0.000191173	0.00154851
PTPRT	208.0394289	-1.855259864	0.314952623	-5.890599822	3.85E-09	2.73E-07
PTPRU	340.3942853	0.946462868	0.242312586	3.905958354	9.39E-05	0.00087153
PTX3	940.9885758	0.882208324	0.298265906	2.957791369	0.003098518	0.01353708
PVRL1	861.5288936	-1.001615965	0.174930702	-5.725787137	1.03E-08	6.08E-07
PYGL	1631.451034	0.708731589	0.14419227	4.915184364	8.87E-07	2.18E-05
RAB11FIP4	547.7866547	-1.294188458	0.20690335	-6.255038678	3.97E-10	3.91E-08
RAB17	16.34162981	0.940246075	0.249986109	3.761193291	0.000169105	0.00140025
RAB27A	224.4405087	0.808779247	0.155239429	5.209882912	1.89E-07	6.31E-06
RAB32	646.5703793	0.642400567	0.173123833	3.710642014	0.000206734	0.00164579
RAB33A	278.1653628	-1.402012054	0.246570013	-5.686060682	1.30E-08	7.39E-07
RAB38	29.46032215	0.870738608	0.212002972	4.10720001	4.00E-05	0.00044882
RAB3A	434.4900633	-1.067854673	0.232187897	-4.59909705	4.24E-06	7.53E-05
RAB6B	2574.780276	-0.795753306	0.145114288	-5.483631655	4.17E-08	1.88E-06
RAB9B	35.52548392	-0.644977092	0.16750401	-3.850517324	0.000117869	0.0010518

RAC2	363.4921888	0.598858437	0.167533077	3.574568368	0.000350806	0.00248855
RAI2	100.9995419	-0.719291614	0.17988958	-3.998517385	6.37E-05	0.00064311
RALGPS1	407.9215551	-1.020926603	0.143559157	-7.111539413	1.15E-12	3.85E-10
RALYL	96.33302769	-1.010696624	0.308025545	-3.281210406	0.001033626	0.00574738
RANBP17	19.22835651	-2.471874937	0.375098871	-6.589929024	4.40E-11	6.97E-09
RAP1GAP2	400.7433048	-0.894838628	0.218532116	-4.094769425	4.23E-05	0.00046938
RAP1GAP	904.8129877	-0.673116264	0.184763681	-3.643120006	0.000269353	0.00202781
RAPGEF4	551.7968431	-0.920694704	0.203051632	-4.53428863	5.78E-06	9.55E-05
RAPGEFL1	257.3150266	-0.720185161	0.144252807	-4.992520955	5.96E-07	1.60E-05
RARRES1	144.3628551	1.443386506	0.277365793	5.203909577	1.95E-07	6.49E-06
RASD2	189.8606072	-0.653226921	0.195965693	-3.333373878	0.000857996	0.00496123
RASGEF1C	133.693957	-1.075065177	0.261438279	-4.112118471	3.92E-05	0.00044098
RASGRF1	129.8654977	-1.046959135	0.294990815	-3.549124519	0.000386514	0.00269575
RASGRP4	97.69844646	0.597423208	0.172584056	3.461636143	0.000536902	0.00347591
RASL10A	99.70416451	-0.997657382	0.223547894	-4.462835074	8.09E-06	0.00012563
RASL10B	471.1600035	-0.630640885	0.141660566	-4.451774436	8.52E-06	0.00013132
RASL11B	181.8612541	-1.765843444	0.299698632	-5.892063754	3.81E-09	2.72E-07
RASL12	1247.210685	-0.607147757	0.203274384	-2.9868385	0.002818786	0.01255815
RASSF8	819.6680364	0.617613187	0.133895836	4.612639251	3.98E-06	7.16E-05
RBM47	322.217322	0.811752383	0.176069881	4.610398879	4.02E-06	7.23E-05
RBMS1	772.98963	0.601262553	0.12568712	4.783804034	1.72E-06	3.69E-05
RBMS3	16.04514551	-0.620434945	0.228324439	-2.717339189	0.006580913	0.02459092
RBP4	43.47500715	-0.741819884	0.300910949	-2.46524723	0.013691874	0.04320742
RCAN1	6613.696826	0.790141536	0.208146168	3.796089762	0.000146996	0.00124643
RCAN3	40.40138768	0.743528075	0.178086592	4.175092954	2.98E-05	0.00035277
RCN3	437.3824591	0.684056187	0.227741735	3.003648786	0.002667631	0.01204065
RCOR2	484.9766914	-1.19325078	0.232216335	-5.138530752	2.77E-07	8.51E-06
RDH10	1584.988853	0.858803862	0.207541409	4.137988003	3.50E-05	0.00040065
RDH5	205.6989412	0.690728587	0.2061772	3.350169594	0.000807621	0.00473561
REC8	547.0587355	-0.636104324	0.232069556	-2.741007197	0.006125117	0.02325348
RELN	87.87274603	-2.303892856	0.344401969	-6.689546116	2.24E-11	3.85E-09
REM1	22.61418981	-1.386467571	0.313657741	-4.420319951	9.86E-06	0.00014772
REPS2	228.1884904	-0.984449204	0.239514127	-4.110192653	3.95E-05	0.00044376
RFPL1S	93.94332647	-1.186484955	0.280246268	-4.233722582	2.30E-05	0.00028915
RFPL3S	18.09081412	-0.729464559	0.203393045	-3.586477391	0.000335175	0.00240119
RGR	48.62582695	-1.226777839	0.299524961	-4.095744926	4.21E-05	0.00046779
RGS1	2751.029869	1.204341991	0.224680053	5.360253285	8.31E-08	3.27E-06
RGS18	108.669979	0.804886911	0.212957189	3.779571449	0.000157099	0.00131668
RGS2	1690.245339	1.267758773	0.221788557	5.71606935	1.09E-08	6.36E-07
RGS4	441.2126169	-0.797766903	0.309975804	-2.573642496	0.01006342	0.03412833
RGS7	88.29338729	-1.363944376	0.265343108	-5.14030451	2.74E-07	8.46E-06
RGS7BP	63.94623988	-0.770130519	0.234438151	-3.285005089	0.001019805	0.00569353
RHBDF2	855.157888	0.583875275	0.165743235	3.522769872	0.000427062	0.00290233
RHBDL3	465.7572243	-0.942085038	0.183508715	-5.133734604	2.84E-07	8.67E-06
RHOD	61.90176609	0.933286316	0.329611408	2.831474555	0.004633392	0.01860658
RHOH	62.22344029	0.86547024	0.18601165	4.652774377	3.27E-06	6.18E-05
RHOV	6.974251797	-0.676489597	0.243599121	-2.777060913	0.00548529	0.02129437
RIC3	348.6287919	-0.60544302	0.149197177	-4.058005867	4.95E-05	0.00053006
RIMBP2	101.4842982	-1.048721758	0.286131805	-3.665170178	0.000247174	0.00189189
RIMS1	85.61448744	-1.83689236	0.269620157	-6.812889593	9.57E-12	1.93E-09
RIMS3	607.8970388	-1.454454945	0.216691804	-6.712090247	1.92E-11	3.43E-09
RIMS4	790.5847203	-0.911542827	0.248674513	-3.665606154	0.000246753	0.00189016
RIN1	372.2768993	0.648064876	0.200219678	3.236769139	0.001208912	0.00647356
RLTPR	36.01441632	-1.038905514	0.279633156	-3.715244392	0.000203007	0.00162479
RMRP	104.7015349	-0.879051492	0.309807887	-2.837408372	0.00454814	0.01831232
RNASE1	3138.084006	0.765904954	0.243449018	3.14605892	0.001654867	0.00831785
RNASE2	172.6322439	0.932931535	0.24792692	3.762929554	0.000167935	0.0013914
RNASE3	25.74994051	0.680818037	0.26626618	2.556907663	0.010560725	0.03535312
RNASE4	426.2051602	0.590654412	0.158699079	3.721851541	0.000197767	0.00159349
RNASE6	517.5220665	0.78909416	0.165253148	4.775062807	1.80E-06	3.83E-05
RNF112	529.1032762	-0.89157364	0.156325436	-5.703317784	1.17E-08	6.77E-07
RNF144A	951.2659	-0.80321124	0.173993972	-4.616316488	3.91E-06	7.06E-05
RNF149	597.0986899	0.58293155	0.154027255	3.784599993	0.000153956	0.00129417
RNF150	65.30320574	-0.694811391	0.188954812	-3.677129914	0.000235873	0.00182569
RNF165	160.3400904	-1.444104539	0.260642463	-5.540557443	3.02E-08	1.42E-06
RNF43	20.2068006	-1.097830666	0.293615713	-3.739005162	0.00018475	0.00150268
RNFT2	347.3503792	-0.958166605	0.135619156	-7.065127341	1.60E-12	4.51E-10
RPL22L1	578.2252762	0.993957323	0.196754098	5.051774447	4.38E-07	1.23E-05
RPLP0P2	18.96045798	0.901590019	0.205385865	4.389737437	1.13E-05	0.00016457
RPPH1	157.50431	-1.153707808	0.302772424	-3.810478488	0.000138698	0.00119447
RPRM	202.1238053	-1.880394827	0.314085653	-5.986885461	2.14E-09	1.63E-07
RPS6KL1	249.5046531	-0.787994508	0.178942015	-4.403630471	1.06E-05	0.00015669
RRAS	635.4301213	0.750403252	0.156227219	4.803281108	1.56E-06	3.41E-05
RSPH10B2	21.62959668	-0.856449693	0.287767084	-2.976190614	0.002918534	0.01291475

RTKN2	151.7875859	-0.784805748	0.209612658	-3.744076124	0.000181059	0.00147614
RTN4R	124.2514303	-0.71839636	0.20090247	-3.57584632	0.000349097	0.00247983
RTN4RL1	40.17202779	-0.788524094	0.285560586	-2.761319775	0.005756828	0.02215477
RTN4RL2	94.47652077	-0.922337965	0.229742913	-4.014652517	5.95E-05	0.00061051
RTP4	135.2062731	0.586940293	0.186547584	3.146330172	0.001653332	0.00831318
RUNDC3A	811.4759523	-1.162077644	0.218527284	-5.317769117	1.05E-07	3.93E-06
RUNX1	916.1950214	0.846207739	0.184726796	4.580860808	4.63E-06	8.06E-05
RUNX1T1	34.35411798	-0.608006421	0.242673434	-2.505451092	0.012229531	0.03961585
RUNX2	136.5170241	0.702734948	0.195395198	3.596480139	0.000322552	0.0023363
RYR1	258.3783426	-0.82798476	0.199412835	-4.15211367	3.29E-05	0.00038257
RYR3	369.4627729	-0.789682237	0.311123897	-2.53816002	0.011143702	0.03691854
S100A11	3341.841344	0.672445126	0.169056727	3.977630107	6.96E-05	0.00068703
S100A13	1878.739713	0.691183386	0.200409536	3.44885477	0.000562969	0.00360185
S100A4	717.2826855	1.122614232	0.215370799	5.212471878	1.86E-07	6.28E-06
S100A6	11160.50675	0.762172728	0.18491961	4.1216436	3.76E-05	0.00042434
S100A8	451.4700367	0.820695535	0.300874887	2.72769703	0.006377816	0.02400752
S100A9	977.9973217	0.76382812	0.28115688	2.716732806	0.006592981	0.02462883
S100Z	8.39000967	0.588810627	0.217485486	2.707355962	0.006782149	0.02519869
S1PR4	23.2480717	0.647728776	0.195773967	3.308554179	0.00093779	0.00531106
S1PR5	85.34817092	-0.615654141	0.236084263	-2.607772892	0.00911334	0.03153578
SALL3	407.2170703	-1.056428853	0.21378876	-4.941461149	7.75E-07	1.95E-05
SAMD12	69.94491495	-0.80253743	0.19419517	-4.132633311	3.59E-05	0.00040865
SAMSN1	423.2929118	0.66234747	0.180802749	3.663370578	0.000248918	0.00190206
SARM1	875.6503729	-0.6189314	0.109305439	-5.662402565	1.49E-08	8.18E-07
SAT1	7426.073868	1.011652588	0.143188004	7.065204909	1.60E-12	4.51E-10
SATB1	736.5612624	-1.17366125	0.150391876	-7.80402025	6.00E-15	4.58E-12
SBK1	775.2525335	-1.349305777	0.205774825	-6.557195606	5.48E-11	8.38E-09
SCAMP5	2216.761189	-0.714699892	0.136944413	-5.21890508	1.80E-07	6.12E-06
SCARNA17	11.45272151	-0.854542498	0.240018051	-3.560325952	0.000370395	0.00260602
SCG2	3363.091815	1.016040709	0.216856119	4.685321834	2.80E-06	5.47E-05
SCN1A	350.5846174	-0.833051714	0.204943051	-4.064796103	4.81E-05	0.00051701
SCN2B	193.4542689	-0.906404255	0.231382726	-3.917337615	8.95E-05	0.00084336
SCN3A	695.800471	-0.769285624	0.226657262	-3.394047984	0.000688676	0.00419361
SCN3B	473.2730163	-1.542711456	0.257639293	-5.987873352	2.13E-09	1.63E-07
SCRT1	113.324461	-1.793392096	0.328836621	-5.453748103	4.93E-08	2.14E-06
SCUBE2	557.881017	-0.714777159	0.234340881	-3.050159906	0.002287196	0.01070625
SCUBE3	28.93715725	-1.557930479	0.298606774	-5.217331331	1.82E-07	6.15E-06
SDC1	577.749063	1.275922113	0.226549951	5.631968165	1.78E-08	9.50E-07
SDC2	2008.360791	0.842371662	0.176715615	4.766820764	1.87E-06	3.94E-05
SDC4	1947.750037	0.704563544	0.206220779	3.416549723	0.000634201	0.00394071
SDK2	150.0852135	-0.952659528	0.213169874	-4.469015765	7.86E-06	0.00012275
SEC14L5	122.5733577	-1.308103253	0.303471658	-4.310462671	1.63E-05	0.00022089
SEC31B	187.70107	-0.592378679	0.208559982	-2.840327622	0.004506722	0.01817214
SEC61A2	401.79231	-0.737690568	0.125606388	-5.873033836	4.28E-09	2.94E-07
SEC61G	14032.45401	1.098955258	0.358919139	3.061846354	0.002199763	0.01039844
SECTM1	282.4540746	0.693117538	0.212259898	3.265419164	0.001093022	0.00596678
SEL1L3	890.5746277	0.587091611	0.202800423	2.894923004	0.003792515	0.01584475
SELM	534.8155425	0.755312424	0.213071614	3.544875865	0.000392798	0.00272393
SEMA3A	280.215731	0.970912992	0.31219553	3.109951618	0.00187118	0.00916412
SEMA4G	140.7215051	-0.700135466	0.135142207	-5.180731345	2.21E-07	7.07E-06
SEMA5B	871.9610488	-0.898986747	0.200906923	-4.474642945	7.65E-06	0.00012079
SEMA6C	354.5663092	-0.816665501	0.166383232	-4.90834017	9.19E-07	2.24E-05
15-Sep	4483.857251	0.604035342	0.129457832	4.665884896	3.07E-06	5.91E-05
3-Sep	2870.149321	-1.027219996	0.183672673	-5.592666452	2.24E-08	1.13E-06
SERINC2	329.0990849	0.612469673	0.226738485	2.701216218	0.006908641	0.02555139
SERINC5	213.4328686	-0.906805728	0.215676244	-4.204476638	2.62E-05	0.00031946
SERPINA1	1813.250404	1.11455	0.209764135	5.313348728	1.08E-07	4.00E-06
SERPINB1	639.4035621	0.759011465	0.158243333	4.796483045	1.61E-06	3.51E-05
SERPINE1	5240.491806	1.453340245	0.262103199	5.544916091	2.94E-08	1.40E-06
SERPING1	5159.951065	0.726410723	0.196882335	3.689567791	0.000224635	0.0017555
SERTAD1	635.3641587	0.674856175	0.149759036	4.506280176	6.60E-06	0.00010632
SERTAD4	23.67656676	-0.822705371	0.303317751	-2.712354842	0.006680703	0.02490245
SFRP4	976.1172003	1.002790533	0.235129494	4.264843656	2.00E-05	0.00025972
SFTA1P	11.55153397	0.583072522	0.237997215	2.44991321	0.014289065	0.04462131
SGCD	117.3469291	-1.185393861	0.311935317	-3.800127128	0.000144622	0.00123467
SGK2	42.73313177	-0.777796864	0.240491934	-3.234191061	0.001219878	0.00651707
SGK494	86.04032815	-0.840432319	0.166225128	-5.055988407	4.28E-07	1.21E-05
SGMS2	106.1454036	0.903906404	0.193830171	4.663393729	3.11E-06	5.97E-05
SGSM1	101.3415788	-0.83956573	0.242885285	-3.456634804	0.000546966	0.00352239
SH2D5	59.23510961	-0.937568726	0.245470094	-3.819482494	0.000133732	0.00116335
SH3D19	591.2157813	-0.613714071	0.163881428	-3.744866496	0.00018049	0.00147325
SH3GL2	489.949424	-1.885622061	0.298625163	-6.314344186	2.71E-10	2.89E-08
SH3GL3	104.6135415	-1.394994382	0.31462167	-4.433878887	9.26E-06	0.00013981
SH3TC2	97.9194243	-0.710265983	0.210996023	-3.366252934	0.000761968	0.00453752

SHANK1	155.2382248	-1.587153838	0.300043915	-5.289738457	1.22E-07	4.48E-06
SHANK2	131.3331228	-1.883015017	0.278920769	-6.751074961	1.47E-11	2.77E-09
SHANK3	591.4945386	-0.664917183	0.15136213	-4.392889977	1.12E-05	0.00016259
SHC1	2551.452449	0.685034535	0.174303305	3.93012935	8.49E-05	0.00080597
SHD	628.5889089	-2.088506914	0.271687658	-7.687161535	1.50E-14	9.00E-12
SHISA7	302.0330611	-1.374590673	0.325874676	-4.218157394	2.46E-05	0.00030483
SHROOM3	459.3776596	0.63018792	0.192570888	3.272498391	0.001066015	0.00587817
SIDT1	142.7240956	-0.661765937	0.241218086	-2.743434163	0.006080024	0.02316211
SIGLEC10	909.2027957	0.641251873	0.203796437	3.146531328	0.001652196	0.0083105
SIGLEC14	146.0869836	0.668441884	0.226361649	2.952982044	0.003147203	0.01372364
SIGLEC1	435.3571526	1.10063919	0.271312692	4.056718405	4.98E-05	0.00053258
SIGLEC7	121.4491908	0.661018242	0.204192848	3.237225248	0.001206981	0.00646573
SIGLEC9	187.4283242	0.768494132	0.182657358	4.207299073	2.58E-05	0.00031643
SIM2	224.0714553	-1.392175027	0.217258571	-6.407917638	1.48E-10	1.78E-08
SIRPB2	150.5965816	0.700995991	0.202282994	3.465422273	0.0005294	0.00344161
SIRT2	4661.30137	-0.631361315	0.153831751	-4.104232779	4.06E-05	0.00045314
SIT1	11.74564319	0.797782932	0.263539575	3.027184554	0.002468432	0.0113538
SIX1	184.5480771	-0.915444947	0.270285636	-3.386953747	0.000706733	0.00427707
SIX4	82.69892716	-0.705226817	0.221999489	-3.176704686	0.001489586	0.00768066
SLA2	13.22654152	0.628105123	0.197568692	3.179173359	0.001476957	0.00762697
SLA	1075.905481	0.699780905	0.194688646	3.594359087	0.000325191	0.00234805
SLAMF6	23.29278548	0.877102223	0.248729957	3.526323226	0.000421372	0.00286934
SLAMF7	32.56827945	0.726001588	0.245107581	2.961971163	0.003056764	0.01340766
SLAMF8	300.1259549	0.883671517	0.213291409	4.143024418	3.43E-05	0.00039473
SLC11A1	1172.534142	0.80500104	0.202525792	3.974807507	7.04E-05	0.00069338
SLC12A5	203.8251289	-1.605262658	0.334270192	-4.802290777	1.57E-06	3.42E-05
SLC16A10	107.1654398	0.77884014	0.249280809	3.124348574	0.001781992	0.00880566
SLC16A3	1315.41449	0.664520464	0.181919378	3.652829457	0.000259367	0.00196661
SLC17A7	1083.649442	-2.004294305	0.396933559	-5.049445322	4.43E-07	1.24E-05
SLC17A9	63.94602539	0.769860048	0.178297689	4.317835264	1.58E-05	0.00021534
SLC1A7	19.12653458	-1.020216229	0.272075284	-3.749757099	0.000177006	0.00145084
SLC20A1	1636.638432	0.792877457	0.161894106	4.89750661	9.71E-07	2.33E-05
SLC25A27	172.0040538	-1.243939501	0.206601171	-6.020970244	1.73E-09	1.38E-07
SLC26A10	167.5968748	-1.669702677	0.245130864	-6.81147468	9.66E-12	1.93E-09
SLC26A1	32.02207983	-0.609614079	0.173357127	-3.516521591	0.000437241	0.00296128
SLC26A4	27.77121096	0.765704013	0.2156105	3.551329892	0.00038329	0.0026803
SLC2A5	1390.542197	0.600733448	0.195635835	3.070671839	0.002135777	0.01019095
SLC30A10	52.05196959	-0.722120335	0.197399444	-3.658168038	0.000254024	0.00193387
SLC30A3	57.47517205	-1.397785744	0.316277995	-4.419484648	9.89E-06	0.000148
SLC37A2	350.9645751	0.62873318	0.18757179	3.351960225	0.000802415	0.00471312
SLC38A1	1419.051063	-0.582834731	0.210433871	-2.769681171	0.005611119	0.02170937
SLC39A8	869.0413161	0.843433494	0.161366143	5.226830597	1.72E-07	5.92E-06
SLC44A1	3366.094182	-0.712175134	0.171747069	-4.146650869	3.37E-05	0.00038918
SLC44A5	226.7521545	-0.911931266	0.337108414	-2.705157236	0.006827207	0.02533873
SLC45A3	131.5496754	-0.605251841	0.225448538	-2.684656316	0.007260442	0.02649647
SLC4A4	4181.204072	0.626231168	0.214154261	2.924205968	0.003453362	0.01471823
SLC4A7	631.1085834	0.604985442	0.127247838	4.754386809	1.99E-06	4.13E-05
SLC6A13	19.11169662	-0.621764458	0.252172631	-2.465630214	0.013677245	0.0431919
SLC6A15	94.19006978	-1.15344979	0.363828769	-3.170309467	0.001522767	0.00780496
SLC6A16	18.74130642	-0.693977035	0.245767656	-2.823711813	0.004747105	0.01898014
SLC6A17	252.3057981	-1.251728151	0.33058075	-3.786452029	0.000152814	0.00128625
SLC7A14	189.0548688	-0.960089877	0.285625161	-3.361363104	0.000775588	0.00459674
SLC7A3	90.06856941	-1.879968397	0.351081858	-5.354786516	8.57E-08	3.33E-06
SLC8A2	148.9225833	-2.066652543	0.320779563	-6.442594167	1.17E-10	1.50E-08
SLC8A3	245.5357584	-1.695388373	0.296131245	-5.725124935	1.03E-08	6.08E-07
SLC9A5	59.30105001	-0.801944709	0.193309635	-4.148498378	3.35E-05	0.00038703
SLCO1A2	299.0713753	-0.954979688	0.273343216	-3.493701804	0.000476373	0.00315792
SLITRK1	203.9518099	-2.414540385	0.327073857	-7.382248175	1.56E-13	6.49E-11
SLITRK2	873.1987419	-1.513318113	0.220231742	-6.87148047	6.35E-12	1.41E-09
SLITRK5	140.0532784	-0.850176906	0.231550442	-3.67167041	0.00024097	0.00185888
SLPI	517.5250917	1.752537537	0.35504558	4.936091692	7.97E-07	1.98E-05
SMAGP	86.1435986	1.147605884	0.187551332	6.118889528	9.42E-10	8.16E-08
SMOC1	2531.987669	-1.824340498	0.363970539	-5.012330133	5.38E-07	1.48E-05
SMPD3	160.4856923	-1.464085103	0.231639562	-6.320531316	2.61E-10	2.80E-08
SMPDL3A	249.6303821	0.696804076	0.143571415	4.853362197	1.21E-06	2.78E-05
SMYD2	852.109074	1.172198367	0.15221338	7.701020561	1.35E-14	8.44E-12
SNAI2	293.8630915	0.901499798	0.242551635	3.716733539	0.000201815	0.00161946
SNAP25	2267.06913	-1.355242275	0.302998457	-4.472769552	7.72E-06	0.00012144
SNAP91	359.5817027	-2.157300243	0.325015647	-6.637527348	3.19E-11	5.35E-09
SNCA	421.708723	-0.598085486	0.22858619	-2.616455029	0.008884807	0.0309865
SNCB	435.5055887	-1.550455272	0.319684886	-4.849948617	1.23E-06	2.83E-05
SNCG	143.7494962	-1.30495659	0.26460096	-4.931790842	8.15E-07	2.02E-05
SNED1	337.7513438	0.686891351	0.210806339	3.258399888	0.001120424	0.00608736
SNPH	594.5377097	-0.707106947	0.145246103	-4.868336795	1.13E-06	2.63E-05

SNRPE	792.0888098	-0.596495929	0.183476511	-3.251075166	0.001149695	0.00622182
SNX10	1443.565171	0.628500778	0.234688143	2.678025275	0.007405763	0.02691973
SNX22	802.8861184	-1.166013482	0.214685272	-5.431269095	5.60E-08	2.33E-06
SNX32	77.06471381	-0.875918751	0.16057743	-5.454806134	4.90E-08	2.14E-06
SOCS1	101.1846449	0.959356942	0.207157461	4.631051847	3.64E-06	6.73E-05
SOCS2	1429.237251	0.990711283	0.259457467	3.81839573	0.000134322	0.00116554
SOCS3	2094.936833	0.661003527	0.234307322	2.821096331	0.004785983	0.01909701
SOD2	19631.51414	0.6770075	0.224909238	3.010136477	0.002611303	0.01183165
SORCS3	208.7047078	-0.740813634	0.260298256	-2.846018434	0.004426963	0.01788722
SOSTDC1	15.5217823	-1.139640516	0.299758913	-3.801856978	0.000143616	0.00122989
SOX10	1560.618581	-2.624045416	0.372615163	-7.042240024	1.89E-12	5.06E-10
SOX11	1446.484855	-1.382840509	0.223987403	-6.173742306	6.67E-10	6.16E-08
SOX13	1066.175439	-0.718434614	0.158110192	-4.543885524	5.52E-06	9.20E-05
SOX1	139.4378723	-0.945851689	0.350518238	-2.698437875	0.006966574	0.02573798
SOX3	81.83258816	-1.347084259	0.304037441	-4.430652541	9.39E-06	0.00014154
SOX4	3362.538421	-1.480791509	0.201322035	-7.355337488	1.90E-13	7.49E-11
SOX5	173.995297	-0.62732991	0.182395147	-3.439400223	0.000583005	0.00369057
SOX6	1037.851548	-0.896777937	0.18793414	-4.771767056	1.83E-06	3.88E-05
SOX8	4053.911163	-1.331307192	0.225288128	-5.909353513	3.43E-09	2.46E-07
SP100	960.7163669	0.722438547	0.148734102	4.857248849	1.19E-06	2.74E-05
SP6	65.40722213	1.688747046	0.214840493	7.860469066	3.83E-15	3.51E-12
SPAG4	84.80744168	0.780217457	0.237665802	3.28283434	0.00102769	0.00572131
SPESP1	23.19044211	-0.942598144	0.259939736	-3.626217985	0.000287603	0.0021383
SPHK1	286.5039332	0.814741186	0.20396386	3.994537009	6.48E-05	0.00065209
SPHKAP	54.35671494	-1.795485085	0.33905126	-5.295615428	1.19E-07	4.35E-06
SPINT1	84.80977786	1.236614584	0.206305279	5.994100546	2.05E-09	1.57E-07
SPOCD1	2207.11925	1.031799544	0.282437295	3.653198647	0.000258994	0.00196487
SPOCK3	391.0542727	-0.849496323	0.323204019	-2.628359404	0.008579781	0.03013694
SPRN	155.8094183	-0.615338341	0.165416019	-3.719944087	0.000199267	0.00160369
SPRY1	964.1716662	0.580141007	0.189912499	3.054780544	0.002252253	0.01057145
SPTB	115.3439216	-0.809206658	0.262262878	-3.085479209	0.002032244	0.0097892
SPTBN2	628.1872272	-1.408057609	0.204399469	-6.888753769	5.63E-12	1.29E-09
SQRDL	570.2670999	0.691991618	0.148971577	4.645125132	3.40E-06	6.38E-05
SRC	914.6215351	-0.642964058	0.122531496	-5.247337053	1.54E-07	5.38E-06
SRCIN1	474.547889	-1.391230573	0.225913609	-6.158241549	7.36E-10	6.75E-08
SRGAP3	858.3787748	-0.610756662	0.13373285	-4.566990558	4.95E-06	8.51E-05
SRGN	3083.506755	0.858041263	0.188439448	4.553405743	5.28E-06	8.88E-05
SRL	20.39668166	-1.568858725	0.261146499	-6.00758093	1.88E-09	1.48E-07
SRPK3	108.2563708	-1.164705718	0.201929597	-5.767880174	8.03E-09	5.00E-07
SRPX2	762.8238252	1.598954742	0.235067319	6.802114161	1.03E-11	2.03E-09
SRPX	2701.006033	2.11586616	0.215744585	9.807273523	1.05E-22	4.81E-19
SRRM3	204.6681983	-1.154533793	0.251077965	-4.598307917	4.26E-06	7.54E-05
SSPO	48.52687506	-0.812260634	0.280302638	-2.897798754	0.003757916	0.01574321
SSR3	3661.161251	0.663353344	0.105192935	6.306063661	2.86E-10	2.94E-08
SSTR2	113.298251	-1.322458759	0.233935175	-5.653099245	1.58E-08	8.55E-07
ST18	134.7132903	-1.102997655	0.30711622	-3.591466624	0.000328822	0.00237038
ST6GALNAC5	103.2353966	1.055354678	0.312819006	3.373691042	0.000741676	0.00444241
ST8SIA3	258.5508715	-1.728793216	0.359321328	-4.811273595	1.50E-06	3.31E-05
ST8SIA4	580.0299742	0.734249644	0.167527345	4.382864443	1.17E-05	0.00016897
STAB1	2838.859412	0.640167186	0.18897908	3.387502918	0.00070532	0.0042704
STAC	265.9261901	0.951590782	0.323282777	2.943524521	0.003244981	0.0140609
STEAP1	134.8701706	0.726396058	0.281948025	2.576347388	0.009985027	0.03391262
STEAP3	2021.340843	0.611776676	0.187008346	3.27138702	0.001070213	0.00589131
STEAP4	43.49067187	1.054893008	0.310210824	3.40056802	0.00067246	0.00412039
STK32C	298.901877	-0.647430769	0.136971974	-4.726738967	2.28E-06	4.60E-05
STMN2	1318.154649	-2.081353958	0.35625042	-5.842390189	5.15E-09	3.42E-07
STMN4	920.9327382	-1.212940521	0.244200007	-4.966996256	6.80E-07	1.77E-05
STOX2	71.22610096	-0.927603674	0.181362919	-5.114626949	3.14E-07	9.45E-06
STRC	17.41169536	-1.084087992	0.214014661	-5.065484712	4.07E-07	1.17E-05
STX11	86.82321148	0.745831098	0.144546048	5.1598166	2.47E-07	7.75E-06
STX1A	370.2572441	-0.58877766	0.192960279	-3.05128943	0.002278608	0.01068059
STX1B	564.0877105	-1.22745916	0.20201274	-6.076147276	1.23E-09	1.01E-07
STXBP1	1769.59406	-0.797450184	0.164271544	-4.854463329	1.21E-06	2.78E-05
STXBP5L	121.4300087	-1.053717977	0.280479435	-3.756845764	0.000172068	0.00141875
SULT1C4	315.0748704	-0.754718457	0.173452157	-4.351162124	1.35E-05	0.00019094
SULT4A1	218.848374	-1.490729671	0.35463774	-4.203528001	2.63E-05	0.00032004
SUMF2	6674.648009	0.857165562	0.214675545	3.99284213	6.53E-05	0.00065581
SUSD2	248.9523745	0.660106804	0.193327293	3.41445221	0.000639104	0.00396429
SUSD4	541.5968388	-1.072560468	0.196965994	-5.445409363	5.17E-08	2.19E-06
SUSD5	197.8831761	-2.355518255	0.328666673	-7.166891101	7.67E-13	2.65E-10
SV2B	209.2180612	-1.450011128	0.370686387	-3.911692413	9.17E-05	0.0008592
SVOP	93.45513384	-1.796308169	0.383515879	-4.683790859	2.82E-06	5.49E-05
SYCE2	29.08905786	-0.662588725	0.176618139	-3.751532717	0.000175757	0.00144318
SYN1	817.5630682	-1.636607511	0.267659641	-6.114509854	9.69E-10	8.28E-08

SYN2	517.4046487	-2.325285154	0.368724131	-6.306300451	2.86E-10	2.94E-08
SYN3	41.46081167	-0.952684056	0.244808213	-3.89155267	9.96E-05	0.00091384
SYNGR3	220.7624669	-1.058092324	0.277056926	-3.819043038	0.00013397	0.00116395
SYNPO2L	10.88253425	-1.228616648	0.249171619	-4.930804937	8.19E-07	2.02E-05
SYP	1325.480001	-0.869699533	0.205576032	-4.230549266	2.33E-05	0.0002924
SYT13	219.918873	-2.124257296	0.336061108	-6.321044728	2.60E-10	2.80E-08
SYT15	27.14460329	-0.961747612	0.206426786	-4.659025258	3.18E-06	6.03E-05
SYT1	982.4910778	-1.664192055	0.327680964	-5.07869616	3.80E-07	1.11E-05
SYT2	38.70239448	-0.774014641	0.291866048	-2.651951625	0.008002801	0.02856186
SYT4	204.3666154	-1.213953718	0.353811001	-3.431079629	0.000601184	0.00377613
SYT5	186.3339796	-1.04644484	0.275846212	-3.793580607	0.00014849	0.00125524
SYT6	265.0137506	-1.098267507	0.253735394	-4.328396957	1.50E-05	0.00020629
SYT7	426.612483	-1.259605789	0.247132015	-5.096894426	3.45E-07	1.02E-05
SYT9	70.86324343	-1.010549883	0.293254997	-3.44597669	0.000568999	0.00363368
SYTL1	15.48181578	0.83815281	0.236084909	3.550217649	0.000384913	0.00268755
SYTL5	80.98158447	0.975479841	0.274436169	3.55448717	0.000378717	0.00265372
TACSTD2	16.18291436	0.913386116	0.26198467	3.486410551	0.000489549	0.00323591
TAGLN3	913.5582634	-1.156715612	0.258281939	-4.478499804	7.52E-06	0.00011891
TAGLN	4357.673226	0.685479941	0.220012528	3.115640488	0.001835459	0.0090245
TAP1	3147.727026	0.83118915	0.165655757	5.017568752	5.23E-07	1.44E-05
TBC1D10C	37.71296959	0.727427057	0.187351195	3.882692377	0.000103306	0.00094402
TBC1D3H	12.52181414	-0.760910246	0.280603378	-2.711693109	0.006694054	0.02493195
TBC1D8B	60.35648864	0.673725105	0.230975003	2.916874532	0.00353558	0.01498511
TBX1	38.31428911	1.400106422	0.35093697	3.989623615	6.62E-05	0.00066236
TBX3	133.878596	-0.747509824	0.228795823	-3.267148041	0.001086369	0.00594221
TCAP	28.16769506	-0.699153192	0.157335244	-4.443716331	8.84E-06	0.0001349
TCEAL2	746.5734027	-0.857245198	0.217341284	-3.944235453	8.01E-05	0.00076991
TCEAL5	303.0256842	-0.693655321	0.18214252	-3.808310762	0.000139919	0.00120273
TCEAL6	106.3187316	-0.997452445	0.260882878	-3.823372593	0.000131639	0.00114856
TCF7L1	596.125617	-0.788225418	0.146067797	-5.396298405	6.80E-08	2.75E-06
TCIRG1	678.4320117	0.657872676	0.153017144	4.299339654	1.71E-05	0.00023045
TCN2	1161.10837	0.672001326	0.120703384	5.567377679	2.59E-08	1.28E-06
TDO2	93.25171498	0.969809632	0.305621991	3.17323249	0.001507518	0.00773833
TDRD9	18.03380684	-0.678392459	0.276950635	-2.44950678	0.014305202	0.04466157
TEC	9.575831502	1.061828793	0.226702751	4.68379316	2.82E-06	5.49E-05
TECTA	51.15266141	-0.618898989	0.166043612	-3.727327902	0.000193521	0.00156368
TET1	52.30401469	-0.822281143	0.198673879	-4.138848791	3.49E-05	0.00039964
TEX14	12.81397657	-0.841458122	0.253307123	-3.321888907	0.000894103	0.00513121
TFAP2A	173.4813081	-1.79031849	0.308169972	-5.809516354	6.27E-09	4.05E-07
TFCP2L1	248.9422731	0.897424446	0.297040182	3.021222384	0.002517564	0.01152969
TFEC	186.0855588	0.702491582	0.176337042	3.983800409	6.78E-05	0.00067635
TG	20.23549485	-0.708694785	0.276608908	-2.562082296	0.010404666	0.03494972
TGFBI	5546.8256	1.329284521	0.226496337	5.868900734	4.39E-09	2.99E-07
TGFBR2	1447.306023	0.606320843	0.152845061	3.966898495	7.28E-05	0.00071169
TGM2	1396.82571	0.625454899	0.183326433	3.411700583	0.00064559	0.00399308
THBD	338.0105713	0.931019211	0.207851766	4.479246093	7.49E-06	0.0001189
THBS1	1991.433604	1.633053427	0.296498443	5.507797643	3.63E-08	1.68E-06
THBS4	1556.82064	0.813931948	0.27672619	2.941289901	0.003268485	0.0141316
THPO	18.70729504	-0.60893144	0.209556077	-2.905816187	0.003662966	0.01541111
THRA	4539.061684	-0.930012833	0.144343347	-6.443059898	1.17E-10	1.50E-08
THSD4	161.676662	-0.77928886	0.248593032	-3.134797682	0.001719727	0.00857806
TICAM2	149.6651276	0.593778645	0.134564227	4.412603975	1.02E-05	0.00015195
TIFA	196.9937544	0.581785783	0.175524261	3.314560505	0.000917873	0.00523055
TIGD3	44.40775148	-0.648580916	0.155044026	-4.183204825	2.87E-05	0.00034277
TIMP1	10628.90563	1.357724299	0.222731287	6.095795161	1.09E-09	9.10E-08
TKTL1	85.48457479	-2.282019878	0.352516809	-6.473506564	9.58E-11	1.29E-08
TLL2	29.80786718	-0.709450383	0.276653599	-2.564399619	0.010335447	0.03480216
TLR2	619.6996989	0.958199749	0.170859642	5.608110467	2.05E-08	1.05E-06
TLR8	98.9109786	1.154221852	0.249927163	4.618232924	3.87E-06	7.03E-05
TMBIM4	1403.024138	0.584128887	0.136905977	4.26664271	1.98E-05	0.00025866
TMCC2	425.5144697	-0.998344181	0.170412566	-5.858395333	4.67E-09	3.17E-07
TMEFF1	491.4805061	-0.83512226	0.158861844	-5.256909019	1.46E-07	5.16E-06
TMEFF2	321.3313201	-1.724628744	0.331532751	-5.201986052	1.97E-07	6.54E-06
TMEM100	642.8114218	-0.92800393	0.231693191	-4.005313774	6.19E-05	0.00062765
TMEM130	353.308829	-1.360863087	0.323739346	-4.203576434	2.63E-05	0.00032004
TMEM132C	160.360158	-1.751878668	0.340741707	-5.141368474	2.73E-07	8.43E-06
TMEM145	347.2021313	-0.618575718	0.200913889	-3.078810129	0.002078291	0.0099587
TMEM151A	251.6485587	-1.176340595	0.27753279	-4.238564364	2.25E-05	0.00028428
TMEM151B	291.2300288	-1.274267871	0.241708122	-5.271928229	1.35E-07	4.85E-06
TMEM154	107.280934	0.921809684	0.162500396	5.672661161	1.41E-08	7.83E-07
TMEM155	47.78218275	-0.910260142	0.292881619	-3.107945615	0.001883927	0.00919383
TMEM200B	39.81918596	0.981282565	0.258447466	3.796835696	0.000146555	0.00124499
TMEM26	52.38385276	0.857068644	0.209186549	4.097149881	4.18E-05	0.00046609
TMEM35	356.2116105	-0.78940499	0.200325148	-3.940618535	8.13E-05	0.00077779

TMEM63C	185.8211713	-1.0579597	0.201141482	-5.259778799	1.44E-07	5.09E-06
TMEM71	136.6307145	0.707581548	0.219843693	3.21856651	0.001288331	0.00681011
TMEM74	37.16342179	-0.622258079	0.257599259	-2.415605078	0.015709089	0.04810603
TMEM98	951.768784	-1.045435441	0.191984601	-5.445412983	5.17E-08	2.19E-06
TMPRSS5	223.9066174	-0.897999903	0.183416753	-4.895953542	9.78E-07	2.34E-05
TMPRSS9	32.5888977	-2.16333092	0.363576519	-5.950139265	2.68E-09	1.95E-07
TMSB15A	280.4141953	-1.951584385	0.357241681	-5.462924651	4.68E-08	2.07E-06
TNC	13006.54227	0.746980369	0.171382533	4.358556003	1.31E-05	0.00018671
TNFAIP3	469.3742703	0.792147334	0.170329294	4.650681708	3.31E-06	6.24E-05
TNFAIP6	330.2284939	0.80094886	0.210797425	3.799614071	0.000144922	0.00123646
TNFAIP8	142.3201549	0.716497314	0.169576114	4.225225461	2.39E-05	0.00029783
TNFAIP8L3	297.8605268	0.647420433	0.190533146	3.397941235	0.00067895	0.00414907
TNFRSF10A	17.2761252	1.066026663	0.210576188	5.062427396	4.14E-07	1.18E-05
TNFRSF10C	57.35588157	0.624417235	0.192699774	3.240363095	0.001193776	0.00640747
TNFRSF10D	155.5570948	0.680014409	0.194242954	3.50084466	0.000463786	0.00309386
TNFRSF11A	58.03386608	0.831529138	0.197960217	4.200486095	2.66E-05	0.00032228
TNFRSF11B	228.8610641	0.954007301	0.226110992	4.219199124	2.45E-05	0.0003037
TNFRSF13C	8.584575431	-1.094153102	0.261804326	-4.179278155	2.92E-05	0.00034724
TNFRSF14	611.1365414	0.687891143	0.126221724	5.449863308	5.04E-08	2.17E-06
TNFRSF18	17.49442854	1.249877773	0.269856825	4.631632991	3.63E-06	6.73E-05
TNFRSF1B	1164.366356	0.726895082	0.178768209	4.066131695	4.78E-05	0.00051553
TNFRSF6B	134.9672488	0.811272667	0.192278429	4.219259909	2.45E-05	0.0003037
TNFSF10	359.2738479	0.876769681	0.184507082	4.751956794	2.01E-06	4.15E-05
TNFSF4	93.65163933	1.48825064	0.201706143	7.378310915	1.60E-13	6.49E-11
TNFSF8	66.96656264	0.59656214	0.198745066	3.00164503	0.002685251	0.01209242
TNFSF9	24.30763644	0.934534304	0.240973805	3.878157237	0.000105251	0.00095924
TNK1	12.01464634	-0.830165488	0.244877554	-3.390124876	0.000698608	0.00424283
TNK2	2124.592081	-0.867004828	0.15568793	-5.568863502	2.56E-08	1.27E-06
TNR	257.3376061	-2.22507397	0.336440789	-6.613567797	3.75E-11	6.07E-09
TNRC6C	568.8948143	-0.652712043	0.131123931	-4.977825448	6.43E-07	1.70E-05
TOP2A	2496.885688	-0.62375094	0.190428098	-3.275519461	0.001054678	0.0058338
TOX3	251.7850355	-1.794556673	0.280907584	-6.388423717	1.68E-10	1.97E-08
TP73	103.0370324	-0.949472581	0.289271529	-3.282288389	0.001029682	0.00573008
TPD52	1418.804741	0.682039466	0.150062182	4.54504564	5.49E-06	9.16E-05
TPM2	1562.066755	0.623327997	0.205042322	3.039996776	0.002365807	0.01098456
TPPP	1019.252748	-1.190554219	0.253352843	-4.699194225	2.61E-06	5.18E-05
TPRG1	17.72348959	0.593571558	0.174294457	3.405567623	0.000660267	0.00405832
TPST2	549.6577068	0.60601748	0.131944899	4.592958769	4.37E-06	7.71E-05
TPT1	41813.26564	0.597613278	0.145738553	4.100584674	4.12E-05	0.0004596
TPTE2P1	25.84809875	-1.325319424	0.241698901	-5.483348979	4.17E-08	1.88E-06
TRAM1L1	106.704804	-1.094650906	0.255411956	-4.285824846	1.82E-05	0.00024208
TREM1	277.8785123	1.004086552	0.302712841	3.316960551	0.000910025	0.00519227
TRIM22	1690.503658	0.818333572	0.17377781	4.709079793	2.49E-06	4.98E-05
TRIM38	240.0488139	0.638274883	0.152974926	4.172415035	3.01E-05	0.00035587
TRIM67	90.23804543	-2.585541993	0.341034164	-7.581475016	3.42E-14	1.81E-11
TRO	1240.770555	-0.707893779	0.149109294	-4.747482605	2.06E-06	4.21E-05
TRPM3	53.4593651	1.16238112	0.273879669	4.244130726	2.19E-05	0.00028026
TRPV1	181.2660074	-0.612901316	0.154462133	-3.967971328	7.25E-05	0.000709
TRPV4	34.6896436	0.608900699	0.221552621	2.748334446	0.005989888	0.02287343
TSFM	1618.329456	-1.344641479	0.255439618	-5.26402869	1.41E-07	5.02E-06
TSHR	91.36406554	-3.332735555	0.419611897	-7.942423892	1.98E-15	2.27E-12
TSLP	27.20857171	0.744279788	0.305817193	2.433740827	0.014943691	0.04612134
TSPAN12	978.602764	-0.649630577	0.195057772	-3.330452147	0.000867051	0.00500518
TSPAN18	516.4782945	-0.814845393	0.226704528	-3.594305774	0.000325258	0.00234805
TSPAN31	6721.155658	-2.198842966	0.319679912	-6.878264416	6.06E-12	1.37E-09
TSPYL4	1691.505152	-0.598054597	0.115133368	-5.194450653	2.05E-07	6.73E-06
TSTD1	254.5833944	0.750668066	0.259299642	2.894983045	0.00379179	0.01584475
TTBK1	468.3194993	-0.828004635	0.162237086	-5.103670548	3.33E-07	9.97E-06
TTC18	88.89960728	-0.836974478	0.28230971	-2.964738545	0.003029403	0.01330269
TTC39A	71.19861908	0.867615671	0.214015717	4.0539811	5.04E-05	0.00053718
TTC39B	49.35266827	1.010472534	0.16856277	5.99463649	2.04E-09	1.57E-07
TTC9B	118.70824	-1.077447979	0.324708093	-3.31820488	0.00090598	0.00517504
TTLL13	15.30919638	-0.646891145	0.161472897	-4.006190241	6.17E-05	0.00062625
TTYH2	1922.372729	-0.754909975	0.138120453	-5.465591523	4.61E-08	2.04E-06
TUB	858.0140355	-0.893310743	0.141552019	-6.310830095	2.78E-10	2.93E-08
TUBA1C	4472.526879	0.640409735	0.165387679	3.872173174	0.000107869	0.00097793
TUBA8	107.2112087	-0.9821091	0.210800129	-4.658958723	3.18E-06	6.03E-05
TWSG1	1693.830186	0.655832515	0.134946063	4.859960357	1.17E-06	2.72E-05
TYMP	794.3992381	0.774747379	0.184130524	4.207598835	2.58E-05	0.00031629
TYROBP	2197.271023	0.634156495	0.180603732	3.511314457	0.000445897	0.0030007
UBE2QL1	279.2001671	-1.368446591	0.249220115	-5.490915504	4.00E-08	1.83E-06
UCP3	37.53769567	-0.62930639	0.146305555	-4.301315756	1.70E-05	0.00022885
UGCG	276.5785052	0.618630378	0.14717916	4.2032471	2.63E-05	0.00032007
UGT8	549.083244	-1.566834151	0.313824903	-4.992701772	5.95E-07	1.60E-05

UNC13A	598.4651896	-0.833650575	0.251645315	-3.312799904	0.00092367	0.00525488
UNC5A	206.5933004	-1.404994653	0.27732932	-5.066159801	4.06E-07	1.17E-05
UNC5CL	36.19091847	-0.614854672	0.171163992	-3.592196378	0.000327903	0.00236509
UNC80	345.6529262	-0.754163261	0.188168819	-4.007907714	6.13E-05	0.00062217
UPK2	7.012842739	-0.79656924	0.183356647	-4.344370665	1.40E-05	0.00019475
UPP1	966.5733701	0.650591282	0.185276531	3.511460843	0.000445651	0.00300051
USP2	614.184818	-0.611671373	0.17236554	-3.548687129	0.000387157	0.00269638
USP43	60.41164895	-1.915746587	0.313576247	-6.109348539	1.00E-09	8.50E-08
USP49	22.07721831	-0.94851512	0.209596015	-4.525444437	6.03E-06	9.84E-05
USP51	41.94027184	-0.59208073	0.19204484	-3.083033794	0.002049019	0.0098562
UST	1045.626833	-0.733386217	0.165002073	-4.444709117	8.80E-06	0.00013443
VAMP5	1438.209034	0.710178452	0.18077573	3.928505509	8.55E-05	0.00081013
VAMP8	909.7466419	0.635112815	0.190266279	3.338020892	0.000843774	0.00489752
VAV1	290.7732582	0.597414851	0.182867467	3.266928015	0.001087213	0.00594447
VAX2	105.6867189	-0.858090254	0.206804206	-4.149288211	3.34E-05	0.00038605
VDR	77.06741805	1.001302281	0.271612165	3.686514855	0.000227346	0.00177165
VEGFC	38.51245316	0.906608117	0.229558409	3.949357039	7.84E-05	0.00075838
VENTX	42.13038493	0.710154211	0.237329706	2.992268534	0.002769126	0.01238822
VEPH1	85.78434963	-2.311477054	0.361400264	-6.395891986	1.60E-10	1.91E-08
VGF	1440.211706	-1.630037951	0.325437006	-5.008766435	5.48E-07	1.50E-05
VGLL3	31.4201787	1.063879514	0.256942567	4.14053431	3.46E-05	0.00039768
VIPR2	384.300044	-4.109030126	0.366826875	-11.20155149	4.01E-29	2.76E-25
VMO1	89.99756963	0.795445419	0.185671175	4.284162154	1.83E-05	0.00024259
VNN1	32.03155853	1.156417092	0.211843131	5.458836852	4.79E-08	2.10E-06
VNN2	80.9306299	0.795367686	0.237662347	3.346628924	0.000818006	0.00477616
VOPP1	5839.925113	0.716430803	0.288578481	2.482620323	0.013042001	0.04164362
VSIG4	4045.390039	0.938596822	0.230964245	4.063818716	4.83E-05	0.00051847
VSNL1	629.5596985	-1.379617679	0.366536061	-3.763934374	0.000167261	0.00138833
VSTM2A	141.1264374	-2.053114825	0.396151447	-5.18265133	2.19E-07	7.03E-06
VSTM2L	214.5242703	-1.01899918	0.273172149	-3.730245504	0.000191293	0.00154858
VTN	12.18626785	-0.650442142	0.229513216	-2.834007352	0.004596827	0.01847592
VWA5B2	46.40979099	-1.389381931	0.23604753	-5.886026127	3.96E-09	2.78E-07
VWCE	125.9310726	-0.937535646	0.204430504	-4.586084901	4.52E-06	7.92E-05
WASF1	905.8418519	-1.135975371	0.17443805	-6.512199431	7.41E-11	1.07E-08
WBSCR27	23.95543715	0.612560603	0.233688527	2.621269481	0.008760298	0.03068446
WFDC1	71.64146379	-0.842057763	0.231517661	-3.63712107	0.000275702	0.00206258
WFIKKN1	42.53248194	-0.72555572	0.215820297	-3.361851181	0.000774218	0.00459118
WFIKKN2	48.51912511	-1.782078506	0.338149086	-5.270097072	1.36E-07	4.89E-06
WISP1	275.4653692	1.225660235	0.331538538	3.696886168	0.00021826	0.00171935
WNK2	246.1855736	-1.371118642	0.306619765	-4.471722949	7.76E-06	0.00012174
WNT2B	31.96053365	0.619795532	0.187601506	3.30378761	0.000953881	0.00539109
WNT7B	119.851001	-1.187385043	0.311189389	-3.815634734	0.000135833	0.00117495
XAF1	1022.510927	0.730233464	0.231120536	3.159535175	0.00158021	0.00802171
XRCC2	77.98794943	-0.680614342	0.181378208	-3.7524593	0.000175108	0.00143871
XRCC6BP1	196.3522572	-1.19026354	0.263260438	-4.521239679	6.15E-06	0.00010001
YJEFN3	199.109692	-0.820122285	0.214555806	-3.822419449	0.000132149	0.0011503
YPEL4	111.0411132	-0.796682083	0.179444513	-4.439712698	9.01E-06	0.00013691
ZBTB12	206.7509839	-0.783325804	0.148659993	-5.269244199	1.37E-07	4.90E-06
ZBTB8B	39.82677734	-0.741927692	0.187930433	-3.947884762	7.88E-05	0.00076156
ZC3H12A	126.3531376	0.961411584	0.175813139	5.46837165	4.54E-08	2.02E-06
ZC3H12B	53.59425485	-1.805760667	0.286667019	-6.299157374	2.99E-10	3.03E-08
ZC3H12D	14.26355745	0.76049984	0.181947604	4.179773856	2.92E-05	0.00034678
ZC4H2	366.4226187	-0.831172064	0.146021272	-5.692130013	1.25E-08	7.16E-07
ZCCHC12	158.6829051	-1.207003869	0.253651578	-4.758511176	1.95E-06	4.07E-05
ZCCHC18	150.280182	-0.804759471	0.162879124	-4.940838651	7.78E-07	1.95E-05
ZDHHC11	59.43575173	-0.961623223	0.229829318	-4.184075526	2.86E-05	0.00034176
ZDHHC22	633.4755892	-1.065598811	0.250812529	-4.248586842	2.15E-05	0.00027617
ZDHHC23	257.1938006	0.841616881	0.221911174	3.792584517	0.000149087	0.00125874
ZFPM2	280.3496585	-1.113544409	0.256576974	-4.340001336	1.42E-05	0.00019786
ZFR2	52.78763232	-1.259319946	0.342033304	-3.681863528	0.000231535	0.0017992
ZIC3	30.46400678	-0.67333657	0.233131448	-2.888227113	0.0038742	0.01609483
ZNF135	154.9268997	-0.681617119	0.220016523	-3.098026951	0.001948137	0.00947024
ZNF217	588.9902184	0.624909782	0.138072887	4.525941316	6.01E-06	9.84E-05
ZNF257	21.21181844	-1.279707731	0.323197371	-3.959523948	7.51E-05	0.00072928
ZNF286A	474.6546685	-0.593788711	0.123211661	-4.819257418	1.44E-06	3.19E-05
ZNF300	402.5647961	-0.747633803	0.169636384	-4.407272682	1.05E-05	0.00015486
ZNF385D	91.99563946	1.217896779	0.284064975	4.287388056	1.81E-05	0.00024106
ZNF423	443.0640321	-0.995662399	0.186175443	-5.347979205	8.89E-08	3.44E-06
ZNF469	196.9069149	-0.597331829	0.212707044	-2.808237184	0.004981352	0.01970491
ZNF488	401.9613181	-2.444381105	0.350364401	-6.976682272	3.02E-12	7.43E-10
ZNF536	95.96752439	-1.411115843	0.278157576	-5.073080748	3.91E-07	1.13E-05
ZNF600	62.58135771	0.669994912	0.138686197	4.831013663	1.36E-06	3.06E-05
ZNF681	151.1084852	-1.168654713	0.179713166	-6.502888688	7.88E-11	1.12E-08
ZNF704	170.5533034	-0.905897064	0.177630703	-5.099890098	3.40E-07	1.01E-05

ZNF711	759.3833833	-0.640430479	0.149490956	-4.284075066	1.84E-05	0.00024259
ZNF74	382.1431025	-0.626537976	0.144378767	-4.33954376	1.43E-05	0.00019788
ZNF804A	90.07613476	-1.326546945	0.266753801	-4.972926117	6.59E-07	1.73E-05
ZNRF2	347.1221903	0.638687954	0.118709438	5.380262621	7.44E-08	2.98E-06
